# Alkynyl Moiety for Triggering 1,2‐Metallate Shifts: Enantiospecific sp^2^–sp^3^ Coupling of Boronic Esters with *p*‐Arylacetylenes

**DOI:** 10.1002/anie.201703894

**Published:** 2017-07-12

**Authors:** Venkataraman Ganesh, Marcin Odachowski, Varinder K. Aggarwal

**Affiliations:** ^1^ School of Chemistry University of Bristol Bristol BS8 1TS UK

**Keywords:** 1,2-metallate rearrangement, organoboron, phenylacetylenes, sp^2^–sp^3^ coupling, stereospecific reactions

## Abstract

The enantiospecific coupling of secondary and tertiary boronic esters to aromatics has been investigated. Using p‐lithiated phenylacetylenes and a range of boronic esters coupling has been achieved by the addition of N‐bromosuccinimide (NBS). The alkyne functionality of the intermediate boronate complex reacts with NBS triggering the 1,2‐migration of the group on boron to carbon giving a dearomatized bromoallene intermediate. At this point elimination and rearomatization occurs with neopentyl boronic esters, giving the coupled products. However, using pinacol boronic esters, the boron moiety migrates to the adjacent carbon resulting in formation of ortho boron‐incorporated coupled products. The synthetic utility of the boron incorporated product has been demonstrated by orthogonal transformation of both the alkyne and boronic ester functionalities.

For over half a century, cross‐coupling reactions, particularly the Suzuki–Miyaura reaction, have been widely used in synthesis with applications spanning pharmaceuticals, agrochemicals and materials.^1^ However, although extraordinarily useful for sp^2^–sp^2^ coupling, this reaction shows rather limited scope for aliphatic boron reagents. Primary organoboron reagents work well, but apart from a few specific examples^2^ (chiral) secondary and tertiary boronic esters do not. Recently, we reported a unique approach to the stereospecific sp^2^–sp^3^ coupling of boronic esters by exploiting the reaction of boronate complexes with electrophiles (Scheme 1 a).^3^ The coupling reaction worked well with electron rich heteroaromatics and aromatics bearing donor groups in the *meta*‐position. However, without such features no coupling occurred and bromination at the sp^3^ center occurred instead (Scheme 1 a).^4^

**Scheme 1:**
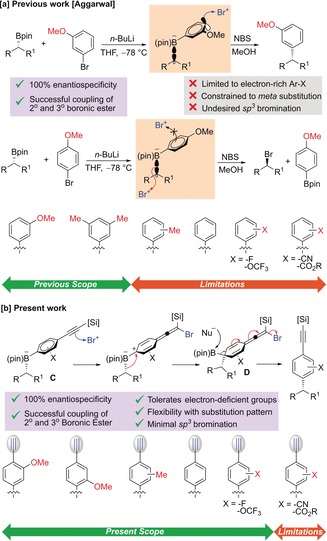
General mechanism of metal‐catalyzed sp^2^–sp^3^ coupling of boronic esters.

In order to broaden the substrate scope to an even greater range of aromatics, we envisaged the introduction of a functional group *exo* to the aromatic ring that would be more reactive than the sp^3^ center, and still trigger the 1,2‐metallate shift. We considered the use of alkynes because they should react with electrophiles in the desired way and because of the ease with which they can be transformed into a variety of other functional groups.^5^ Furthermore, alkynes are an important substituent in their own right owing to their prominence in natural products and as a site for rapid and site‐selective conjugation, through a variety of Click reactions.^6^ We hypothesized that treatment of the TMS‐phenylacetylene derived boronate complex (**C**) with NBS should result in bromination of the alkyne^7^ which would trigger 1,2‐metallate shift^8^ leading to a dearomatized bromoallene intermediate (**D**) (Scheme 1 b).^8^ Upon reaction with a nucleophile, elimination and rearomatization would result.^5^

Here, we describe the realization of this hypothesis. To test our idea, we chose cyclohexyl pinacol boronic ester (CyBpin, **1 a**) and TMS‐*p*‐bromophenylacetylene (**2 a**) as standard substrates. Treatment of bromoalkyne **2 a** with *n*‐BuLi in THF at −78 °C followed by CyBpin gave boronate complex **3 a**. Subsequent addition of NBS in MeOH afforded a mixture of products comprising the desired coupled product **4 a** (40 %), a product with boron incorporation in the *ortho*‐position **4 b** (52 %) as well as a small amount of **5** (6 %) along with Cy−Br formed through the direct bromination at the sp^3^ carbon (Scheme 2, entry 1). At this point, we decided to optimize the reaction conditions to maximize the formation of either **4 a** or **4 b**, initially focusing on the maximally functionalized boron‐incorporated product **4 b**. We had previously observed such products when coupling electron‐rich aromatics with boronic esters and found that *i*PrOH/MeCN gave the best ratio.3b We therefore carried out a brief solvent study (entries 2–5) and again found that *i*PrOH/MeCN was optimal here too, giving the highest ratio, leading to a 76 % yield of **4 b** (entry 4). In THF/MeCN the reaction predominantly favored the undesired sp^3^ bromination pathway, showing the need for an alcohol co‐solvent (entry 3).

**Scheme 2:**
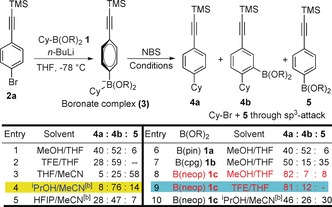
General scheme and optimization of reaction conditions.^[a]^ [a] Reaction conditions: *p*‐bromophenylacetylene **2 a** (1.1 equiv), *n*‐BuLi (1.1 equiv) in THF (0.3 m) at −78 °C for 1 h, then **1 a**–**c** (1.0 equiv) in THF (0.3 m) at −78 °C, then at 0 °C addition of NBS (1.5 equiv) in specified solvent (0.3 m). Yields were determined by ^1^H‐NMR spectroscopy. [b] Solvent exchange. cpg: *cis*‐1,2‐cyclopentyl glycol.

In order to promote the formation of the de‐borinated coupled product **4 a**, we needed to promote nucleophilic attack at the boron atom and so decided to tune the steric environment around the boron center with a variety of diol ligands. Of the diols tested, the least hindered neopentyl glycol gave the highest selectivity for the coupled product **4 a** (82 %) with minimal amounts of **4 b** and **5** (entry 8). With increasing steric hindrance around boron, an increasing proportion of the boron incorporation product **4 b** was observed. Additional solvent screening showed that in TFE/THF, the sp^3^ bromination pathway could be eliminated (entry 9).

Using the optimized conditions for creating boron‐free products we explored the substrate scope of the aromatic component, employing a range of arylalkynes with a standard secondary boronic ester **6 a** obtained in 96:4 er using our lithiation–borylation methodology (Table 1).^9^ With simple *p*‐bromophenylalkyne **2 a**, the reaction furnished the expected coupled product **7 a** in 92 % yield and with 100 % enantiospecificity. With alkyl substituents in the *ortho*‐ (**2 b**) and *meta*‐positions (**2 c**) the desired product **7 b** and **7 c** were obtained in 85 % and 86 % yield, respectively. Similarly, the naphthylalkyne **2 d** also afforded the expected coupled product **7 d** in good yield (89 %) (minor amounts (≈5 %) of boron incorporation was observed in all cases). Electron‐donating substituents on the aromatic ring (**2 e** and **2 f**) smoothly afforded the coupled products **7 e** and **7 f** in excellent yields (82 and 90 % respectively). However, the introduction of electron‐withdrawing groups such as fluoro (**2 g**) or trifluoromethoxy (**2 h**) on the aromatic ring favored the direct sp^3^ bromination pathway (≈9:1) with neopentyl boronic esters, so the corresponding pinacol boronic esters were tested.

**Table 1:** Scope of NBS‐mediated coupling of phenylacetylenes with secondary boronic ester.^[a]^ 
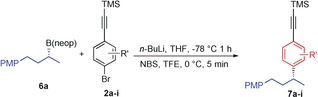

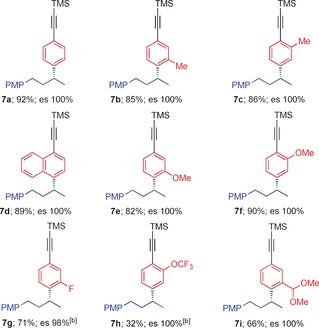

[a] Reaction conditions: *p*‐bromophenylacetylenes **2 a**–**i** (1.1 equiv), *n*‐BuLi (1.1 equiv) in THF (0.3 m) at −78 °C for 1 h, then **6 a** (1.0 equiv) in THF (0.3 m) at −78 °C, then at 0 °C NBS (1.5 equiv) in TFE (0.3 m) was added. [b] B(pin) **9 a** was used.

In comparison to pinacol, it is known that neopentyl boronic esters promote undesired S_E_2 reaction at the sp^3^ carbon.^10^ Pleasingly, with **2 g**, and the pinacol boronic ester **9 a** the coupled product **7 g** was obtained in 71 % yield. With trifluoromethoxy **2 h**, the desired product **7 h** was obtained in a modest yield of 32 % together with undesired direct bromination at the sp^3^ carbon (in 2:3 ratio) and minor amounts of boron incorporated products (≈10 %). With other strongly electron withdrawing groups for example, CF_3_, CN, CO_2_^t^Bu bromination of the sp^3^ carbon dominated over the attack on the deactivated aromatic ring. A dimethylacetal functionality **2 i** (representing a masked aldehyde) reacted efficiently with the corresponding neopentyl boronic ester to provide the coupled product **7 i** in 66 % yield. In all cases the reactions occurred with complete enantiospecificity.

We then turned our attention to the scope of secondary and tertiary neopentyl boronic esters in our coupling chemistry (Table 2). Secondary boronic esters bearing alkyl, alkenyl, cyclopropyl and silyl ether functionalities **6 a**–**d** and natural product‐derived boronic ester **6 e** smoothly converted to the corresponding coupled product **7 a**, **8 b**–**e** in good yields and 100 % es. Other commonly occurring functional groups were tolerated in the boronic ester including azide (**6 f**) and carbamate (**6 g**). With tertiary neopentyl boronic esters **6 h** and **6 i**, the reaction proceeded smoothly to furnish the coupled products **8 h** and **8 i** in 43 % and 79 % yield, respectively.

**Table 2:** Scope of NBS‐mediated coupling of Bneop esters with **2 a**.^[a]^ 
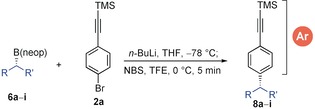

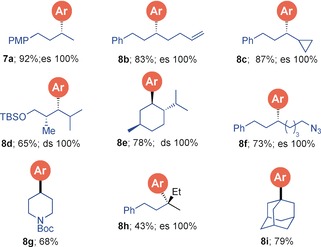

[a] Reaction conditions: *p*‐bromophenylacetylene **2 a** (1.1 equiv), *n*‐BuLi (1.1 equiv) in THF (0.3 m) at −78 °C for 1 h; then **6 a**–**i** (1.0 equiv) in THF (0.3 m) at −78 °C; then NBS (1.5 equiv) in TFE (0.3 m).

We then turned to exploring the scope for the boron incorporation using pinacol boronic esters using the identified conditions (Scheme 2, entry 4). Reaction of boronic ester **9 a** with **2 a** gave the expected boron‐incorporated product **10 a** in 78 % yield with 100 % es (Table 3). On a gram‐scale under standard reaction conditions, **10 a** was obtained in 66 % yield. Similarly, with other electron‐rich phenylacetylenes **2 b**,**c** and **2 f,** the reaction proceeded smoothly to provide the corresponding products **10 ab**, **10 ac** and **10 af** in good yields. In the case of **2 c** and **2 f**, a regioisomeric mixture of products (**10 ac1**–**c2** and **10 af1**–**f2**) were observed. With other electron‐withdrawing groups (e.g. CF_3_, CN, CO_2_^t^Bu, F and OCF_3_) on the aromatic ring, in MeCN/*i*PrOH solvent, bromination at the sp^3^ carbon was favored (>95 %) over bromination of the deactivated phenylacetylene.

**Table 3:** Scope of NBS‐mediated coupling of boronic esters with phenylacetylenes providing boron‐incorporated products. 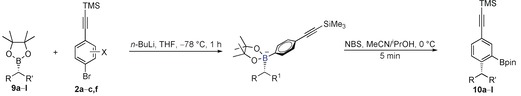

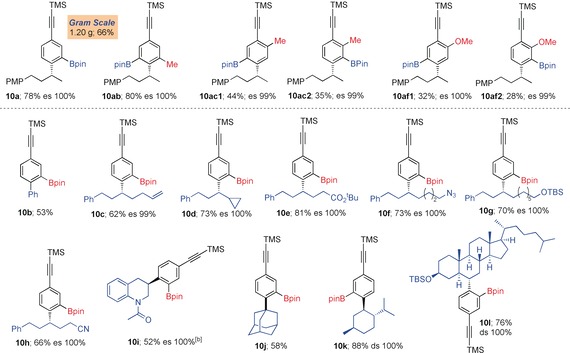

[a] Reaction conditions: *p*‐bromophenylacetylene **2 a**–**c**,**f** (1.1 equiv), *n*‐BuLi (1.1 equiv) in THF (0.3 m) at −78 °C for 1 h, then **9 a**–**l** (1.0 equiv) in THF (0.3 m) at −78 °C, then solvent exchange to ^*i*^PrOH followed by addition of NBS (1.5 equiv) in MeCN (0.3 m). [b] Isolated as phenol after oxidation with H_2_O_2_/NaOH.

The scope of secondary pinacol boronic esters was also investigated (Table 3). An array of aromatic, secondary and tertiary boronic esters bearing phenyl, alkyl, alkenyl, cyclopropyl, ester, azido, silylether, nitrile and amide^11^ functional groups **9 b**–**j** all worked well furnishing the products **10 b**–**j** in good yield (52–81 %) and 100 % es. Natural product‐derived boronic esters **9 k** and **9 l** were transformed exclusively to the boron incorporated product **10 k** and **10 l** in 88 % and 76 % yields, respectively with complete diastereospecificity.

The mechanism that accounts for the generation of the boron‐free and boron‐incorporated products is shown in Scheme 3. Following the formation of boronate complex **I**, the reaction with NBS leads to the bromoallene intermediate **II**. If the boronic ester is unhindered, subsequent attack by MeOH at boron promotes elimination leading to product **Va** (**Path a**). In contrast, if the boronic ester is hindered, nucleophilic attack is less favored, especially with *i*PrOH as solvent, and migration of the boron to the adjacent carbon occurs instead, relieving steric encumbrance and eliminating bromide. This leads to carbocation intermediate **IV**, which then eliminates to the product **Vb** (**Path b**).

**Scheme 3:**
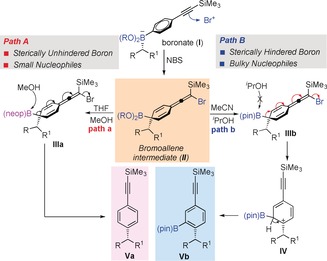
Plausible mechanism for sp^2^–sp^3^ coupling and boron incorporation.

The boron incorporated products provide a rich source of functionality which can be chemoselectively converted into a range of diverse products (Scheme 4). Using K_2_CO_3_/MeOH the orthogonal deprotection of the TMS group was achieved providing the terminal alkyne **11 a** in 87 % yield.^12^ Hydration of **10 a** with 20 mol % triflic acid in TFE furnished ketone **11 b** in 76 % yield.^13^ Under standard CuAAC conditions,^14^ **11 a** was transformed to the corresponding triazole product **11 c** in 85 % yield. Oxidation of the boronic ester with H_2_O_2_/NaOH and hydroxylamine sulfonic acid (HSA)^15^ gave the desired phenol **11 d** and aniline **11 e** in 90 and 62 % respectively. Under standard Sonagashira conditions with iodobenzene, **11 a** smoothly converted to the functionalized alkyne **11 f** in 90 % yield.

**Scheme 4:**
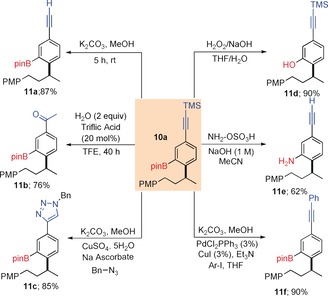
Synthetic transformations of product **10 a**.

In summary, we have successfully developed an efficient enantiospecific sp^2^–sp^3^ coupling of a range of aromatic alkynes with a broad range of enantioenriched boronic esters. The alkyne acts as a reactive handle for reaction with NBS which triggers the coupling process. Importantly, conditions were found which either lead to the coupled product or to the coupled product bearing an *ortho* boronic ester. The maximally functionalized product is highly versatile as each functional group can be transformed chemoselectively making it an ideal intermediate in synthesis.

## Conflict of interest

The authors declare no conflict of interest.

## Supporting information

As a service to our authors and readers, this journal provides supporting information supplied by the authors. Such materials are peer reviewed and may be re‐organized for online delivery, but are not copy‐edited or typeset. Technical support issues arising from supporting information (other than missing files) should be addressed to the authors.

Supplementary

## References

[1a] N. Miyaura , A. Suzuki , Chem. Rev. 1995, 95, 2457–2483;

[1b] A. Suzuki , Angew. Chem. Int. Ed. 2011, 50, 6722–6737; 10.1002/anie.201101379 21618370

[2a] D. Imao , B. W. Glasspoole , V. r. S. Laberge , C. M. Crudden , J. Am. Chem. Soc. 2009, 131, 5024–5025; 19301820 10.1021/ja8094075

[2b] D. L. Sandrock , L. Jean-Gérard , C.-y. Chen , S. D. Dreher , G. A. Molander , J. Am. Chem. Soc. 2010, 132, 17108–17110; 21077687 10.1021/ja108949w PMC3045699

[2c] T. Awano , T. Ohmura , M. Suginome , J. Am. Chem. Soc. 2011, 133, 20738–20741; 22129167 10.1021/ja210025q

[2d] L. Li , S. Zhao , A. Joshi-Pangu , M. Diane , M. R. Biscoe , J. Am. Chem. Soc. 2014, 136, 14027–14030; 25226092 10.1021/ja508815w PMC4195375

[2e] D. Leonori , V. K. Aggarwal , Angew. Chem. Int. Ed. 2015, 54, 1082–1096; 10.1002/anie.201407701 25414056

[2f] C.-Y. Wang , J. Derosa , M. R. Biscoe , Chem. Sci. 2015, 6, 5105–5113. 26388985 10.1039/c5sc01710f PMC4571484

[3a] A. Bonet , M. Odachowski , D. Leonori , S. Essafi , V. K. Aggarwal , Nat. Chem. 2014, 6, 584–589; 24950327 10.1038/nchem.1971

[3b] M. Odachowski , A. Bonet , S. Essafi , P. Conti-Ramsden , J. N. Harvey , D. Leonori , V. K. Aggarwal , J. Am. Chem. Soc. 2016, 138, 9521–9532. 27384259 10.1021/jacs.6b03963 PMC5063455

[4] R. Larouche-Gauthier , T. G. Elford , V. K. Aggarwal , J. Am. Chem. Soc. 2011, 133, 16794–16797. 21939203 10.1021/ja2077813

[5] Alkenes can also be used in place of alkynes but reactions are not as clean or high yielding (48 % yield) as the bromohydrin methyl ether was also formed from further bromination of the alkene and trapping by MeOH. See the Supporting Information for details.

[6a] J. Lam , Chemistry and biology of naturally-occurring acetylenes and related compounds (NOARC): proceedings of a Conference on the Chemistry and Biology of Naturally-Occurring Acetylenes and Related Compounds (NOARC), Elsevier, Amsterdam, 1988;

[6b] H. C. Kolb , M. G. Finn , K. B. Sharpless , Angew. Chem. Int. Ed. 2001, 40, 2004–2021; 10.1002/1521-3773(20010601)40:11<2004::AID-ANIE2004>3.0.CO;2-5 11433435

[7] D. Yue , N. Della Cà , R. C. Larock , Org. Lett. 2004, 6, 1581–1584. 15128241 10.1021/ol049690s

[8a] G. M. Davies , P. S. Davies , W. E. Paget , J. M. Wardleworth , Tetrahedron Lett. 1976, 17, 795–798;

[8b] A. B. Levy , J. Org. Chem. 1978, 43, 4684–4685;

[8c] I. Akimoto , A. Suzuki , Synthesis 1979, 146–147;

[8d] E. R. Marinelli , A. B. Levy , Tetrahedron Lett. 1979, 20, 2313–2316;

[8e] J. Kagan , S. K. Arora , Tetrahedron Lett. 1983, 24, 4043–4046;

[8f] A. Pelter , H. Williamson , G. M. Davies , Tetrahedron Lett. 1984, 25, 453–456;

[8g] M. Ishikura , H. Kato , Tetrahedron 2002, 58, 9827–9838.

[9a] J. L. Stymiest , G. Dutheuil , A. Mahmood , V. K. Aggarwal , Angew. Chem. Int. Ed. 2007, 46, 7491–7494; 10.1002/anie.200702146 17659521

[9b] J. L. Stymiest , V. Bagutski , R. M. French , V. K. Aggarwal , Nature 2008, 456, 778–782; 19079057 10.1038/nature07592

[9c] R. Larouche-Gauthier , C. J. Fletcher , I. Couto , V. K. Aggarwal , Chem. Commun. 2011, 47, 12592–12594; 10.1039/c1cc14469c 21892499

[9d] A. P. Pulis , D. J. Blair , E. Torres , V. K. Aggarwal , J. Am. Chem. Soc. 2013, 135, 16054–16057. 24138162 10.1021/ja409100y

[10] K. Feeney , G. Berionni , H. Mayr , V. K. Aggarwal , Org. Lett. 2015, 17, 2614–2617. 25973673 10.1021/acs.orglett.5b00918

[11] K. Kubota , Y. Watanabe , H. Ito , Adv. Synth. Catal. 2016, 358, 2379–2384.

[12] U. Dutta , S. Maity , R. Kancherla , D. Maiti , Org. Lett. 2014, 16, 6302–6305. 25401303 10.1021/ol503025n

[13] W. Liu , H. Wang , C.-J. Li , Org. Lett. 2016, 18, 2184–2187. 27082159 10.1021/acs.orglett.6b00801

[14] F. Himo , T. Lovell , R. Hilgraf , V. V. Rostovtsev , L. Noodleman , K. B. Sharpless , V. V. Fokin , J. Am. Chem. Soc. 2005, 127, 210–216. 15631470 10.1021/ja0471525

[15] S. Voth , J. W. Hollett , J. A. McCubbin , J. Org. Chem. 2015, 80, 2545–2553. 25594547 10.1021/jo5025078

