# Supplementary material for: Alkynyl Moiety for Triggering 1,2‐Metallate Shifts: Enantiospecific sp2–sp3 Coupling of Boronic Esters with p‐Arylacetylenes
Source: Angew Chem Int Ed Engl. 2017 Jul 12;56(33):9752–6. doi: 10.1002/anie.201703894 (PMC5577510; doi:10.1002/anie.201703894)
Supplement: Supplementary file 1 — Supplementary [file ANIE-56-9752-s001.pdf]

Angewandte Chemie
Eine Zeitschrift der Gesellschaft Deutscher Chemiker

Supporting Information

Alkynyl Moiety for Triggering 1,2-Metallate Shifts: Enantiospecific sp^2–sp^3 Coupling of Boronic Esters with *p*-Arylacetylenes

*Venkataraman Ganesh, Marcin Odachowski, and Varinder K. Aggarwal**

anie_201703894_sm_miscellaneous_information.pdf

Contents

1. General Information S2
2. Reaction Optimization S3
3. General Procedures and Characterization Data S5
4. ^{1}H, ^{13}C, ^{19}F and 2D NMR trace S45
5. References S108

1. General Information

All required fine chemicals were used directly without purification unless stated otherwise. All solvents were commercially supplied or provided by the communal stills of the School of Chemistry, University of Bristol. All air– and water–sensitive reactions were carried out in flame–dried glassware under nitrogen atmosphere using standard Schlenk manifold technique. Cryogenic temperature was achieved using an acetone/CO_2 cold bath.

^{1}H, ^{13}C, ^{19}F and 2D Nuclear Magnetic Resonance (NMR) spectra were acquired at various field strengths as indicated, and were referenced to $CHCl_3$ (7.26 and 77.0 ppm for ^{1}H and ^{13}C, respectively) or TMS (0.00 ppm for ^{1}H and ^{13}C). ^{1}H NMR coupling constants are reported as observed and refer to apparent multiplicities and not true coupling constants. Data are reported as follows: chemical shift, multiplicity (s = singlet, br s = broad singlet, d = doublet, t = triplet, q = quartet, qi = quintet, sx = sextet, sp = septet, m = multiplet, dd = doublet of doublets, etc.) and integration. High resolution mass spectra (HRMS) were recorded on a Brüker Daltonics MicroTOF II by Electrospray Ionisation (ESI) or on a VG Micromass Autospec (Triple–sector) by Electron Impact (EI). All IR data was obtained on a Perkin–Elmer Spectrum One FT–IR spectrometer. Optical rotations were obtained on a Perkin–Elmer 241MC polarimeter. Optical rotation ($[\alpha]_D^T$) was measured on a Bellingham and Stanley Ltd. ADP220 polarimeter.

Analytical TLC: aluminium–backed plates pre–coated (0.25 mm) with Merck Silica Gel 60 F254. Compounds were visualized by exposure to UV–light or by dipping the plates in permanganate ($KMnO_4$) stain followed by heating. Flash column chromatography was performed using Merck Silica Gel 60 (40–63 μm). All mixed solvent eluents are reported as v/v solutions. Chiral HPLC was performed using Diacel Chiralpak IA, IB and IC columns (4.6 × 250 mm × 5 μm) fitted with the respective guards (4 × 10 mm) and monitored by DAD (Diode Array Detector). Enantiospecificity: es = 100 × (ee product) / (ee reactant). GC–MS was performed on an Agilent 7820A using a HP–5MS UI column (30 m × 0.25 mm × 0.25 μm).

n–BuLi was purchased from Acros. The molarity of *n*–BuLi solution was determined by titration using *N*–benzyl benzamide as an indicator. *N*–bromosuccinimide was recrystallized from water.

2. Reaction Optimization

Attempts with Styrenes as a Handle for 1,2-metallate shift

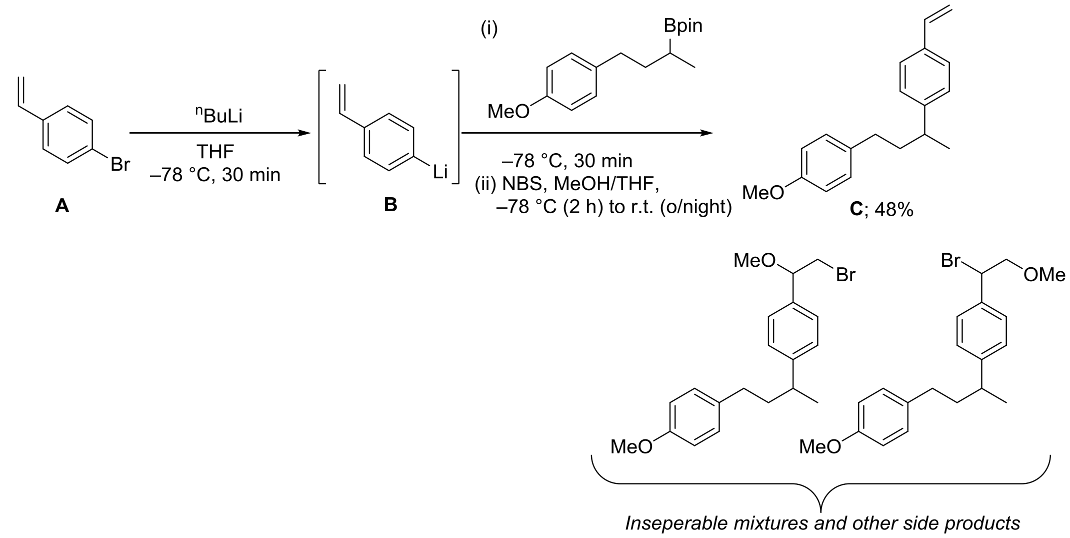

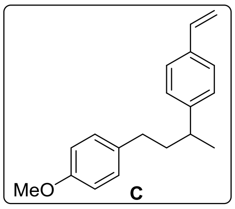

1-Methoxy-4-(3-(4-vinylphenyl)butyl)benzene: To a stirred solution of 4-bromostyrene (**A**, 34 μL, 0.26 mmol, 1.3 eq) in THF (2.0 mL) under N_2 at –78 °C was added *n*-butyllithium (1.6 M in hexane, 0.16 mL, 0.26 mmol, 1.3 eq). The reaction was stirred for 30 min, before a solution of the boronic ester **9a** (58 mg, 0.20 mmol, 1.0 eq) in THF (0.50 mL) was added. The reaction mixture was stirred for a further 30 min at –78 °C, at which point ^{11}B NMR spectroscopy showed formation of the ate complex [(^{11}B NMR, 96 Hz, THF) δ_B ~ 7 ppm]. A solution of *N*-bromosuccinimide (53 mg, 0.30 mmol, 1.5 eq) in MeOH (1.0 mL) was added dropwise, and the reaction was stirred at –78 °C for 2 h before warming to r.t. overnight. The mixture was quenched by the addition of H_2O/Et_2O (1:1 v/v, 10 mL). The aqueous phase was extracted with Et_2O (2 x 10 mL), the combined organic layers were washed with brine (10 mL), dried ($MgSO_4$), filtered and concentrated *in vacuo*. The crude product was purified by FCC (petroleum ether/Et_2O = 100/0 to 95/5) to afford the title compound **C** as a pale yellow oil in 58% yield (31 mg). R_f (90/10 petroleum ether/Et_2O): 0.39; IR (film) ν_{max}/cm^{-1}: 2926, 1611, 1510, 1455, 1299, 1242, 1176, 1036, 990, 904, 839, 821; ^{1}H NMR ($CDCl_3$, 400 MHz) 7.37 (d, *J* = 8.5 Hz, 2H), 7.17 (d, *J* = 8.5 Hz, 2H), 7.05 (d, *J* = 8.5 Hz, 2H), 6.82 (d, *J* = 8.5 Hz, 2H), 6.73 (dd, *J* = 17.5, 11.0 Hz, 1H), 5.74 (dd, *J* = 17.5, 1.0 Hz, 1H), 5.22 (dd, *J* = 11.0, 1.0 Hz, 1H), 3.80 (s, 3H), 2.73 (tq, *J* = 7.5, 7.0 Hz, 1H), 2.54 – 2.41 (m, 2H), 1.99 – 1.80 (m, 2H), 1.28 (d, *J* = 7.0 Hz, 3H); ^{13}C NMR ($CDCl_3$, 100 MHz) δ (ppm): 157.7, 147.1, 136.7, 135.4, 134.5, 129.2, 127.3, 126.3, 113.7, 112.9, 55.2, 40.1, 39.2, 33.0, 22.5; HRMS (EI) exact mass calculated for $[M]^+$ $C_{19}H_{22}O$ requires 266.1665, found 266.1677.

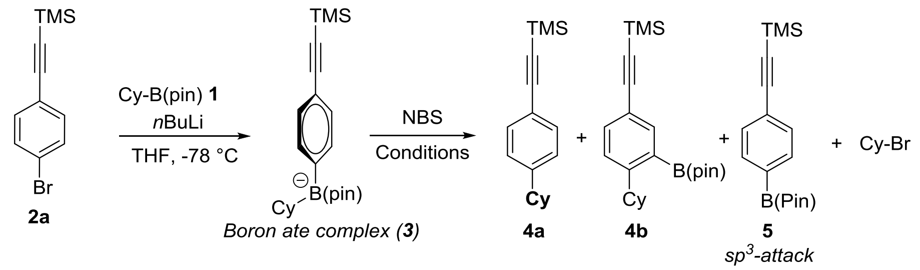

Solvent	*T °C*	*Reagent*	*Equiv. NBS*	*Ratio (4a/4b)*	*Combined Yield**	*Comments*
THF	–78 °C (1 h)	NBS/MeOH	2.0	–	NR	
THF	–20 °C (12 h)	NBS/MeOH	2.0	100/0	36%	
THF	0 °C (12 h)	NBS/MeOH	2.0	100/0	30%	
THF	0 °C (5 min)	NBS/MeOH	1.5	37/63	75%	sp^3 attack (20%)
Toluene	0 °C (5 min)	NBS/MeOH	2.0	–	–	sp^3 attack (100%)
Ether	0 °C (5 min)	NBS/MeOH	2.0	55/45	22%	
DCM[a]	0 °C (5 min)	NBS/MeOH	2.0	75/25	59%	
DCE[a]	0 °C (5 min)	NBS/MeOH	2.0	45/55	63%	
Dioxane	rt (5 min)	NBS/MeOH	2.0	57/43	49%	
DMF[a]	0 °C (5 min)	NBS/MeOH	2.0	45/55	48%	sp^3 attack (20%)
MeCN[a]	0 °C (5 min)	NBS/MeOH	2.0	45/55	69%	
MeCN[a]	0 °C (5 min)	NBS/^{i}PrOH	1.5	10/90	84%	sp^3 attack (14%)
THF	0 °C (5 min)	NBS/TFE	1.5	22/78	90%	
MeCN	0 °C (5 min)	NBS/HFIP	1.5	38/62	75%	sp^3 attack (7%)
THF	0 °C (5 min)	NBS/ MeOH	2.0	58/42	86%	NaOMe–50%
THF	0 °C (5 min)	NBS/MeCN	1.5	17/83	30%	sp^3 attack (58%)

TFE: Trifluoroethanol; HFIP: Hexafluoroisopropyl alcohol.

Steric Effect

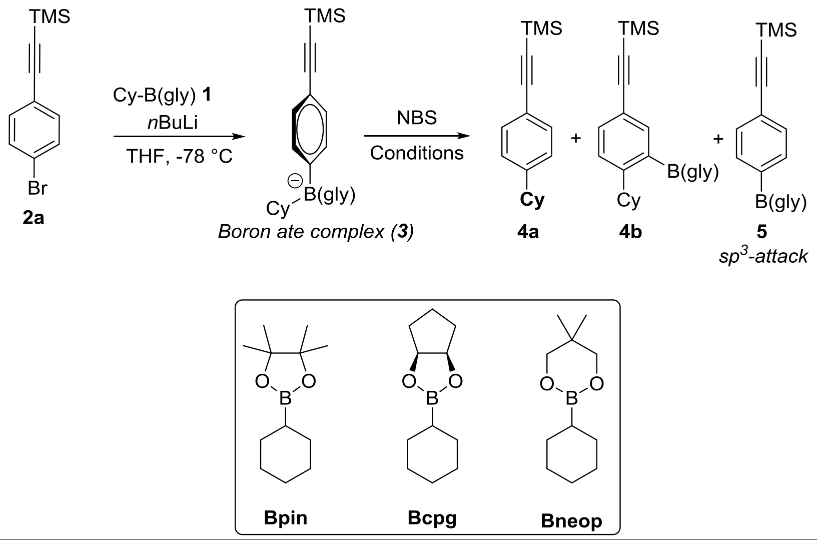

Glycol	*T °C*	*Reagent*	*Equiv.*	*Ratio (4a/4b)*	*Combined Yield**	*Comments*
Bpin	0 °C (5 min)	NBS/MeOH	1.5	43/57	92%	*sp*³ attack (24%)
Bcpg	0 °C (5 min)	NBS/MeOH	1.5	77/23	65%	*sp*³ attack (35%)
Bneop	0 °C (5 min)	NBS/MeOH	1.5	92/8	89%	*sp*³ attack (8%)
Bneop	0 °C (5 min)	NBS/TFE	1.5	87/13	93%	

Reaction conditions: *p*–Bromophenylacetylene **2a** (1.1 equiv), *n*–BuLi (1.1 equiv) in THF (0.3 M) at –78 °C for 1 h, then **1a–c** (1.0 equiv) in THF (0.3 M) at –78 °C, then at 0 °C addition of NBS (1.5 equiv) in specified solvent (0.3 M).[a]Solvent Exchange.* Determined by ^{1}H NMR analysis of the crude mixture.

Starting Material Synthesis

A. General Procedure for the preparation of TMS–*p*–bromophenylacetylenes:

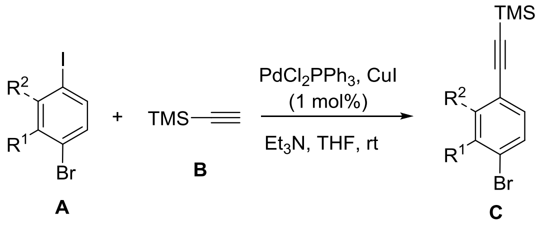

A solution of *p*–iodobromoarene **A** (1 mmol, 1.0 equiv) in THF (5 mL, 0.2 M) at rt was treated with $PdCl_2(PPh_3)_2$ (0.01 mmol, 1 mol%) and CuI (0.01 mmol, 1 mol%). To this mixture Et_3N (5 mmol, 5 equiv) was added and stirred followed by dropwise addition of TMS–acetylene **B** (1.1 mmol, 1.1 equiv) at the same temperature. The mixture was stirred for 3 h and the crude mixture was diluted with Et_2O and filtered through a pad of silica. The filtrate was

concentrated in vacuo and crude material was adsorbed on silica and purified by flash column chromatography on silica gel eluting with *n*–hexane/Et_2O (hexane 100% to Et_2O/Hex 5%).

((4–Bromonaphthalen–1–yl)ethynyl)trimethylsilane (2d): 1–bromo–4–iodonaphthalene (1 g, 3 mmol) was subjected to the General Procedure A. The crude product was purified by column chromatography (Et_2O/hexane–2%) to afford the title compound **2d** as a colourless oil in 89% yield (812 mg). R_f (95/5 petroleum ether/EtOAc): 0.8; IR (film) ν_{max}/cm^{-1}: 2958, 1248, 836, 756, 653; ^{1}H NMR (400 MHz, $CDCl_3$) 8.40 – 8.33 (m, 1H), 8.28 – 8.21 (m, 1H), 7.71 (d, *J* = 7.7 Hz, 1H), 7.66 – 7.59 (m, 2H), 7.53 (d, *J* = 7.7 Hz, 1H), 0.36 (s, 9H). ^{13}C NMR (101 MHz, $CDCl_3$) 134.50, 131.80, 130.87, 129.42, 127.89, 127.70, 127.60, 126.89, 123.99, 121.02, 102.39, 100.82, 0.16. HRMS (EI) exact mass calculated for [M$^+$] $C_{15}H_{15}BrSi$ requires 302.0126, found 302.0130.

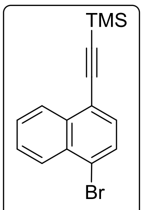

2d

((4–bromo–3–methoxyphenyl)ethynyl)trimethylsilane (2e): 4–Bromo–1–iodo–3–methoxybenz–ene (1 g, 3.2 mmol) was subjected to the General Procedure A. The crude product was purified by column chromatography (Et_2O/hexane–4%) to afford the title compound **2e** as a colourless oil in 85% yield (767 mg). R_f (95/5 petroleum ether/EtOAc): 0.5; IR (film) ν_{max}/cm^{-1}: 2959, 2899, 1477, 1391, 1248, 838, 758; ^{1}H NMR (400 MHz, $CDCl_3$) 7.45 (d, *J* = 8.0 Hz, 1H), 6.97 (d, *J* = 1.7 Hz, 1H), 6.94 (dd, *J* = 8.0, 1.7 Hz, 1H), 3.90 (s, 3H), 0.25 (s, 9H). ^{13}C NMR (126 MHz, $CDCl_3$) 155.47, 133.10, 125.42, 123.35, 115.00, 112.52, 104.07, 95.10, 56.23, −0.13. HRMS (EI) exact mass calculated for [M$^+$] $C_{12}H_{15}OSiBr$ requires 282.0076, found *m/z* 282.0081.

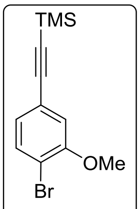

2e

((4–bromo–2–methoxyphenyl)ethynyl)trimethylsilane (2f) : 4–Bromo–1–iodo–2–methoxybenz–ene (1 g, 3.2 mmol) was subjected to the General Procedure A. The crude product was purified by column chromatography (Et_2O/hexane–4%) to afford the title compound **(2f)** as a colourless oil in 77% yield (698 mg). R_f (95/5 petroleum ether/EtOAc): 0.5; IR (film) ν_{max}/cm^{-1}: 2958, 2899, 1583, 1485, 1249, 835, 758; ^{1}H NMR (400 MHz, $CDCl_3$) 7.28 (d, *J* = 8.1 Hz, 1H), 7.03 (dd, *J* = 8.1, 1.8 Hz, 1H), 6.99 (d, *J* = 1.8 Hz, 1H), 3.87 (s, 3H), 0.26 (s, 9H). ^{13}C NMR (126 MHz, $CDCl_3$) 160.71, 134.86, 123.52, 123.39, 114.36, 111.48, 100.10, 99.73, 56.09, −0.02. HRMS (EI) exact mass calculated for [M$^+$] $C_{12}H_{15}OSiBr$ requires 282.0076, found 282.0070.

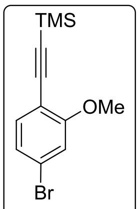

2f

((4–bromo–2–(trifluoromethoxy)phenyl)ethynyl)trimethylsilane (2h): 4–bromo–2–(trifluoromethoxy)–1–iodobenzene (1 g, 2.73 mmol) was subjected to the General Procedure A. The crude product was purified by column chromatography (Et_2O/hexane–4%) to afford the title compound **2h** as a colourless oil in 67% yield (723 mg). R_f (95/5 petroleum ether/EtOAc): 0.72; IR (film) ν_{max}/cm^{-1}: 2962, 1481, 1247, 1208, 1168, 839, 759; ^{1}H NMR (301 MHz, $CDCl_3$) 7.43 – 7.35 (m, 3H), 0.24 (s, 9H). ^{13}C NMR (76 MHz, $CDCl_3$) 149.97, 134.52, 130.13, 124.88, 122.53, 120.49 (q, J = 429 Hz), 117.24, 102.21, 97.69, –0.31. ^{19}F NMR (283 MHz, $CDCl_3$) −57.60. HRMS (EI) exact mass calculated for [M$^+$] $C_{12}H_{12}BrF_3OSi$ requires 335.9793, found 335.9797.

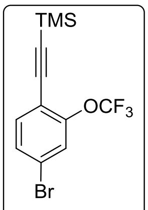

2h

((4–bromo–3–(dimethoxymethyl)phenyl)ethynyl)trimethylsilane (2i): 1,4–bromo–3–(dimethoxy methyl)benzene (1 g, 3.25 mmol) was subjected to the General Procedure A. The reaction was carried out at 40 °C. The crude product was purified by column chromatography (Et_2O/hexane–4%) to afford the title compound **2i** as a colourless oil in 67% yield (709 mg). R_f (95/5 petroleum ether/EtOAc): 0.45; IR (film) ν_{max}/cm^{-1}: 2957, 2829, 1464, 1249, 1058, 843, 760; ^{1}H NMR (500 MHz, $CDCl_3$) 7.69 (d, J = 2.0 Hz, 1H), 7.49 (d, J = 8.2 Hz, 1H), 7.27 (d, J = 8.2 Hz, 1H), 5.52 (s, 1H), 3.36 (s, 6H), 0.24 (s, 9H). ^{13}C NMR (126 MHz, $CDCl_3$) 136.90, 133.06, 132.81, 131.92, 123.02, 122.35, 103.81, 102.22, 95.55, 67.95, 53.60, 25.59, –0.15. HRMS (EI) exact mass calculated for [M$^+$] ($C_{14}H_{19}O_2SiBr$) requires *m*/*z* 326.0338, found *m*/*z* 326.0338

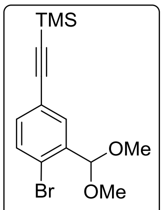

2i

2–cyclohexyltetrahydro–4H–cyclopenta[*d*][1,3,2]dioxaborole (1b)

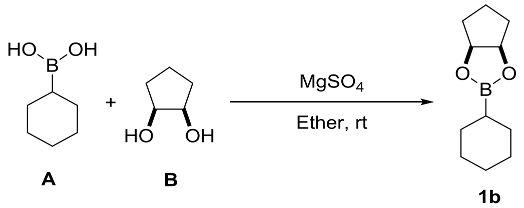

To a flame–dried 100 mL round bottomed flask containing cyclohexyl boronic acid **A** (1 g, 7.8 mmol, 1.0 equiv) in Et_2O (20 mL) at rt under N_2 was added *cis*–cyclopentyl glycol **B** (876 mg, 8.59 mmol, 1.1 equiv). The reaction mixture became homogenous in 5 min; then $MgSO_4$ (stored in oven) (3 g) was added and the mixture was stirred over–night. The reaction mixture was filtered through a plug of cotton and the filtrate was concentrated in vacuo. The crude mixture was then distilled with a Hickman distillation setup to afford the product **1b** (1.19 g, 80%). IR (film) ν_{max}/cm^{-1}: 2959, 2928, 1511, 1243, 1215, 1163, 1035, 829; ^{1}H NMR (500 MHz, $CDCl_3$) ^{1}H

NMR (500 MHz, $CDCl_3$) 4.79 (m, 2H), 1.94 – 1.82 (m, 2H), 1.72 – 1.47 (m, 9H), 1.40 – 1.21 (m, 5H), 1.09 – 0.95 (m, 1H). ^{13}C NMR (126 MHz, $CDCl_3$) 81.94, 34.69, 28.15, 27.13, 26.73, 21.40. ^{11}B NMR (96 MHz, $CDCl_3$) 34.02. HRMS (EI) exact mass calculated for $[M^+]$ $C_{11}H_{19}O_2B$ requires 194.1478 found: 194.1476.

B. General Procedure for the Preparation of Bneop Esters through Lithiation–Borylation

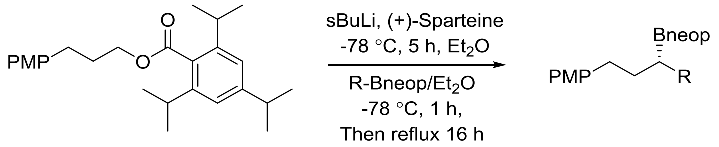

To a two–neck 100 mL round–bottomed flask containing the TIB ester (7.37 mmol, 1.0 equiv) and (+)–sparteine (2.20 mL, 9.58 mmol, 1.30 equiv) in Et_2O (60 mL) at –78 °C under N_2 was added s–butyllithium (1.3 M in hexanes, 6.80 mL, 8.84 mmol, 1.20 equiv) dropwise. The reaction mixture was stirred at –78 °C for 5 h, and a solution of the boronic ester (9.58 mmol, 1.30 eq) in Et_2O (5.0 mL) was added dropwise and stirred at –78 °C for 1 h and then warmed to reflux for 16 h. The reaction mixture was then quenched by the addition of H_2O/Et_2O (1:1, 50 mL) and the aqueous phase was extracted with Et_2O (2 x 30 mL). The organic layer was washed successively with HCl (1 M, 50 mL), $NaHCO_3$ (saturated, 50 mL), H_2O (50 mL) and brine (50 mL), dried over Na_2SO_4), filtered and concentrated. The crude product was then purified by a quick flash column chromatography (petroleum ether/EtOAc = 95/5) to afford product as a colourless oil. The enantiomeric ratio was determined by oxidative hydrolysis to the corresponding alcohol and subsequent HPLC analysis.

***(R)*–2–(4–(4–methoxyphenyl)butan–2–yl)–5,5–dimethyl–1,3,2–dioxaborinane (6a):**

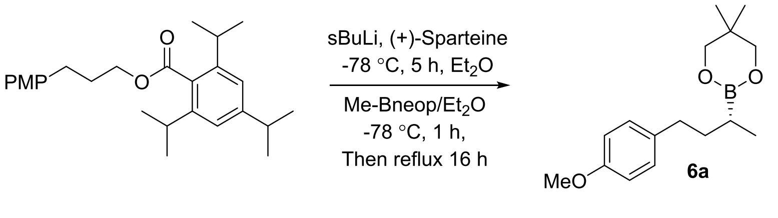

The TIB ester (2 g; 5.05 mmol, 1 equiv) was subjected to the General procedure B using Me–Bneop (840 mg, 6.56 mmol, 1.3 equiv). The crude product was then purified by distillation at 250 °C in 0.8 mm Hg to afford product (926 mg, 66%) as a colourless oil. The data matched well with the data previously reported in the literature[1] **$[\alpha]_D^{23}$** = –30.21 (*c* 0.96, $CHCl_3$); ^1H NMR (301 MHz, $CDCl_3$) 7.10 (d, *J* = 8.7 Hz, 2H), 6.80 (d, *J* = 8.7 Hz, 2H), 3.77 (s, 3H), 3.55 (s, 4H), 2.64 – 2.45 (m, 2H), 1.74 (ddt, *J* = 13.2, 9.6, 6.8 Hz, 1H), 1.51 (ddt, *J* = 13.2, 9.6, 6.8 Hz, 1H),

0.97 – 0.92 (m, 10H). The enantiomeric ratio was determined by oxidation to the corresponding alcohol. HPLC Conditions: Chiralpak IB column with guard, 3% iPrOH in hexane, flow rate: 0.7 mL/min, t_R 18.0 (minor) and t_R 21.2 min (major) er – 95.5:4.5.[2]

***(R)*–5,5–dimethyl–2–(1–phenylhept–6–en–3–yl)–1,3,2–dioxaborinane (6b):**

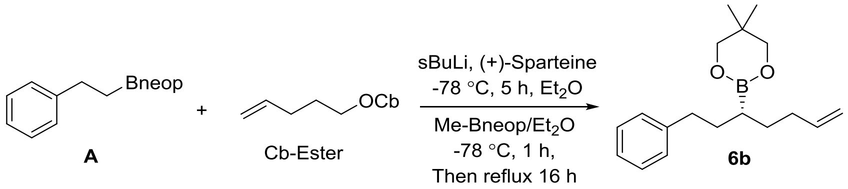

To a two–neck 100 mL round–bottomed flask containing the Cb ester (1.28 g, 6.0 mmol, 1.3 equiv) and (+)–sparteine (1.5 mL, 6.0 mmol, 1.30 equiv) in TBME (60 mL) at –78 °C under N_2 was added s–butyllithium (1.3 M in hexanes, 4.25 mL, 5.52 mmol, 1.20 equiv) dropwise. The reaction mixture was stirred at –78 °C for 5 h, and a solution of the boronic ester A (1 g, 4.6 mmol, 1.0 eq) in TBME (5.0 mL) was added dropwise and stirred at –78 °C for 1 h and then warmed to reflux for 16 h. The reaction mixture was then quenched by the addition of H_2O/Et_2O (1:1, 50 mL) and the aqueous phase was extracted with Et_2O (2 x 30 mL). The organic layer was washed successively with HCl (1 M, 50 mL), $NaHCO_3$ (saturated, 50 mL), H_2O (50 mL) and brine (50 mL), dried over Na_2SO_4), filtered and concentrated. The crude product was then purified by distillation (850 mg) followed a quick flash column chromatography (3 x 0.5″ silica column) (petroleum ether/EtOAc = 95/5) to afford product **6b** (600 mg, 45%) as a colourless oil. The enantiomeric ratio was determined by oxidative hydrolysis to the corresponding alcohol and subsequent HPLC analysis. $[\alpha]_D^{23}$ = –10.00 (*c* 0.8, $CHCl_3$); IR (film) ν_{max}/cm^{-1}: 2958, 2924, 1413, 1250, 1072, 907, 698; ^{1}H NMR (500 MHz, $CDCl_3$) 7.30 – 7.23 (m, 2H), 7.19 (d, *J* = 7.1 Hz, 2H), 7.15 (t, *J* = 7.3 Hz, 1H), 5.82 (ddt, *J* = 16.9, 10.2, 6.7 Hz, 1H), 5.04 – 4.88 (m, 2H), 3.56 (s, 4H), 2.61 (t, *J* = 8.1 Hz, 2H), 2.06 (q, *J* = 7.8 Hz, 2H), 1.75 (dq, *J* = 13.2, 8.3 Hz, 1H), 1.69 – 1.50 (m, 2H), 1.50 – 1.40 (m, 1H), 0.96 (s, 6H), 0.96 – 0.90 (m, 1H). ^{13}C NMR (126 MHz, $CDCl_3$) 143.23, 139.49, 128.44, 128.14, 125.43, 113.98, 71.83, 35.66, 33.47, 33.28, 31.54, 30.69, 21.97. ^{11}B NMR (96 MHz, $CDCl_3$) 29.68. HRMS (ESI) exact mass calculated for $[M+Na^+]$ $C_{18}H_{27}BNaO_2$ requires 309.2000 found: 309.1995. The enantiomeric ratio was determined by oxidation to the corresponding alcohol. HPLC Conditions: Chiralpak IB column with guard,

5% iPrOH in hexane, flow rate: 0.5 mL/min, t_R 14.0 (minor) and t_R 17.6 min (major) er – 90.5:9.5.[3]

***(S)*–2–(1–cyclopropyl–3–phenylpropyl)–5,5–dimethyl–1,3,2–dioxaborinane (6c)**

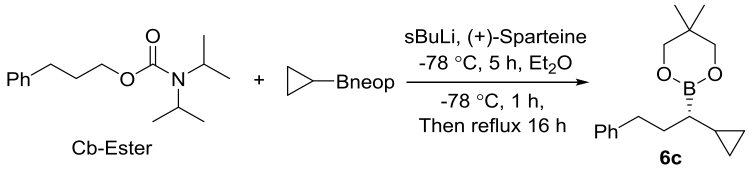

The Cb ester[4] (1 g, 3.80 mmol, 1.0 equiv) was subjected to the General Procedure B using cyclopropyl Bneop (0.76 g, 4.94 mmol, 1.3 equiv). The crude product was then purified by a quick flash column chromatography (3 x 0.5″ silica column) (petroleum ether/EtOAc = 95/5) to afford product **6c** (630 mg; 61%) as a colourless oil. $[\alpha]_D^{23}$ = –13.64 (*c* 0.88, $CHCl_3$); IR (film) ν_{max}/cm^{-1}: 2997, 2961, 2928, 1475, 1414, 1252, 698; ^{1}H NMR (500 MHz, $CDCl_3$) 7.29 – 7.24 (m, 2H), 7.23 – 7.17 (m, 2H), 7.17 – 7.12 (m, 1H), 3.56 (s, 4H), 2.72 – 2.58 (m, 2H), 1.89 – 1.71 (m, 2H), 0.96 (s, 6H), 0.69 (dtt, *J* = 9.9, 8.0, 5.0 Hz, 1H), 0.47 – 0.34 (m, 2H), 0.18 (td, *J* = 9.4, 6.0 Hz, 1H), 0.09 (dtd, *J* = 9.1, 4.9, 3.8 Hz, 1H), 0.05– –0.05 (m, 1H). ^{13}C NMR (126 MHz, $CDCl_3$) 143.31, 128.45, 128.10, 125.38, 71.87, 35.89, 33.67, 31.63, 21.84, 12.96, 5.33, 3.73. ^{11}B NMR (96 MHz, $CDCl_3$) 29.05. HRMS (ESI) exact mass calculated for [M+Na$^+$] $C_{17}H_{25}BNaO_2$ requires 295.1843 found 295.1848. The enantiomeric ratio was determined after oxidation to the corresponding alcohol. HPLC Conditions: Chiralpak IB column with guard, 3.0% iPrOH in hexane, flow rate: 0.7 mL/min, 20 °C; t_R 14.1 (minor) and t_R 18.2 min (major) er – 99:1.[5]

***(R)*–2–(7–azido–1–phenylheptan–3–yl)–5,5–dimethyl–1,3,2–dioxaborinane (6f)**

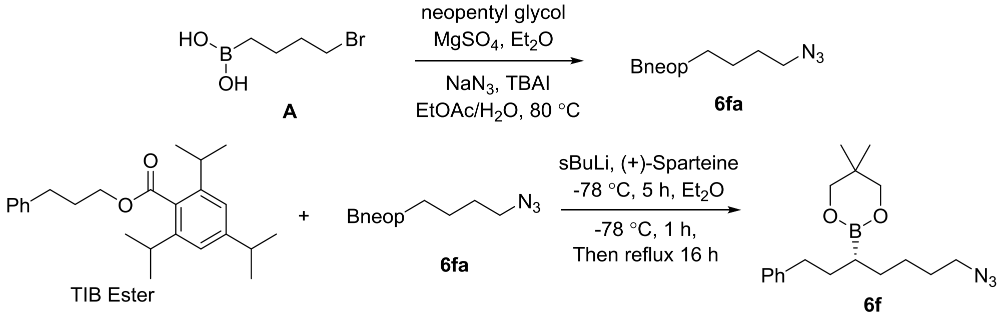

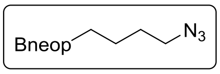

To a flame–dried 100 mL round bottomed flask containing bromobutyl boronic acid **A** (1 g, 5.5 mmol, 1.0 equiv) in Et_2O (15 mL) at rt under N_2 was

added neopentyl glycol (634 mg, 6.10 mmol, 1.1 equiv). The reaction mixture became homogenous in 5 min, then $MgSO_4$ (stored in oven) (2 g) was stirred over–night. The reaction mixture was filtered through a plug of cotton and the filtrate was concentrated in vacuo. The crude mixture was then dissolved in EtOAc (15 mL) and water (15 mL) was added. To the biphasic mixture, NaN_3 (3.62 g, 55.0 mmol, 10 equiv) and TBAI (1.03g, 2.78 mmol, 0.5 equiv) in sequence and the mixture was stirred at 80 °C over–night. The aqueous phase was extracted with EtOAc (2 x 30 mL). The combined organic layer was washed with brine (50 mL), dried over Na_2SO_4, filtered and concentrated. The crude product was then purified by a quick flash column chromatography (3 x 0.5″ silica column) (petroleum ether/EtOAc = 90/10) to afford product **6fa** (724 mg, 61%) as a colourless oil. **IR** (film) ν_{max}/cm^{-1}: 2962, 2932, 2873, 2090, 1476, 1248, 1173, 813; ^{1}H NMR (500 MHz, $CDCl_3$) 3.58 (s, 4H), 3.24 (t, *J* = 7.0 Hz, 2H), 1.65 – 1.56 (m, 2H), 1.45 (m, 2H), 0.95 (s, 6H), 0.74 (t, *J* = 7.8 Hz, 2H). ^{13}C NMR (126 MHz, $CDCl_3$) 71.98, 51.35, 31.60, 31.31, 21.82, 21.27. ^{11}B NMR (96 MHz, $CDCl_3$) 29.20. In EI or ESI heavy fragmentation was observed $[M–N_2–H]^+$ is the molecular ion peak with a mass of 181.2, 182.2, 183.2 [with boron's isotopic pattern]

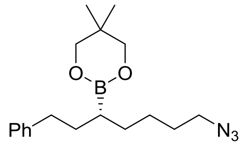

6f

The TIB ester (550 mg; 1.5 mmol, 1.3 equiv) was subjected to the General Procedure B using azidobutyl Bneop ester **6af**. The crude product was then purified by a quick flash column chromatography (3 x 0.5″ silica column) (petroleum ether/EtOAc = 85/15) to afford product **6f** (120 mg, 24%) as a colourless oil. $[\alpha]_D^{23}$ = +22.73 (*c* 0.88, $CHCl_3$); IR (film) ν_{max}/cm^{-1}: 2961, 2932, 2873, 2090, 1476, 1289, 1249, 1173, 813; ^{1}H NMR (500 MHz, $CDCl_3$) 7.29 – 7.24 (m, 2H), 7.21 – 7.17 (m, 2H), 7.17 – 7.12 (m, 1H), 3.55 (s, 4H), 2.62 – 2.46 (m, 2H), 1.69 (m, 1H), 1.51 – 1.42 (m, 2H), 1.29 – 1.23 (m, 1H), 0.95 (s, 6H). ^{13}C NMR (126 MHz, $CDCl_3$) 143.12, 128.43, 128.15, 125.46, 71.85, 51.41, 35.66, 33.28, 31.55, 30.81, 29.14, 26.31, 21.94. ^{11}B NMR (96 MHz, $CDCl_3$) 29.58. HRMS (ESI) exact mass calculated for $[M+Na^+]$ $C_{18}H_{28}BN_3NaO_2$ requires 352.2170 found 352.2176. The enantiomeric ratio was determined by oxidation to the corresponding alcohol. SFC conditions: Chiralpak IB column with guard, 5% MeOH, flow rate: 4 mL/min, 40 °C, 125 bar, t_R 4.12 min (major) and t_R 4.75 min; er – 99:1.[3]

***(S)*–6–phenyl–4–(4,4,5,5–tetramethyl–1,3,2–dioxaborolan–2–yl)hexanenitrile (9h)**

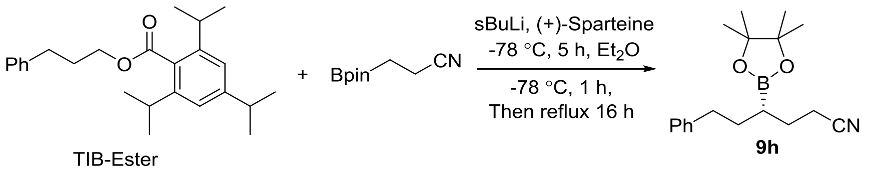

The TIB ester[2] (550 mg; 1.5 mmol, 1.0 equiv) was subjected to the General Procedure B using cyanoethyl Bpin ester (300 mg, 1.66 mmol, 1.1 equiv). The crude product was then purified by a flash column chromatography (petroleum ether/EtOAc = 95/5) to afford product **9h** (186 mg, 41%) as a colourless oil. $[\alpha]_D^{23}$ = -11.12 (*c* 1.34, $CHCl_3$); ^{1}H NMR (500 MHz, $CDCl_3$) 7.28 (d, *J* = 7.8 Hz, 2H), 7.22 – 7.12 (m, 3H), 2.62 (m, 2H), 2.47 – 2.29 (m, 2H), 1.91 – 1.60 (m, 4H), 1.26 (s, 12H), 1.16 (m, 1H). ^{13}C NMR (126 MHz, $CDCl_3$) 142.28, 128.33, 128.32, 125.79, 120.01, 83.45, 35.04, 32.65, 26.79, 24.87, 24.78, 16.45; ^{11}B NMR (96 MHz, $CDCl_3$) 34.22. HRMS (ESI) exact mass calculated for [M+H$^+$] $C_{18}H_{26}BNNaO_2$ requires 322.1952 found 322.1967. The enantiomeric ratio was determined by oxidation to the corresponding alcohol. HPLC conditions: Chiralpak IB column with guard, 5.0% iPrOH in hexane, flow rate: 0.7 mL/min, t_R 41.9 min (minor) and t_R 45.8 min; er – 97:3.[5]

tert-butyl(((2S,3R)-3-(5,5-dimethyl-1,3,2-dioxaborinan-2-yl)-2,4-dimethylpentyl)oxy) dimethylsilane (7d)

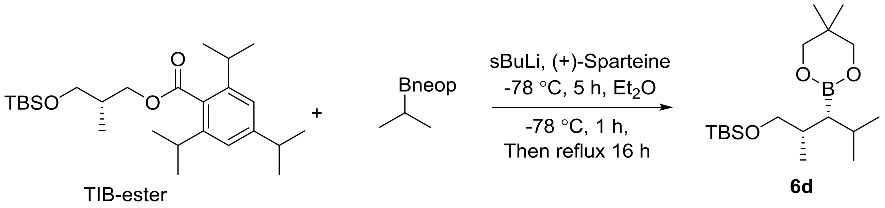

The TIB ester (300 mg; 1.5 mmol, 1.3 equiv) was subjected to the General Procedure B using isopropyl Bneop ester. The crude product was then purified by a quick flash column chromatography (3 x 0.5" silica column) (petroleum ether/EtOAc = 85/15) to afford product **6d** (120 mg, 51%) as a colourless oil; dr 92:8. $[\alpha]_D^{23}$ = -20.34 (*c* 1.05, $CHCl_3$); ^{1}H NMR (500 MHz, Chloroform-*d*) δ 3.59 (s, 4H), 3.35 (dd, *J* = 9.7, 8.0 Hz, 1H), 1.88 – 1.73 (m, 2H), 1.27 – 1.23 (m, 2H), 0.98 (s, 6H), 0.93 – 0.88 (m, 18H), 0.65 (dd, *J* = 8.3, 6.6 Hz, 1H), 0.04 (s, 6H). ^{13}C NMR (126 MHz, cdcl$_3$) δ 71.55, 68.49, 35.19, 31.38, 26.60, 26.00, 22.53, 22.31, 22.29, 18.40,

15.54, -5.26, -5.31. ^{11}B NMR (96 MHz, $CDCl_3$) 30.64. HRMS (ESI) exact mass calculated for [M+H$^+$] $C_{18}H_{39}BNaO_3Si$ requires 365.2656 found 365.2657.

5,5–dimethyl–2–(3–methyl–1–phenylpentan–3–yl)–1,3,2–dioxaborinane (6h)

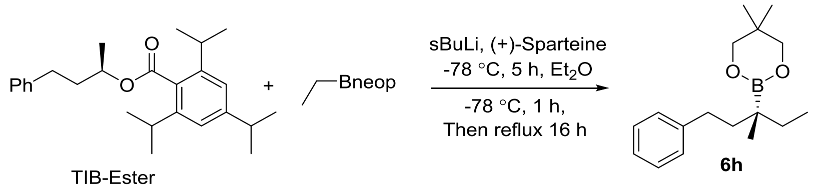

To a two–neck 100 mL round bottomed flask containing the enantioenriched TIB ester (520 mg, 1.37 mmol, 1.0 equiv) and TMEDA (1.23 mL, 8.22 mmol, 6.0 equiv) in CPME (7 mL) at –60 °C under N_2 was added s–butyllithium (1.3 M in hexanes, 1.68 mL, 2.19 mmol, 1.20 equiv) dropwise. The reaction mixture was stirred at –60 °C for 1 h, and a solution of ethyl Bneop (390 mg, 2.74 mmol, 2.0 equiv) in CPME (2.0 mL) was added dropwise. The mixture was then stirred at –60 °C for 1 h and then warmed to reflux for 16 h. The reaction mixture was then quenched by the addition of H_2O/Et_2O (1:1, 50 mL) and the aqueous phase was extracted with Et_2O (2 x 30 mL). The organic layer was washed successively with HCl (1 M, 50 mL), $NaHCO_3$ (saturated, 50 mL), H_2O (50 mL) and brine (50 mL), dried over Na_2SO_4), filtered and concentrated. The crude product was then purified by a quick flash column chromatography (3 x 0.5″ silica column) (petroleum ether/EtOAc = 95/5) to afford product **6h** (293 mg, 78%) as a colourless oil. The enantiomeric ratio was determined by oxidative hydrolysis to the corresponding alcohol and subsequent HPLC analysis. $[\alpha]_D^{23}$ = –14.58 (*c* 0.48, $CHCl_3$); R_f (95/5 petroleum ether/EtOAc): 0.72; IR (film) ν_{max}/cm^{-1}: 2972, 2929, 1512, 1373, 1246, 1065, 861; ^{1}H NMR (500 MHz, $CDCl_3$) 7.29 – 7.24 (m, 2H), 7.21 – 7.17 (m, 2H), 7.17 – 7.12 (m, 1H), 3.55 (s, 4H), 2.62 – 2.46 (m, 2H), 1.69 (ddd, *J* = 13.2, 11.9, 5.3 Hz, 1H), 1.51 – 1.42 (m, 2H), 1.29 – 1.23 (m, 1H), 0.95 (s, 6H), 0.93 (s, 3H), 0.86 (t, *J* = 7.5 Hz, 3H). ^{13}C NMR (126 MHz, $CDCl_3$) 143.91, 128.40, 128.15, 125.31, 71.83, 41.23, 32.45, 31.50, 31.41, 21.99, 20.82, 10.11. ^{11}B NMR (96 MHz, $CDCl_3$) 30.12. HRMS: In EI or ESI heavy fragmentation was observed with molecular ion peak with a mass of 161.03. The enantiomeric ratio was determined by oxidation to the corresponding alcohol. HPLC conditions: Chiralpak IB column with guard, 2% iPrOH/hexane, flow rate: 0.75 mL/min, t_R 23.6 min (minor) and t_R 25.1 min (major); er – 99:1.[6]

2–((3r,5r,7r)–adamantan–1–yl)–5,5–dimethyl–1,3,2–dioxaborinane (6i): To a flame–dried 100 mL round bottomed flask containing adamanty boronic acid (1 g, 5.5 mmol, 1.0 equiv) in Et_2O (20 mL) at rt under N_2 was added *cis*–cyclopentyl glycol (635 mg, 6.11 mmol, 1.1 equiv). The reaction mixture became homogenous in 5 min, then $MgSO_4$ (stored in oven) (2 g) was stirred over–night. The reaction mixture was filtered through a plug of cotton and the filtrate was concentrated in vacuo. The crude mixture was then distilled with a Hickman distillation setup to afford the product (1.19 g, 80%). ^{1}H NMR (400 MHz, $CDCl_3$) 3.55 (s, 4H), 1.83 (bs, 3H), 1.78 – 1.64 (m, 12H), 0.91 (s, 6H). ^{13}C NMR (101 MHz, $CDCl_3$) 71.96, 38.27, 37.66, 31.60, 27.82, 21.68. ^{11}B NMR (96 MHz, $CDCl_3$) 29.52. HRMS: In EI or ESI heavy fragmentation was observed with molecular ion peak $[M–Me]^+$ with a mass of 232.17, 233.17, 234.17.

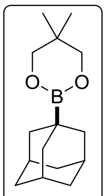

6i

2–((1R,2R,5R)–2–isopropyl–5–methylcyclohexyl)–5,5–dimethyl–1,3,2–dioxaborinane (6e):

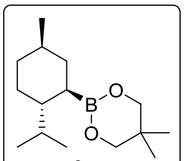

6e

To a two–neck 100 mL round–bottomed flask containing copper (I) chloride (59 mg, 0.6 mmol, 0.1 equiv) and bis(neopentylglycolato)diboron (152.4 mg, 7.0 mmol, 1.2 equiv), Xantphos (347 mg, 0.6 mmol; 0.1 equiv) in THF (25 mL) under N_2 was added KO^tBu/THF (1.0 M, 5.75 mL, 5.75 mmol, 1 equiv). Then menthyl chloride (1 g, 5.75 mmol) was added dropwise and the mixture was stirred for 24 h. Then the reaction mixture was diluted with Et_2O and the mixture was passed through a pad of Celite and washed with HCl (1 M). The organic layer was dried over Na_2SO_4 and concentrated in vacuo. The crude mixture was further purified by quick flash column chromatography (petroleum ether/EtOAc = 97/3) to afford product **6e** (406 mg, 28%) as a colourless oil. $[\alpha]_D^{23}$ = –90.38 (*c* 0.52, $CHCl_3$); R_f (95/5 petroleum ether/EtOAc): 0.72; IR (film) ν_{max}/cm^{-1}: 2927, 2883, 1511, 1253, 1065, 861; ^{1}H NMR (500 MHz, $CDCl_3$) 3.57 (s, 4H), 1.73 – 1.54 (m, 4H), 1.29 – 1.18 (m, 2H), 1.00 – 0.73 (m, 19H). ^{13}C NMR (126 MHz, $CDCl_3$) 71.86, 43.81, 37.88, 35.48, 33.49, 32.14, 31.62, 26.27, 22.77, 21.87, 21.48, 16.85. ^{11}B NMR (96 MHz, $CDCl_3$) 29.32. HRMS: In EI or ESI heavy fragmentation was observed with molecular ion peak with a mass of 208.17, 209.17, 210.17.

tert–butyl 4–(5,5–dimethyl–1,3,2–dioxaborinan–2–yl)piperidine–1–carboxylate (6g):

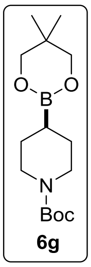

To a two–neck 100 mL round–bottomed flask containing the copper (I) chloride (75 mg, 0.76 mmol, 0.2 equiv) and bis(neopentylglycolato)diboron (1.04 g, 4.6 mmol, 1.2 equiv), Xantphos (440 mg, 0.76 mmol; 0.2 equiv) in THF (20 mL) under N_2 was added KO^{t}Bu /THF (1.0 M, 3.8 mL, 3.8 mmol, 1 equiv). Then alkyl bromide (1 g, 3.8 mmol) was added dropwise and the mixture was stirred for 24 h. Then the reaction mixture was diluted with Et_2O and the mixture was passed through a pad of Celite and washed with HCl (1 M). The organic layer was dried over Na_2SO_4 and concentrated in vacuo. The crude mixture was further purified by quick flash column chromatography (petroleum ether/EtOAc = 90/10) to afford product **6g** (508 mg, 45%) as a colourless oil. R_f (95/5 petroleum ether/EtOAc): 0.72; IR (film) ν_{max}/cm^{-1}: 2930, 1692, 1228, 1166, 863, 841; ^{1}H NMR (500 MHz, $CDCl_3$) 3.85 (bs, 2H), 3.57 (s, 4H), 2.81 (m, 2H), 1.67 – 1.58 (m, 2H), 1.44 (s, 9H), 1.49 – 1.39 (m, 2H), 1.00–0.95 (m, 1H), 0.93 (s, 6H). ^{13}C NMR (126 MHz, $CDCl_3$) 154.92, 78.89, 71.98, 44.64, 31.61, 28.49, 27.13, 21.74. ^{11}B NMR (96 MHz, $CDCl_3$) 29.53. HRMS (ESI) exact mass calculated for [M+Na$^+$] $C_{15}H_{28}BNNaO_4$ requires 320.2006 found 320.1992.

***(R)*–1–(3–(4,4,5,5–tetramethyl–1,3,2–dioxaborolan–2–yl)–3,4–dihydroquinolin–1(2H)–yl)ethan–1–one:**

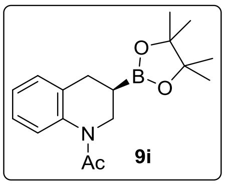

The title compound was obtained in 71% yield following the literature procedure.[7] The racemic product was obtained by using Xantphos in the place of QuinoxP* ligand. The crude mixture was further purified by flash column chromatography (Toluene/EtOAc = 100/0 to 80/20). R_f (80/20 Toluene/EtOAc): 0.4; $[\alpha]_D^{23}$ = –9.38 (*c* 0.88, $CHCl_3$) IR (film) ν_{max}/cm^{-1}: 2931, 2918, 1703, 1228, 1166, 863, 841; ^{1}H NMR (500 MHz, $CDCl_3$) 7.23 – 7.10 (m, 3H), 7.06 (t, *J* = 7.4 Hz, 1H), 3.98 (s, 1H), 3.67 (dd, *J* = 12.7, 9.9 Hz, 1H), 3.03 – 2.52 (m, 2H), 2.24 (s, 3H), 1.56 (s, 1H), 1.20 (s, 6H), 1.20 (s, 6H). ^{13}C NMR (126 MHz, $CDCl_3$) 170.10, 139.03, 128.19, 125.89, 124.71, 124.58, 83.62, 44.87, 28.72, 24.72, 23.45, 20.67. ^{11}B NMR (96 MHz, $CDCl_3$) 29.51. HRMS (ESI) exact mass calculated for [M+Na$^+$] $C_{17}H_{24}BNNaO_3$ requires 324.1744 found 324.1746. The enantiomeric ratio was determined by oxidation to the corresponding alcohol. The ratio was found to be 99.5:0.5.

C. General Procedure: C(sp^3)–C(sp^2) coupling of boronic esters with *p*–bromophenylacetylenes using NBS

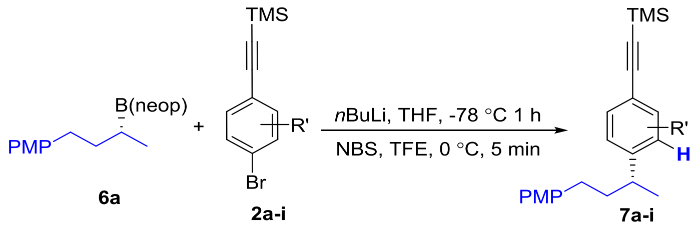

A solution of *p*–bromophenylacetylene **2a-i** (0.2 mmol, 1.2 equiv) in THF (0.7 mL, 0.3 M) was cooled to –78 °C and treated with *n*–BuLi (1.2 equiv, 1.6 M in hexanes) and the mixture was stirred at this temperature for 1 h. The boronic ester **6a** (0.18 mmol, 1.0 eq.) was added dropwise as a solution in THF (0.6 mL, 0.3 M). The mixture was stirred at –78 °C for 1 h at which point ^{11}B NMR analysis showed complete formation of the 'ate' complex [^{11}B NMR (96 MHz, THF) δ_B ~ 8 ppm]. The reaction mixture was warmed to 0 °C with an ice bath and TFE (0.6 mL) was added. Then, a solution of NBS (0.27 mmol; 1.5 equiv) in TFE (0.3 M) was added dropwise. After 5 min at 0 °C, $Na_2S_2O_3$ (sat. aq.) was added and the reaction mixture was allowed to warm to room temperature. The reaction mixture was diluted with DCM and water. The layers were separated and the aqueous layer was extracted with DCM. The combined organic layers were dried (Na_2SO_4), filtered and concentrated under vacuum. The crude material was adsorbed on silica and purified by flash column chromatography on silica gel eluting with *n*–hexane/Et_2O.

((4–cyclohexylphenyl)ethynyl)trimethylsilane 4a: The starting CyBneop **1c** (35 mg, 0.18 mmol) was subjected to the conditions in General Procedure C. The crude product was purified by column chromatography (pentane/Et_2O – 98/2) to afford the title product **4a** (36 mg, 78%) as oil. (R_f (95/5 pentane/Et_2O): 0.7; IR (film) ν_{max}/cm^{-1}: 2987, 2970, 1511, 1106, 843; ^{1}H NMR (500 MHz, $CDCl_3$) 7.38 (d, *J* = 8.1 Hz, 2H), 7.13 (d, *J* = 8.1 Hz, 2H), 2.54 – 2.40 (m, 1H), 1.84 (m, 4H), 1.74 (m, 1H), 1.38 (m, 4H), 1.30 – 1.17 (m, 1H), 0.23 (s, 9H). ^{13}C NMR (126 MHz, $CDCl_3$) 148.69, 131.89, 126.68, 120.37, 105.40, 93.16, 44.49, 34.20, 26.77, 26.07, 0.02. HRMS (EI) exact mass calculated for [M^+] $C_{17}H_{24}Si$ requires 256.1647 Found 256.1645.

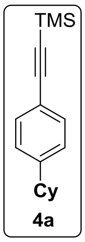

***(R)*–((4–(4–(4–Methoxyphenyl)butan–2–yl)phenyl)ethynyl)trimethylsilane (7a):** The starting boronic ester **6a** (52 mg, 0.18 mmol) was subjected to the conditions in General

Procedure C. The crude product was purified by column chromatography (petroleum ether/EtOAc – 95/5) to afford the title product **7a** (55 mg, 92%) as a gummy oil. $[\alpha]_D^{23}$ = +19.38 (c 0.31, $CHCl_3$); R_f (95/5 petroleum ether/EtOAc): 0.6; IR (film) ν_{max}/cm^{-1}: 2982, 2967, 1512, 1106, 843; ^{1}H NMR (500 MHz, $CDCl_3$) 7.41 (d, J = 8.2 Hz, 2H), 7.12 (d, J = 8.2 Hz, 2H), 7.01 (d, J = 8.8 Hz, 2H), 6.80 (d, J = 8.8 Hz, 2H), 3.78 (s, 3H), 2.69 (qt, J = 7.1 Hz, 1H), 2.41 (dt, J = 9.0, 4.9 Hz, 2H), 1.85 (q, J = 7.7 Hz, 2H), 1.24 (d, J = 7.0 Hz, 3H), 0.25 (s, 9H). ^{13}C NMR (126 MHz) 157.65, 148.00, 134.33, 132.03, 129.17, 126.99, 120.56, 113.69, 105.30, 93.35, 55.23, 39.97, 39.31, 32.82, 22.26, 0.02. HRMS (ESI) exact mass calcd. for [M+Na$^+$] $C_{22}H_{28}NaOSi$ requires 359.1802, found: 359.1820. SFC Conditions: Chiracel IA Column, 5% [10% IPA:Hexane]/$CO_{2(scf)}$, flow rate – 4 mL/min, Pressure:125 bar, Injection vol. – 10 µL, t_R (minor) = 7.7 min, t_R (major) = 8.4 min. er 95.7:4.3

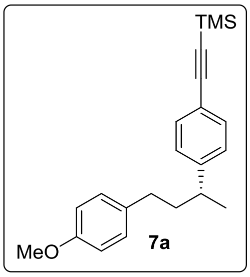

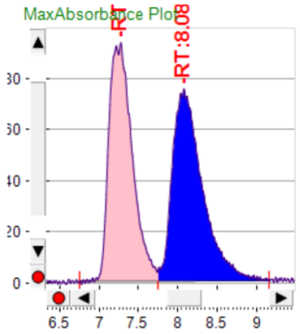

Peak #	Area %	Area	Ret. Time
1	49.267	1926.3392	7.27 min
2	50.733	1983.6559	8.08 min

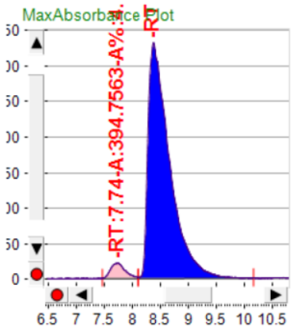

Peak #	Area %	Area	Ret. Time
1	4.2731	394.7563	7.74 min
2	95.7269	8843.331	8.38 min

(*R*)–((4–(4–(4–Methoxyphenyl)butan–2–yl)–3–methylphenyl)ethynyl)trimethyl silane (7b):

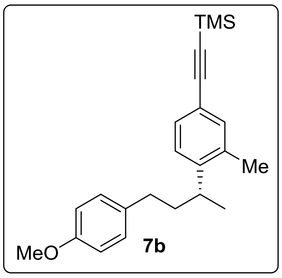

The starting boronic ester **6a** (52 mg, 0.18 mmol) was subjected to the conditions in General Procedure C. The crude product was purified by column chromatography (petroleum ether/EtOAc – 95/5) to afford the title product **7b** (53 mg, 85%). $[\alpha]_D^{23}$ = +29.44 (c *0.53*, $CHCl_3$); R_f (95/5 petroleum ether/EtOAc): 0.7; IR (film) ν_{max}/cm^{-1}: 2962, 1512, 1247, 1066, 1042, 842; ^{1}H NMR (500 MHz, $CDCl_3$) 7.30 (dd, J = 8.0, 1.8 Hz, 1H), 7.27 (dt, J = 1.8, 0.6 Hz, 1H), 7.15 (d, J = 8.0 Hz, 1H), 7.00 (d, J = 8.4 Hz, 2H), 6.80 (d, J = 8.4 Hz, 2H), 3.78 (s, 3H), 2.94 (qt, J = 7.0 Hz, 1H), 2.52–2.35 (m, 2H), 2.19 (s, 3H), 1.95–1.76 (m, 2H), 1.19 (d, J = 6.9 Hz, 3H), 0.25 (s, 9H). ^{13}C NMR (126 MHz, $CDCl_3$) 157.68, 146.27, 135.54, 134.30, 133.69, 129.83, 129.16, 125.21, 119.99, 113.70, 105.48, 93.01, 55.24, 39.27, 33.77, 32.78, 21.61, 19.24, 0.04. HRMS

(ESI) exact mass calculated for [M+Na$^+$] $C_{23}H_{30}NaOSi$ requires 373.1958, found 373.1960. HPLC Conditions: Chiracel IA Column wih Guard, Hexane/IPA – 99.9/0.1, flow rate – 1 mL/min, Injection vol. – 5 µL, t_R (minor) = 8.65 min, t_R (major) = 9.2 min. er 96:4

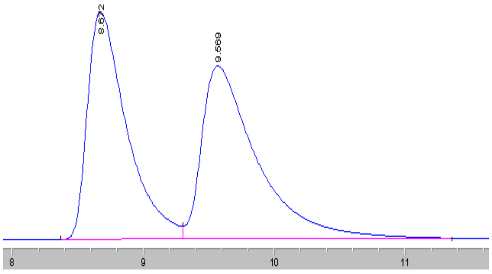

Peak #	RetTime [min]	Type	Width [min]	Area [mAU*s]	Height [mAU]	Area %
1	8.672	PV	0.3124	8338.00098	398.06824	48.2360
2	9.569	VB	0.4294	8947.84180	302.21576	51.7640

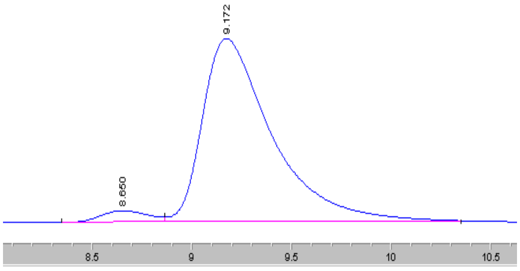

Peak #	RetTime [min]	Type	Width [min]	Area [mAU*s]	Height [mAU]	Area %
1	8.650	PV	0.2626	133.53928	8.08929	4.0645
2	9.172	VB	0.3607	3151.98096	129.15680	95.9355

***(R)*–((4–(4–(4–Methoxyphenyl)butan–2–yl)–2–methylphenyl)ethynyl)trimethylsilane (7c):**

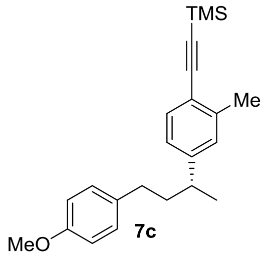

7c

The starting boronic ester **6a** (52 mg, 0.18 mmol) was subjected to the conditions in General Procedure C. The crude product was purified by column chromatography (petroleum ether/EtOAc – 95/5) to afford the title product **7c** (54 mg, 86%). $[\alpha]_D^{23}$ = –36.67 (*c* 1.2, $CHCl_3$); R_f (95/5 petroleum ether/EtOAc): 0.7; IR (film) ν_{max}/cm^{-1}: 2958, 2925, 1511, 1246, 858, 840; ^{1}H NMR (500 MHz, $CDCl_3$) 7.37 (d, *J* = 7.9 Hz, 1H), 7.06–6.99 (m, 3H), 6.95 (t, *J* = 1.9, 0.8 Hz, 1H), 6.80 (d, *J* = 8.6 Hz, 2H), 3.78 (s, 3H), 2.65 (qt, *J* = 7.0 Hz, 1H), 2.48–2.35 (m, 5H), 1.84 (tdd, *J* = 8.4, 7.1, 1.8 Hz, 2H), 1.23 (d, *J* = 6.9 Hz, 3H), 0.25 (s, 9H). ^{13}C NMR (126 MHz, $CDCl_3$) 157.63, 148.00, 140.60, 134.44, 132.13, 129.18, 128.20, 124.22, 120.41, 113.67, 104.28, 97.38, 55.23, 39.96, 39.32, 32.89, 22.29, 20.71, 0.11. HRMS (ESI) exact mass calculated for [M+Na$^+$] $C_{23}H_{30}NaOSi$ requires 373.1958, found 373.1957. HPLC Conditions: Chiracel IB Column, Hexane/IPA – 99.9/0.1, flow rate – 0.5 mL/min, Injection vol. – 5 µL, t_R (minor) = 34.7 min, t_R (major) = 37.2 min. er 95.2:4.8.

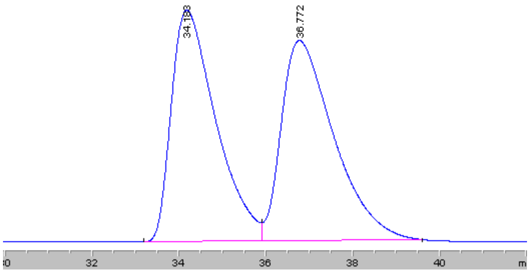

Peak #	RetTime [min]	Type	Width [min]	Area [mAU*s]	Height [mAU]	Area %
1	34.183	BV	1.0533	1.58655e4	222.67159	49.3264
2	36.772	VB	1.1940	1.62989e4	192.85364	50.6736

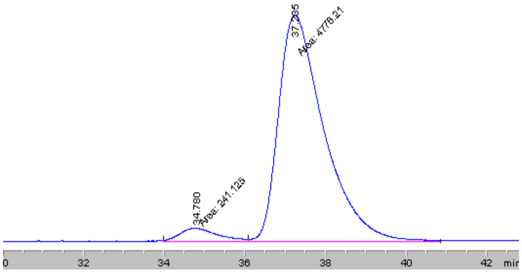

Peak #	RetTime [min]	Type	Width [min]	Area [mAU*s]	Height [mAU]	Area %
1	34.780	MF	1.1187	241.12503	3.59235	4.8058
2	37.235	FM	1.3069	4776.20898	60.91191	95.1942

(*R*)–((4–(4–(4–methoxyphenyl)butan–2–yl)naphthalen–1–yl)ethynyl)trimethylsilane (7d):

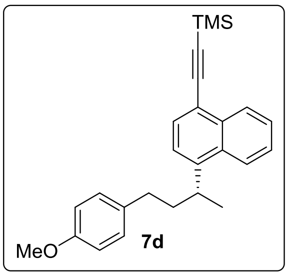

The starting boronic ester **6a** (52 mg, 0.18 mmol) was subjected to the conditions in General Procedure C. The crude product was purified by column chromatography (petroleum ether/EtOAc – 95/5) to afford the title product **7d** (62 mg, 89%). $[\alpha]_D^{23}$ = +9.26 (*c* 1.08, $CHCl_3$); R_f (95/5 petroleum ether/EtOAc): 0.7; IR (film) ν_{max}/cm^{-1}: 2958, 2931, 1519, 1245, 1037, 841, 760; ^{1}H NMR (400 MHz, $CDCl_3$) 8.42 (dd, *J* = 8.3, 1.2 Hz, 1H), 7.99 (d, *J* = 8.5 Hz, 1H), 7.71 (d, *J* = 7.6 Hz, 1H), 7.61–7.54 (m, 1H), 7.50 (td, *J* = 7.6, 6.8, 1.3 Hz, 1H), 7.37 (d, *J* = 7.6 Hz, 1H), 7.03 (d, *J* = 8.6 Hz, 2H), 6.81 (d, *J* = 8.6 Hz, 2H), 3.79 (s, 3H), 3.61 (sx, *J* = 6.9 Hz, 1H), 2.65–2.48 (m, 2H), 2.04 (m, 2H), 1.40 (d, *J* = 6.9 Hz, 3H), 0.34 (s, 9H). ^{13}C NMR (126 MHz, $CDCl_3$) 157.74, 144.98, 134.28, 133.79, 131.38, 130.76, 129.29, 127.14, 126.23, 126.14, 123.45, 121.95, 118.81, 113.74, 103.53, 98.83, 55.27, 39.65, 33.07, 32.91, 21.69, 0.17. HRMS (ESI) exact mass calculated for [M+H$^+$] $C_{26}H_{31}OSi$ requires 387.2139, Found 387.2137. HPLC Conditions: Chiracel IB with Guard Column, Hexane/IPA – 99.9/0.1, flow rate –0.5 mL/min, Injection vol. – 5 µL, t_R (major) = 26.72 min, t_R (minor) = 30.56 min. er 95.8:4.2.

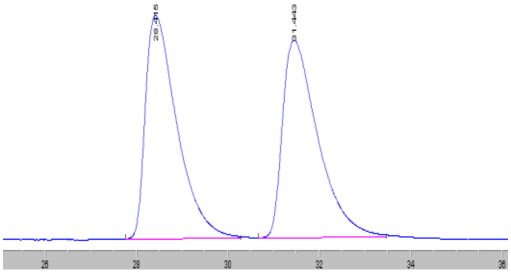

Peak #	RetTime [min]	Type	Width [min]	Area [mAU*s]	Height [mAU]	Area %
1	28.415	BB	0.7303	7644.46777	158.60571	50.2701
2	31.443	BB	0.8014	7562.31104	140.17732	49.7299

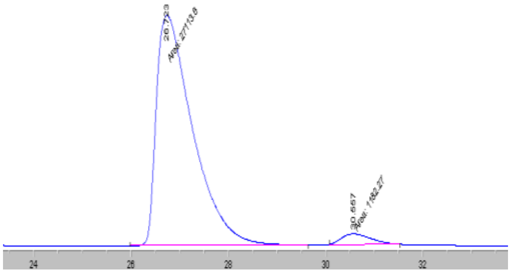

Peak #	RetTime [min]	Type	Width [min]	Area [mAU*s]	Height [mAU]	Area %
1	26.723	MM	0.8695	2.71136e4	519.71606	95.8218
2	30.557	MM	0.7483	1182.26770	26.33124	4.1782

(*R*)–((3–methoxy–4–(4–(4–methoxyphenyl)butan–2–yl)phenyl)ethynyl)trimethylsilane

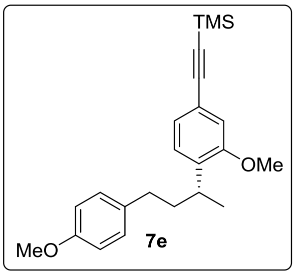

(7e): The starting boronic ester **6a** (52 mg, 0.18 mmol) was subjected to the conditions in General Procedure C. The crude product was purified by column chromatography (petroleum ether/EtOAc – 95/5) to afford the title product **7e** (54 mg, 82%). $[\alpha]_D^{23}$ = +30.77 (*c* 1.04, $CHCl_3$); R_f (95/5 petroleum ether/EtOAc): 0.6; IR (film) ν_{max}/cm^{-1}: 2958, 2927, 1511, 1247, 1040, 854, 842; ^{1}H NMR (500 MHz, $CDCl_3$) 7.11 (d, *J* = 7.8 Hz, 1H), 7.08 – 7.05 (m, 1H), 7.03 (d, *J* = 8.6 Hz, 2H), 6.94 (d, *J* = 1.5 Hz, 1H), 6.79 (d, *J* = 8.6 Hz, 2H), 3.80 (s, 3H), 3.78 (s, 3H), 3.19 (qt, *J* = 6.8, 0.8 Hz, 1H), 2.44 (m, 2H), 1.89 (dddd, *J* = 13.5, 10.2, 7.9, 5.7

Hz, 1H), 1.79 (dddd, J = 13.5, 10.2, 7.9, 5.7 Hz, 1H), 1.20 (d, J = 6.8 Hz, 3H), 0.25 (s, 9H). ^{13}C NMR (126 MHz, $CDCl_3$) 157.57, 156.63, 136.77, 134.78, 129.17, 126.73, 124.64, 121.04, 113.69, 113.60, 105.43, 93.08, 55.38, 55.24, 38.92, 32.95, 31.74, 20.83, 0.03. HRMS (ESI) exact mass calculated for [M+H$^+$] $C_{23}H_{30}NaO_2Si$ requires 389.1907, Found 389.1913. SFC Conditions: Chiracel IB Column, 5% [10% IPA:Hexane]/$CO_{2(scf)}$, flow rate – 4 mL/min, Pressure:125 bar, Injection vol. – 10 μL, t_R (major) = 9.8 min, t_R (minor) = 10.8 min. er 96:4.

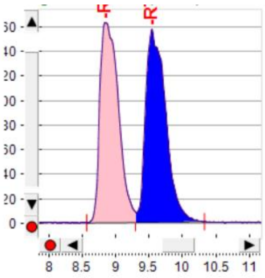

Peak #	Area %	Area	Ret. Time
1	49.0409	3152.1492	8.85 min
2	50.9591	3275.4384	9.54 min

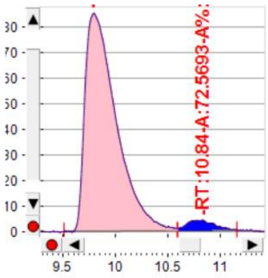

Peak #	Area %	Area	Ret. Time
1	96.0596	1769.1135	9.79 min
2	3.9404	72.5693	10.84 min

***(R)*–((2–methoxy–4–(4–(4–methoxyphenyl)butan–2–yl)phenyl)ethynyl)trimethylsilane (7f):**

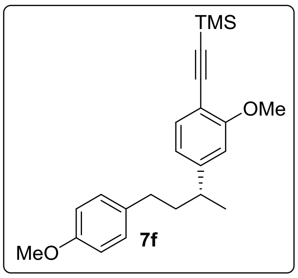

The starting boronic ester **6a** (52 mg, 0.18 mmol) was subjected to the conditions in General Procedure C. The crude product was purified by column chromatography (petroleum ether/EtOAc – 95/5) to afford the title product **7f** (59 mg, 90%). $[\alpha]_D^{23}$ = –26.30 (c 1.18, $CHCl_3$); R_f (95/5 petroleum ether/EtOAc): 0.5; IR (film) ν_{max}/cm^{-1}: 2958, 2931, 1510, 1244, 857, 839, 758; ^{1}H NMR (500 MHz, $CDCl_3$) 7.37 (d, J = 7.8 Hz, 1H), 7.02 (d, J = 8.7 Hz, 2H), 6.81 (d, J = 8.7 Hz, 2H), 6.73 (dd, J = 7.8, 1.5 Hz, 1H), 6.66 (d, J = 1.4 Hz, 1H), 3.88 (s, 3H), 3.78 (s, 3H), 2.67 (sx, J = 7.1 Hz, 1H), 2.43 (m, 2H), 1.90 – 1.81 (m, 2H), 1.25 (d, J = 6.9 Hz, 3H), 0.26 (s, 9H). ^{13}C NMR (126 MHz, $CDCl_3$) 157.57, 156.63, 136.77, 134.78, 129.17, 126.73, 124.64, 121.04, 113.69, 113.60, 105.43, 93.08, 55.38, 55.24, 38.92, 32.95, 31.74, 20.83, 0.03. HRMS (ESI) exact mass calculated for [M+Na$^+$] $C_{23}H_{30}NaO_2Si$ requires 389.1907 Found 389.1893. SFC Conditions: Chiracel IB Column, 5% [10% IPA:Hexane]/$CO_{2(scf)}$, flow rate – 4 mL/min, Pressure:125 bar, Injection vol. – 10 μL, t_R (minor) = 9.5 min, t_R (major) = 9.9 min. er 95.7:4.3.

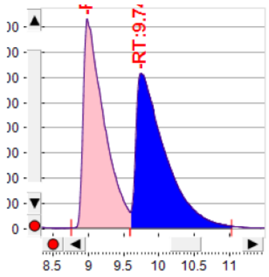

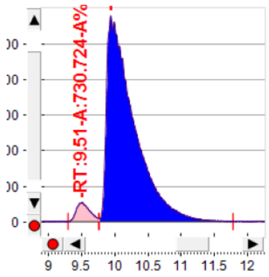

Peak #	Area %	Area	Ret. Time
1	48.8765	16865.5665	8.98 min
2	51.1235	17640.8947	9.74 min

Peak #	Area %	Area	Ret. Time
1	4.2873	730.724	9.51 min
2	95.7127	16313.1298	9.94 min

(*R*)–((3–fluoro–4–(4–(4–methoxyphenyl)butan–2–yl)phenyl)ethynyl)trimethylsilane (7g):

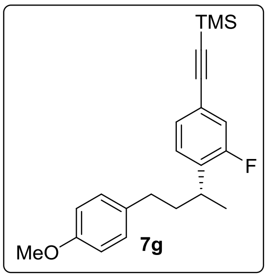

7g

The starting boronic ester **9a** (52 mg, 0.18 mmol) was subjected to the conditions in General Procedure C. The crude product was purified by column chromatography (petroleum ether/EtOAc – 95/5) to afford the title product **7g** (45 mg, 71%). $[\alpha]_D^{23}$ = –30.00 (*c* 1.03, $CHCl_3$); R_f (95/5 petroleum ether/EtOAc): 0.72; IR (film) ν_{max}/cm^{-1}: 2958, 2928, 1511, 1246, 859, 840, 759; ^{1}H NMR (500 MHz, $CDCl_3$) 7.37 (t, *J* = 7.7 Hz, 1H), 7.01 (d, *J* = 8.7 Hz, 2H), 6.93 – 6.86 (m, 2H), 6.81 (d, *J* = 8.6 Hz, 2H), 3.78 (s, 3H), 2.69 (sx, *J* = 7.0 Hz, 1H), 2.48 – 2.36 (m, 2H), 1.89 – 1.79 (m, 2H), 1.23 (d, *J* = 7.0 Hz, 3H), 0.26 (s, 9H). ^{13}C NMR (126 MHz, $CDCl_3$) 163.09 (d, *J* = 251.8 Hz), 157.72, 150.80 (d, *J* = 6.7 Hz), 134.02, 133.69 (d, *J* = 1.8 Hz), 129.16, 122.69 (d, *J* = 3.2 Hz), 113.91 (d, *J* = 20.6 Hz), 113.74, 109.09 (d, *J* = 16.2 Hz), 99.18 (d, *J* = 2.9 Hz), 98.16, 55.24, 39.81, 39.22 (d, *J* = 1.5 Hz), 32.73, 22.06, –0.08. ^{19}F NMR (470 MHz, $CDCl_3$) –110.11, –110.13, –110.13, –110.15. HRMS (ESI) exact mass calculated for [M+H$^+$] $C_{22}H_{27}FNaOSi$ requires 377.1707, Found 377.1706. HPLC Conditions: Chiracel IA with Guard Column, Hexane 100%, flow rate –0.3 mL/min, Injection vol. – 5 µL, t_R (minor) = 46.42 min, t_R (major) = 50.66 min. er 95.3:4.7.

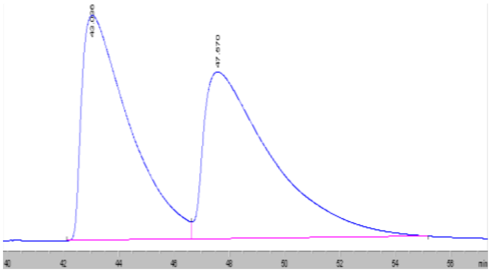

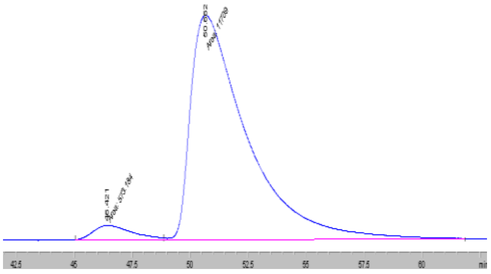

Peak #	RetTime [min]	Type	Width [min]	Area [mAU*s]	Height [mAU]	Area %
1	43.036	BV	1.6416	7.32053e4	608.47473	48.6008
2	47.570	VB	2.2485	7.74203e4	451.05579	51.3992

Peak #	RetTime [min]	Type	Width [min]	Area [mAU*s]	Height [mAU]	Area %
1	46.421	MF	2.0547	573.18378	4.64928	4.6668
2	50.662	FM	2.8697	1.17090e4	68.00415	95.3332

(*R*)–1–ethynyl–4–(4–(4–methoxyphenyl)butan–2–yl)–2–(trifluoromethoxy)benzene: A solution of *o*–trifluoromethoxy–*p*–bromophenylacetylene **2h** (0.2 mmol, 1.2 equiv) in THF (0.7

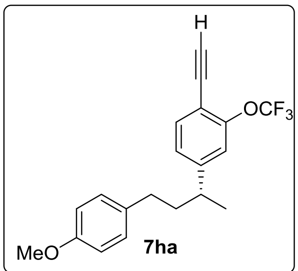

7ha

mL, 0.3 M) was cooled to –78 °C and treated with *n*–BuLi (1.2 equiv, 1.6 M in hexanes) and the mixture was stirred at this temperature for 1 h. The pinacol boronic ester **9a** (52 mg, 0.18 mmol, 1.0 eq.) was added dropwise as a solution in THF (0.6 mL, 0.3 M). The mixture was stirred at –78 °C for 1 h at which point ^{11}B NMR analysis showed complete formation of the 'ate' complex [^{11}B NMR (96 MHz, THF) δ_B ~ 8 ppm]. The reaction mixture was warmed to 0 °C with an ice bath and TFE (0.6 mL) was added. Then, a solution of NBS (0.27 mmol; 1.5 equiv) in TFE (0.3 M) was added dropwise. After 5 min at 0 °C, $Na_2S_2O_3$ (sat. aq.) was added and the reaction mixture was allowed to warm to room temperature. The reaction mixture was diluted with ether (20 mL) and water. The layers were separated and the aqueous layer was extracted again with ether (20 mL). The combined organic layers were dried (Na_2SO_4), filtered and concentrated under vacuum. The crude material was then dissolved in MeOH (1 mL) and K_2CO_3 (0.54 mmol; 3 equiv) was added and stirred for 5 h. The reaction mixture was diluted with ether (20 mL) and water. The layers were separated and the aqueous layer was extracted again with ether (20 mL). The combined organic layers were dried (Na_2SO_4), filtered and concentrated under vacuum. The crude material was adsorbed on silica and purified by flash column chromatography on silica gel eluting with *n*–hexane/Et_2O to afford the title product **7ha** (20 mg, 32%). $[\alpha]_D^{23}$ = –27.50 (*c* 0.8, $CHCl_3$); R_f (95/5 petroleum ether/EtOAc): 0.65; IR (film) ν_{max}/cm^{-1}: 2959, 2931, 1612, 1511, 1243, 1215, 1162, 1036, 829; ^{1}H NMR (500 MHz, $CDCl_3$) 7.48 (d, *J* = 8.2 Hz, 1H), 7.14 – 7.05 (m, 2H), 7.01 (d, *J* = 8.7 Hz, 2H), 6.81 (d, *J* = 8.7 Hz, 2H), 3.78 (s, 3H), 3.26 (s, 1H), 2.72 (q, *J* = 7.1 Hz, 1H), 2.53 – 2.35 (m, 2H), 1.95 – 1.79 (m, 2H), 1.25 (d, *J* = 6.9 Hz, 3H). ^{13}C NMR (126 MHz, $CDCl_3$) 157.77, 150.53, 149.91, 134.09, 133.82, 129.16, 125.48, 120.53 (q, *J* = 258.3 Hz), 120.00, 114.21, 113.77, 81.73, 77.84, 55.24, 39.73, 39.21, 32.70, 22.06. ^{19}F NMR (470 MHz, $CDCl_3$) –57.48. HRMS (EI) exact mass calculated for [M$^+$] $C_{20}H_{19}O_2F_3$ requires 348.1337, found 348.1334. HPLC Conditions: Chiracel IB with Guard Column, Hexane 100%, flow rate – 1 mL/min, Injection vol. – 5 µL, t_R (minor) = 29.93 min, t_R (major) = 39.16 min. er 94.9:5.1

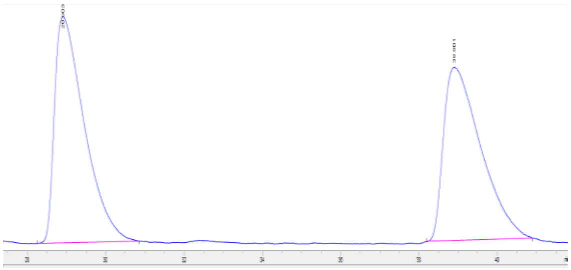

#	Time	Type	Area	Height	Width	Area%	Symmetry
1	28.903	VV R	4257.2	80.5	0.6188	51.021	0.388
2	38.901	BV R	4086.8	61.8	0.7741	48.979	0.422

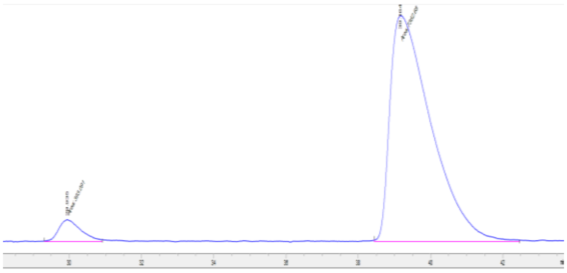

#	Time	Type	Area	Height	Width	Area%	Symmetry
1	29.935	MM	393.1	9.1	0.7209	5.068	0.612
2	39.164	MM	7362.7	92.9	1.3213	94.932	0.342

(*R*)–((3–(dimethoxymethyl)–4–(4–(4–methoxyphenyl)butan–2–yl)phenyl)ethynyl)trimethyl silane (7i):

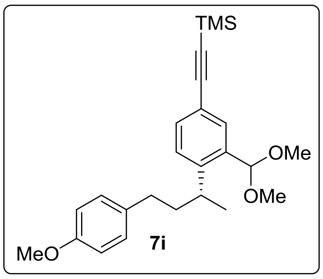

The starting boronic ester **6a** (52 mg, 0.18 mmol) was subjected to the conditions in General Procedure C. The crude product was purified by column chromatography (petroleum ether/EtOAc – 95/5) to afford the title product **7i** (49 mg, 66%). $[\alpha]_D^{23}$ = –9.52 (*c* 0.84, $CHCl_3$); R_f (95/5 petroleum ether/EtOAc): 0.4; IR (film) ν_{max}/cm^{-1}: 2970, 2900, 1247, 1055, 856; ^{1}H NMR (500 MHz, $CDCl_3$) 7.67 (d, *J* = 1.8 Hz, 1H), 7.43 (dd, *J* = 8.1, 1.8 Hz, 1H), 7.25 (d, *J* = 8.1 Hz, 1H), 6.99 (d, *J* = 8.7 Hz, 2H), 6.79 (d, *J* = 8.7 Hz, 2H), 5.30 (s, 1H), 3.77 (s, 3H), 3.29 (s, 3H), 3.19 (s, 3H), 3.18 – 3.10 (m, 1H), 2.51 – 2.32 (m, 2H), 1.88 (m, 2H), 1.20 (d, *J* = 6.8 Hz, 3H), 0.24 (s, 9H). ^{13}C NMR (126 MHz, $CDCl_3$) 157.69, 146.46, 134.98, 134.32, 132.20, 130.51, 129.14, 125.91, 119.97, 113.73, 105.31, 100.41, 93.34, 55.24, 53.30, 51.98, 39.67, 32.94, 32.87, 22.51, 0.01. HRMS (ESI) exact mass calculated for [M+Na$^+$] $C_{25}H_{34}NaO_3Si$ requires 433.2169 found 433.2171. SFC Conditions: Chiracel IB Column, 5% [10% IPA:Hexane]/$CO_{2(scf)}$, flow rate – 4 mL/min, Pressure:125 bar, Injection vol. – 10 μL, t_R (minor) = 7.66 min, t_R (major) = 9.4 min. er 95.7:4.3.

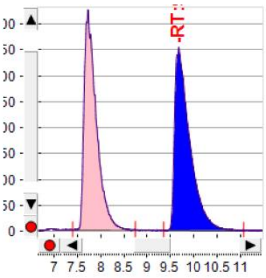

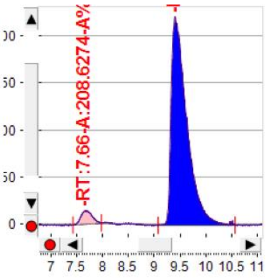

Peak #	Area %	Area	Ret. Time
1	49.3533	7755.6873	7.73 min
2	50.6467	7958.9517	9.68 min

Peak #	Area %	Area	Ret. Time
1	4.3313	208.6274	7.66 min
2	95.6687	4608.0815	9.4 min

(*R*)–trimethyl((4–(1–phenylhept–6–en–3–yl)phenyl)ethynyl)silane (8b): The starting

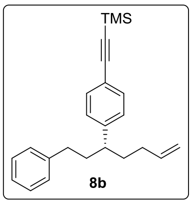

boronic ester **6b** (52 mg, 0.18 mmol) was subjected to the conditions in General Procedure C. The crude product was purified by column chromatography (petroleum ether/EtOAc – 95/5) to afford the title product **8b** (52 mg, 83%). $[\alpha]_D^{23}$ = +7.64 (*c* 1.44, $CHCl_3$); R_f (95/5 petroleum ether/EtOAc): 0.72; IR (film) ν_{max}/cm^{-1}: 2959, 2927, 1511, 1247, 861, 840; ^{1}H NMR (500 MHz, $CDCl_3$) 7.43 (d, *J* = 8.4 Hz, 2H), 7.27 – 7.22 (m, 2H), 7.19 – 7.13 (m, 1H), 7.11 (d, *J* = 8.4 Hz, 2H), 7.07 (m, 2H), 5.79 – 5.65 (m, 1H), 4.95 – 4.83 (m, 2H), 2.56 (tt, *J* = 9.7, 5.0 Hz, 1H), 2.41 (t, *J* = 8.0 Hz, 2H), 1.96 (dddd, *J* = 13.7, 8.8, 7.6, 4.9 Hz, 1H), 1.91 – 1.81 (m, 3H), 1.73 (dtd, *J* = 15.8, 7.6, 5.1 Hz, 1H), 1.69 – 1.59 (m, 1H), 0.25 (d, *J* = 0.5 Hz, 9H). ^{13}C NMR (126 MHz, $CDCl_3$) 145.91, 142.26, 138.46, 132.08, 128.30, 128.26, 127.74, 125.67, 120.74, 114.57, 105.23, 93.54, 44.88, 38.36, 35.96, 33.68, 31.57, 0.01. HRMS (EI) exact mass calculated for $[M^+]$ $C_{24}H_{30}Si$ requires 346.2117, found 346.2120. SFC Conditions: Chiracel IB Column, 5% [10% IPA:Hexane]/$CO_{2(scf)}$, flow rate – 2 mL/min, Pressure:100 bar, Injection vol. – 10 µL, t_R (major) = 10.7 min, t_R (minor) = 11.4 min. er 90.5:9.5

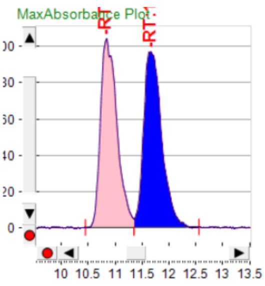

Peak #	Area %	Area	Ret. Time
1	49.5233	2210.0052	10.84 min
2	50.4767	2252.5523	11.66 min

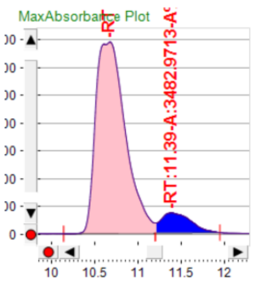

Peak #	Area %	Area	Ret. Time
1	90.5106	33220.6524	10.67 min
2	9.4894	3482.9713	11.39 min

(*S*)–((4–(1–cyclopropyl–3–phenylpropyl)phenyl)ethynyl)trimethylsilane (8c): The starting

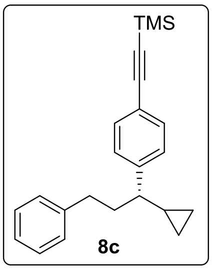

boronic ester **6c** (48 mg, 0.18 mmol) was subjected to the conditions in General Procedure C. The crude product was purified by column chromatography (petroleum ether/EtOAc – 95/5) to afford the title product **8c** (52 mg, 87%). $[\alpha]_D^{23}$ = +48.21 (*c* 0.27, $CHCl_3$); R_f (95/5 petroleum ether/EtOAc): 0.72; IR (film) ν_{max}/cm^{-1}: 2958, 2925, 1497, 1248, 864, 698; ^{1}H NMR (500 MHz, $CDCl_3$) 7.43 (d, *J* = 8.4 Hz, 2H), 7.28 – 7.22 (m, 2H), 7.20 – 7.11 (m, 3H), 7.11 – 7.03 (m, 2H), 2.49 (m, 2H), 2.15 – 1.96 (m, 2H), 1.79 (td, *J* = 9.2, 5.5 Hz, 1H), 1.03 – 0.90 (m, 1H), 0.64 – 0.53 (m, 1H), 0.38 – 0.31 (m, 1H), 0.25 (s, 9H), 0.18 (dt, *J* = 9.9, 4.6 Hz, 1H), 0.01 (dq, *J* =

10.2, 5.0 Hz, 1H). ^{13}C NMR (126 MHz, $CDCl_3$) 146.22, 142.33, 131.97, 128.28, 128.25, 127.53, 125.65, 120.69, 105.29, 93.46, 50.30, 38.00, 33.60, 17.44, 5.51, 3.55, 0.02. HRMS (EI) exact mass calculated for [M$^+$] $C_{23}H_{28}Si$ requires 332.1960, found 332.1965. SFC Conditions: Whelk–01 Column, 5% [Hexane]/$CO_{2(scf)}$, flow rate – 2 mL/min, Pressure:100 bar, Injection vol. – 10 µL, t_R (minor) = 14.7 min, t_R (major) = 15.6 min. er 98:2.

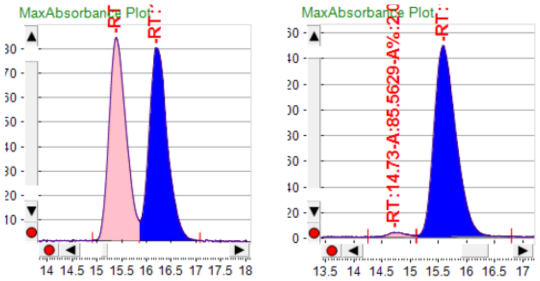

Peak #	Area %	Area	Ret. Time
1	49.4689	1994.211	15.39 min
2	50.5311	2037.0269	16.19 min

Peak #	Area %	Area	Ret. Time
1	2.0719	85.5629	14.73 min
2	97.9281	4044.1756	15.59 min

tert–butyl(((2S,3R)–2,4–dimethyl–3–(4–((trimethylsilyl)ethynyl)phenyl)pentyl)oxy)dimethylsilane (8d): The starting boronic ester **6d** (62 mg, 0.18 mmol) was subjected to the conditions in General Procedure C. The crude product was purified by column chromatography (petroleum ether/EtOAc – 95/5) to afford the title product **8d** (47 mg, 65%). $[\alpha]_D^{23}$ = –23.75 (*c* 0.8, $CHCl_3$); R_f (95/5 petroleum ether/EtOAc): 0.9; IR (film) ν_{max}/cm^{-1}: 2956, 2928, 2857, 1249, 1094, 865, 863; ^{1}H NMR (500 MHz, $CDCl_3$) 7.39 (d, *J* = 8.2 Hz, 2H), 7.07 (d, *J* = 8.2 Hz, 2H), 3.31 – 3.14 (m, 2H), 2.51 (dd, *J* = 9.1, 5.7 Hz, 1H), 2.22 – 1.99 (m, 2H), 0.94 (d, *J* = 6.6 Hz, 3H), 0.93 (s, 9H), 0.72 (d, *J* = 6.6 Hz, 6H), 0.27 (s, 9H), 0.03 (s, 6H). ^{13}C NMR (126 MHz, $CDCl_3$) 142.34, 131.19, 129.76, 120.29, 105.44, 93.38, 66.40, 53.27, 36.35, 28.30, 25.95, 21.34, 20.79, 18.26, 12.34, 0.04, –5.36, –5.38. HRMS (EI) exact mass calculated for [M–Me$^+$] $C_{23}H_{39}OSi_2$ requires 387.2539, found 387.2531.

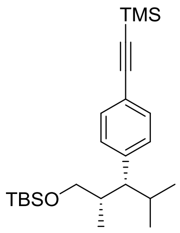

8d

((4–((1R,2S,5R)–2–isopropyl–5–methylcyclohexyl)phenyl)ethynyl)trimethylsilane (8e): The starting boronic ester **6e** (49 mg, 0.18 mmol) was subjected to the conditions in General Procedure C. The crude product was purified by column chromatography (petroleum ether/EtOAc – 95/5) to afford the title product **8e** (44 mg, 78%). $[\alpha]_D^{23}$ = –18.33 (*c* 0.6, $CHCl_3$); R_f (95/5 petroleum ether/EtOAc): 0.72; IR (film) ν_{max}/cm^{-1}: 2957, 2919, 1248, 1066, 863, 841; ^{1}H NMR (500 MHz, $CDCl_3$) 7.38 (d, *J* = 8.2 Hz, 2H), 7.08 (d, *J* = 8.2 Hz, 2H), 2.40 (td, *J* = 11.6, 3.5 Hz, 1H),

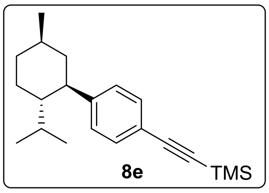

8e

1.80 (dq, J = 12.6, 3.1 Hz, 1H), 1.77 – 1.68 (m, 2H), 1.51 – 1.30 (m, 3H), 1.18 – 1.07 (m, 2H), 1.00 (tdd, J = 12.8, 11.4, 3.3 Hz, 1H), 0.89 (d, J = 6.5 Hz, 3H), 0.77 (d, J = 7.1 Hz, 3H), 0.64 (d, J = 6.9 Hz, 3H), 0.23 (s, 9H). ^{13}C NMR (126 MHz, $CDCl_3$) 147.45, 131.97, 127.38, 120.28, 105.39, 93.20, 47.99, 47.42, 44.89, 35.23, 33.16, 27.43, 24.50, 22.47, 21.43, 15.32, 0.01. HRMS (EI) exact mass calculated for [M^+] $C_{21}H_{32}Si$ requires 312.2273 found 312.2262.

***(R)*–((4–(7–azido–1–phenylheptan–3–yl)phenyl)ethynyl)trimethylsilane (8f):** The starting boronic ester **6f** (50 mg, 0.18 mmol) was subjected to the conditions in General Procedure C. The crude product was purified by column chromatography (petroleum ether/EtOAc – 95/5) to afford the title product **8f** (42 mg, 73%). $[\alpha]_D^{23}$ = +22.95 (c 1.3, $CHCl_3$); R_f (95/5 petroleum ether/EtOAc): 0.72; IR (film) ν_{max}/cm^{-1}: 2933, 2859, 2157, 2094, 1499, 1249, 864, 759; ^{1}H NMR (500 MHz, $CDCl_3$) 7.44 (d, J = 7.8 Hz, 2H), 7.25 (d, J = 7.1 Hz, 2H), 7.19 – 7.04 (m, 5H), 3.24 – 3.07 (m, 2H), 2.52 (m, 1H), 2.41 (m, 2H), 2.03 – 1.78 (m, 2H), 1.71 – 1.40 (m, 4H), 1.28 – 1.05 (m, 2H), 0.26 (s, 9H). ^{13}C NMR (126 MHz, $CDCl_3$) 145.82, 142.16, 132.13, 128.31, 128.28, 127.63, 125.72, 120.85, 105.16, 93.63, 51.27, 45.36, 38.33, 36.37, 33.65, 28.81, 24.64, 0.01. HRMS (EI) exact mass calculated for [M^+] $C_{24}H_{31}N_3Si$ requires 389.2287, found 389.2283. HPLC Conditions: Chiracel IB with Guard Column, Hexane:IPA 99.9:0.1, flow rate – 1 mL/min, Injection vol. – 5 μL, t_R (minor) = 11.7 min, t_R (major) = 14.7 min. er 97.5:2.5

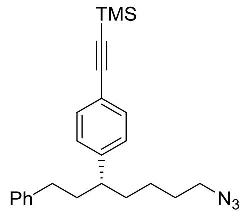

8f

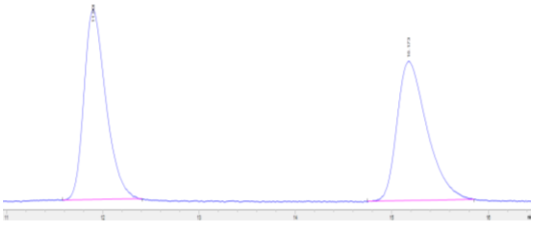

#	Time	Type	Area	Height	Width	Area%	Symmetry
1	11.9	VV R	1744.3	109.5	0.2039	49.968	0.694
2	15.173	VV R	1746.5	80.4	0.257	50.032	0.604

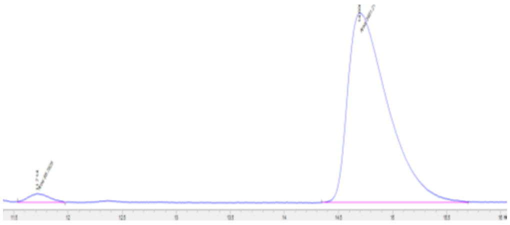

#	Time	Type	Area	Height	Width	Area%	Symmetry
1	11.714	MM	88.8	6.3	0.2347	2.500	0.742
2	14.696	MM	3461.2	134.2	0.4299	97.500	0.419

tert–butyl 4–(4–((trimethylsilyl)ethynyl)phenyl)piperidine–1–carboxylate (8g): The starting boronic ester **6g** (53 mg, 0.18 mmol) was subjected to the conditions in General Procedure C. The crude product was purified by column chromatography (petroleum ether/EtOAc – 95/5) to afford the title product **8g** (44 mg, 68%). R_f (95/5 petroleum ether/EtOAc): 0.3; IR (film) ν_{max}/cm^{-1}: 2969, 2956, 2933, 1694, 1422, 1169, 864, 842, 760; ^{1}H NMR (500 MHz, $CDCl_3$) 7.40 (d, J = 8.2 Hz, 2H), 7.12 (d, J = 8.2 Hz, 2H), 4.24 (bs, 2H), 2.78 (bs, 2H), 2.62 (tt, J = 12.2, 3.5 Hz, 1H), 1.79 (d, J = 12.9 Hz, 2H), 1.65 – 1.56 (m, 2H), 1.47 (s, 9H), 0.23 (s, 9H). ^{13}C NMR (126 MHz, $CDCl_3$) 154.78, 146.29, 132.09,

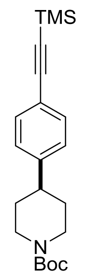

8g

126.64, 121.06, 105.03, 93.68, 79.46, 44.21, 42.62, 32.95, 28.46, –0.02. HRMS (ESI) exact mass calculated for [M+Na$^+$] $C_{21}H_{31}NNaO_2Si$ requires 380.2016 found 380.2031.

***(S)*–trimethyl((4–(3–methyl–1–phenylpentan–3–yl)phenyl)ethynyl)silane (8h):** The starting

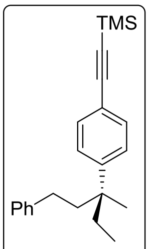

8h

boronic ester **6h** (49 mg, 0.18 mmol) was subjected to the conditions in General Procedure C. The crude product was purified by column chromatography (petroleum ether/EtOAc – 95/5) to afford the title product **8h** (26 mg, 43%). $[\alpha]_D^{23}$ = –36.95 (*c* 0.92, $CHCl_3$); R_f (95/5 petroleum ether/EtOAc): 0.72; IR (film) ν_{max}/cm^{-1}: 2963, 2928, 1501, 1249, 864, 841; ^{1}H NMR (500 MHz, $CDCl_3$) 7.44 (d, *J* = 8.6 Hz, 2H), 7.27 (d, *J* = 8.6 Hz, 2H), 7.25 – 7.21 (m, 2H), 7.17 – 7.11 (m, 1H), 7.08 – 7.04 (m, 2H), 2.41 (td, *J* = 13.0, 5.0 Hz, 1H), 2.18 (td, *J* = 13.0, 4.3 Hz, 1H), 1.97 (td, *J* = 13.2, 4.3 Hz, 1H), 1.86 – 1.71 (m, 2H), 1.60 (dq, *J* = 14.8, 7.4 Hz, 1H), 1.34 (s, 3H), 0.67 (t, *J* = 7.4 Hz, 3H), 0.25 (s, 9H). ^{13}C NMR (126 MHz, $CDCl_3$) 148.15, 143.00, 131.75, 128.28, 128.18, 126.43, 125.56, 120.03, 105.26, 93.51, 45.24, 41.45, 35.61, 30.82, 22.98, 8.53, 0.01. HRMS (EI) exact mass calculated for [M$^+$] $C_{23}H_{30}Si$ requires 334.2117, found 334.2125. HPLC Conditions: Chiracel IA+IA with Guard Column, Hexane 100%, flow rate – 0.5 mL/min, Injection vol. – 5 µL, t_R (major) = 18.29 min, t_R (minor) = 19.43 min. er 99:1.

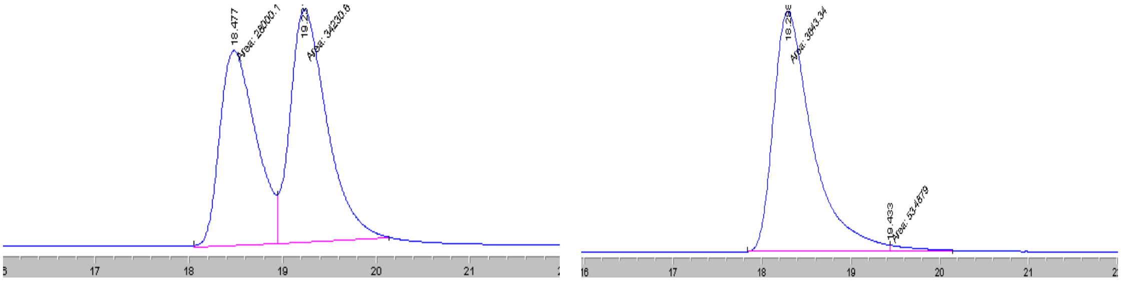

Peak #	RetTime [min]	Type	Width [min]	Area [mAU*s]	Height [mAU]	Area %
1	18.477	MF	0.4502	2.80001e4	1036.69006	44.9941
2	19.231	FM	0.4607	3.42306e4	1238.26746	55.0059

Peak #	RetTime [min]	Type	Width [min]	Area [mAU*s]	Height [mAU]	Area %
1	18.288	MF	0.5061	3643.34473	119.98144	98.5531
2	19.433	FM	0.3027	53.48792	2.94491	1.4469

((4–((3r,5r,7r)–adamantan–1–yl)phenyl)ethynyl)trimethylsilane (8i): The starting boronic

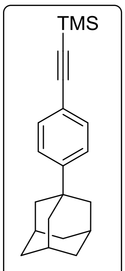

8i

ester **6i** (45 mg, 0.18 mmol) was subjected to the conditions in General Procedure C. The crude product was purified by column chromatography (petroleum ether/EtOAc – 95/5) to afford the title product **8i** (44 mg, 79%). R_f (95/5 petroleum ether/EtOAc): 0.72; IR (film) ν_{max}/cm^{-1}: 2959, 2903, 2849, 1248, 1056, 866; ^{1}H NMR (500 MHz, $CDCl_3$) 7.41 (d, *J* = 8.3 Hz, 2H), 7.29 (d, *J* = 8.3 Hz, 2H), 2.09 (s, 3H), 1.89 (d, *J* = 2.7 Hz, 6H), 1.76 (m, 6H), 0.24 (s, 9H). ^{13}C NMR (126 MHz, $CDCl_3$) 151.92, 131.70, 124.75,

120.08, 105.41, 93.22, 42.93, 36.71, 36.30, 28.85, 0.03. HRMS (EI) exact mass calculated for [M+] $C_{21}H_{28}Si$ requires 308.1960, found 308.1950.

D. General Procedure 2: C(*sp*[3])–C(*sp*[2]) coupling of boronic esters with *p*–bromophenylacetylenes: Boron incorporation

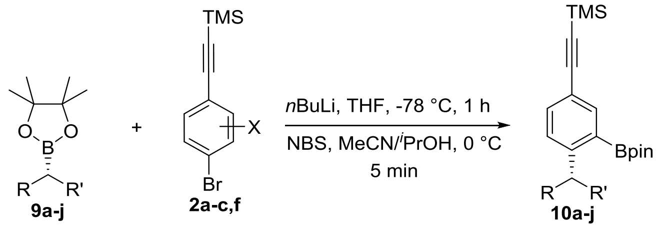

A solution of *p*–bromophenylacetylene **2a-c,f** (0.2 mmol, 1.2 equiv) in THF (0.7 mL, 0.3 M) was cooled to –78 °C and treated with *n*–BuLi (1.2 eq., 1.6 M in hexanes); the mixture was stirred at this temperature for 1 h. The boronic ester **9a-j** (0.18 mmol, 1.0 equiv) was added dropwise as a solution in THF (0.6 mL, 0.5 M). The mixture was stirred at –78 °C for 1 h at which point ^{11}B NMR analysis showed complete formation of the 'ate' complex [^{11}B NMR (96 MHz, THF) δ_B ~ 8 ppm]. At that time, the reaction mixture was concentrated under vacuum to dryness and redissolved with 2:1 mixture of dry ^{i}PrOH/MeCN (2.0 mL); the mixture was cooled to 0 °C. A freshly prepared solution of NBS (0.27 mmol, 1.5 equiv) in another portion of MeCN (0.5 mL, 0.6 M) was added dropwise. After 5 min at 0 °C, $Na_2S_2O_3$ (sat. aq.) was added and the reaction mixture was allowed to warm to room temperature. The reaction mixture was diluted with DCM and water. The layers were separated and the aqueous layer was extracted with DCM. The combined organic layers were dried (Na_2SO_4), filtered and concentrated under vacuum. The crude material was adsorbed on silica and purified by flash column chromatography on silica gel eluting with *n*–hexane/Et_2O.

((4–cyclohexyl–3–(4,4,5,5–tetramethyl–1,3,2–dioxaborolan–2–yl)phenyl)ethynyl)trimethyl

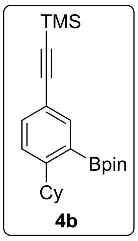

silane (4b): The starting boronic ester **1a** (38 mg, 0.18 mmol) was subjected to the conditions in General Procedure D. The crude product was purified by column chromatography (petroleum ether/EtOAc – 95/5) to afford the title product **4b** (49 mg, 72%). R_f (95/5 petroleum ether/EtOAc): 0.7; IR (film) ν_{max}/cm^{-1}: 2985, 2970, 1511, 1106, 843; ^{1}H NMR (500 MHz, $CDCl_3$) 7.82 (d, *J* = 1.8 Hz, 1H), 7.44 (dd, *J* = 8.1, 1.9 Hz, 1H), 7.19 (d, *J* = 8.1 Hz, 1H), 3.23 (m, 1H), 1.78 (m, 5H), 1.48 – 1.15 (m, 17H), 0.23 (s, 9H). ^{13}C NMR (126 MHz, $CDCl_3$) 154.92, 139.14, 134.04, 124.81, 119.58, 105.49, 93.05, 83.57,

42.25, 34.73, 27.02, 26.25, 24.80, 0.04. ^{11}B NMR (96 MHz, $CDCl_3$) 30.00. HRMS (ESI) exact mass calculated for [M+H$^+$] $C_{23}H_{36}BO_2Si$ requires 383.2577 found 383.2575.

***(R)*–((4–(4–(4–methoxyphenyl)butan–2–yl)–3–(4,4,5,5–tetramethyl–1,3,2–dioxaborolan–2–yl)phenyl)ethynyl)trimethylsilane (10a):** The starting boronic ester **9a** (52 mg, 0.18 mmol) was subjected to the conditions in General Procedure D. The crude product was purified by column chromatography (petroleum ether/EtOAc – 95/5) to afford the title product **10a** (65 mg, 78%). $[\alpha]_D^{23}$ = +18.75 (*c* 0.96, $CHCl_3$); R_f (95/5 petroleum ether/EtOAc): 0.72; IR (film) ν_{max}/cm^{-1}: 2959, 2928, 1511, 1338, 1245, 1142, 841, 759, 677; ^{1}H NMR (500 MHz, $CDCl_3$) 7.86 (d, *J* = 1.9 Hz, 1H), 7.48 (dd, *J* = 8.1 Hz, 1.9 Hz, 1H), 7.24 (d, *J* = 8.1 Hz, 1H), 7.01 (d, *J* = 8.6 Hz, 2H), 6.78 (d, *J* = 8.6 Hz, 2H), 3.76 (s, 3H), 3.56 (sx, *J* = 7.0 Hz, 1H), 2.51 (ddd, *J* = 13.8, 10.6, 6.0 Hz, 1H), 2.39 (ddd, *J* = 13.8, 10.6, 5.4 Hz, 1H), 2.01 – 1.68 (m, 2H), 1.32 (s, 6H), 1.31 (s, 6H), 1.23 (d, *J* = 6.8 Hz, 3H), 0.24 (s, 9H); ^{13}C NMR (126 MHz, $CDCl_3$) 157.54, 154.61, 139.31, 134.89, 134.24, 129.11, 129.10, 125.04, 119.72, 113.64, 105.40, 93.21, 83.60, 55.21, 40.53, 36.61, 33.06, 24.84, 24.77, 22.32, 0.04; ^{11}B NMR (96 MHz, $CDCl_3$) 30.16. HRMS (ESI) exact mass calculated for [M+Na$^+$] $C_{28}H_{39}BNaO_3Si$ requires 485.2659 found 485.2659. SFC Conditions: Chiracel IB Column, 5% [10% IPA:Hexane]/$CO_{2(scf)}$, flow rate – 4 mL/min, Pressure: 125 bar, Injection vol. – 10 µL, t_R (minor) = 7.3 min, t_R (major) = 8.65 min.

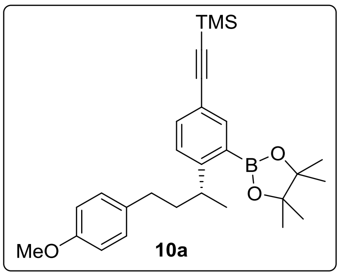

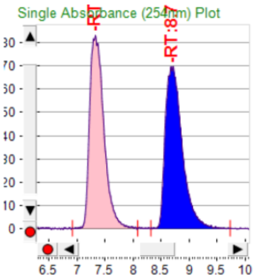

Peak #	Area %	Area	Ret. Time
1	50.1339	1460.9724	7.33 min
2	49.8661	1453.1666	8.71 min

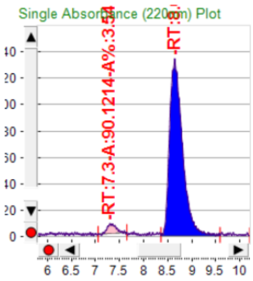

Peak #	Area %	Area	Ret. Time
1	3.5453	90.1214	7.3 min
2	96.4547	2451.8979	8.65 min

***(R)*–((4–(4–(4–methoxyphenyl)butan–2–yl)–2–methyl–3–(4,4,5,5–tetramethyl–1,3,2–dioxaborolan–2–yl)phenyl)ethynyl)trimethylsilane (10ab):** The starting boronic ester **9a** (52 mg, 0.18 mmol) was subjected to the conditions in General Procedure D. The crude product was purified by column chromatography (petroleum ether/EtOAc – 95/5) to afford the title product **10ab** (68 mg, 80%). $[\alpha]_D^{23}$ = +33.80

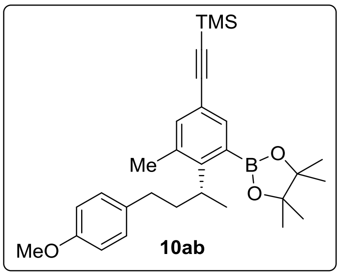

(c 1.2, $CHCl_3$); R_f (95/5 petroleum ether/EtOAc): 0.72; IR (film) ν_{max}/cm^{-1}: 2960, 2928, 1511, 1307, 1245, 840; ^{1}H NMR (500 MHz, $CDCl_3$) 7.31 (d, J = 8.0 Hz, 1H), 7.14 (d, J = 8.0 Hz, 1H), 6.99 (d, J = 8.6 Hz, 2H), 6.79 (d, J = 8.6 Hz, 2H), 3.77 (s, 3H), 2.96 (sx, J = 7.0 Hz, 1H), 2.47 – 2.37 (m, 2H), 2.24 (s, 3H), 1.90 – 1.75 (m, 2H), 1.42 (s, 12H), 1.15 (d, J = 6.9 Hz, 3H), 0.22 (s, 9H). ^{13}C NMR (126 MHz, $CDCl_3$) 157.61, 145.38, 138.07, 134.44, 130.67, 129.16, 125.95, 123.12, 113.67, 106.10, 93.69, 84.06, 77.25, 76.99, 76.74, 55.24, 39.28, 33.61, 32.77, 25.07, 25.04, 21.69, 18.90, 0.05. ^{11}B NMR (96 MHz, $CDCl_3$) 30.64. HRMS (ESI) exact mass calculated for [M+Na$^+$] $C_{29}H_{41}BNaO_3Si$ requires 499.2816 found 499.2803. SFC Conditions: Chiracel IB Column, 5% [10% IPA:Hexane]/$CO_{2(scf)}$, flow rate – 4 mL/min, Pressure: 125 bar, Injection vol. – 10 μL, t_R (minor) = 11.2 min, t_R (major) = 12.0 min. er 96:4

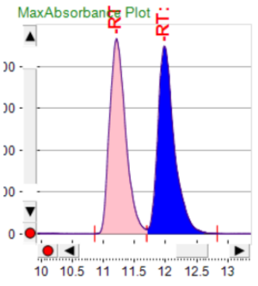

Peak #	Area %	Area	Ret. Time
1	50.3524	16734.5104	11.21 min
2	49.6476	16500.2537	11.98 min

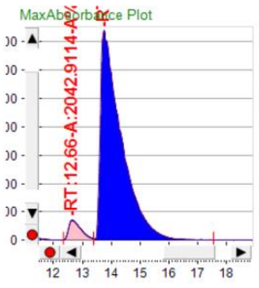

Peak #	Area %	Area	Ret. Time
1	5.2579	2042.9114	12.66 min
2	94.7421	36810.881	13.75 min

(R)–((4–(4–(4–methoxyphenyl)butan–2–yl)–2–methyl–5–(4,4,5,5–tetramethyl–1,3,2–dioxaborolan–2–yl)phenyl)ethynyl)trimethylsilane (10ac1): The starting boronic ester **9a** (49 mg, 0.18 mmol) was subjected to the conditions in General Procedure D. The crude product was purified by column chromatography (petroleum ether/EtOAc – 95/5) to afford the regioisomeric products (**10ac1** and **10ac2**) (37.5 mg, 44%; 30 mg, 35%). $[\alpha]_D^{23}$ = +23.53 (c 0.68, $CHCl_3$); R_f (95/5 petroleum ether/EtOAc): 0.72; IR (film) ν_{max}/cm^{-1}: 2978, 2927, 1511, 1301, 1243, 1136, 841; ^{1}H NMR (500 MHz, $CDCl_3$) 7.83 (s, 1H), 7.11 (s, 1H), 7.01 (d, J = 8.6 Hz, 2H), 6.78 (d, J = 8.6 Hz, 2H), 3.76 (s, 3H), 3.60 – 3.47 (m, 1H), 2.52 (ddd, J = 13.8, 10.7, 6.0 Hz, 1H), 2.46 – 2.35 (m, 4H), 1.94 – 1.68 (m, 2H), 1.31 (s, 6H), 1.29 (s, 6H), 1.23 (d, J = 6.9 Hz, 3H), 0.24 (s, 9H); ^{13}C NMR (126 MHz, $CDCl_3$) 157.51, 154.70, 143.27, 139.76, 135.00, 129.11, 126.22, 119.79, 113.62, 104.29, 97.09, 83.40, 55.22, 40.56, 36.43, 33.10, 24.84, 24.76, 22.29, 20.97, 0.12; ^{11}B NMR (96 MHz, $CDCl_3$) 30.48. HRMS (ESI) Mass calculated for [M+Na$^+$] $C_{29}H_{42}BNaO_3Si$ requires 499.2816 found 499.2835. SFC Conditions: Chiracel IB

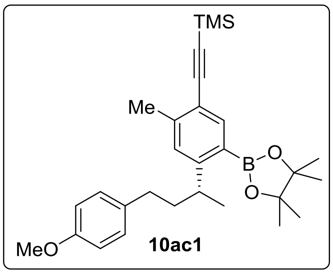

Column, 5% [10% IPA:Hexane] /$CO_{2(scf)}$, flow rate – 3 mL/min, Pressure: 125 bar, Injection vol. – 10 μL, t_R (minor) = 9.7 min, t_R (major) = 10.4 min. er 95.3:4.7.

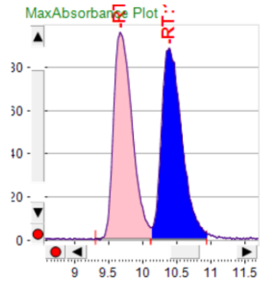

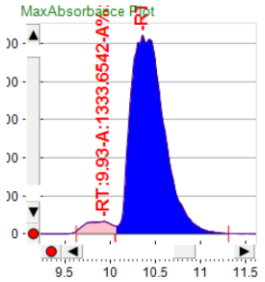

Peak #	Area %	Area	Ret. Time
1	49.4062	1809.8629	9.67 min
2	50.5938	1853.366	10.39 min

Peak #	Area %	Area	Ret. Time
1	4.6738	1333.6542	9.93 min
2	95.3262	27201.2616	10.35 min

***(R)*–((4–(4–(4–methoxyphenyl)butan–2–yl)–2–methyl–3–(4,4,5,5–tetramethyl–1,3,2–dioxaborolan–2–yl)phenyl)ethynyl)trimethylsilane (10ac2):**

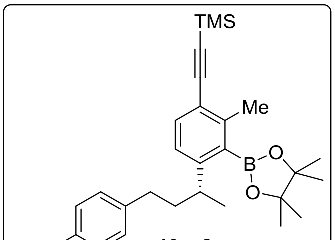

10ac2

[α]$_D^{23}$ = +26.14 (*c* 0.88, $CHCl_3$); R_f (95/5 petroleum ether/EtOAc): 0.72; **IR** (film) ν_{max}/cm^{-1}: 2958, 2927, 1511, 1332, 1245, 1141, 858, 841, 759; ^{1}H NMR (500 MHz, $CDCl_3$) 7.40 (d, *J* = 8.1 Hz, 1H), 7.06 – 6.96 (m, 3H), 6.79 (d, *J* = 8.6 Hz, 2H), 3.77 (s, 3H), 2.78 (sx, *J* = 6.9 Hz, 1H), 2.54–2.49 (m, 4H), 2.39 (ddd, *J* = 13.9, 10.5, 5.6 Hz, 1H), 1.94 (dddd, *J* = 13.4, 10.6, 7.7, 5.6 Hz, 1H), 1.81 (dddd, *J* = 13.4, 10.6, 6.8, 5.8 Hz, 1H), 1.35 (s, 12H), 1.24 (d, *J* = 6.8 Hz, 3H), 0.24 (s, 9H). ^{13}C NMR (126 MHz, $CDCl_3$) 157.57, 151.40, 143.33, 134.68, 133.16, 129.15, 121.76, 120.18, 113.68, 104.76, 97.22, 83.94, 55.22, 39.76, 39.57, 33.07, 25.07, 24.93, 22.73, 20.62, 0.11. ^{11}B NMR (96 MHz, $CDCl_3$) 33.41. HRMS (ESI) Mass calculated for [M+Na$^+$] $C_{29}H_{41}BNaO_3Si$ requires 499.2816 found 499.2811. SFC Conditions: Chiracel IB Column, 5% [10% IPA:Hexane] /$CO_{2(scf)}$, flow rate – 4 mL/min, Pressure: 125 bar, Injection vol. – 10 μL, t_R (minor) = 9.3 min, t_R (major) = 11.5 min. er 94.5:5.5

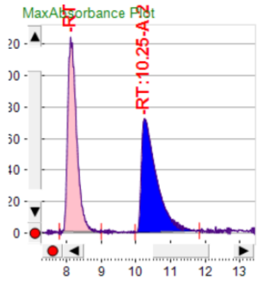

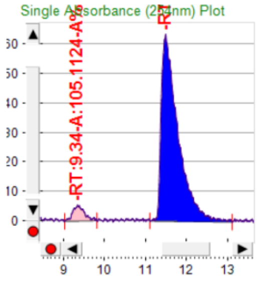

Peak #	Area %	Area	Ret. Time
1	50.0766	2183.7116	8.11 min
2	49.9234	2177.0322	10.25 min

Peak #	Area %	Area	Ret. Time
1	5.5263	105.1124	9.34 min
2	94.4737	1796.919	11.49 min

(*R*)–((2–methoxy–4–(4–(4–methoxyphenyl)butan–2–yl)–5–(4,4,5,5–tetramethyl–1,3,2–dioxaborolan–2–yl)phenyl)ethynyl)trimethylsilane (10af1):

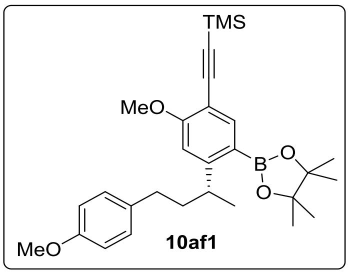

10af1

The starting boronic ester **9a** (52 mg, 0.18 mmol) was subjected to the conditions in General Procedure D. The crude product was purified by column chromatography (petroleum ether/EtOAc – 95/5) to afford the mixture of boron incorporated products (**10af1** and **10af2**) (53 mg, 60%). For characterization, the mixture was separated by preparative TLC after eluting 5 times with 98/2 EtOAc/Hexane. $[\alpha]_D^{23}$ = +26.14 (*c* 0.88, $CHCl_3$); R_f (95/5 petroleum ether/EtOAc): 0.72; IR (film) ν_{max}/cm^{-1}: 2958, 2927, 1511, 1332, 1245, 1141, 858, 841, 759; ^{1}H NMR (500 MHz, $CDCl_3$) δ 7.89 (s, 1H), 7.03 (d, *J* = 8.6 Hz, 2H), 6.85 – 6.77 (m, 3H), 3.91 (s, 3H), 3.79 (s, 3H), 3.72 – 3.63 (m, 1H), 2.55 (ddd, *J* = 13.8, 10.7, 6.3 Hz, 1H), 2.41 (ddd, *J* = 13.8, 10.6, 5.5 Hz, 1H), 1.92 – 1.76 (m, 2H), 1.33 (s, 6H), 1.33 (s, 6H), 1.27 (d, *J* = 6.8 Hz, 3H), 0.28 (s, 9H). ^{13}C NMR (126 MHz, $CDCl_3$) δ 162.38, 157.70, 157.58, 142.20, 134.93, 129.13, 113.69, 109.27, 107.28, 101.43, 97.46, 83.34, 55.65, 55.25, 40.81, 36.75, 33.12, 24.88, 24.79, 22.30, 0.18. ^{11}B NMR (96 MHz, $CDCl_3$) 30.10. HRMS (ESI): Mass calculated for [M+Na$^+$] $C_{29}H_{41}BNaO_4Si$ requires 515.2765 found 515.2764. HPLC Conditions: Chiracel IB with Guard Column, Hexane:IPA 99.5:0.5, flow rate – 1 mL/min, Injection vol. – 5 µL, t_R (minor) = 4.86 min, t_R (major) = 5.38 min. er 95.4:4.6. The minor impurity seen in the enantioenriched trace is of the other regioisomer which integrates to ~0.5%. The es was calculated to be 98.7% taking this into consideration.

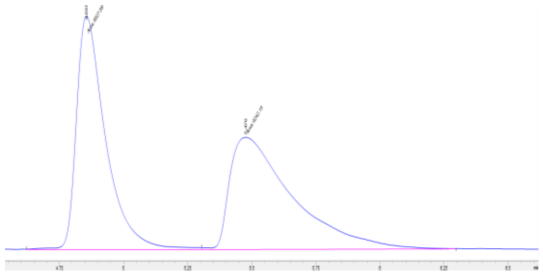

#	Time	Type	Area	Height	Width	Area%	Symmetry
1	4.855	MF	8927.9	1122.9	0.1325	49.135	0.512
2	5.476	FM	9242.2	539.1	0.2857	50.865	0.33

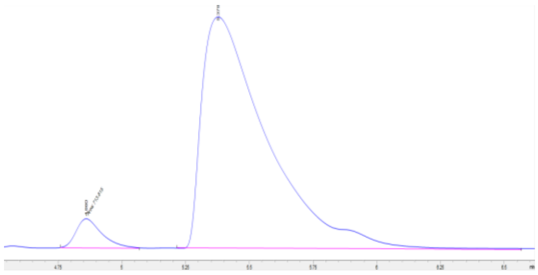

#	Time	Type	Area	Height	Width	Area%	Symmetry
1	4.86	MM	713.8	101	0.1178	4.751	0.623
2	5.378	MM R	14312.1	803	0.2783	95.249	0

(*R*)–((2–methoxy–4–(4–(4–methoxyphenyl)butan–2–yl)–3–(4,4,5,5–tetramethyl–1,3,2–dioxaborolan–2–yl)phenyl)ethynyl)trimethylsilane (10af2):

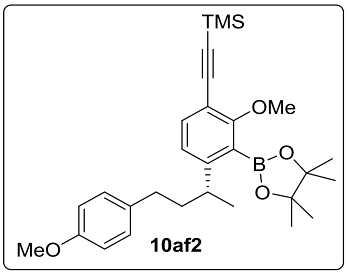

10af2

R_f (95/5 petroleum ether/EtOAc): 0.72; IR (film) ν_{max}/cm^{-1}: 2958, 2927, 1511, 1332, 1245, 1141, 858, 841, 759; ^{1}H NMR (600 MHz, $CDCl_3$) 7.40 – 7.35 (m, 1H), 7.03 (d, *J* = 8.3 Hz, 2H), 6.91 (d, *J* = 8.0 Hz, 1H), 6.79 (d, *J* = 8.3 Hz, 2H), 3.97 (s, 3H), 3.77 (s, 3H), 2.70 (sx,

J = 6.9 Hz, 1H), 2.52 (ddd, *J* = 14.1, 10.7, 5.7 Hz, 1H), 2.44 – 2.37 (m, 1H), 1.93 (dddd, *J* = 13.2, 10.8, 7.4, 5.9 Hz, 1H), 1.79 (ddt, *J* = 13.2, 10.8, 6.2 Hz, 1H), 1.34 (s, 6H), 1.33 (s, 6H), 1.24 (d, *J* = 6.9 Hz, 3H), 0.23 (s, 9H). ^{13}C NMR (151 MHz, $CDCl_3$) 167.05, 160.24, 156.04, 137.71, 137.26, 131.86, 123.02, 116.33, 115.38, 104.44, 100.94, 86.66, 64.06, 57.89, 42.39, 42.26, 35.72, 27.51, 27.41, 24.96, 2.58. ^{11}B NMR (96 MHz, $CDCl_3$) 30.18. HRMS (ESI): Mass calculated for [M+Na$^+$] $C_{29}H_{41}BNaO_4Si$ requires 515.2765 found 515.2760. HPLC Conditions: Chiracel IB with Guard Column, Hexane:IPA 99.9:0.1, flow rate – 1 mL/min, Injection vol. – 5 μL, t_R (minor) = 10.32 min, t_R (major) = 14.08 min. er 95.7:4.7

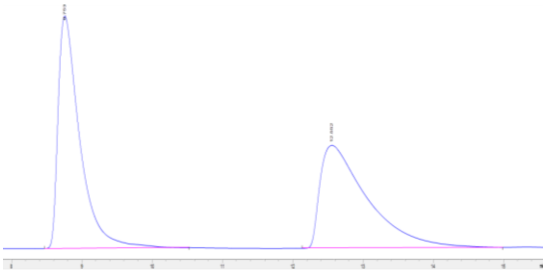

#	Time	Type	Area	Height	Width	Area%	Symmetry
1	8.753	BB	3021.4	140.5	0.3172	50.985	0.402
2	12.552	BB	2904.6	62.1	0.6326	49.015	0.307

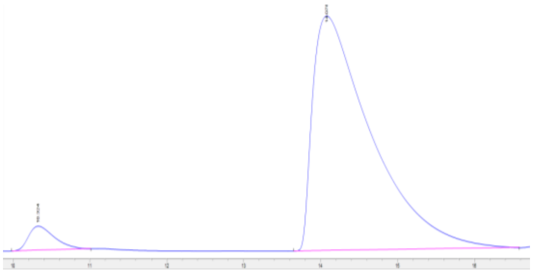

#	Time	Type	Area	Height	Width	Area%	Symmetry
1	10.324	BB	206.2	8.8	0.3175	4.331	0.558
2	14.078	BB	4555.5	85.6	0.7372	95.669	0.293

Trimethyl((2–(4,4,5,5–tetramethyl–1,3,2–dioxaborolan–2–yl)–[1,1'–biphenyl]–4–yl)ethynyl)silane (10b):

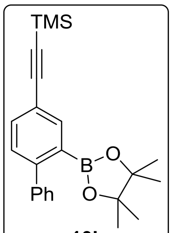

10b

The starting boronic ester, PhBpin **9b** (36 mg, 0.18 mmol) was subjected to the conditions in General Procedure D. The crude product was purified by column chromatography (petroleum ether/EtOAc – 98/2) to afford the title product **10b** (36 mg, 53%). R_f (98/2 petroleum ether/EtOAc): 0.40; IR (film) ν_{max}/cm^{-1}: 2977, 2925, 1342, 1143, 842, 759; ^{1}H NMR (500 MHz, $CDCl_3$) 7.84 (d, *J* = 1.6 Hz, 1H), 7.56 (dd, *J* = 8.0, 1.8 Hz, 1H), 7.42 – 7.32 (m, 6H), 1.23 (s, 12H), 0.28 (s, 9H). ^{13}C NMR (126 MHz, $CDCl_3$) 147.45, 142.46, 138.12, 133.37, 129.00, 128.88, 127.84, 127.17, 121.22, 105.07, 94.51, 83.94, 24.60, 0.03. ^{11}B NMR (96 MHz, $CDCl_3$) 30.20. HRMS (ESI) exact mass calculated for [M+Na$^+$] $C_{23}H_{29}BNaO_2Si$ requires 399.1931 found 339.1927.

***(R)*–trimethyl((4–(1–phenylhept–6–en–3–yl)–3–(4,4,5,5–tetramethyl–1,3,2–dioxaborolan–2–yl)phenyl)ethynyl)silane (10c):**

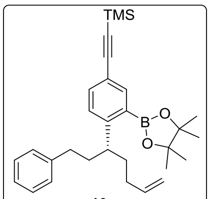

10c

The starting boronic ester **9c** (54 mg, 0.18 mmol) was subjected to the conditions in General Procedure D. The crude product was purified by column chromatography (petroleum ether/EtOAc – 95/5) to afford the title product **10c** (52 mg, 62%). $[\alpha]_D^{23}$ = +11.48 (*c* 1.05, $CHCl_3$); R_f (95/5 petroleum ether/EtOAc): 0.72; IR (film) ν_{max}/cm^{-1}: 2957, 2923, 2901, 1392, 1340, 1065, 842, 759; ^{1}H NMR (500 MHz, $CDCl_3$) 7.90 (dd, *J* = 1.9, 0.5 Hz, 1H), 7.52 (dd, *J* = 8.1, 1.9 Hz,

1H), 7.25 – 7.20 (m, 3H), 7.16 – 7.11 (m, 1H), 7.08 – 7.0.k8705 (m, 2H), 5.77 (ddt, *J* = 16.8, 10.2, 6.6 Hz, 1H), 4.93 – 4.84 (m, 2H), 3.57 (tt, *J* = 9.2, 5.3 Hz, 1H), 2.50 (ddd, *J* = 13.8, 11.1, 5.8 Hz, 1H), 2.34 (ddd, *J* = 13.8, 11.1, 5.0 Hz, 1H), 2.01 – 1.89 (m, 2H), 1.89 – 1.78 (m, 2H), 1.77 – 1.61 (m, 2H), 1.32 (s, 6H) 1.31 (s, 6H), 0.25 (s, 9H). ^{13}C NMR (126 MHz, $CDCl_3$) 152.74, 142.88, 139.32, 139.03, 134.36, 128.22, 128.19, 125.49, 125.45, 119.83, 114.05, 105.34, 93.39, 83.64, 41.78, 38.83, 36.40, 33.79, 31.62, 24.85, 0.04. ^{11}B NMR (96 MHz, $CDCl_3$) 30.35. HRMS (ESI) exact mass calculated for [M+Na$^+$] $C_{30}H_{41}BNaO_2Si$ requires 495.2867 found 495.2864. SFC Conditions: Chiracel IB Column, 5% [10% IPA:Hexane] /$CO_{2(scf)}$, flow rate – 2 mL/min, Pressure: 125 bar, Injection vol. – 10 μL, t_R (major) = 14.7 min, t_R (minor) = 15.5 min. er 96.3:3.7

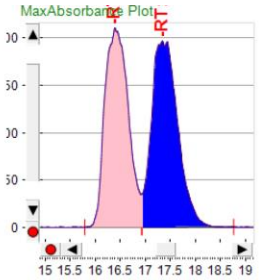

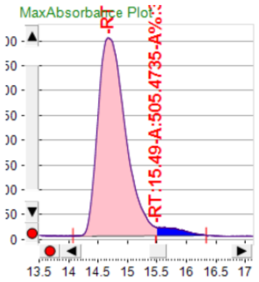

Peak #	Area %	Area	Ret. Time
1	48.8617	6890.4046	16.4 min
2	51.1383	7211.4445	17.35 min

Peak #	Area %	Area	Ret. Time
1	96.2918	13125.6829	14.67 min
2	3.7082	505.4735	15.49 min

***(S)*–((4–(1–cyclopropyl–3–phenylpropyl)–3–(4,4,5,5–tetramethyl–1,3,2–dioxaborolan–2–yl)phenyl)ethynyl)trimethylsilane (10d):** The starting boronic ester **9d** (52 mg, 0.18 mmol) was subjected to the conditions in General Procedure D. The crude product was purified by column chromatography (petroleum ether/EtOAc – 95/5) to afford the title product **10d** (60 mg, 73%). $[\alpha]_D^{23}$ = +99.04 (*c* 1.04, $CHCl_3$); R_f (95/5 petroleum ether/EtOAc): 0.72; IR (film) ν_{max}/cm^{-1}: 2976, 2926, 1340, 1063, 844; ^{1}H NMR (500 MHz, $CDCl_3$) 7.88 (d, *J* = 1.9 Hz, 1H), 7.52 (dd, *J* = 8.1, 1.9 Hz, 1H), 7.37 (d, *J* = 8.1 Hz, 1H), 7.27 – 7.20 (m, 2H), 7.17 – 7.06 (m, 3H), 2.81 (td, *J* = 8.9, 6.1 Hz, 1H), 2.60 (ddd, *J* = 13.9, 11.2, 5.7 Hz, 1H), 2.42 (ddd, *J* = 13.9, 11.1, 5.2 Hz, 1H), 2.14 – 1.94 (m, 2H), 1.30 (s, 6H), 1.29 (s, 6H), 0.96 (dtt, *J* = 10.1, 7.9, 5.1 Hz, 1H), 0.64 – 0.55 (m, 1H), 0.32 – 0.22 (m, 1H), 0.25 (s, 9H) 0.13 (m, 2H). ^{13}C NMR (126 MHz, $CDCl_3$) 153.22, 142.95, 139.20, 134.23, 128.21, 128.18, 126.02, 125.47, 119.82, 105.42, 93.29, 83.61, 47.14, 39.24, 33.94, 24.81, 24.78, 17.70, 6.03, 3.08, 0.05. ^{11}B NMR (96 MHz, $CDCl_3$) 30.04. HRMS (ESI) exact mass calculated for [M+Na$^+$] $C_{29}H_{39}BNaO_2Si$ requires 481.2710 found 481.2713. SFC Conditions: Whelk–01 Column, 5% [10% IPA:Hexane] /$CO_{2(scf)}$, flow rate

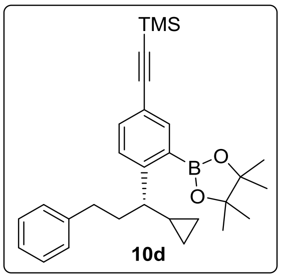

– 4 mL/min, Pressure: 125 bar, Injection vol. – 10 μL, t_R (minor) = 6.9 min, t_R (major) = 7.5 min. er 98.2:1.8.

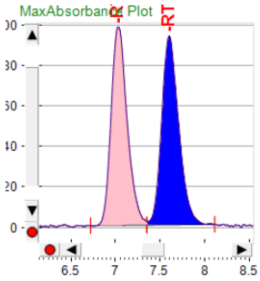

Peak #	Area %	Area	Ret. Time
1	49.8494	1220.0193	7.03 min
2	50.1506	1227.3919	7.6 min

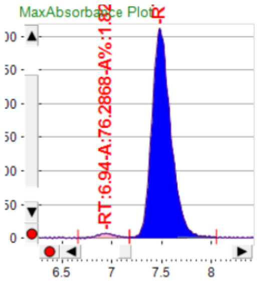

Peak #	Area %	Area	Ret. Time
1	1.828	76.2868	6.94 min
2	98.172	4096.8455	7.48 min

Tert–butyl–(*S*)–6–phenyl–4–(2–(4,4,5,5–tetramethyl–1,3,2–dioxaborolan–2–yl)–4–((trimethyl silyl)ethynyl)phenyl)hexanoate (10e):

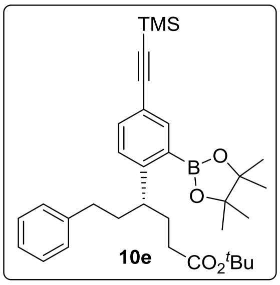

The starting boronic ester **9e** (67 mg, 0.18 mmol) was subjected to the conditions in General Procedure D. The crude product was purified by column chromatography (petroleum ether/EtOAc – 95/5) to afford the title product **10e** (64 mg, 81%). $[\alpha]_D^{23}$ = +11.96 (*c* 0.92, $CHCl_3$); R_f (95/5 petroleum ether/EtOAc): 0.72; IR (film) ν_{max}/cm^{-1}: 2976, 2930, 1728, 1339, 1143, 843; ^1H NMR (500 MHz, $CDCl_3$) 7.90 (d, *J* = 2.0 Hz, 1H), 7.52 (dd, *J* = 8.2, 2.0 Hz, 1H), 7.30 – 7.17 (m, 4H), 7.16 – 7.09 (m, 1H), 7.08 – 7.01 (m, 2H), 3.53 (tt, *J* = 9.9, 4.9 Hz, 1H), 2.50 (ddd, *J* = 13.7, 10.8, 5.9 Hz, 1H), 2.36 (ddd, *J* = 13.7, 10.8, 5.2 Hz, 1H), 2.16 – 2.08 (m, 1H), 2.05 – 1.76 (m, 6H), 1.38 (s, 9H), 1.32 (s, 6H), 1.31 (s, 6H), 0.24 (s, 9H). ^{13}C NMR (126 MHz, $CDCl_3$) 173.23, 151.86, 142.62, 139.45, 134.50, 128.21, 125.55, 125.46, 120.10, 105.19, 93.57, 83.72, 79.84, 41.73, 38.79, 33.73, 33.61, 31.96, 28.06, 24.84, 24.83, 0.02. ^{11}B NMR (96 MHz, $CDCl_3$) 30.66. HRMS (ESI) exact mass calculated for [M+Na$^+$] $C_{33}H_{47}BNaO_4Si$ requires 569.3235 found 569.3217. SFC Conditions: Chiracel IB Column, 5% [10% IPA:Hexane] /$CO_{2(scf)}$, flow rate – 4 mL/min, Pressure: 125 bar, Injection vol. – 10 μL, t_R (major) = 12.3 min, t_R (minor) = 13.8 min. er 96:4.

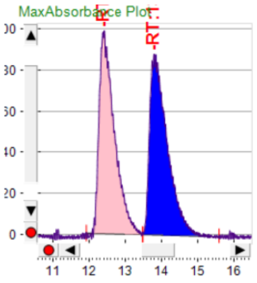

Peak #	Area %	Area	Ret. Time
1	49.3679	2874.6619	12.42 min
2	50.6321	2948.2728	13.79 min

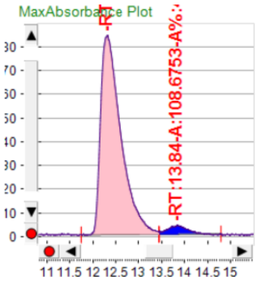

Peak #	Area %	Area	Ret. Time
1	95.9542	2577.4404	12.32 min
2	4.0458	108.6753	13.84 min

(*R*)–((4–(7–azido–1–phenylheptan–3–yl)–3–(4,4,5,5–tetramethyl–1,3,2–dioxaborolan–2–yl)phenyl)ethynyl)trimethylsilane (10f): The starting boronic ester **9f** (62 mg, 0.18 mmol) was subjected to the conditions in General Procedure D. The crude product was purified by column chromatography (petroleum ether/EtOAc – 95/5) to afford the title product **10f** (68 mg, 73%). $[\alpha]_D^{23}$ = +55.52 (*c* 0.95, $CHCl_3$); R_f (95/5 petroleum ether/EtOAc): 0.72; IR (film) ν_{max}/cm^{-1}: 2973, 2922, 2094, 1393, 1047, 844; ^1H NMR (500 MHz, $CDCl_3$) 7.90 (d, *J* = 1.7 Hz, 1H), 7.52 (dd, *J* = 8.1, 1.8 Hz, 1H), 7.22 (m, 3H), 7.13 (t, *J* = 7.3 Hz, 1H), 7.06 (d, *J* = 7.2 Hz, 2H), 3.55 (tt, *J* = 9.8, 5.4 Hz, 1H), 3.15 (tt, *J* = 12.2, 6.1 Hz, 2H), 2.48 (ddd, *J* = 13.7, 11.1, 5.9 Hz, 1H), 2.38 – 2.29 (m, 1H), 2.00 – 1.78 (m, 2H), 1.70 – 1.43 (m, 4H), 1.38 – 1.05 (m, 2H), 1.33 (s, 6H), 1.32 (s, 6H), 0.25 (s, 9H). ^{13}C NMR (126 MHz, $CDCl_3$) 152.68, 142.75, 139.40, 134.41, 128.21, 128.21, 125.53, 125.37, 119.91, 105.27, 93.48, 83.69, 51.35, 41.79, 38.84, 36.65, 33.80, 28.78, 24.86, 24.80, 24.41, 0.03. ^{11}B NMR (96 MHz, $CDCl_3$) 30.38. HRMS (ESI) exact mass calculated for [M+Na$^+$] $C_{30}H_{42}BN_3NaO_2Si$ requires 538.3037 found 538.3027. er 98.7:1.3

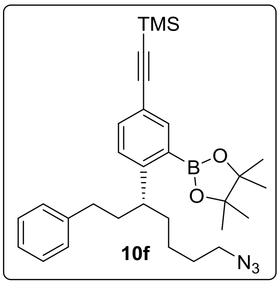

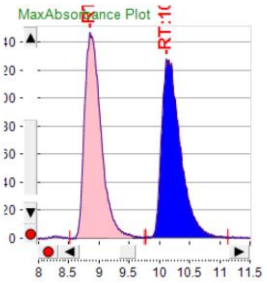

Peak #	Area %	Area	Ret. Time
1	50.6548	2789.5518	8.85 min
2	49.3452	2717.4344	10.11 min

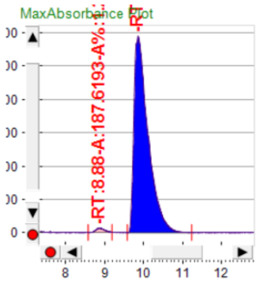

Peak #	Area %	Area	Ret. Time
1	1.2623	187.6193	8.88 min
2	98.7377	14675.4514	9.86 min

(*R*)–tert–butyldimethyl((9–phenyl–7–(2–(4,4,5,5–tetramethyl–1,3,2–dioxaborolan–2–yl)–4–((trimethylsilyl)ethynyl)phenyl)nonyl)oxy)silane (10g): The starting boronic ester (83 mg, 0.18 mmol) was subjected to the conditions in General Procedure D. The crude product was purified by column chromatography (petroleum ether/EtOAc – 98/2) to afford the title product (79 mg, 70%). $[\alpha]_D^{23}$ = +53.91 (*c* 1.28, $CHCl_3$); R_f (95/5 petroleum ether/EtOAc): 0.72; IR (film) ν_{max}/cm^{-1}: 2928, 2856, 1249, 1097, 838, 774; ^1H NMR (500 MHz, $CDCl_3$) 7.88 (d, *J* = 1.8 Hz, 1H), 7.50 (dd, *J* = 8.1, 1.8 Hz, 1H), 7.22 (t, *J* = 7.5 Hz, 3H), 7.12 (t, *J* = 7.5 Hz, 1H), 7.06 (d, *J* =

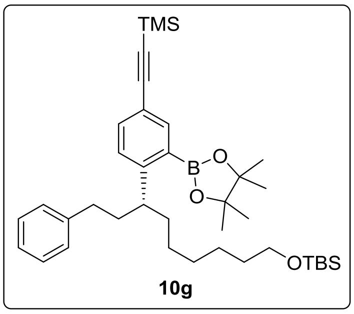

7.1 Hz, 2H), 3.59 – 3.48 (m, 3H), 2.48 (ddd, *J* = 13.8, 11.2, 5.7 Hz, 1H), 2.33 (ddd, *J* = 13.8, 11.3, 5.0 Hz, 1H), 1.93 (ddd, *J* = 16.6, 9.5, 5.6 Hz, 1H), 1.86 – 1.75 (m, 1H), 1.61 (td, *J* = 12.1, 10.7, 5.6 Hz, 4H), 1.49 – 1.38 (m, 2H), 1.36 – 1.13 (m, 16H), 0.88 (s, 9H), 0.24 (s, 9H), 0.02 (s, 6H). ^{13}C NMR (126 MHz, $CDCl_3$) 153.29, 143.00, 139.24, 134.28, 128.22, 128.16, 125.44, 125.44, 119.65, 105.43, 93.25, 83.59, 63.30, 42.08, 38.94, 37.15, 33.85, 32.84, 29.50, 27.31, 25.97, 25.71, 24.83, 18.35, 0.04, –5.28. ^{11}B NMR (96 MHz, $CDCl_3$) 30.53. HRMS (ESI) exact mass calculated for [M+Na$^+$] $C_{38}H_{61}BNaO_3Si_2$ requires 655.4152 found 655.4134. SFC Conditions: Chiracel IB Column, 5% [Hexane] /$CO_{2(scf)}$, flow rate – 4 mL/min, Pressure: 125 bar, Injection vol. – 10 μL, t_R (minor) = 26.5 min, t_R (major) = 27.3 min. er 96.3:3.7.

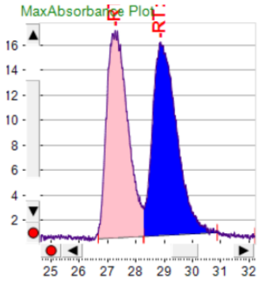

Peak #	Area %	Area	Ret. Time
1	48.3938	865.5853	27.27 min
2	51.6062	923.044	28.87 min

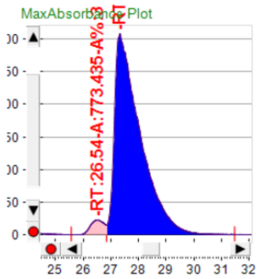

Peak #	Area %	Area	Ret. Time
1	3.7379	773.435	26.54 min
2	96.2621	19918.3164	27.34 min

***(S)*–6–phenyl–4–(2–(4,4,5,5–tetramethyl–1,3,2–dioxaborolan–2–yl)–4–((trimethylsilyl)ethynyl) phenyl)hexanenitrile (10h):** The starting boronic ester **9h** (54 mg, 0.18 mmol) was subjected to the conditions in General Procedure D. The crude product was purified by column chromatography (petroleum ether/EtOAc – 95/5) to afford the title product **10h** (57 mg, 67%). $[\alpha]_D^{23}$ = +21.42 (*c* 0.56, $CHCl_3$); R_f (95/5 petroleum ether/EtOAc): 0.72; IR (film) ν_{max}/cm^{-1}: 2957, 2929, 2245, 1249, 863, 849, 759; ^{1}H NMR (500 MHz, $CDCl_3$) 7.91 (d, *J* = 1.9 Hz, 1H), 7.54 (dd, *J* = 8.1, 1.9 Hz, 1H), 7.27 – 7.18 (m, 3H), 7.15 (d, *J* = 7.4 Hz, 1H), 7.09 – 7.01 (m, 2H), 3.62 (dt, *J* = 10.0, 5.1 Hz, 1H), 2.53 – 2.29 (m, 2H), 2.24 – 2.11 (m, 1H), 2.12 – 1.82 (m, 5H), 1.32 (s, 6H), 1.31 (s, 6H), 0.24 (s, 9H). ^{13}C NMR (126 MHz, $CDCl_3$) 149.86, 141.99, 139.69, 134.77, 128.32, 128.18, 125.76, 125.19, 120.86, 119.93, 104.76, 94.19, 84.02, 41.35, 38.35, 33.55, 32.94, 24.94, 24.74, 15.05, –0.02. ^{11}B NMR (96 MHz, $CDCl_3$) 29.82. HRMS (ESI) exact mass calculated for [M+Na$^+$] $C_{29}H_{38}BNNaO_2Si$ requires 494.2657 found 494.2651. SFC Conditions: Chiracel IB Column, 20% [10% IPA:Hexane] /$CO_{2(scf)}$, flow rate – 4

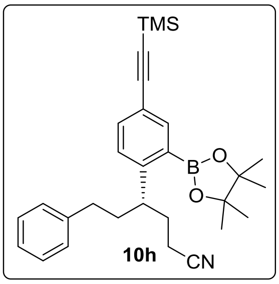

mL/min, Pressure: 125 bar, Injection vol. – 10 μL, t_R (minor) = 5.8 min, t_R (major) = 10.7 min. er 98:2.

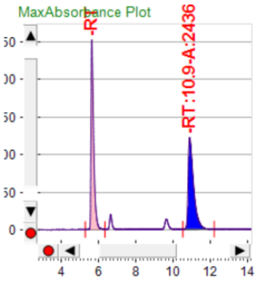

Peak #	Area %	Area	Ret. Time
1	49.7944	2416.8653	5.63 min
2	50.2056	2436.8253	10.9 min

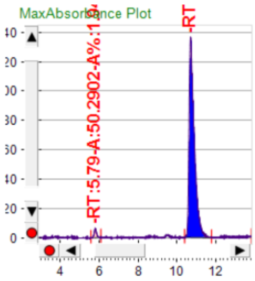

Peak #	Area %	Area	Ret. Time
1	1.9438	50.2902	5.79 min
2	98.0562	2536.8807	10.7 min

1-(3-(2-(4,4,5,5-tetramethyl-1,3,2-dioxaborolan-2-yl)-4-((trimethylsilyl)ethynyl)phenyl)-3,4-dihydroquinolin-1(2H)-yl)ethan-1-one (10i): The starting boronic ester **9i** (54 mg, 0.18 mmol) was subjected to the conditions in General Procedure D. The crude product was subjected to oxidation in THF (1 mL) with 2 M NaOH (1 mL) and 30% aq. H_2O_2 (1 mL) at 0 °C and stirred for 1 h. The reaction mixture was then warmed to rt and stirred for additional 1 h. After the complete consumption of the starting material (monitored by TLC), the reaction mixture was acidified with 1 M HCl (2 mL) was extracted with DCM (2x10 mL). The organic layer was separated and dried over anhydrous Na_2SO_4 and concentrated under vacuum. The crude product was purified by flash column chromatography on silica gel eluting with Toluene/EtOAc – 90/10 to afford the title product **10i** (34 mg, 52%). $[\alpha]_D^{23}$ = +21.42 (*c* 0.56, $CHCl_3$); R_f (85/15 petroleum ether/EtOAc): 0.4; IR (film) ν_{max}/cm^{-1}: 3189, 2957, 2922, 2852, 1628, 1409, 1248, 840, 758; ^1H NMR (500 MHz, $CDCl_3$) δ 7.22 (dq, *J* = 21.9, 6.7 Hz, 4H), 7.04 – 6.93 (m, 3H), 4.10 (bs, 1H), 3.89 (bs, 1H), 3.56 (bs, 1H), 3.19 – 3.00 (m, 2H), 2.22 (s, 3H), 0.26 (s, 9H). ^{13}C NMR (126 MHz, $CDCl_3$) δ 170.69, 153.59, 139.03, 132.48, 129.75, 128.85, 127.23, 126.09, 125.37, 124.57, 124.47, 122.57, 118.77, 104.87, 93.93, 48.15, 35.32, 32.51, 22.84, -0.09. HRMS (ESI) exact mass calculated for $[M+Na^+]$ $C_{22}H_{25}NNaO_2Si$ requires 386.1551 found 386.1547. SFC Conditions: Chiracel IB Column, 20% [50% IPA:Hexane] /$CO_{2(scf)}$, flow rate – 2 mL/min, Pressure: 100 bar, Injection vol. – 10 μL, t_R (minor) = 15.91 min, t_R (major) = 17.37 min. er 99.4:0.6.

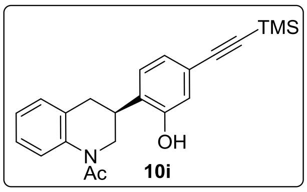

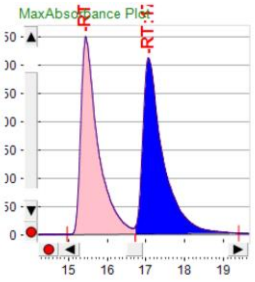

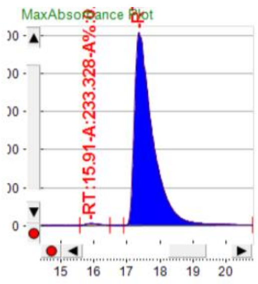

Peak #	Area %	Area	Ret. Time
1	49.186	10769.254	15.44 min
2	50.814	11125.6945	17.06 min

Peak #	Area %	Area	Ret. Time
1	0.6032	233.328	15.91 min
2	99.3968	38450.7625	17.37 min

((4–((3r,5r,7r)–adamantan–1–yl)–3–(4,4,5,5–tetramethyl–1,3,2–dioxaborolan–2–yl)phenyl)ethynyl)trimethylsilane (10j): The starting boronic ester **9j** (54 mg, 0.18 mmol) was subjected to the conditions in General Procedure D. The crude product was purified by column chromatography (petroleum ether/EtOAc – 95/5) to afford the title product **10j** (57 mg, 67%). R_f (95/5 petroleum ether/EtOAc): 0.6; IR (film) ν_{max}/cm^{-1}: 2957, 2928, 1151, 842, 799; ^{1}H NMR (400 MHz, $CDCl_3$) 7.52 (d, *J* = 1.9 Hz, 1H), 7.40 (dd, *J* = 8.3, 2.0 Hz, 1H), 7.30 (d, *J* = 8.3 Hz, 1H), 2.09 (s, 3H), 2.01 (m, 6H), 1.76 (m, 7H), 1.39 (s, 12H), 0.23 (s, 9H). ^{13}C NMR (126 MHz, $CDCl_3$) 155.50, 137.32, 132.70, 124.84, 119.41, 105.50, 93.30, 83.99, 42.78, 37.90, 36.66, 29.09, 24.89, 0.08. ^{11}B NMR (96 MHz, $CDCl_3$) 32.37. HRMS (ESI) exact mass calculated for [M+Na$^+$] $C_{27}H_{39}BNaO_2Si$ requires 457.2710 found 457.2706.

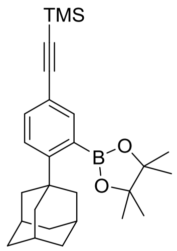

10j

((4–((1R,2S,5R)–2–isopropyl–5–methylcyclohexyl)–3–(4,4,5,5–tetramethyl–1,3,2–dioxaborolan–2–yl)phenyl)ethynyl)trimethylsilane (10k): The starting boronic ester **9k** (48 mg, 0.18 mmol) was subjected to the conditions in General Procedure D. The crude product was purified by column chromatography (petroleum ether/EtOAc – 95/5) to afford the title product **10k** (69 mg, 88%). $[\alpha]_D^{23}$ = –27.88 (*c* 1.04, $CHCl_3$); R_f (95/5 petroleum ether/EtOAc): 0.72; IR (film) ν_{max}/cm^{-1}: 2954, 2926, 1341, 1143, 842; ^{1}H NMR (500 MHz, $CDCl_3$) 7.82 (d, *J* = 2.0 Hz, 1H), 7.45 (dd, *J* = 8.2, 2.0 Hz, 1H), 7.18 (d, *J* = 8.2 Hz, 1H), 3.32 (td, *J* = 11.6, 3.4 Hz, 1H), 1.83 – 1.69 (m, 3H), 1.62 – 1.33 (m, 16H), 1.05 – 0.81 (m, 8H), 0.77 (d, *J* = 7.0 Hz, 3H), 0.61 (d, *J* = 6.9 Hz, 3H), 0.22 (s, 9H). ^{13}C NMR (126 MHz, $CDCl_3$) 153.91, 139.21, 133.97, 125.73, 119.33, 105.53, 93.01, 83.52, 46.48, 46.19, 44.85, 35.39, 33.29, 27.46, 24.89, 24.77, 24.62, 22.57, 21.55, 15.51, 0.04. ^{11}B NMR (96 MHz, $CDCl_3$) 30.38. HRMS (ESI) exact mass calculated for [M+Na$^+$] $C_{27}H_{43}BNaO_2Si$ requires 461.3023 found 461.3008.

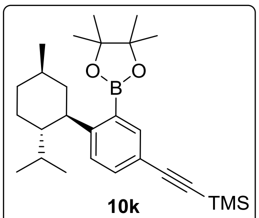

10k

***O*–TBS–6–(2–(4,4,5,5–tetramethyl–1,3,2–dioxaborolan–2–yl)–4–((trimethylsilyl)ethynyl) phenyl)–5–hydrocholesterol (10l):** The starting boronic ester **9l** (95 mg, 0.18 mmol) was subjected to the conditions in General Procedure D. The crude product was purified by column chromatography (petroleum ether/EtOAc – 95/5) to afford the title product **10l** (93 mg, 76%). $[\alpha]_D^{23}$ = +13.75 (*c* 0.8, $CHCl_3$); R_f (95/5 petroleum ether/EtOAc): 0.72; IR (film) ν_{max}/cm^{-1}: 2936, 2854, 1340, 1097, 854, 837; ^{1}H NMR (500 MHz, $CDCl_3$) 7.83 (d, *J* = 1.8 Hz, 1H), 7.44 (dd, *J* = 8.2, 2.0 Hz, 1H), 7.13 (d, *J* = 8.2 Hz, 1H), 3.47 (td, *J* = 11.8, 3.6 Hz, 1H), 3.38 (tt, *J* = 10.4, 4.8 Hz, 1H), 2.00 (dd, *J* = 12.7, 3.4 Hz, 1H), 1.86 – 1.61 (m, 5H), 1.59 – 1.41 (m, 7H), 1.33 (m, 16H), 1.21 – 1.05 (m, 7H), 1.04 – 0.82 (m, 17H), 0.77 (s, 9H), 0.68 (s, 3H), 0.23 (d, *J* = 0.7 Hz, 9H), –0.12 (d, *J* = 8.3 Hz, 6H). ^{13}C NMR (126 MHz, $CDCl_3$) 153.50, 139.26, 133.98, 125.30, 119.49, 105.58, 92.94, 83.54, 72.50, 56.36, 56.24, 54.52, 49.39, 42.58, 41.92, 40.75, 40.11, 39.49, 37.50, 36.24, 36.15, 35.80, 35.52, 34.60, 31.77, 28.25, 27.98, 25.91, 24.92, 24.88, 24.16, 23.81, 22.79, 22.54, 21.34, 18.67, 18.23, 13.18, 12.05, 0.06, –4.54, –4.82. ^{11}B NMR (96 MHz, $CDCl_3$) 33.26. HRMS (ESI) exact mass calculated for $[M+Na^+]$ $C_{50}H_{85}BNaO_3Si_2$ requires 823.6032, found 823.6031.

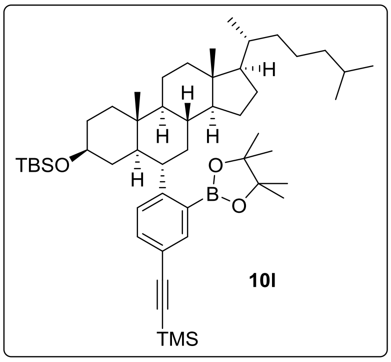

10l

2–(5–ethynyl–2–(4–(4–methoxyphenyl)butan–2–yl)phenyl)–4,4,5,5–tetramethyl–1,3,2–dioxaborolane (11a):

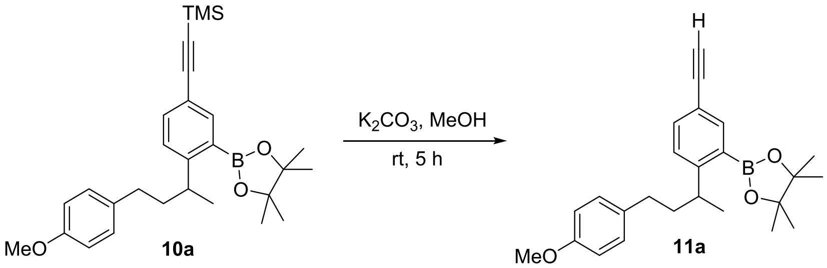

To a solution of starting boronic ester **10a** (20 mg, 0.044 mmol) in MeOH (0.5 mL) solid K_2CO_3 (0.132 mmol, 3 equiv) was added and stirred at rt for 5 h. After the complete consumption of the starting material (monitored by TLC), the reaction mixture was concentrated under vacuum and redissolved in DCM and filtered through a short silica pad. The filtrate was concentrated under vacuum and purified by flash column chromatography on silica gel eluting with petroleum ether/EtOAc – 95/5 to afford the title product **11a** (15 mg, 87%). R_f (95/5 petroleum ether/EtOAc): 0.5; IR (film) ν_{max}/cm^{-1}: 3403, 2961,

1511, 1412, 1246, 847, 759; ^{1}H NMR (500 MHz, $CDCl_3$) 7.89 (d, *J* = 1.7 Hz, 1H), 7.51 (dd, *J* = 8.0, 1.7 Hz, 1H), 7.26 (d, *J* = 8.0 Hz, 1H), 7.02 (d, *J* = 8.6 Hz, 2H), 6.78 (d, *J* = 8.6 Hz, 2H), 3.76 (s, 3H), 3.56 (sx, *J* = 7.1 Hz, 1H), 3.01 (s, 1H), 2.52 (ddd, *J* = 13.9, 10.6, 5.9 Hz, 1H), 2.41 (tt, *J* = 10.8, 5.5 Hz, 1H), 1.96 – 1.74 (m, 2H), 1.31 (s, 6H), 1.30 (s, 6H), 1.24 (d, *J* = 6.9 Hz, 3H). ^{13}C NMR (126 MHz, $CDCl_3$) 157.55, 154.88, 139.49, 134.83, 134.35, 129.11, 125.14, 118.69, 113.65, 83.87, 83.65, 76.37, 55.22, 40.48, 36.69, 33.07, 24.84, 24.76, 22.30. ^{11}B NMR (96 MHz, $CDCl_3$) 30.45. HRMS (ESI) exact mass calculated for [M+H$^+$] $C_{25}H_{32}BO_3$ requires 391.2444, found 391.2452.

1–(4–(4–(4–methoxyphenyl)butan–2–yl)–3–(4,4,5,5–tetramethyl–1,3,2–dioxaborolan–2–yl)phen yl)ethan–1–one (11b):

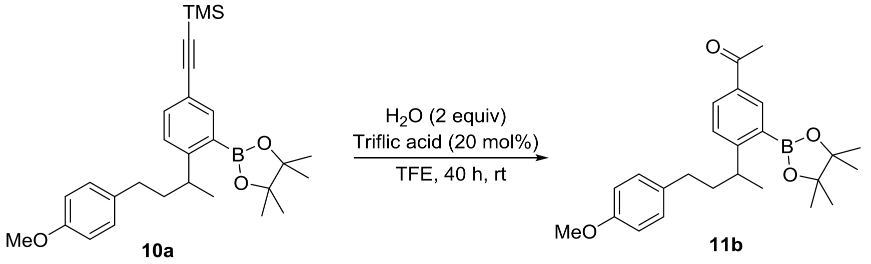

To a solution of starting boronic ester **10a** (20 mg, 0.044 mmol) in TFE (0.5 mL) deionised water (8 μL, 0.088 mmol, 2 equiv) and triflic acid (2 mg; 0.009 mmol, 20 mol%) were added and stirred at rt for 40 h. The color of the reaction turned pale red immediately after the addition of triflic acid. After the complete consumption of the starting material (monitored by TLC), the reaction mixture e was quenched with aqueous $NaHCO_3$ and extracted with DCM (2x10 mL). The organic layer was separated and dried over anhydrous Na_2SO_4 and concentrated under vacuum. The crude product was purified by flash column chromatography on silica gel eluting with petroleum ether/EtOAc – 90/10 to afford the title product **11b** (13.5 mg, 76%). R_f (95/5 petroleum ether/EtOAc): 0.72; IR (film) ν_{max}/cm^{-1}: 2971, 2928, 1683, 1512, 1245, 846; ^{1}H NMR (500 MHz, $CDCl_3$) 8.31 (d, *J* = 2.0 Hz, 1H), 7.99 (dd, *J* = 8.2, 2.0 Hz, 1H), 7.40 (d, *J* = 8.2 Hz, 1H), 7.02 (d, *J* = 8.6 Hz, 2H), 6.78 (d, *J* = 8.6 Hz, 2H), 3.76 (s, 3H), 3.63 (sx, *J* = 7.0 Hz, 1H), 2.61 (s, 3H), 2.53 (ddd, *J* = 13.8, 10.6, 6.0 Hz, 1H), 2.46 – 2.36 (m, 1H), 1.98 – 1.78 (m, 2H), 1.33 (s, 6H), 1.32 (s, 6H), 1.27 (d, *J* = 6.9 Hz, 3H). ^{13}C NMR (126 MHz, $CDCl_3$) 198.11, 159.84, 157.59, 136.06, 134.67, 134.12, 130.62, 129.10, 125.44, 113.67, 83.79, 55.22, 40.36, 36.92, 33.07, 26.61, 24.86, 24.79, 22.24. ^{11}B NMR (96 MHz, $CDCl_3$) 31.95. HRMS (ESI) exact mass calculated for [M+Na$^+$] $C_{25}H_{33}BNaO_4$ requires 431.2369, found 431.2368.

1–benzyl–4–(4–(4–methoxyphenyl)butan–2–yl)–3–(4,4,5,5–tetramethyl–1,3,2–dioxaborolan–2–yl)phenyl)–1H–1,2,3–triazole (11c):

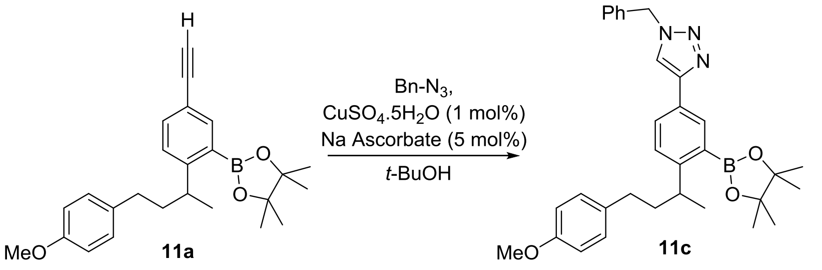

To a solution of starting boronic ester **11a** (20 mg, 0.051 mmol) in *t*-BuOH (0.5 mL) benzyl azide (6.8 mg, 0.051 mmol), $CuSO_4$. $5H_2O$ (0.2mg, 0.005 mmol, 0.01 equiv) and sodium ascorbate (5 mg, 0.025 mmol, 0.05 equiv) were added and stirred at rt for 18 h. After the complete consumption of the starting material (monitored by TLC), the reaction mixture was concentrated under vacuum and redissolved in DCM and filtered through a short silica pad. The filtrate was concentrated under vacuum and purified by flash column chromatography on silica gel eluting with petroleum ether/EtOAc – 85/15 to afford the title product **11c** (23 mg, 85%). R_f (85/15 petroleum ether/EtOAc): 0.3; IR (film) ν_{max}/cm^{-1}: 2959, 2931, 2835, 1511, 1243, 1162, 1036, 829; ^1H NMR (500 MHz, $CDCl_3$) 8.06 (d, *J* = 2.1 Hz, 1H), 7.94 (dd, *J* = 8.2, 2.1 Hz, 1H), 7.70 (s, 1H), 7.37 (m, 4H), 7.29 (dd, *J* = 7.5, 1.7 Hz, 2H), 7.07 – 6.99 (m, 2H), 6.83 – 6.73 (m, 2H), 5.57 (s, 2H), 3.76 (s, 3H), 3.64 – 3.52 (m, 1H), 2.53 (ddd, *J* = 13.9, 10.7, 6.0 Hz, 1H), 2.42 (ddd, *J* = 13.9, 10.7, 5.4 Hz, 1H), 1.96 – 1.77 (m, 2H), 1.29 (m, 15H). ^{13}C NMR (126 MHz, $CDCl_3$) 157.51, 154.26, 148.31, 135.01, 134.87, 132.87, 129.13, 129.09, 128.66, 128.35, 127.92, 127.22, 125.64, 119.34, 113.63, 83.53, 55.21, 54.14, 40.65, 36.51, 33.13, 24.87, 24.80, 22.47. ^{11}B NMR (96 MHz, $CDCl_3$) 30.83. HRMS (ESI) exact mass calculated for [M+Na$^+$] $C_{32}H_{38}BN_3NaO_3$ requires 546.2904, found 546.2889.

2–(4–(4–methoxyphenyl)butan–2–yl)–5–((trimethylsilyl)ethynyl)phenol (11d):

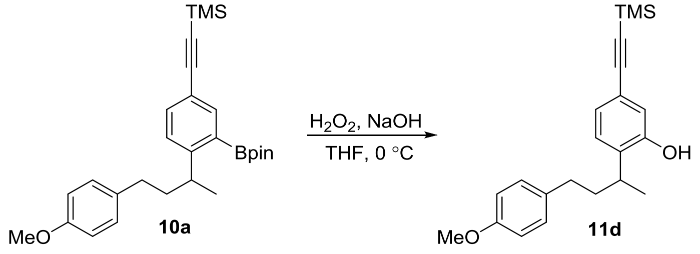

To a solution of starting boronic ester **10a** (25 mg, 0.054 mmol) in THF (0.5 mL) at 0 °C was added 2 M NaOH (0.5 mL) and 30% aq. H_2O_2 (0.5 mL) and stirred 1 h. The reaction

mixture was then warmed to rt and stirred for additional 1 h. After the complete consumption of the starting material (monitored by TLC), the reaction mixture was acidified with 1 M HCl (2 mL) was extracted with DCM (2x10 mL). The organic layer was separated and dried over anhydrous Na_2SO_4 and concentrated under vacuum. The crude product was purified by flash column chromatography on silica gel eluting with petroleum ether/EtOAc – 90/10 to afford the title product **11d** (17 mg, 90%). R_f (95/5 petroleum ether/EtOAc): 0.3; IR (film) ν_{max}/cm^{-1}: 3403, 2961, 1511, 1412, 1246, 847, 759; ^{1}H NMR (500 MHz, $CDCl_3$) 7.07 (m, 4H), 6.86 (d, *J* = 1.4 Hz, 1H), 6.80 (d, *J* = 8.6 Hz, 2H), 4.60 (s, 1H), 3.78 (s, 3H), 3.03 (sx, *J* = 6.8 Hz, 1H), 2.48 (m, 2H), 1.98 – 1.78 (m, 2H), 1.24 (d, *J* = 6.9 Hz, 3H), 0.23 (s, 9H). ^{13}C NMR (126 MHz, $CDCl_3$) 157.67, 152.58, 134.37, 134.15, 129.18, 127.17, 124.97, 121.26, 118.58, 113.73, 104.74, 93.52, 55.24, 38.75, 32.79, 31.79, 20.77, –0.03. HRMS (ESI) exact mass calculated for [M+H$^+$] $C_{19}H_{22}NO$ requires 280.1696, found 280.1708.

5–ethynyl–2–(4–(4–methoxyphenyl)butan–2–yl)aniline (11e):

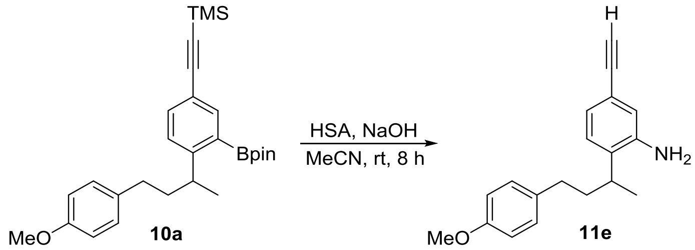

To a solution of starting boronic ester **10a** (23 mg, 0.05 mmol) in MeCN (0.5 mL) at rt was added hydroxylamine sulfonic acid (8.2 mg, 0.0725 mmol, 1.5 equiv.) and 1 M aq. NaOH (0.5 mL) and stirred for 8 h. After the complete consumption of the starting material (monitored by TLC), the reaction mixture was extracted with DCM (2x10 mL). The organic layer was separated and dried over anhydrous Na_2SO_4 and concentrated under vacuum. The crude product was purified by flash column chromatography on silica gel eluting with petroleum ether/EtOAc – 80/20 to afford the title product **11e** (9 mg, 62%). R_f (95/5 petroleum ether/EtOAc): 0.3; IR (film) ν_{max}/cm^{-1}: 3283, 2958, 2928, 1617, 1511, 1244, 1177, 817; ^{1}H NMR (500 MHz, $CDCl_3$) 7.11 – 7.02 (m, 3H), 6.92 (dd, *J* = 7.9, 1.6 Hz, 1H), 6.82 (d, *J* = 8.7 Hz, 2H), 6.79 (d, *J* = 1.6 Hz, 1H), 3.78 (s, 3H), 3.49 (m, 2H), 2.97 (s, 1H), 2.67 (sx, *J* = 7.0 Hz, 1H), 2.61 – 2.48 (m, 2H), 1.93 (ddt, *J* = 13.8, 8.5, 6.9 Hz, 1H), 1.81 (ddt, *J* = 13.8, 8.3, 7.0 Hz, 1H), 1.25 (d, *J* = 6.8 Hz, 3H). ^{13}C NMR (126 MHz, $CDCl_3$) 157.77, 143.48,

134.06, 132.50, 129.28, 126.13, 122.92, 119.85, 119.13, 113.78, 83.98, 75.88, 55.24, 38.41, 32.61, 31.70, 20.26. HRMS (ESI) exact mass calculated for [M+Na$^+$] $C_{22}H_{28}BNaO_2Si$ requires 375.1751, found 375.1753.

2-(2-(4-(4-methoxyphenyl)butan-2-yl)-5-(phenylethynyl)phenyl)-4,4,5,5-tetramethyl-1,3,2-dioxaborolane (11f)

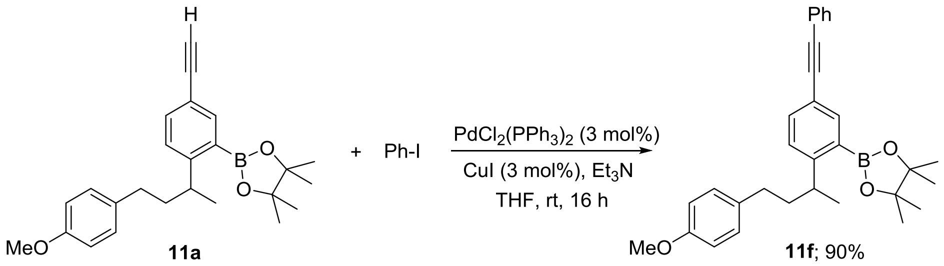

To a solution of starting boronic ester **11a** (40 mg, 0.1 mmol) in THF (1 mL) at rt was added Ph-I (31 mg, 0.15 mmol, 1.5 equiv), $PdCl_2(PPh_3)_2$ (2 mg; 0.003 mol, 3 mol%) and CuI (0.6 mg, 0.003 mol, 3 mol%) and stirred. To the reaction mixture triethyl amine (0.080 mL, 0.5 mmol, 5 equiv) was added slowly and the reaction mixture was allowed to stir over-night. After the complete consumption of the starting material (monitored by TLC), the reaction mixture was diluted with ether and filtered through a short pad of silica. The filtrate was concentrated in vacuo and the crude product was purified by flash column chromatography on silica gel eluting with petroleum ether/EtOAc – 95/5 to afford the title product **11f** (43 mg, 90%). R_f (95/5 petroleum ether/EtOAc): 0.6; IR (film) ν_{max}/cm^{-1}: 2975, 2931, 1511, 1342, 1138, 855, 755; ^{1}H NMR (500 MHz, $CDCl_3$) δ 7.96 (d, *J* = 1.8 Hz, 1H), 7.57 (dd, *J* = 8.1, 1.9 Hz, 1H), 7.55 – 7.51 (m, 2H), 7.39 – 7.29 (m, 4H), 7.04 (d, *J* = 8.6 Hz, 2H), 6.80 (d, *J* = 8.7 Hz, 2H), 3.78 (s, 3H), 3.67 – 3.55 (m, 1H), 2.61 – 2.39 (m, 2H), 1.98 – 1.77 (m, 2H), 1.34 (d, *J* = 5.0 Hz, 12H), 1.28 (d, *J* = 6.9 Hz, 3H). ^{13}C NMR (126 MHz, $CDCl_3$) δ 157.56, 154.40, 139.01, 134.90, 133.88, 131.54, 129.14, 128.27, 127.96, 125.22, 123.61, 119.89, 113.66, 89.65, 88.75, 83.65, 55.22, 40.57, 36.66, 33.12, 24.87, 24.80, 22.35. ^{11}B NMR (96 MHz, $CDCl_3$) 31.42. HRMS (ESI) exact mass calculated for [M+Na$^+$] $C_{31}H_{35}BNaO_3Si$ requires 489.2575, found 489.2577.

Attempts to Detect the Bromoallene Intermediate

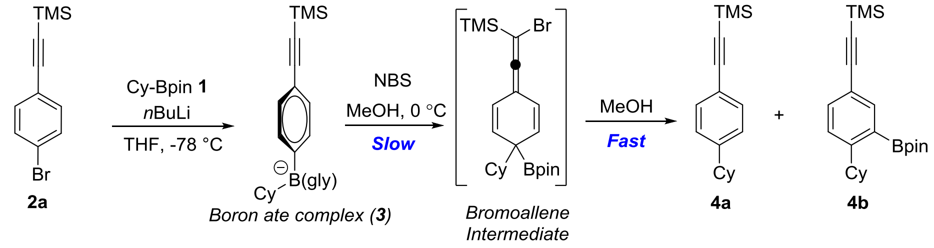

In situ IR Studies of the Reaction

IR Background correction was done at 0 °C with a well stirred mixture of THF (2.5 mL) + MeOH (2 mL) under inert atmosphere.

The General Procedure has been modified to observe weakly absorbing functional groups and for IR probe compatibility. A solution of p–bromophenylacetylene **2a** (330 mg; 1.32 mmol, 1.0 equiv) in THF (2.5 mL, 0.53 M) in a three-neck flask was cooled to –78 °C and treated with n–BuLi (0.825 mL, 1.0 equiv, 1.6 M in hexanes) and the mixture was stirred at this temperature for 1 h, the reaction turned viscous. The boronic ester **1** (305 mg, 1.45 mmol, 1.1 eq.) was added neat dropwise. The mixture was stirred at –78 °C for 1 h as it solidified and the stirring stopped. The mixture was then brought to rt for complete ate-complex formation (clear solution) and then cooled to 0 °C with an ice bath. At this point, MeOH (2 mL) was added. The IR probe was dipped into the solution to record a reference spectrum of the ate-complex. After this, continuous IR scanning was turned on at 15 seconds interval. Then, NBS (solid) (188 mg, 1.06 mmol; 0.8 equiv) was added in one-go. The IR spectrum was collected in set 15 min intervals. The reaction was worked up and the product formation was confirmed by GC-MS (see below).

Instrument Information:

ReactIR 15, Probe A; ReactIR 15 with MCT Detector using HappGenzel apodization; SiComp (Silicon) probe connected via AgX 9.5mm x 1.5m Fiber (Silver Halide); Sampling 3000 to 650 at 8 wavenumber resolution; Scan option: AutoSelect; Gain: 1x

1. Trends

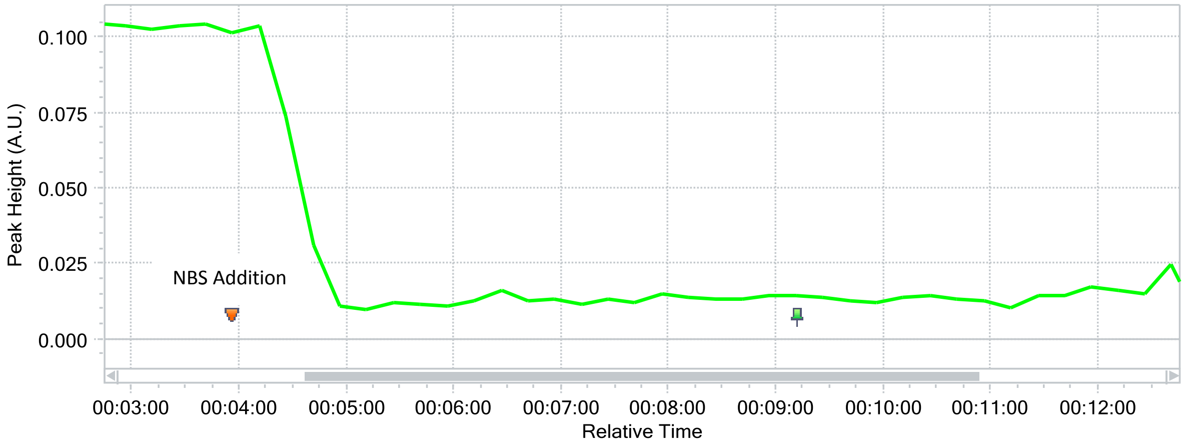

2. Peaks

Peak Profile	Description
Peak at 2152 cm-1	Height to Zero, Peak at 2152
Peak at 2160 cm-1	Height to Zero, Peak at 2160

3. Spectra

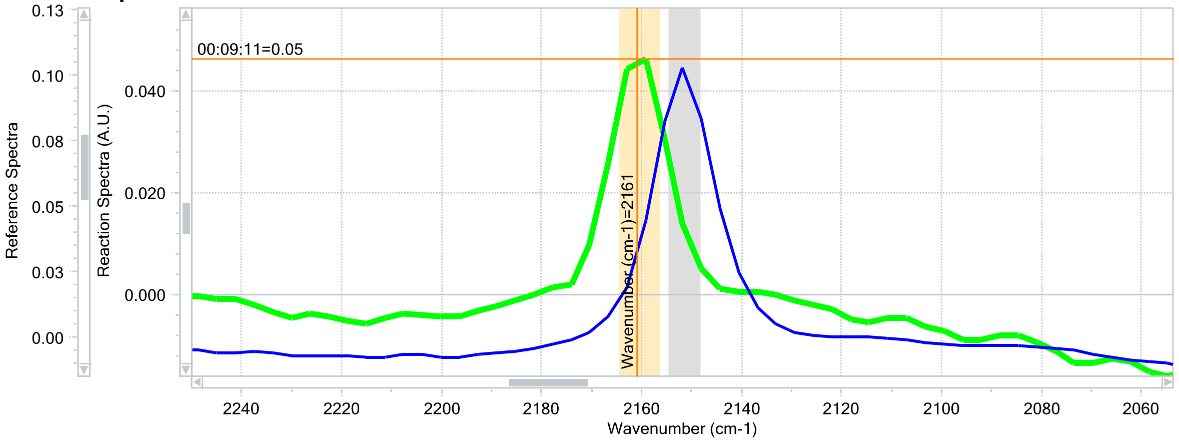

4. Surface

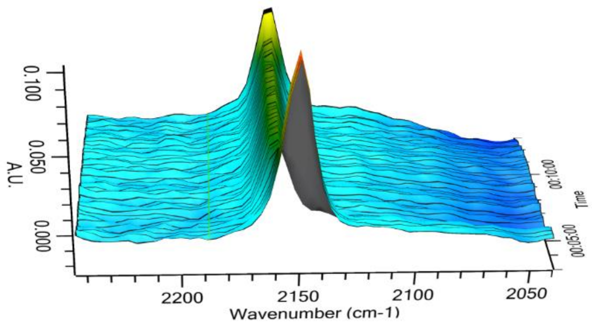

Discussion

1 This peak at 2152 cm^{-1} represents the boronate complex. After addition of NBS, this peak disappears over 1 minute.

2&3 Peak at 2152 cm^{-1} is the alkyne stretch of the boronate complex and the peak at 2160 cm^{-1} is the alkyne strecth of the product.

4 3-D Visualisation of the frequency against time plot. This shows rapid decline of the starting boronate complex at 2152 cm^{-1} (same as picture 1 – "trends") with growth of the product at 2160 cm^{-1} after the addition of NBS. The reaction is essentially instantaneous at 0 °C. Spectra 3 shows that the peaks overlap and so there is no distinct demarcation between events. No allene intermediate at lower wavenumber was observed. This implies that the initial dearomatisation is slow and rearomatisation is fast.

GC-MS Trace of the reaction monitored by ReactIR

File : C:\DATA\GANESH\VGD-94.D
Operator : ganesh
Acquired : 3 Jun 2017 16:47 using AcqMethod 70-1X
Instrument : Instrumen
Sample Name: cy-bpn thf/meoh 0.3M
Misc Info :
Vial Number: 50

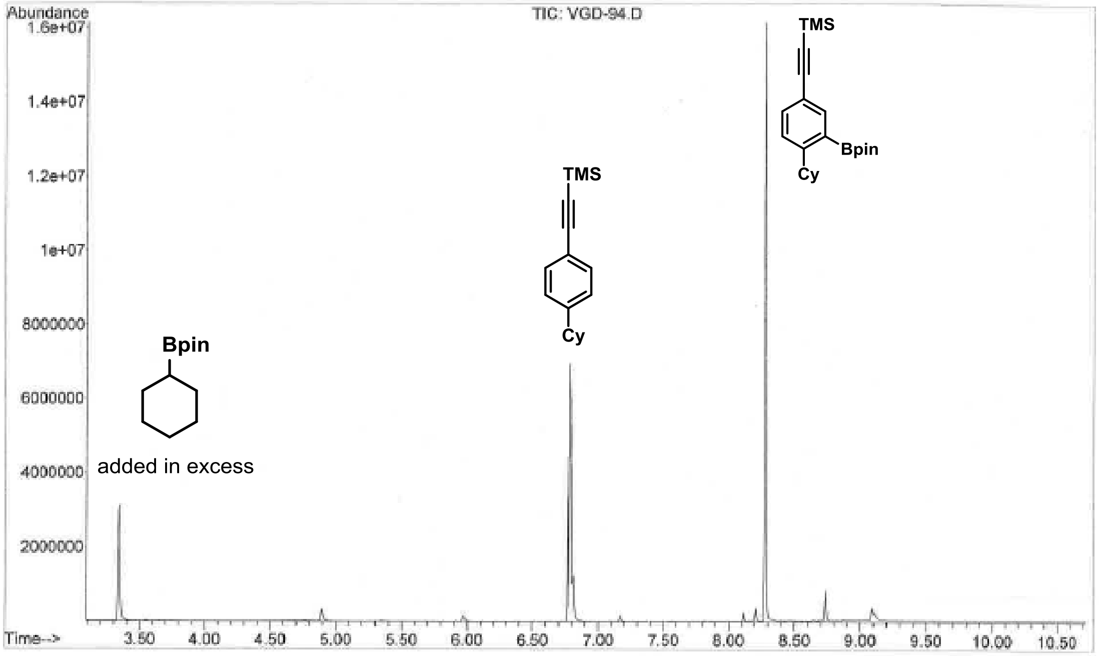

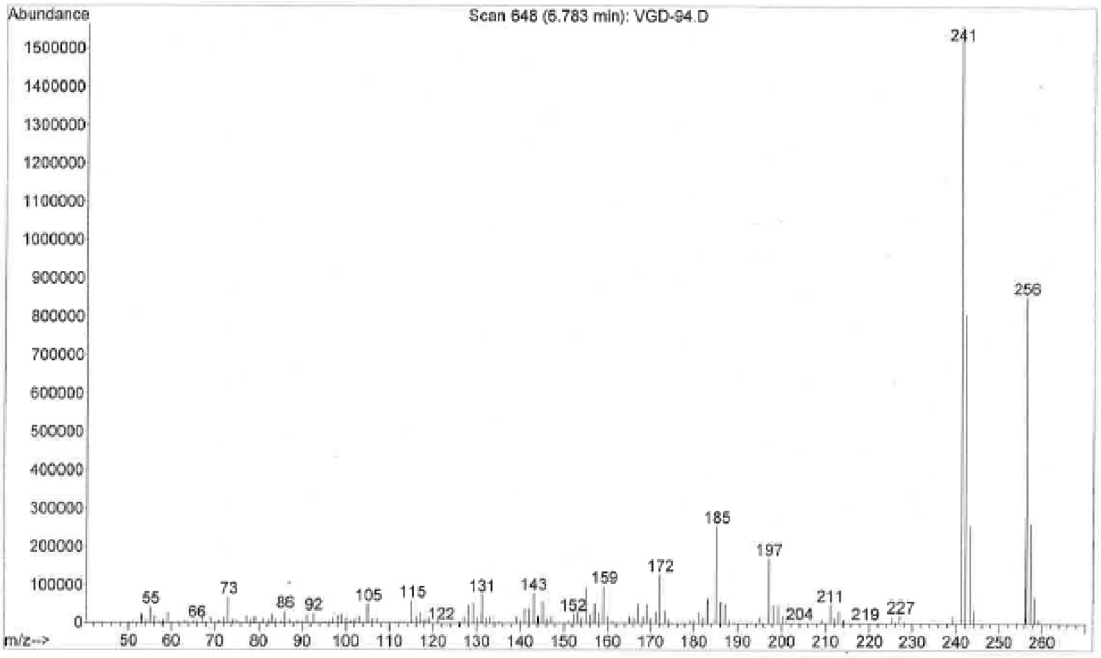

NMR Traces

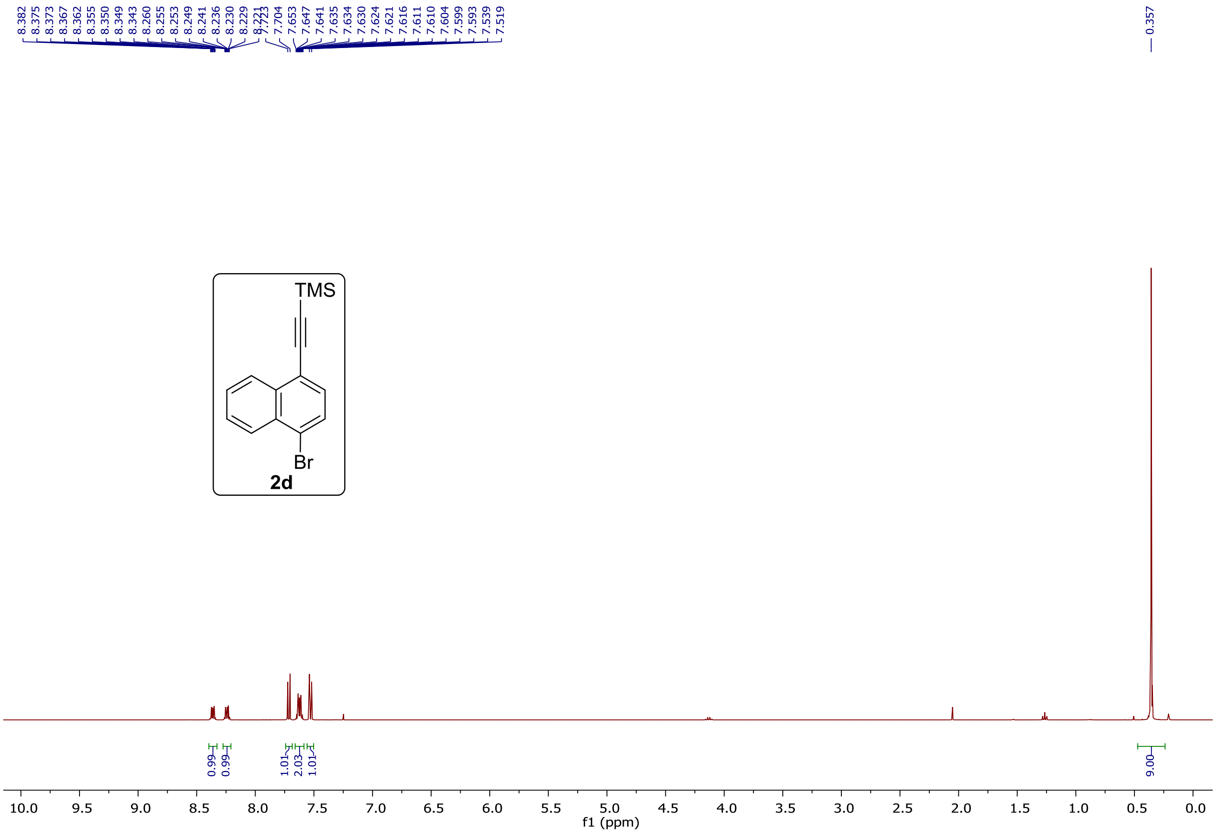

8.382
8.375
8.373
8.367
8.362
8.355
8.350
8.349
8.343
8.260
8.255
8.253
8.249
8.241
8.236
8.230
8.229
8.221
7.723
7.704
7.653
7.647
7.641
7.635
7.634
7.630
7.624
7.621
7.616
7.611
7.610
7.604
7.599
7.593
7.539
7.519
0.357
TMS
Br
2d
0.99
0.99
1.01
2.03
1.01
9.00
f1 (ppm)

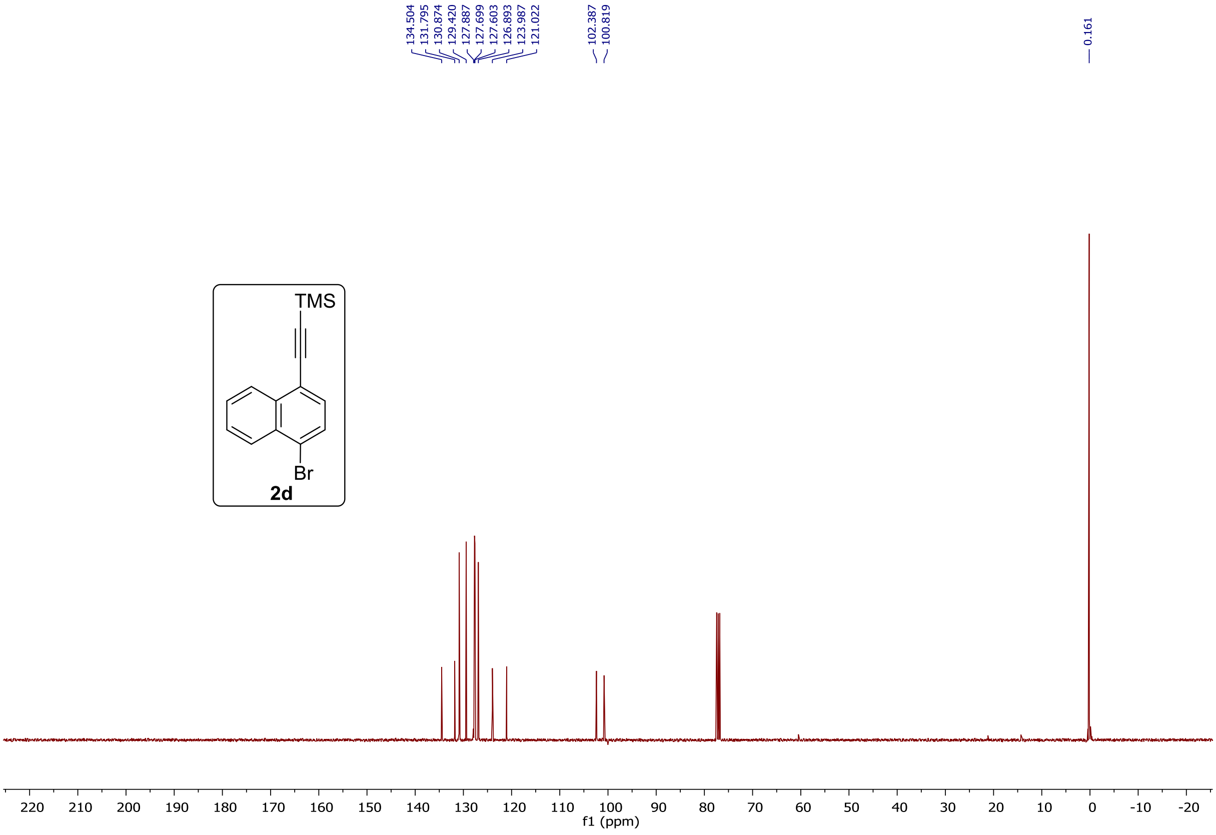

134.504
131.795
130.874
129.420
127.887
127.699
127.603
126.893
123.987
121.022
102.387
100.819
0.161
TMS
Br
2d
f1 (ppm)

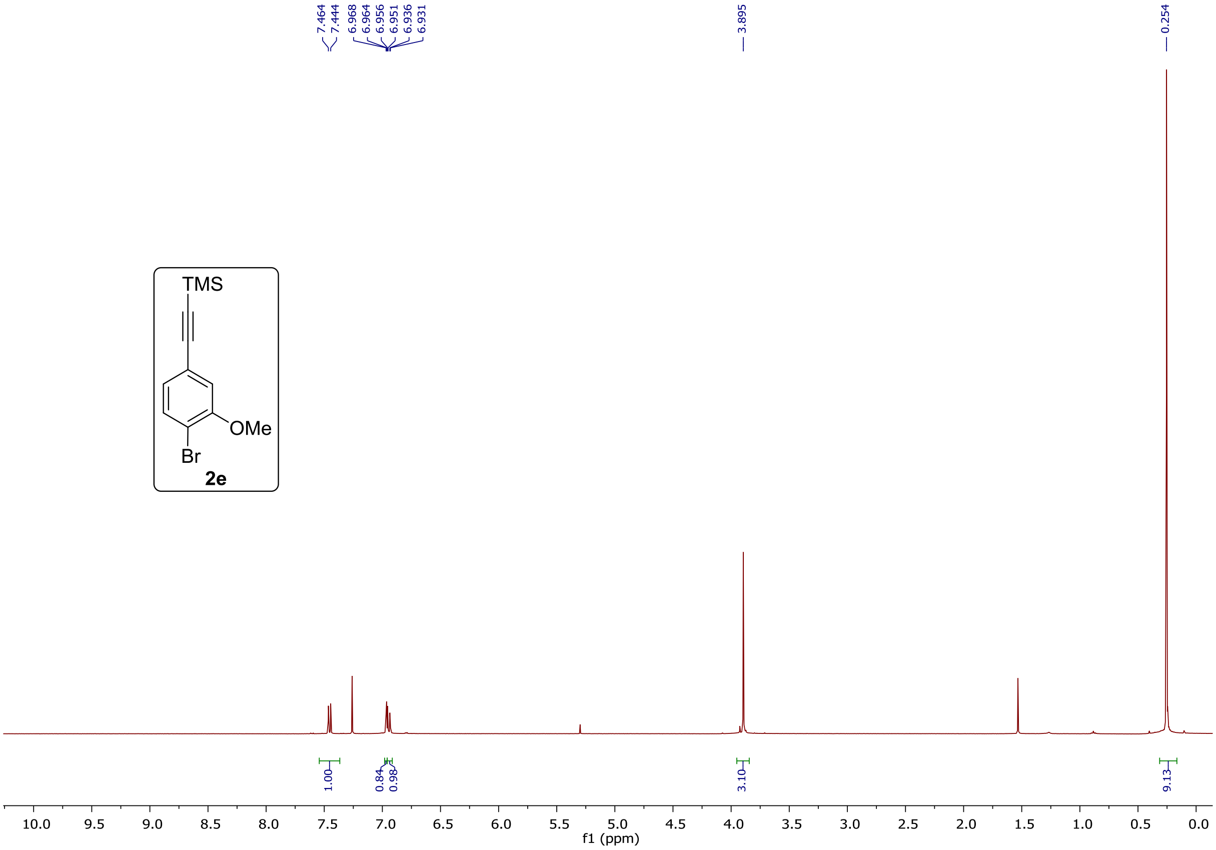
7.464
7.444
6.968
6.964
6.956
6.951
6.936
6.931
3.895
0.254
TMS
OMe
Br
2e
1.00
0.84
0.98
3.10
9.13
10.0 9.5 9.0 8.5 8.0 7.5 7.0 6.5 6.0 5.5 5.0 4.5 4.0 3.5 3.0 2.5 2.0 1.5 1.0 0.5 0.0
f1 (ppm)

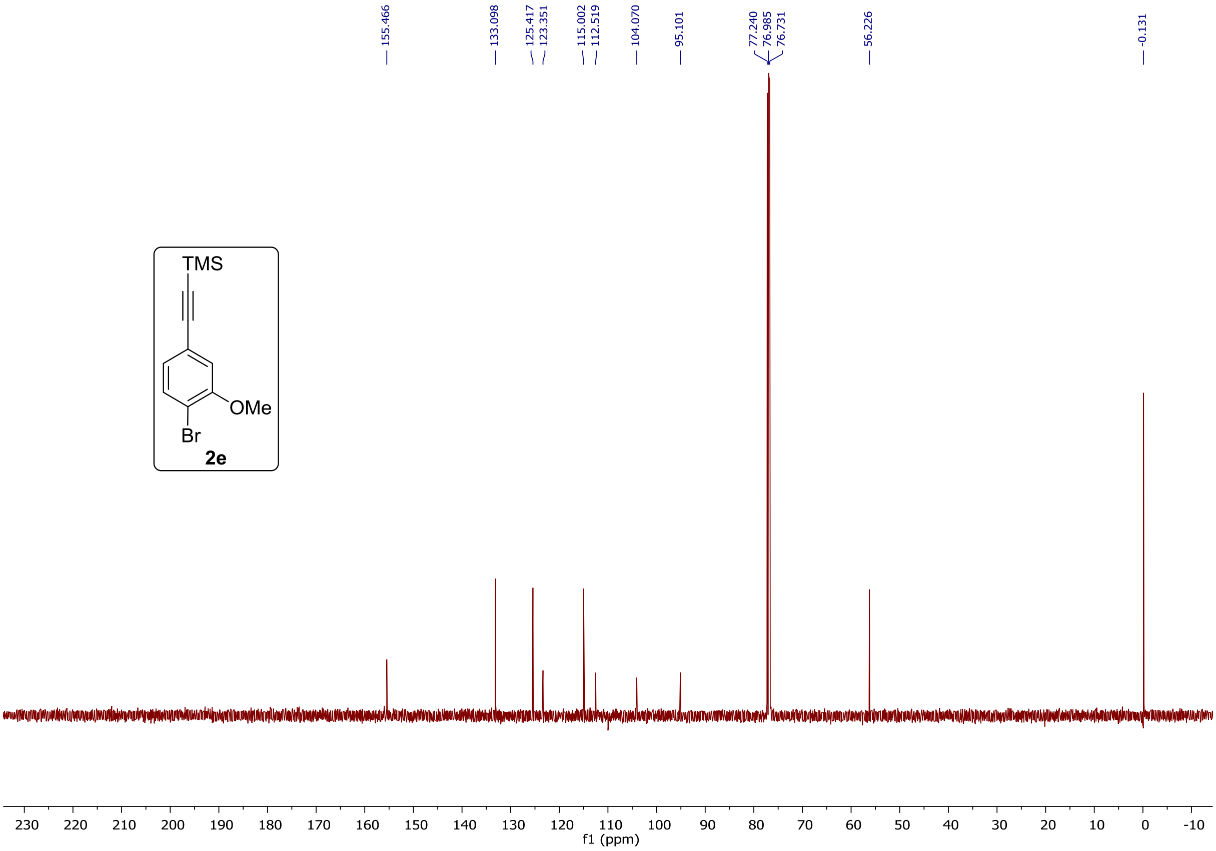
155.466
133.098
125.417
123.351
115.002
112.519
104.070
95.101
77.240
76.985
76.731
56.226
-0.131
TMS
OMe
Br
2e
230 220 210 200 190 180 170 160 150 140 130 120 110 100 90 80 70 60 50 40 30 20 10 0 -10
f1 (ppm)

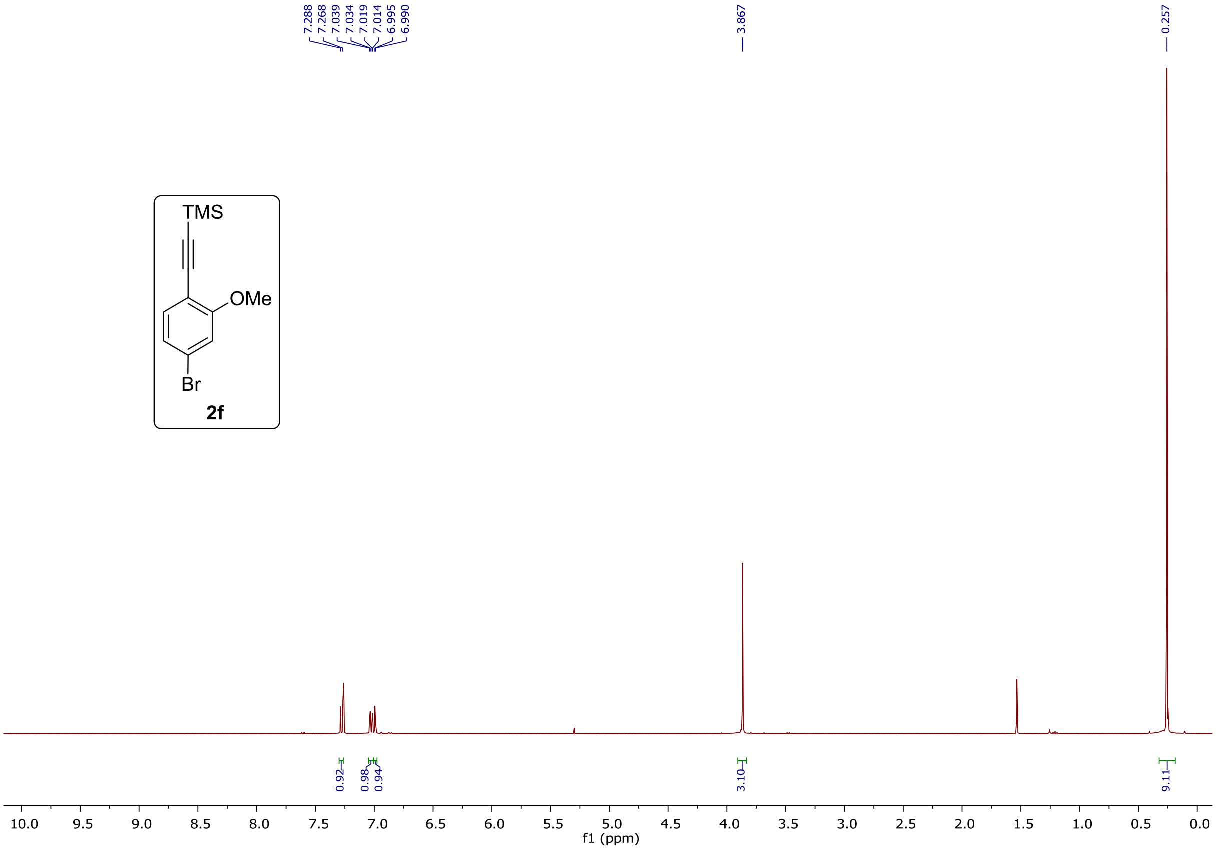
7.288
7.268
7.039
7.034
7.019
7.014
6.995
6.990
3.867
0.257
TMS
OMe
Br
2f
0.92
0.98
0.94
3.10
9.11
10.0 9.5 9.0 8.5 8.0 7.5 7.0 6.5 6.0 5.5 5.0 4.5 4.0 3.5 3.0 2.5 2.0 1.5 1.0 0.5 0.0
f1 (ppm)

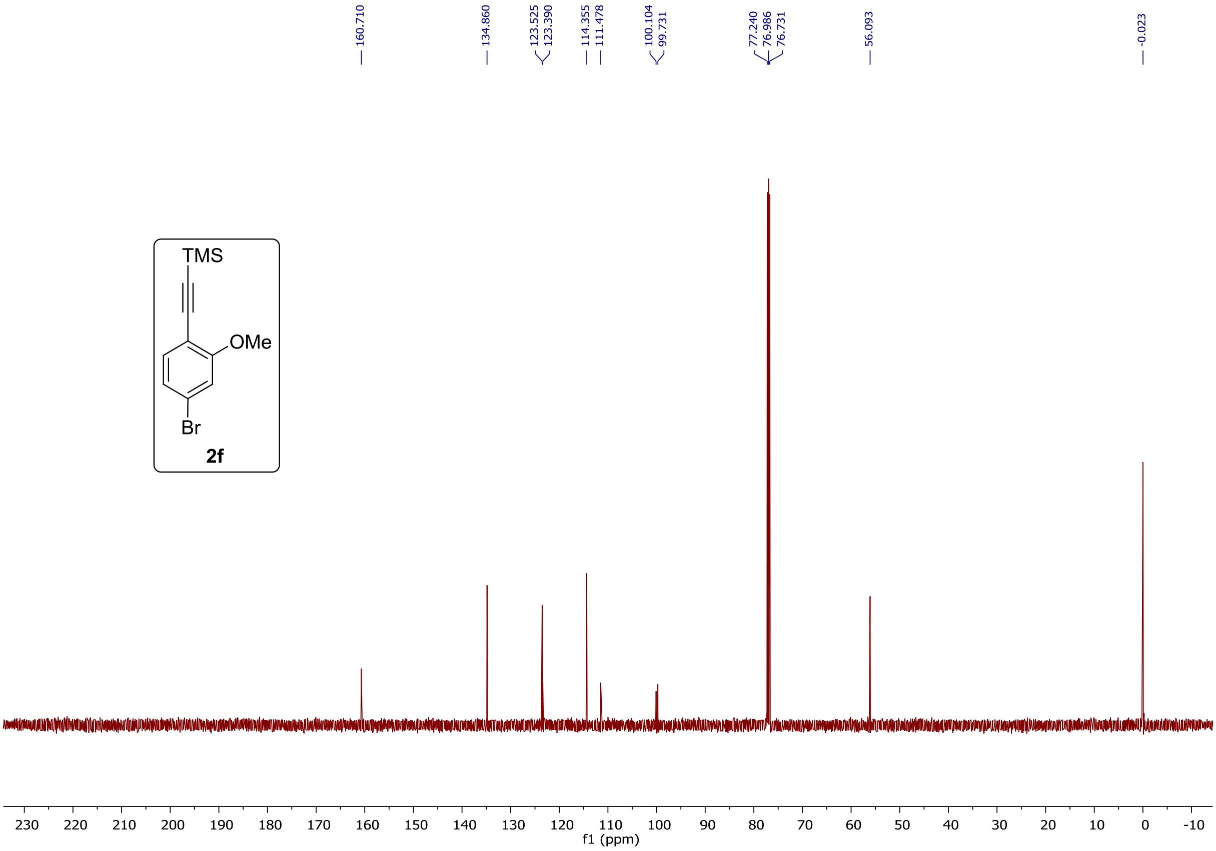
160.710
134.860
123.525
123.390
114.355
111.478
100.104
99.731
77.240
76.986
76.731
56.093
-0.023
TMS
OMe
Br
2f
230 220 210 200 190 180 170 160 150 140 130 120 110 100 90 80 70 60 50 40 30 20 10 0 -10
f1 (ppm)

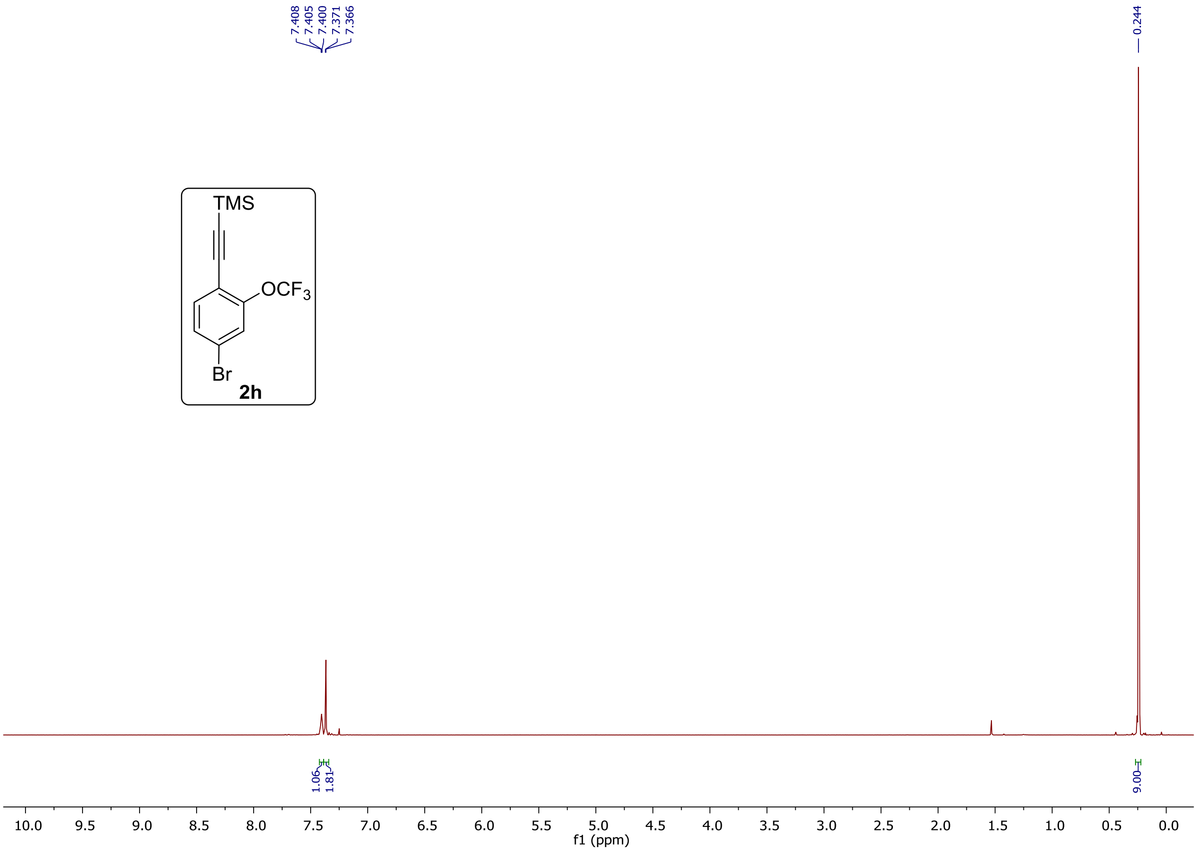
7.408
7.405
7.400
7.371
7.366
0.244
TMS
OCF3
Br
2h
1.06
1.81
9.00
f1 (ppm)

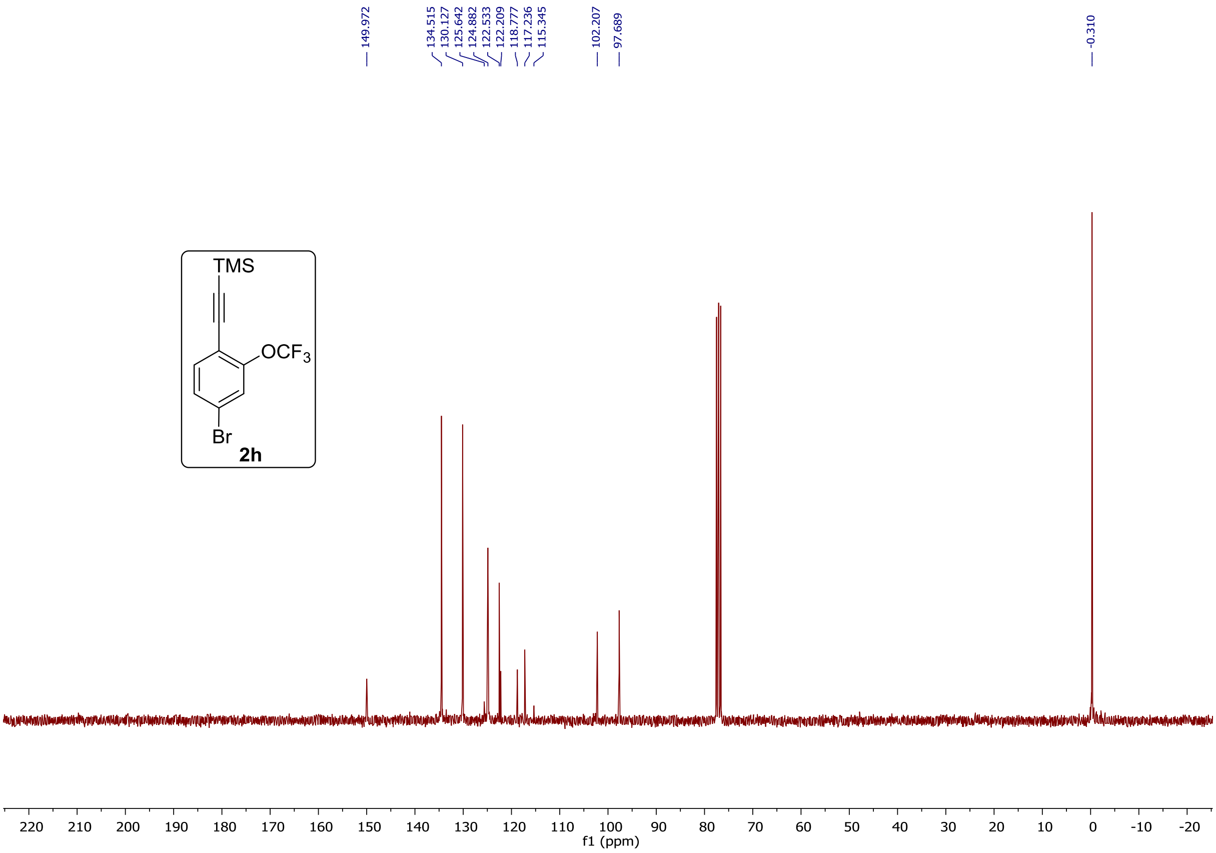
149.972
134.515
130.127
125.642
124.882
122.533
122.209
118.777
117.236
115.345
102.207
97.689
-0.310
TMS
OCF3
Br
2h
f1 (ppm)

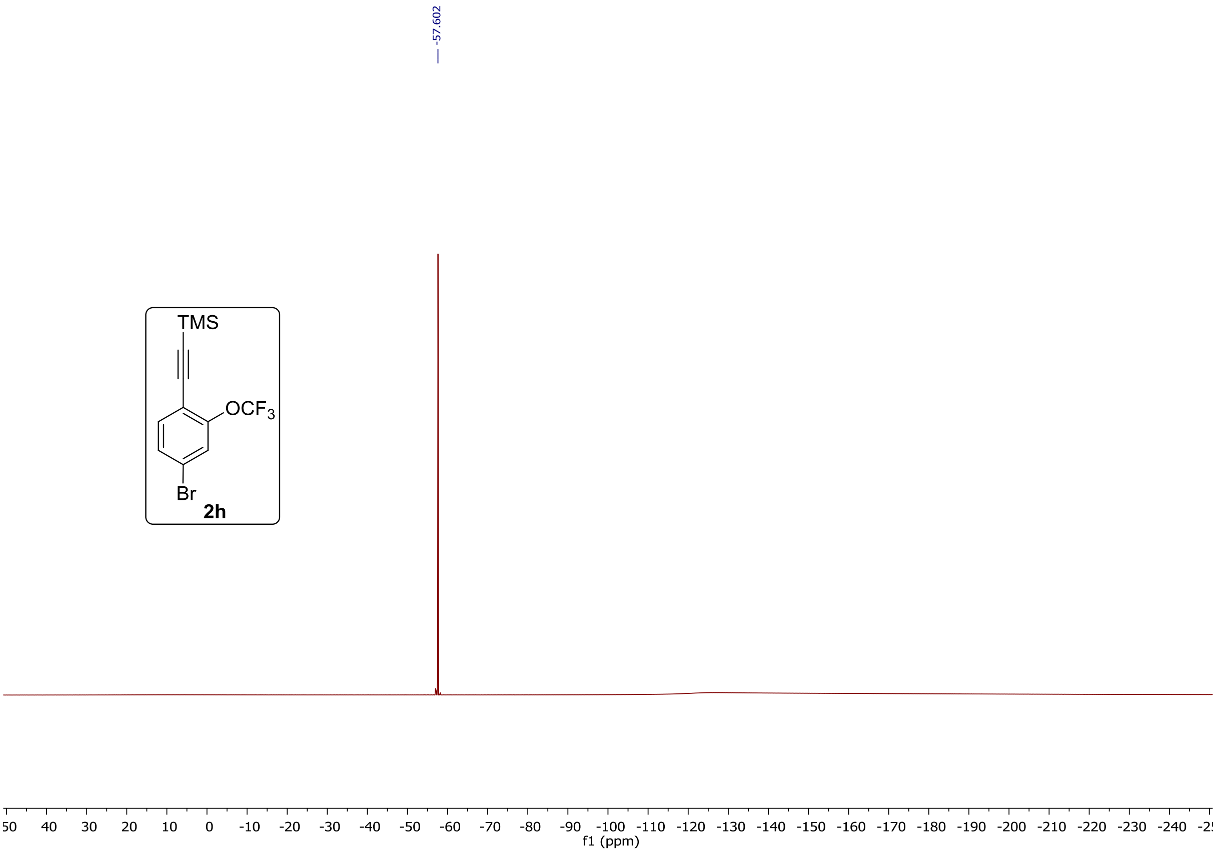
-57.602
TMS
OCF3
Br
2h
50 40 30 20 10 0 -10 -20 -30 -40 -50 -60 -70 -80 -90 -100 -110 -120 -130 -140 -150 -160 -170 -180 -190 -200 -210 -220 -230 -240
f1 (ppm)

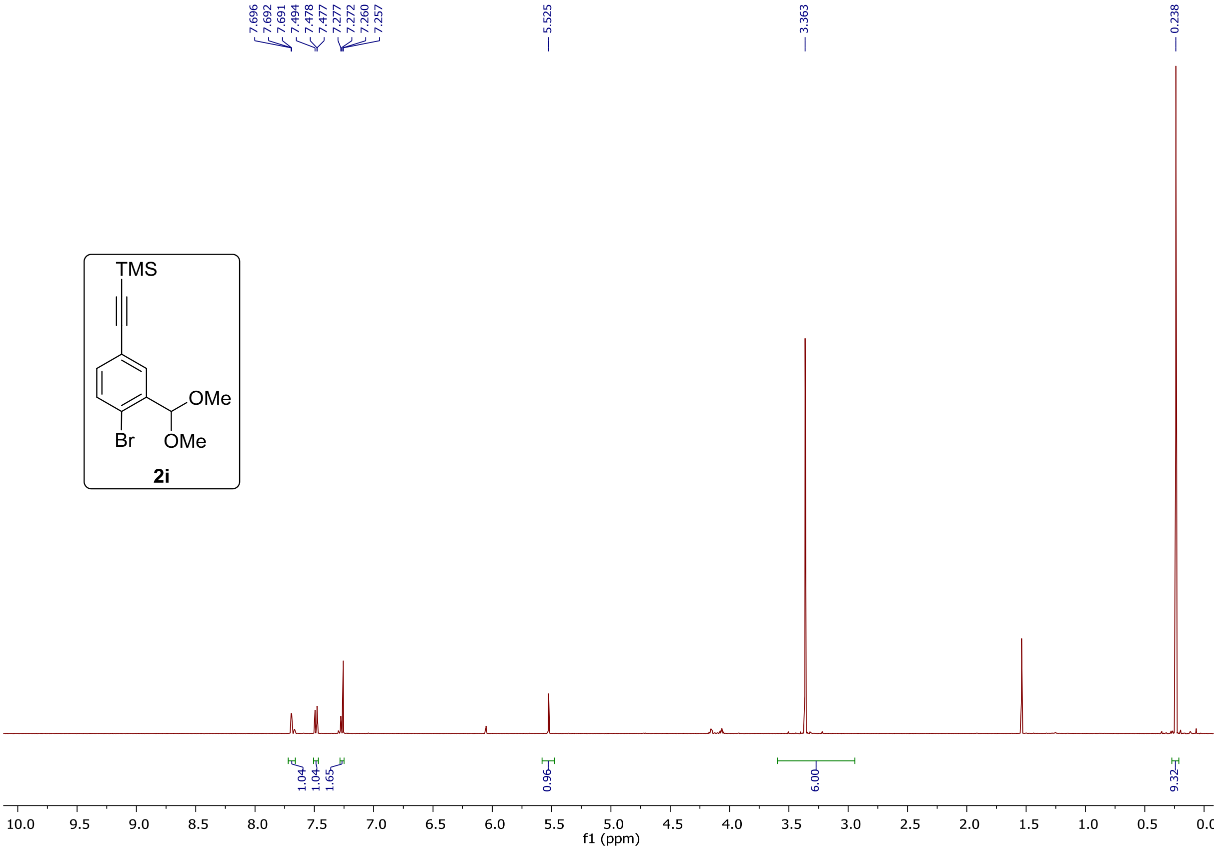
7.696
7.692
7.691
7.494
7.478
7.477
7.277
7.272
7.260
7.257
5.525
3.363
0.238
TMS
OMe
Br
OMe
2i
1.04
1.04
1.65
0.96
6.00
9.32
10.0 9.5 9.0 8.5 8.0 7.5 7.0 6.5 6.0 5.5 5.0 4.5 4.0 3.5 3.0 2.5 2.0 1.5 1.0 0.5
f1 (ppm)

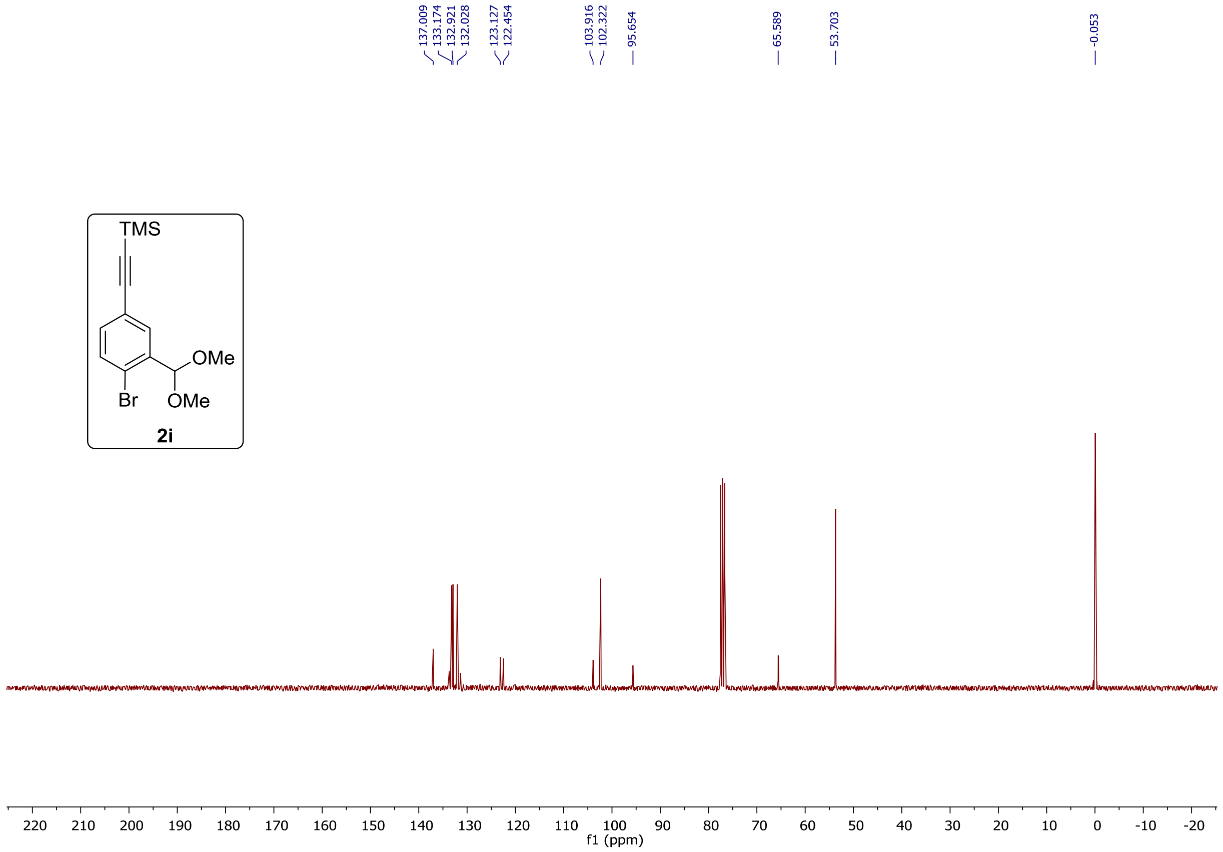
137.009
133.174
132.921
132.028
123.127
122.454
103.916
102.322
95.654
65.589
53.703
-0.053
TMS
OMe
Br
OMe
2i
f1 (ppm)

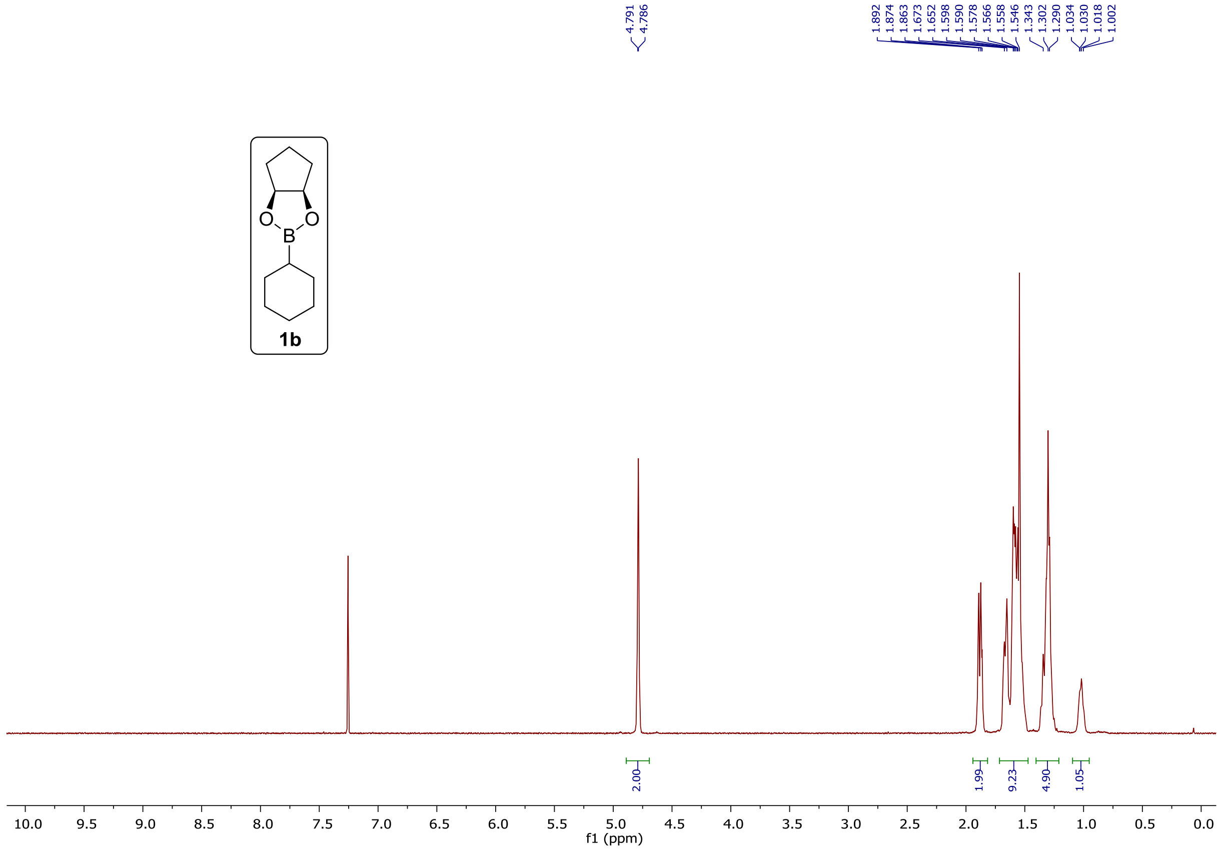
4.791
4.786
1.892
1.874
1.863
1.673
1.652
1.598
1.590
1.578
1.566
1.558
1.546
1.343
1.302
1.290
1.034
1.030
1.018
1.002
O
B
O
1b
2.00
1.99
9.23
4.90
1.05
f1 (ppm)

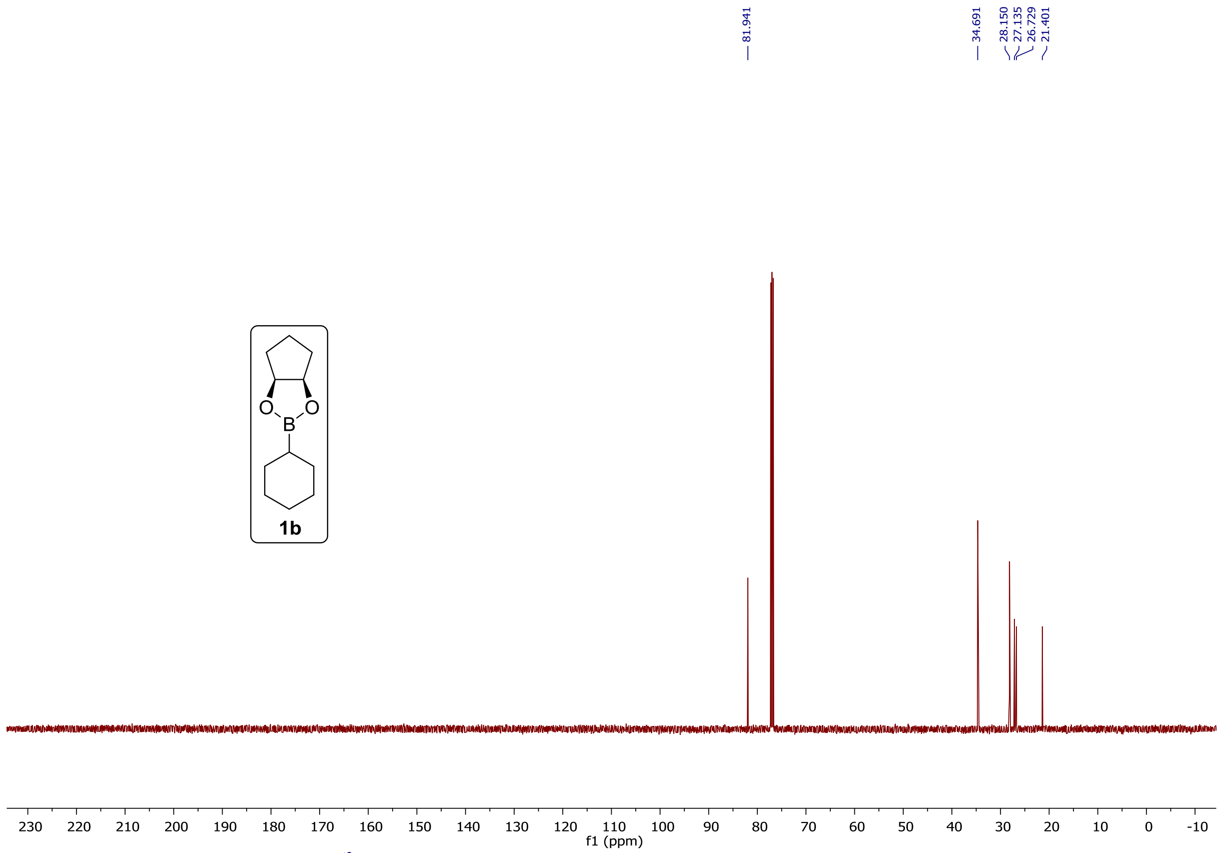
81.941
34.691
28.150
27.135
26.729
21.401
1b
230 220 210 200 190 180 170 160 150 140 130 120 110 100 90 80 70 60 50 40 30 20 10 0 -10
f1 (ppm)

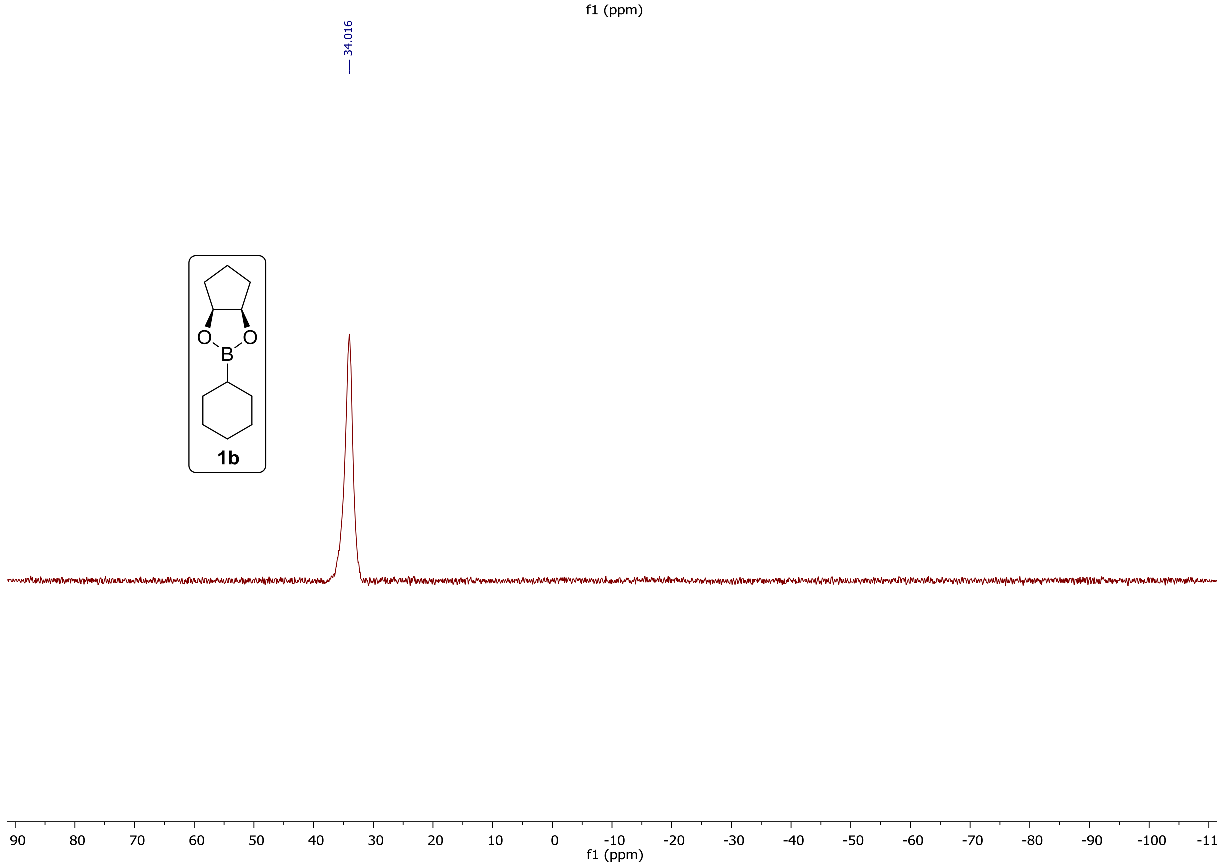
34.016
1b
90 80 70 60 50 40 30 20 10 0 -10 -20 -30 -40 -50 -60 -70 -80 -90 -100 -11
f1 (ppm)

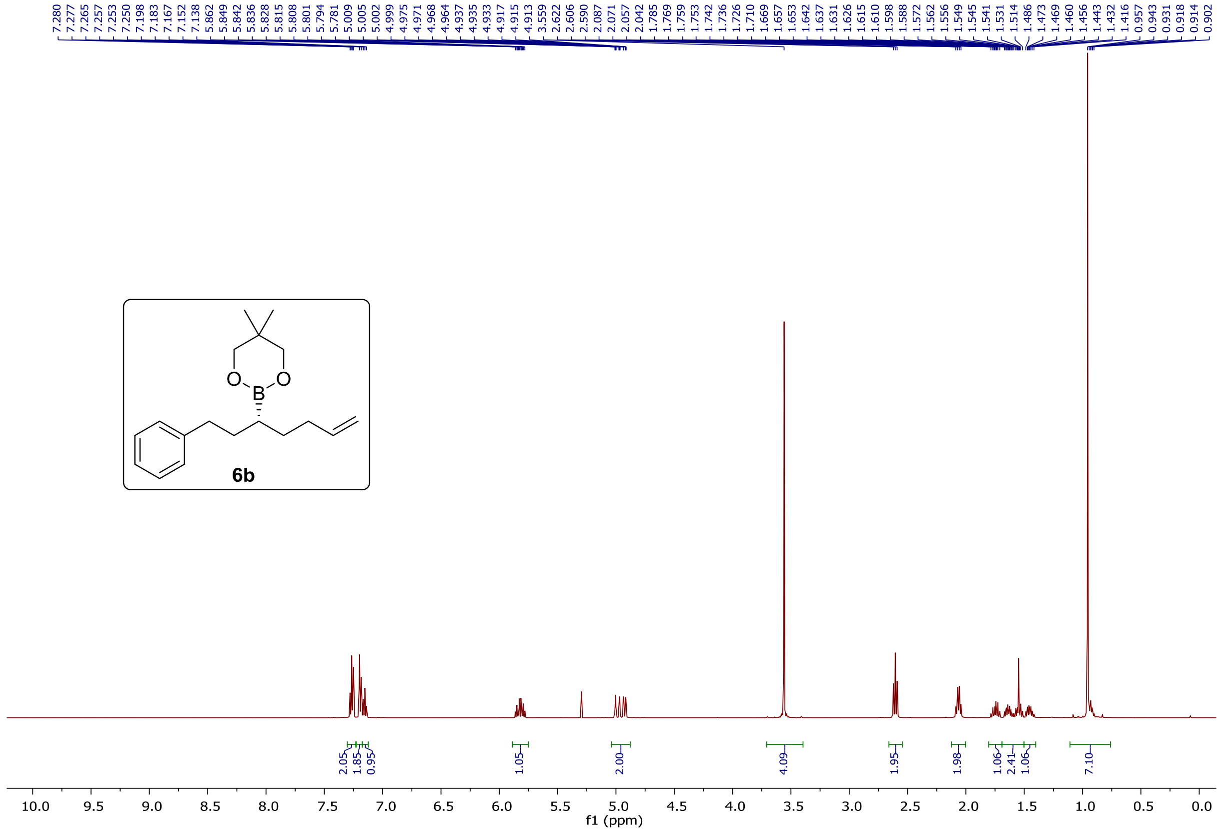
7.280
7.277
7.265
7.257
7.253
7.250
7.198
7.183
7.167
7.152
7.138
5.862
5.849
5.842
5.836
5.828
5.815
5.808
5.801
5.794
5.781
5.009
5.005
5.002
4.999
4.975
4.971
4.968
4.964
4.937
4.935
4.933
4.917
4.915
4.913
3.559
2.622
2.606
2.590
2.087
2.071
2.057
2.042
1.785
1.769
1.759
1.753
1.742
1.736
1.726
1.710
1.669
1.657
1.653
1.642
1.637
1.631
1.626
1.615
1.610
1.598
1.588
1.572
1.562
1.556
1.549
1.545
1.541
1.531
1.514
1.486
1.473
1.469
1.460
1.456
1.443
1.432
1.416
0.957
0.943
0.931
0.918
0.914
0.902
O
B
O
6b
2.05
1.85
0.95
1.05
2.00
4.09
1.95
1.98
1.06
2.41
1.06
7.10
f1 (ppm)

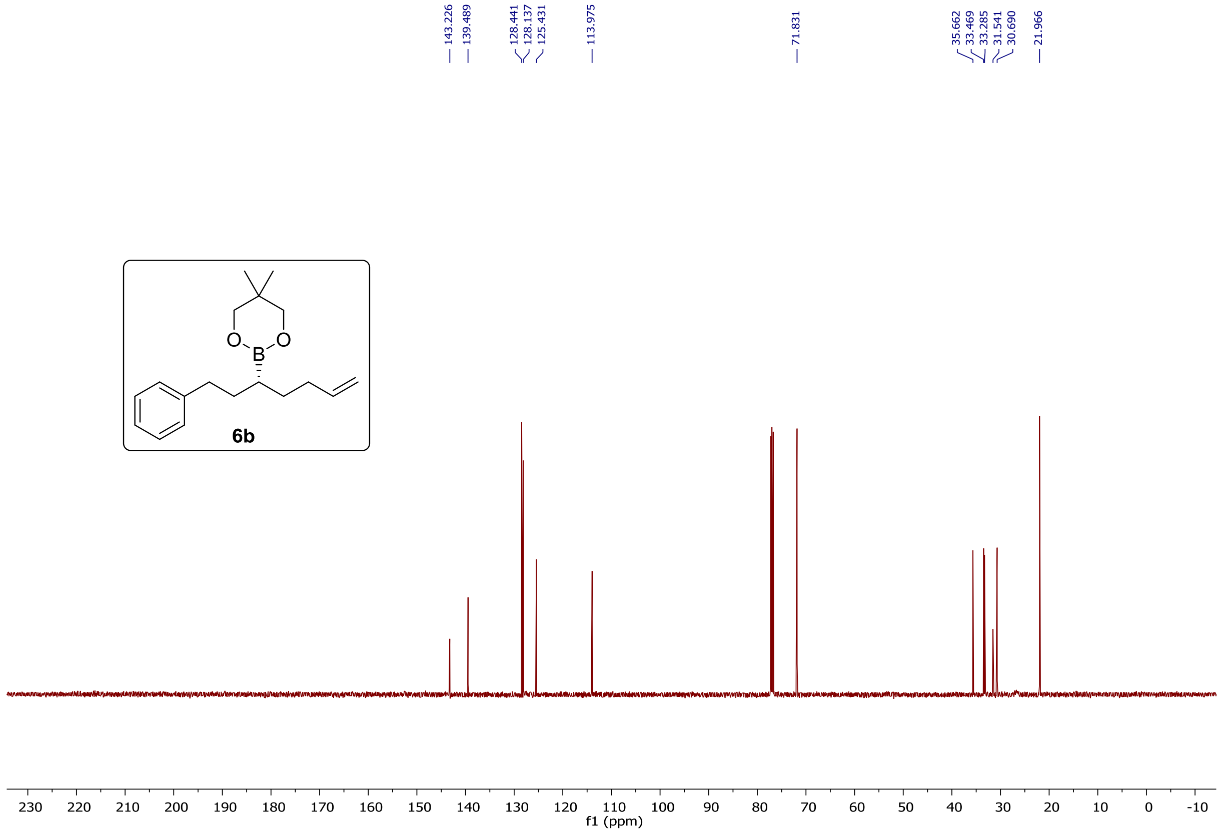
143.226
139.489
128.441
128.137
125.431
113.975
71.831
35.662
33.469
33.285
31.541
30.690
21.966
O
B
O
6b
f1 (ppm)

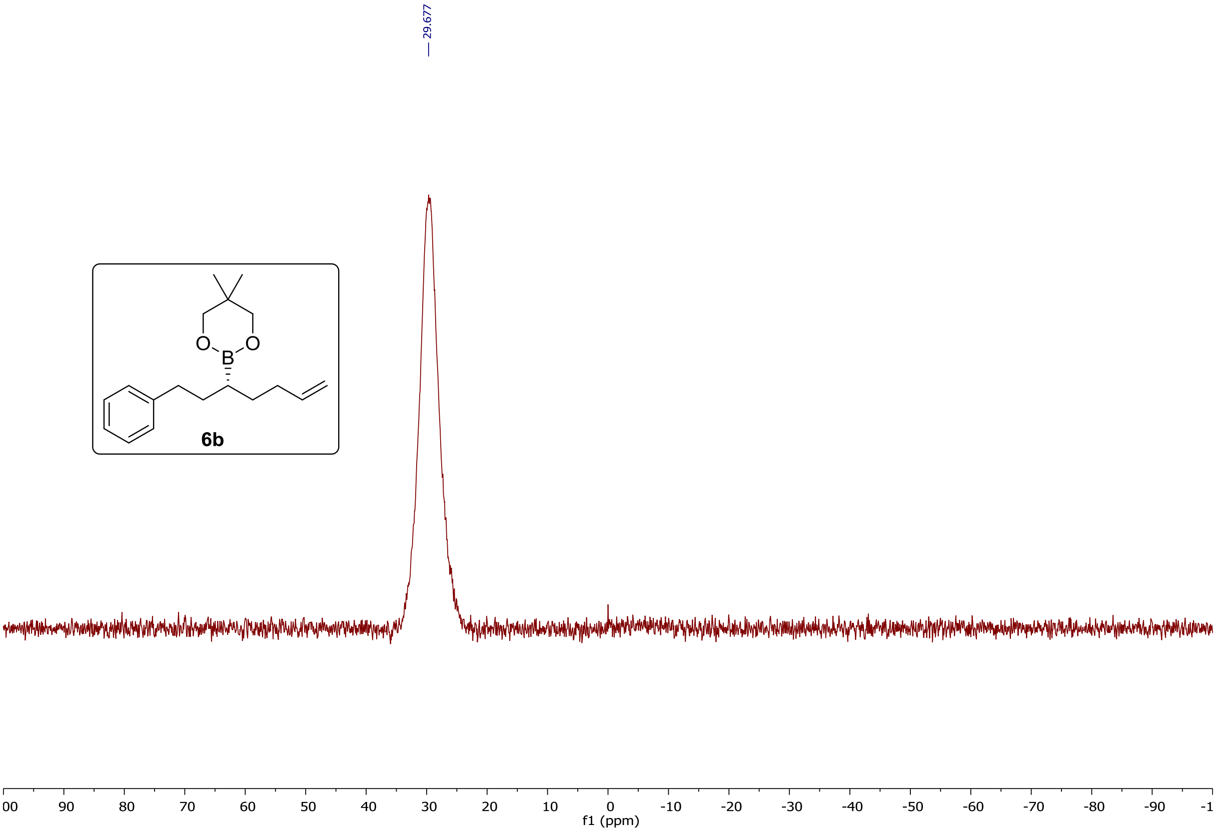
29.677
6b
00 90 80 70 60 50 40 30 20 10 0 -10 -20 -30 -40 -50 -60 -70 -80 -90 -1
f1 (ppm)

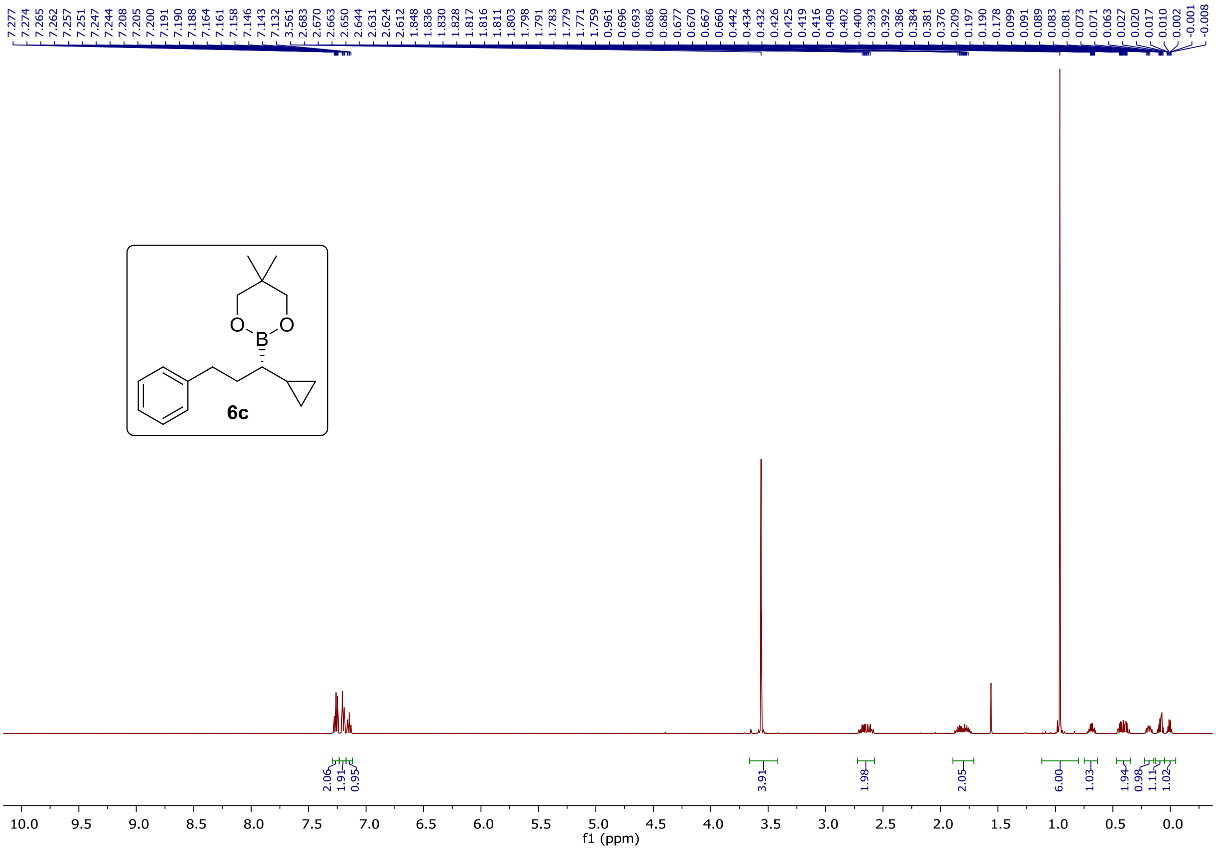
7.277 7.274 7.265 7.262 7.257 7.251 7.247 7.244 7.208 7.205 7.200 7.191 7.190 7.188 7.164 7.161 7.158 7.146 7.143 7.132 3.561 2.683 2.670 2.663 2.650 2.644 2.631 2.624 2.612 1.848 1.836 1.830 1.828 1.817 1.816 1.811 1.803 1.798 1.791 1.783 1.779 1.771 1.759 0.961 0.696 0.693 0.686 0.680 0.677 0.667 0.660 0.442 0.434 0.432 0.426 0.425 0.419 0.416 0.409 0.402 0.400 0.393 0.392 0.386 0.384 0.381 0.376 0.209 0.197 0.190 0.178 0.099 0.091 0.089 0.083 0.081 0.073 0.071 0.063 0.027 0.020 0.017 0.010 0.002 -0.001 -0.008
6c
2.06 1.91 0.95 3.91 1.98 2.05 6.00 1.03 1.94 0.98 1.11 1.02
10.0 9.5 9.0 8.5 8.0 7.5 7.0 6.5 6.0 5.5 5.0 4.5 4.0 3.5 3.0 2.5 2.0 1.5 1.0 0.5 0.0
f1 (ppm)

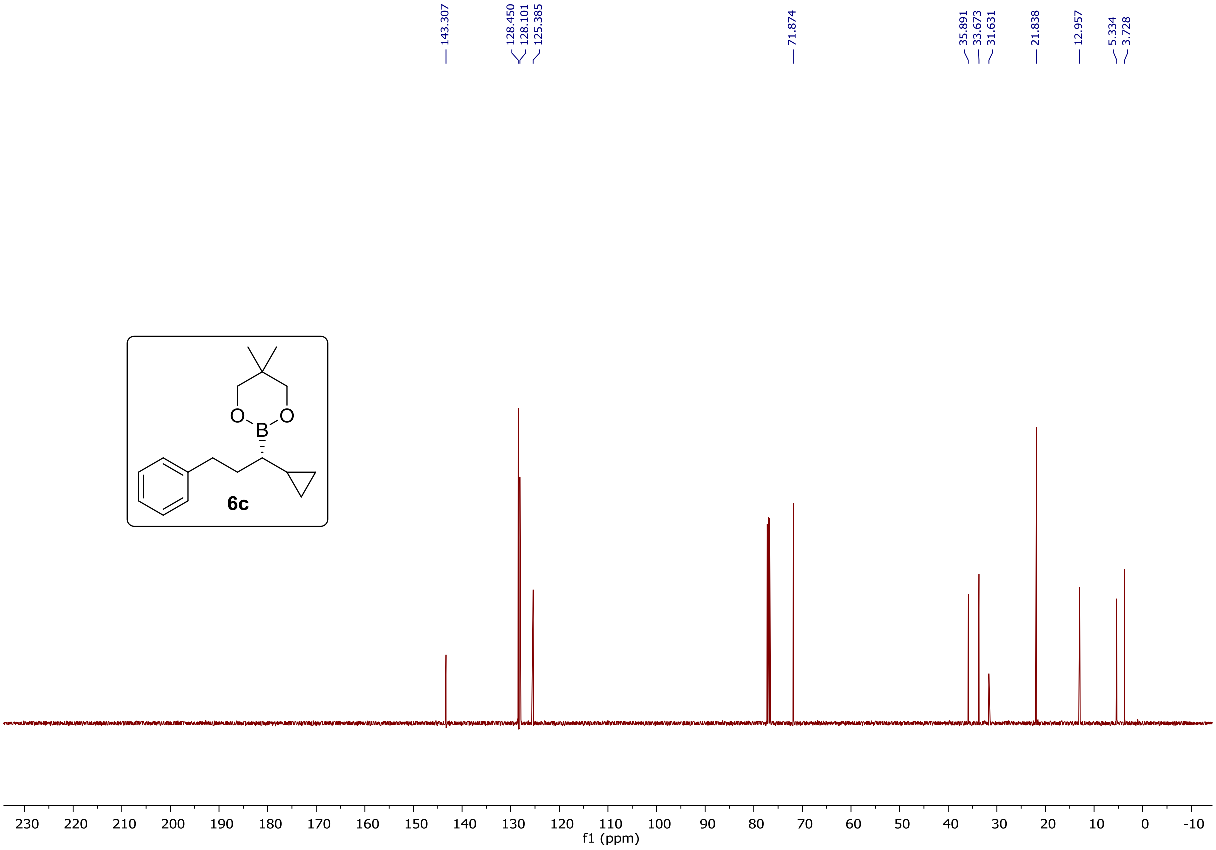
143.307
128.450
128.101
125.385
71.874
35.891
33.673
31.631
21.838
12.957
5.334
3.728
6c
230 220 210 200 190 180 170 160 150 140 130 120 110 100 90 80 70 60 50 40 30 20 10 0 -10
f1 (ppm)

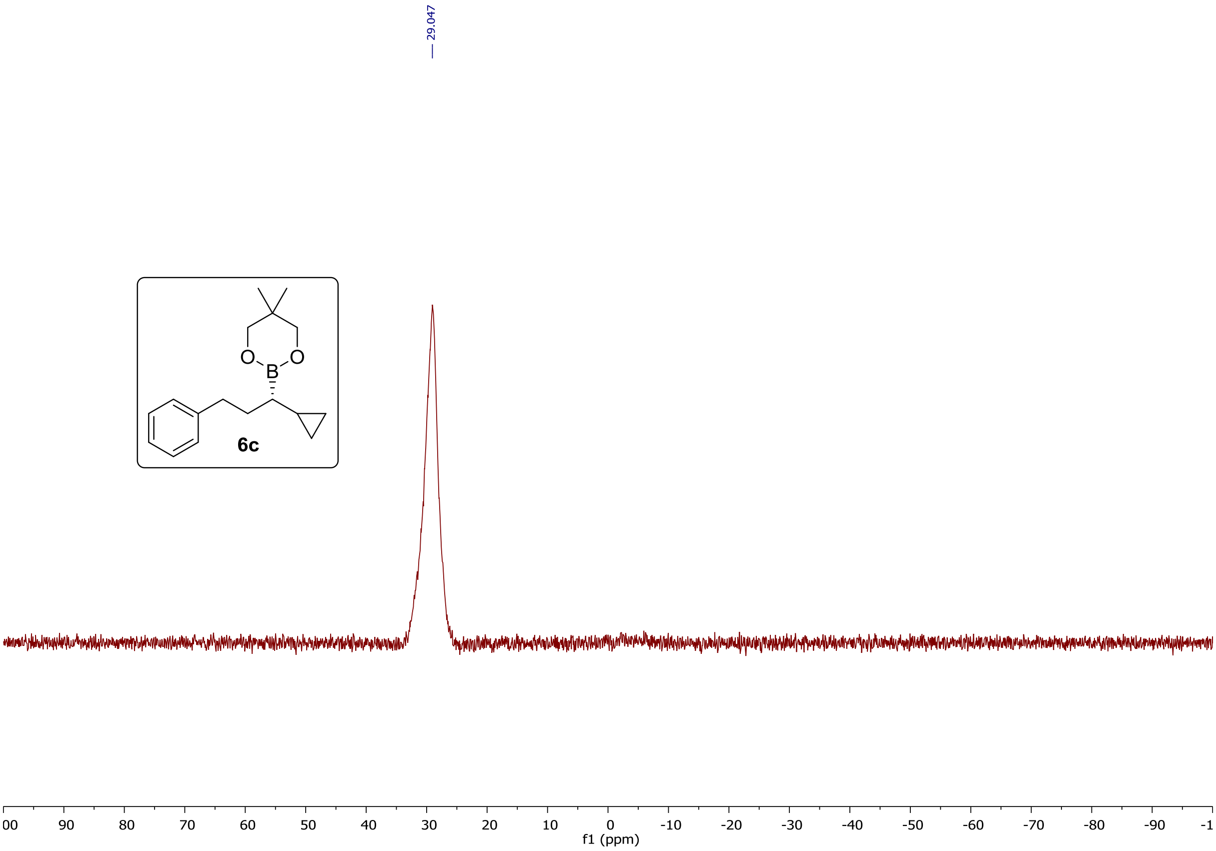
29.047
6c
00 90 80 70 60 50 40 30 20 10 0 -10 -20 -30 -40 -50 -60 -70 -80 -90 -1
f1 (ppm)

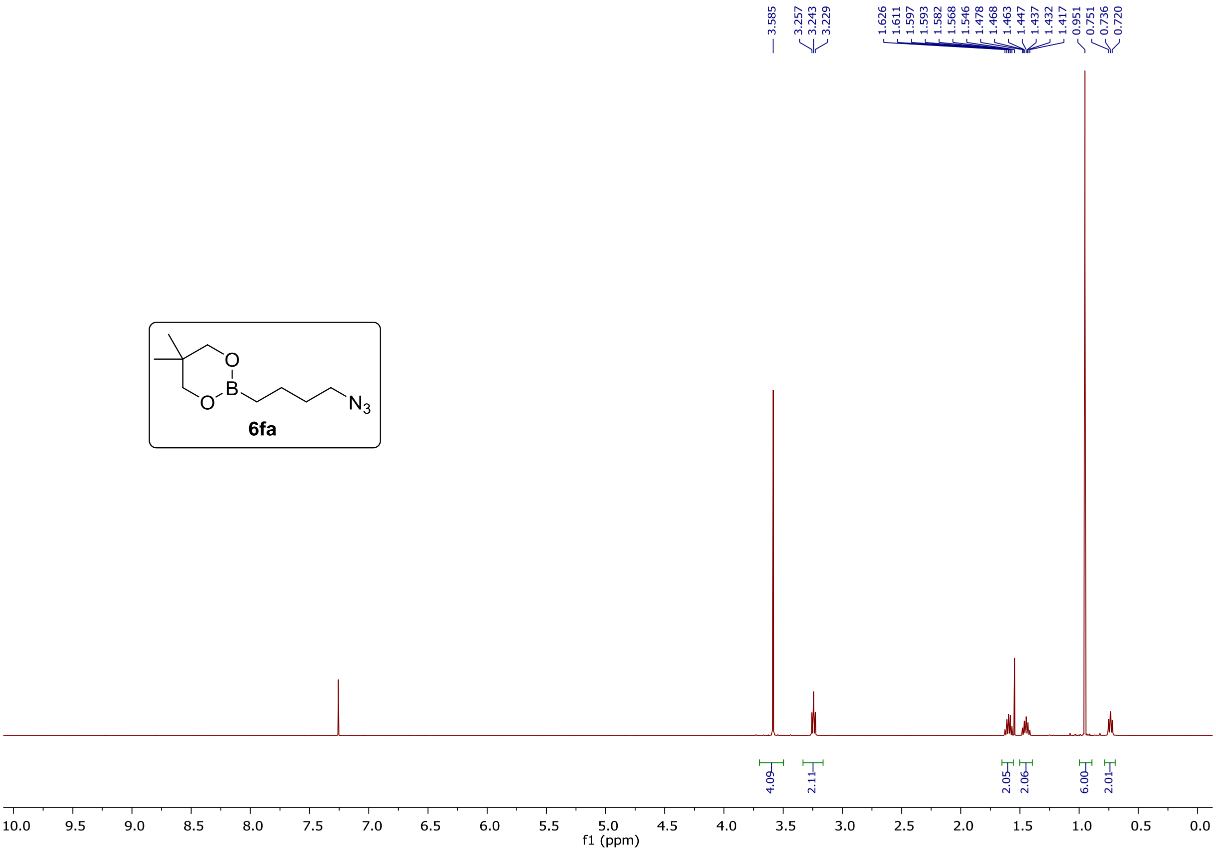
3.585
3.257
3.243
3.229
1.626
1.611
1.597
1.593
1.582
1.568
1.546
1.478
1.468
1.463
1.447
1.437
1.432
1.417
0.951
0.751
0.736
0.720
O
B
O
N3
6fa
4.09
2.11
2.05
2.06
6.00
2.01
10.0 9.5 9.0 8.5 8.0 7.5 7.0 6.5 6.0 5.5 5.0 4.5 4.0 3.5 3.0 2.5 2.0 1.5 1.0 0.5 0.0
f1 (ppm)

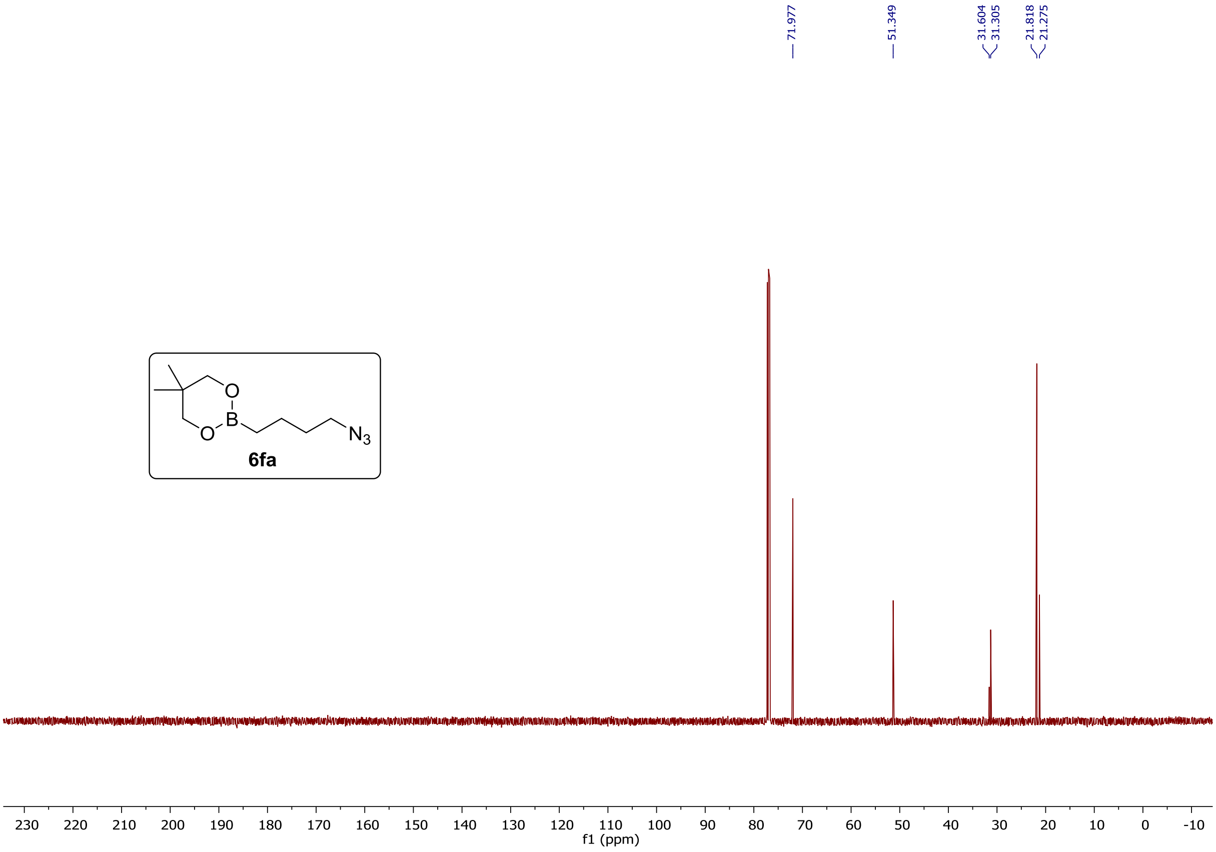
71.977
51.349
31.604
31.305
21.818
21.275
O
B
O
N3
6fa
230 220 210 200 190 180 170 160 150 140 130 120 110 100 90 80 70 60 50 40 30 20 10 0 -10
f1 (ppm)

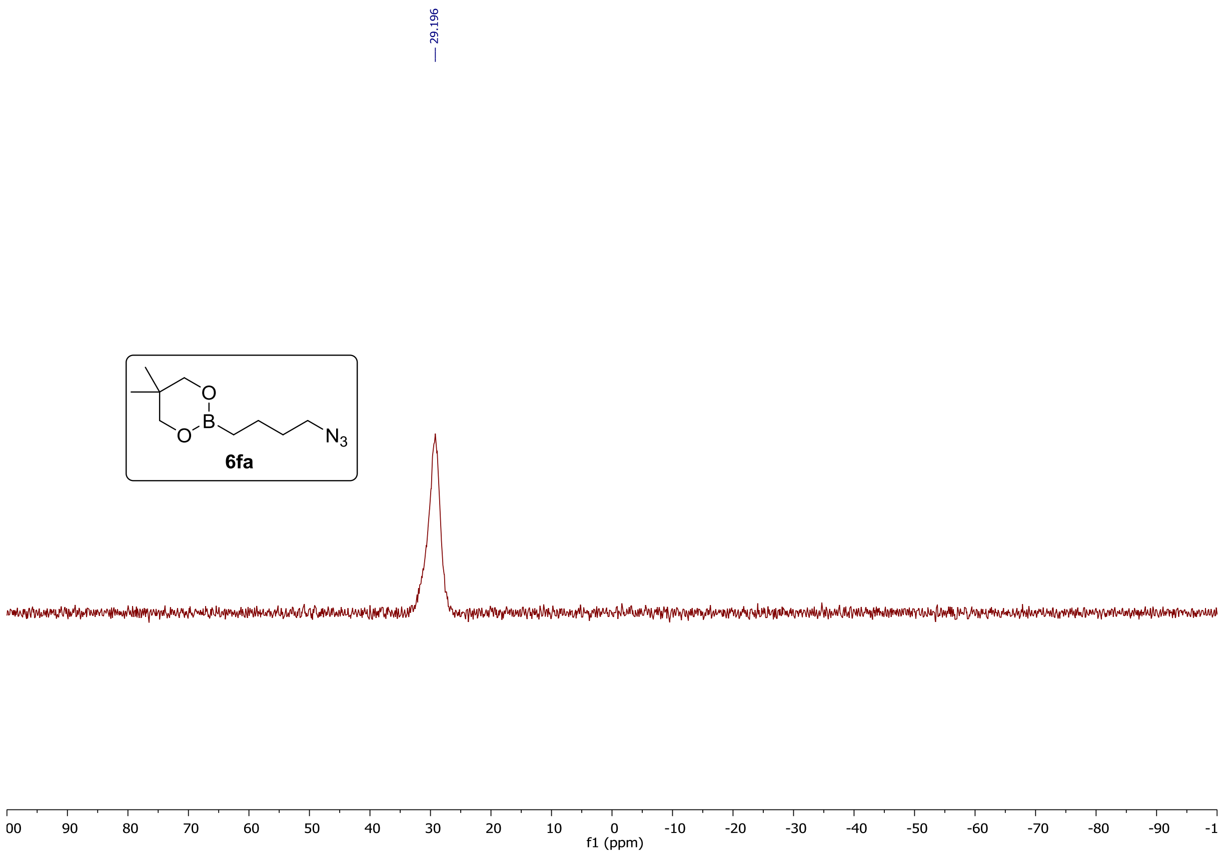
29.196
6fa
f1 (ppm)

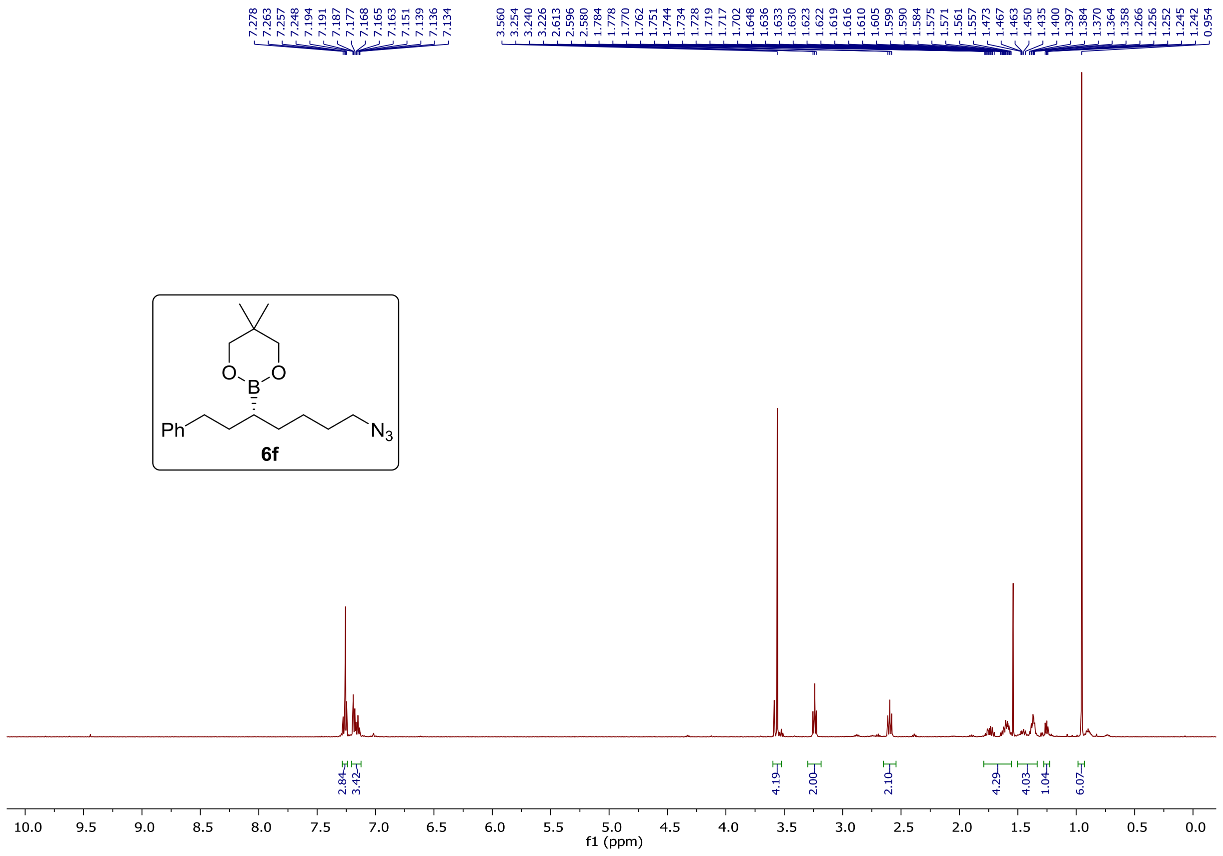
6f
f1 (ppm)

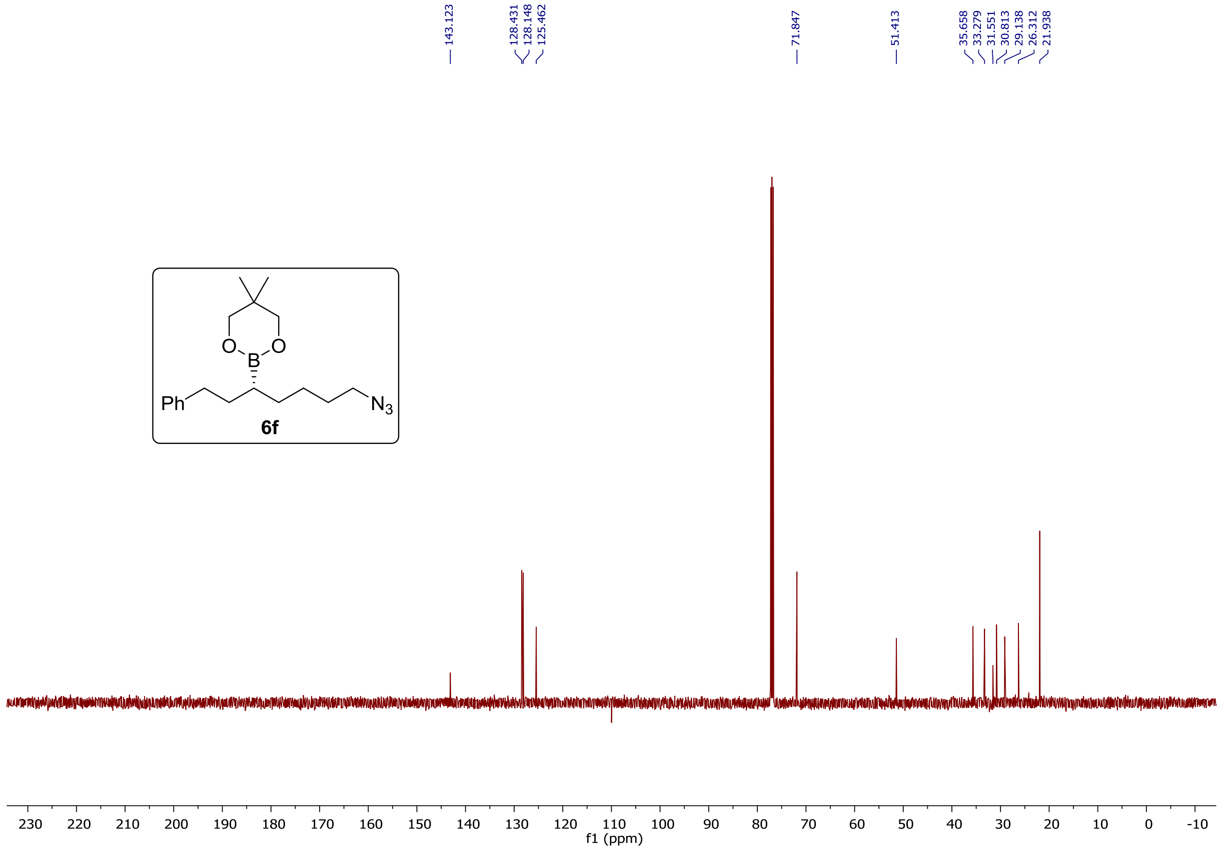
143.123
128.431
128.148
125.462
71.847
51.413
35.658
33.279
31.551
30.813
29.138
26.312
21.938
Ph
N3
6f
230 220 210 200 190 180 170 160 150 140 130 120 110 100 90 80 70 60 50 40 30 20 10 0 -10
f1 (ppm)

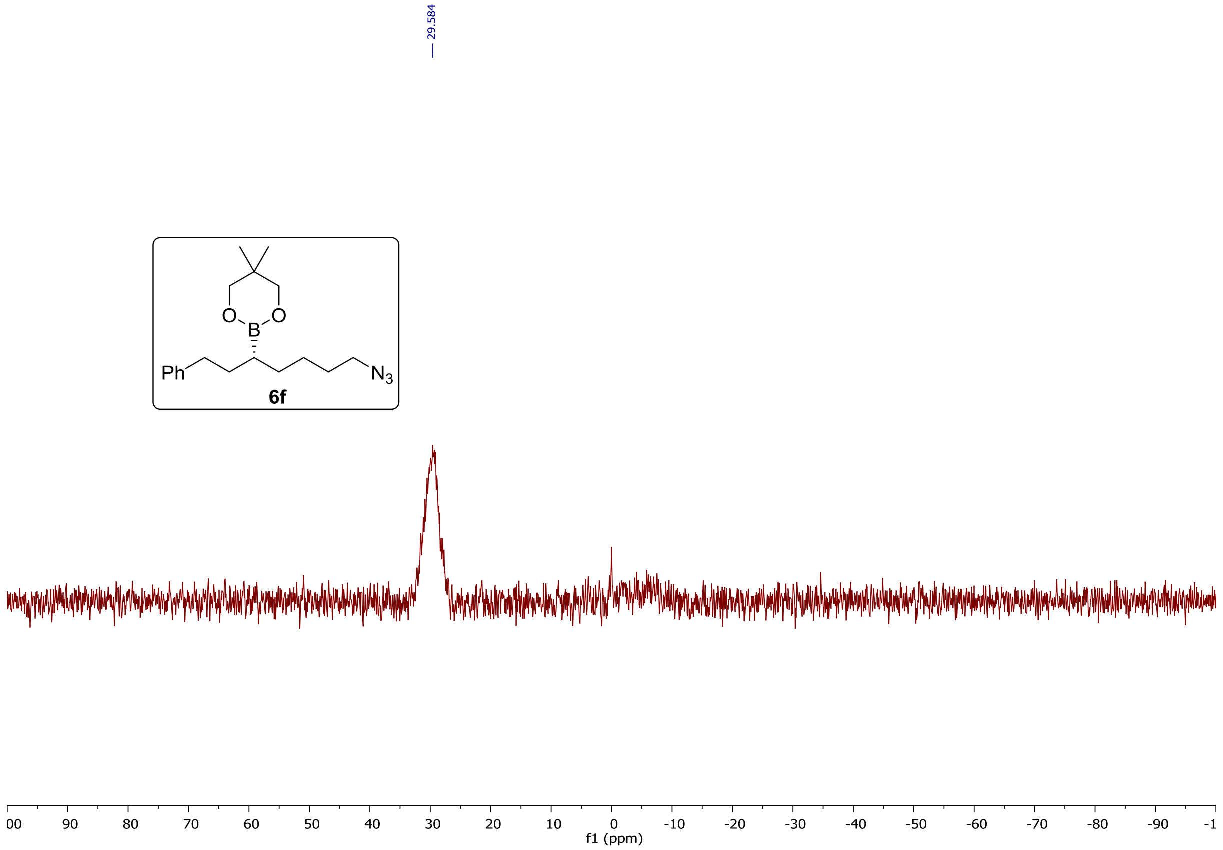
29.584
Ph
N3
6f
00 90 80 70 60 50 40 30 20 10 0 -10 -20 -30 -40 -50 -60 -70 -80 -90 -1
f1 (ppm)

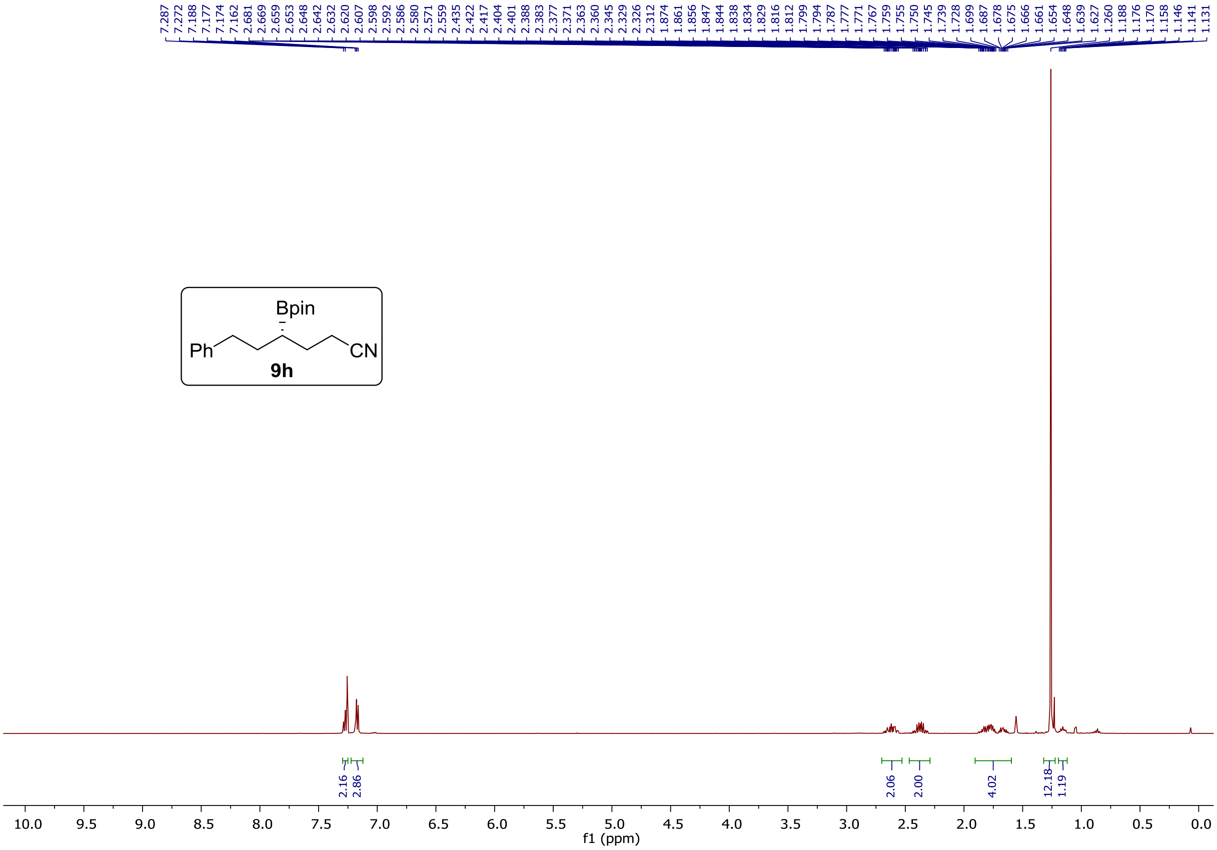
7.287 7.272 7.188 7.177 7.174 7.162 2.681 2.669 2.659 2.653 2.648 2.642 2.632 2.620 2.607 2.598 2.592 2.586 2.580 2.571 2.559 2.435 2.422 2.417 2.404 2.401 2.388 2.383 2.377 2.371 2.363 2.360 2.345 2.329 2.326 2.312 1.874 1.861 1.856 1.847 1.844 1.838 1.834 1.829 1.816 1.812 1.799 1.794 1.787 1.777 1.771 1.767 1.759 1.755 1.750 1.745 1.739 1.728 1.699 1.687 1.678 1.675 1.666 1.661 1.654 1.648 1.639 1.627 1.260 1.188 1.176 1.170 1.158 1.146 1.141 1.131
Bpin
Ph
CN
9h
2.16
2.86
2.06
2.00
4.02
12.18
1.19
f1 (ppm)

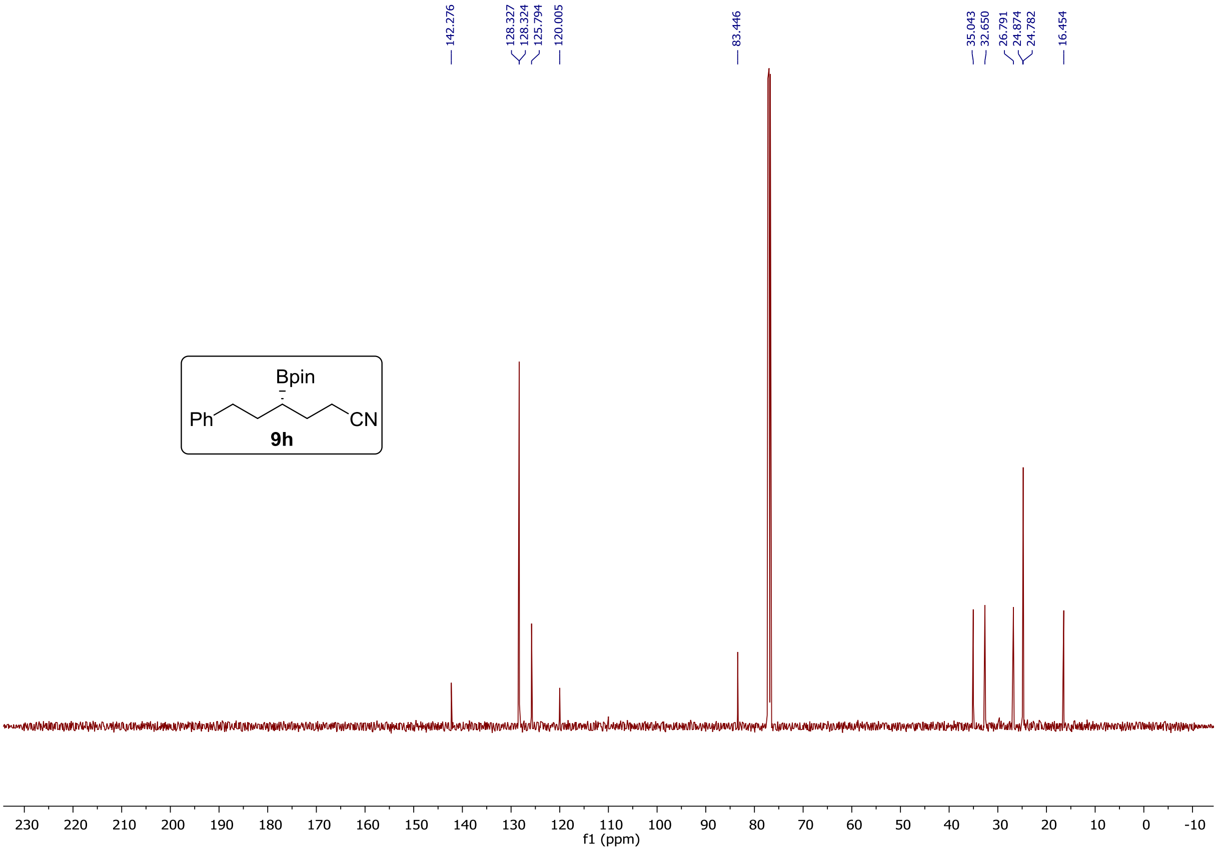
142.276
128.327 128.324 125.794
120.005
83.446
35.043 32.650 26.791 24.874 24.782
16.454
Bpin
Ph
CN
9h
f1 (ppm)

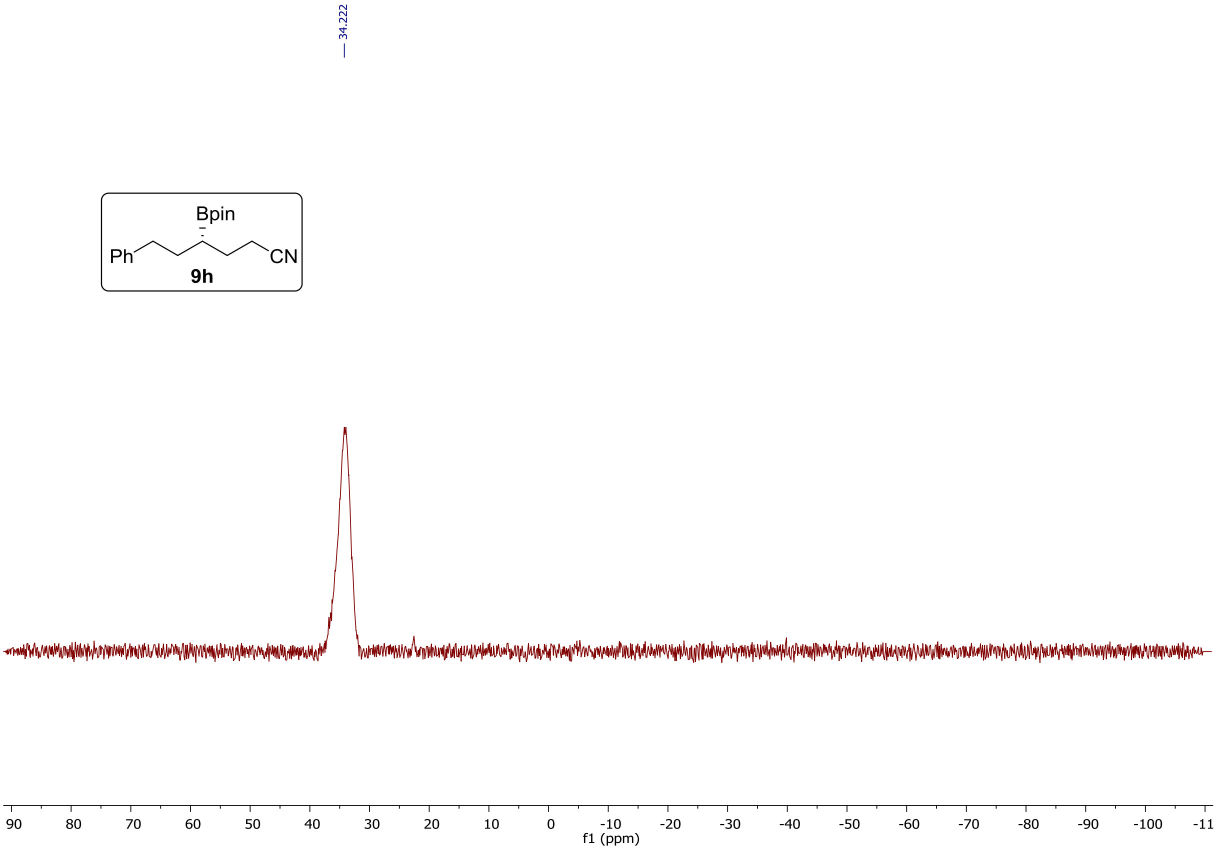
34.222
Bpin
Ph
CN
9h
90 80 70 60 50 40 30 20 10 0 -10 -20 -30 -40 -50 -60 -70 -80 -90 -100 -11
f1 (ppm)

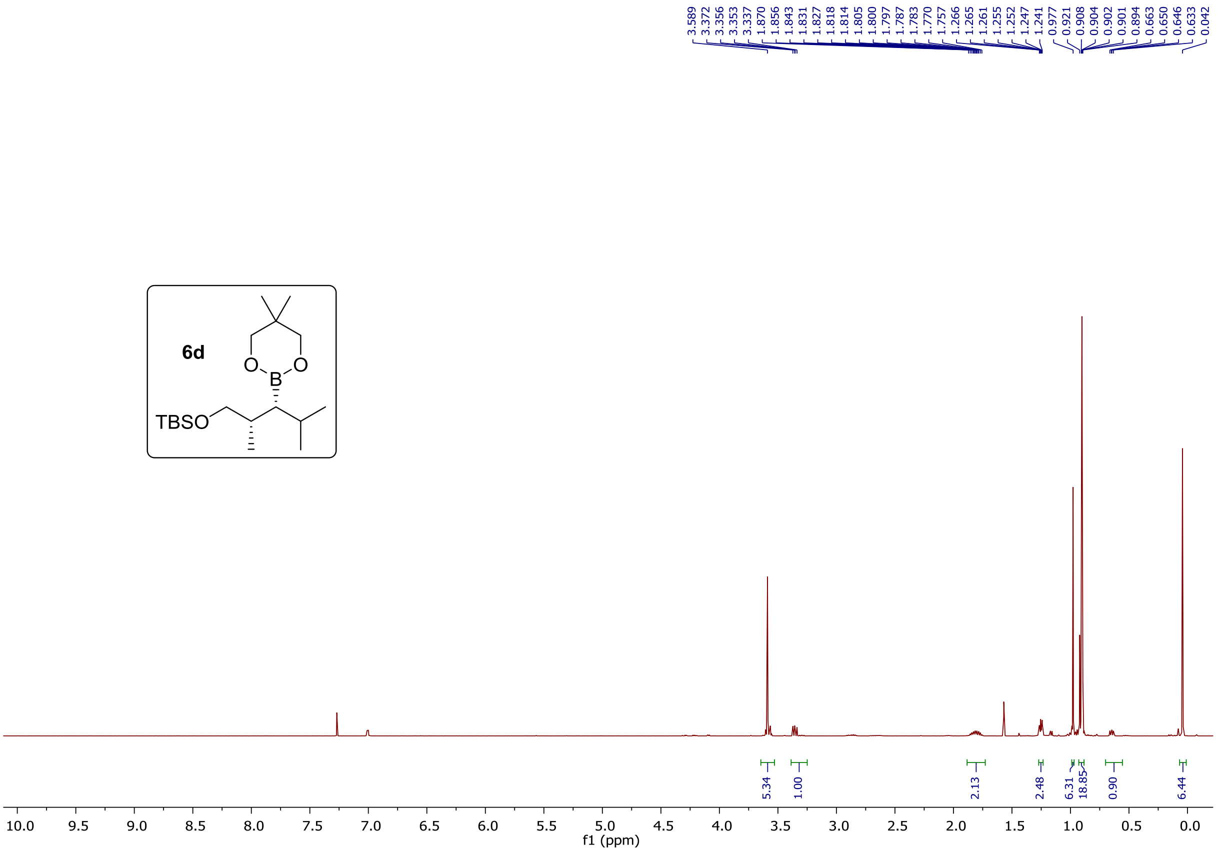
3.589 3.372 3.356 3.353 3.337 1.870 1.856 1.843 1.831 1.827 1.818 1.814 1.805 1.800 1.797 1.787 1.783 1.770 1.757 1.266 1.265 1.261 1.255 1.252 1.247 1.241 0.977 0.921 0.908 0.904 0.902 0.901 0.894 0.663 0.650 0.646 0.633 0.042
6d
O
B
O
TBSO
5.34 1.00 2.13 2.48 6.31 18.85 0.90 6.44
10.0 9.5 9.0 8.5 8.0 7.5 7.0 6.5 6.0 5.5 5.0 4.5 4.0 3.5 3.0 2.5 2.0 1.5 1.0 0.5 0.0
f1 (ppm)

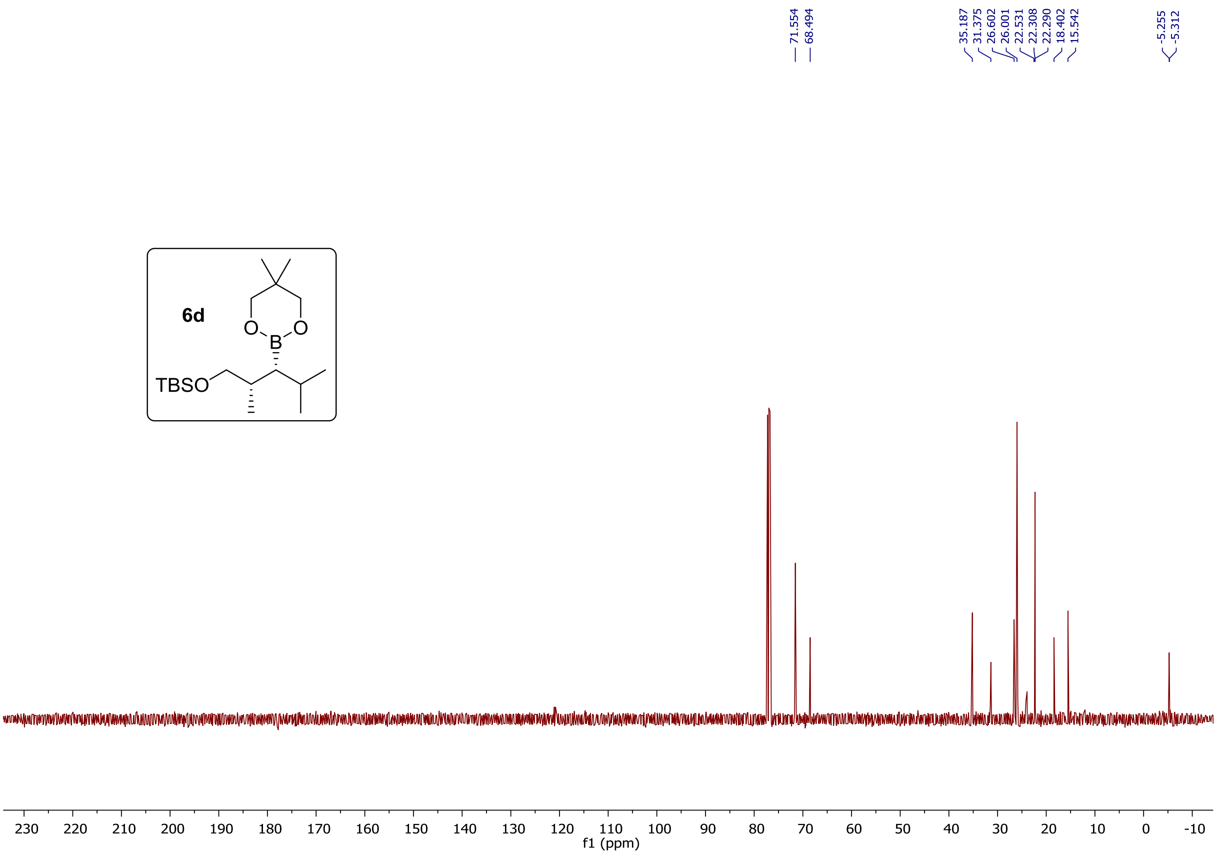
71.554
68.494
35.187
31.375
26.602
26.001
22.531
22.308
22.290
18.402
15.542
-5.255
-5.312
6d
TBSO
f1 (ppm)

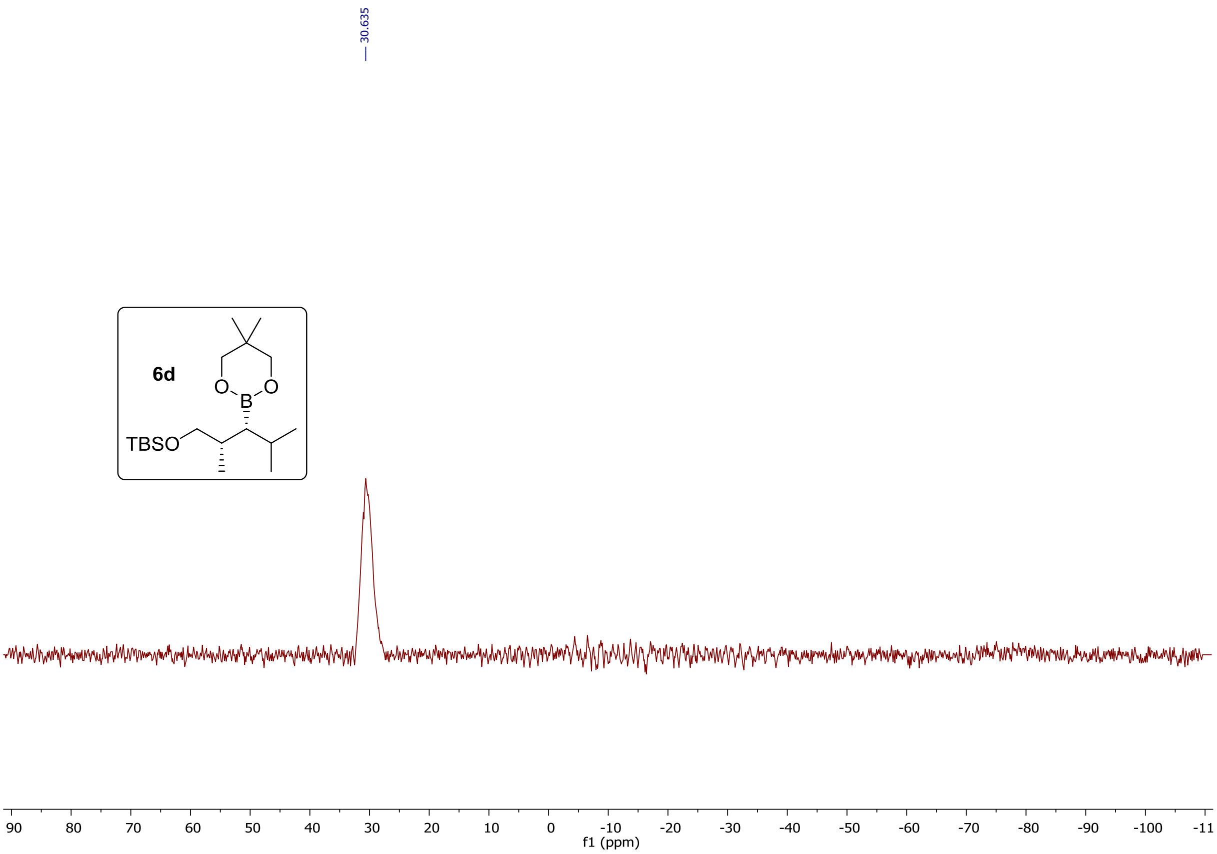
30.635
6d
TBSO
f1 (ppm)

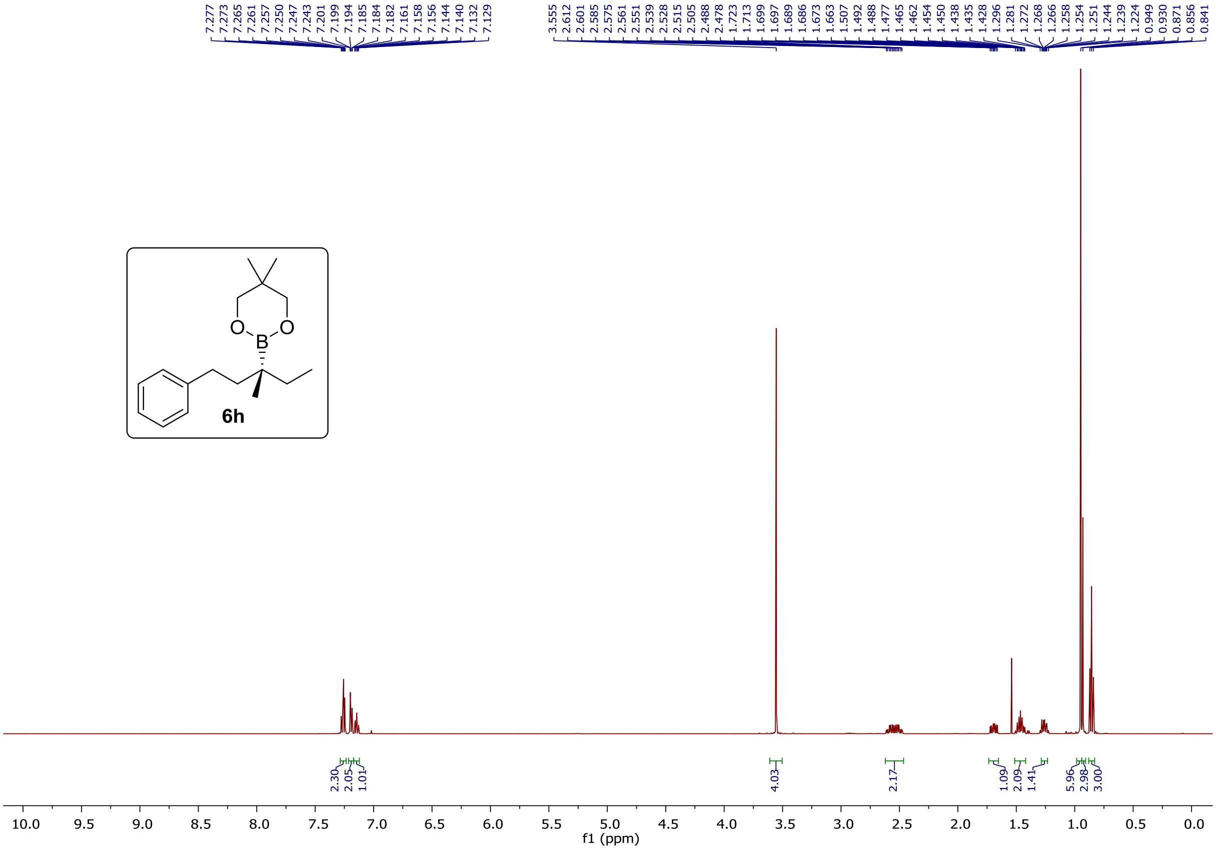
7.277 7.273 7.265 7.261 7.257 7.250 7.247 7.243 7.201 7.199 7.194 7.185 7.184 7.182 7.161 7.158 7.156 7.144 7.140 7.132 7.129
3.555 2.612 2.601 2.585 2.575 2.561 2.551 2.539 2.528 2.515 2.505 2.488 2.478 1.723 1.713 1.699 1.697 1.689 1.686 1.673 1.663 1.507 1.492 1.488 1.477 1.465 1.462 1.454 1.450 1.438 1.435 1.428 1.296 1.281 1.272 1.268 1.266 1.258 1.254 1.251 1.244 1.239 1.224 0.949 0.930 0.871 0.856 0.841
O
B
O
6h
2.30 2.05 1.01 4.03 2.17 1.09 2.09 1.41 5.96 2.98 3.00
f1 (ppm)

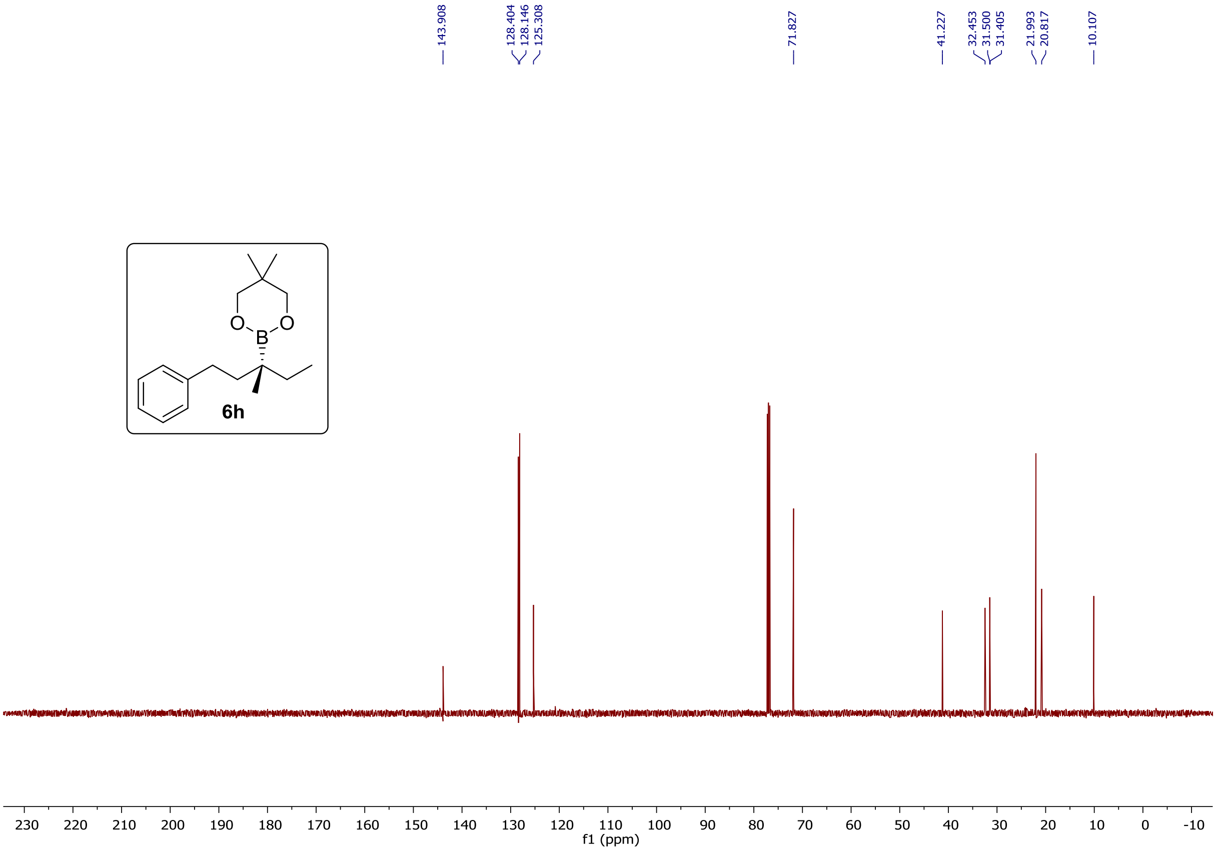
143.908
128.404 128.146 125.308
71.827
41.227
32.453 31.500 31.405
21.993 20.817
10.107
O
B
O
6h
f1 (ppm)

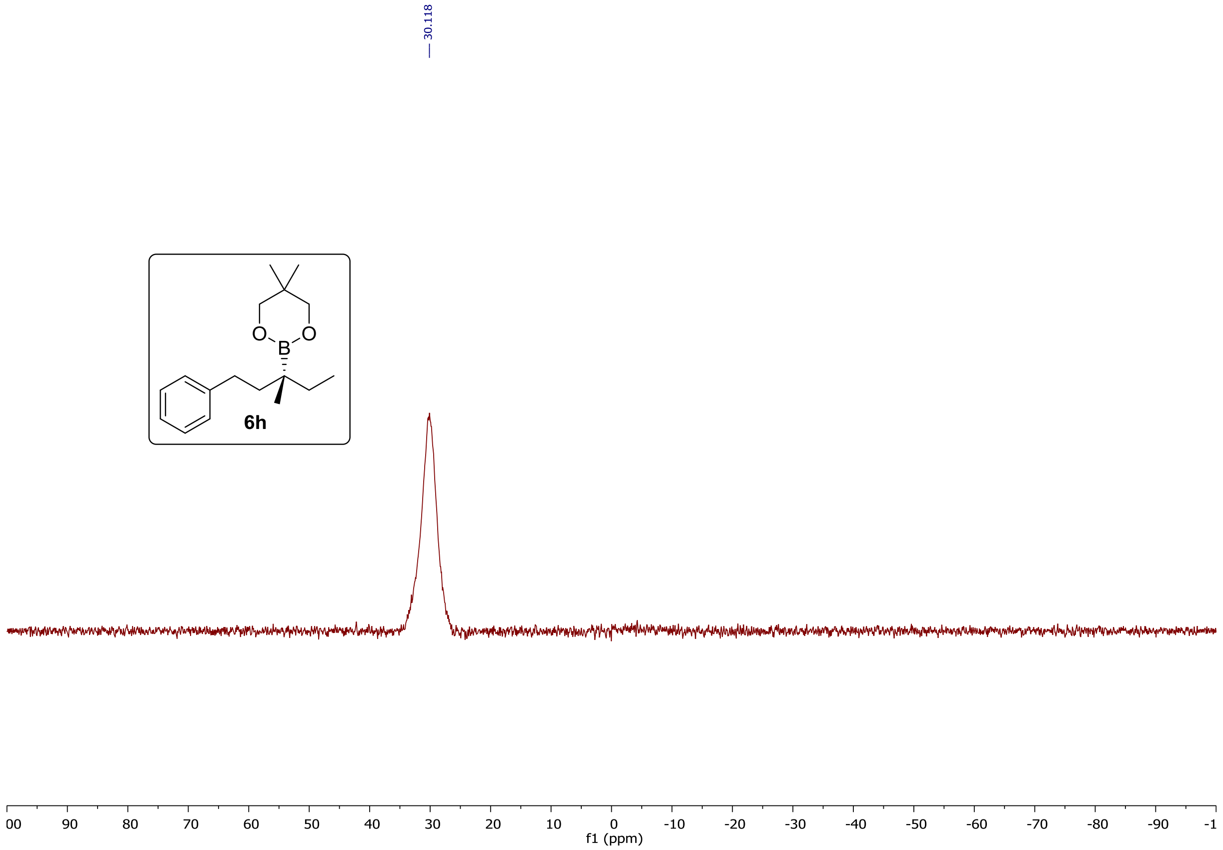
30.118
6h
00 90 80 70 60 50 40 30 20 10 0 -10 -20 -30 -40 -50 -60 -70 -80 -90
f1 (ppm)

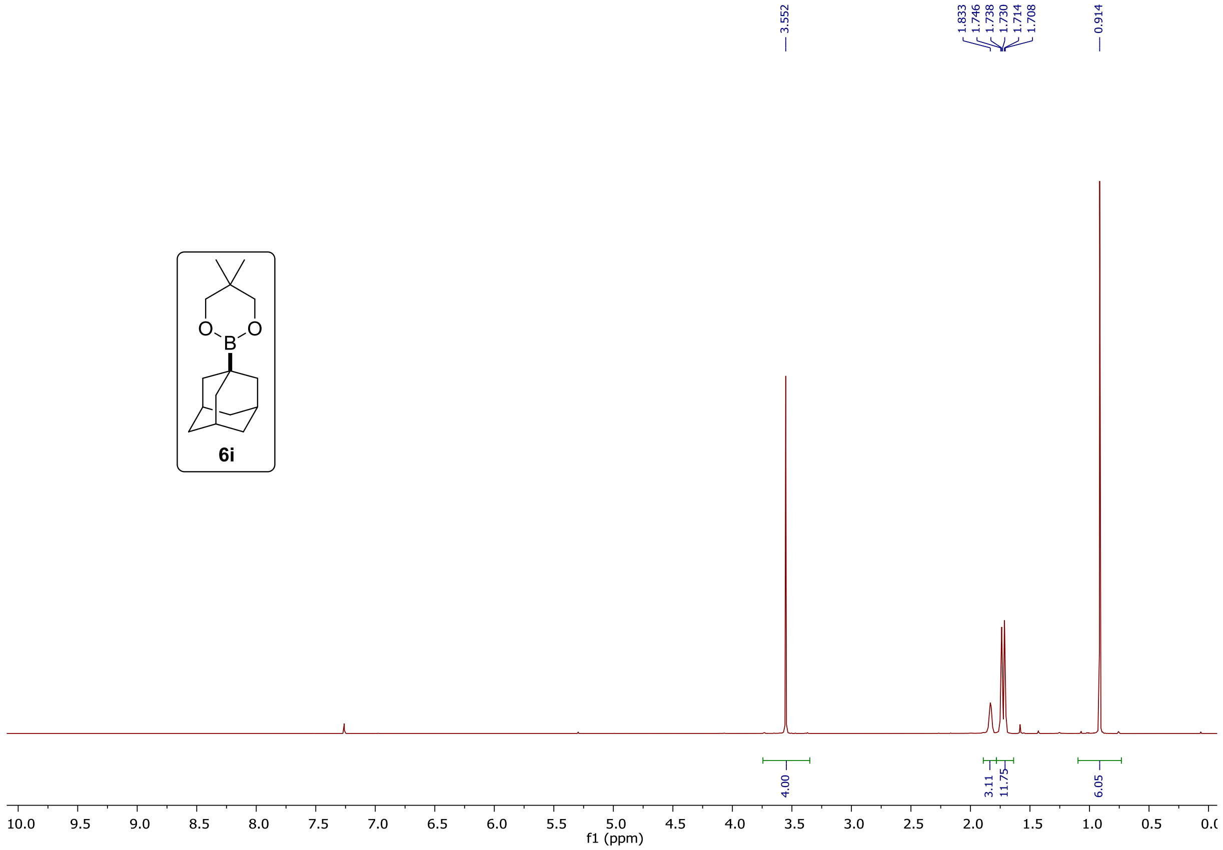
3.552
1.833
1.746
1.738
1.730
1.714
1.708
0.914
6i
4.00
3.11
11.75
6.05
10.0 9.5 9.0 8.5 8.0 7.5 7.0 6.5 6.0 5.5 5.0 4.5 4.0 3.5 3.0 2.5 2.0 1.5 1.0 0.5
f1 (ppm)

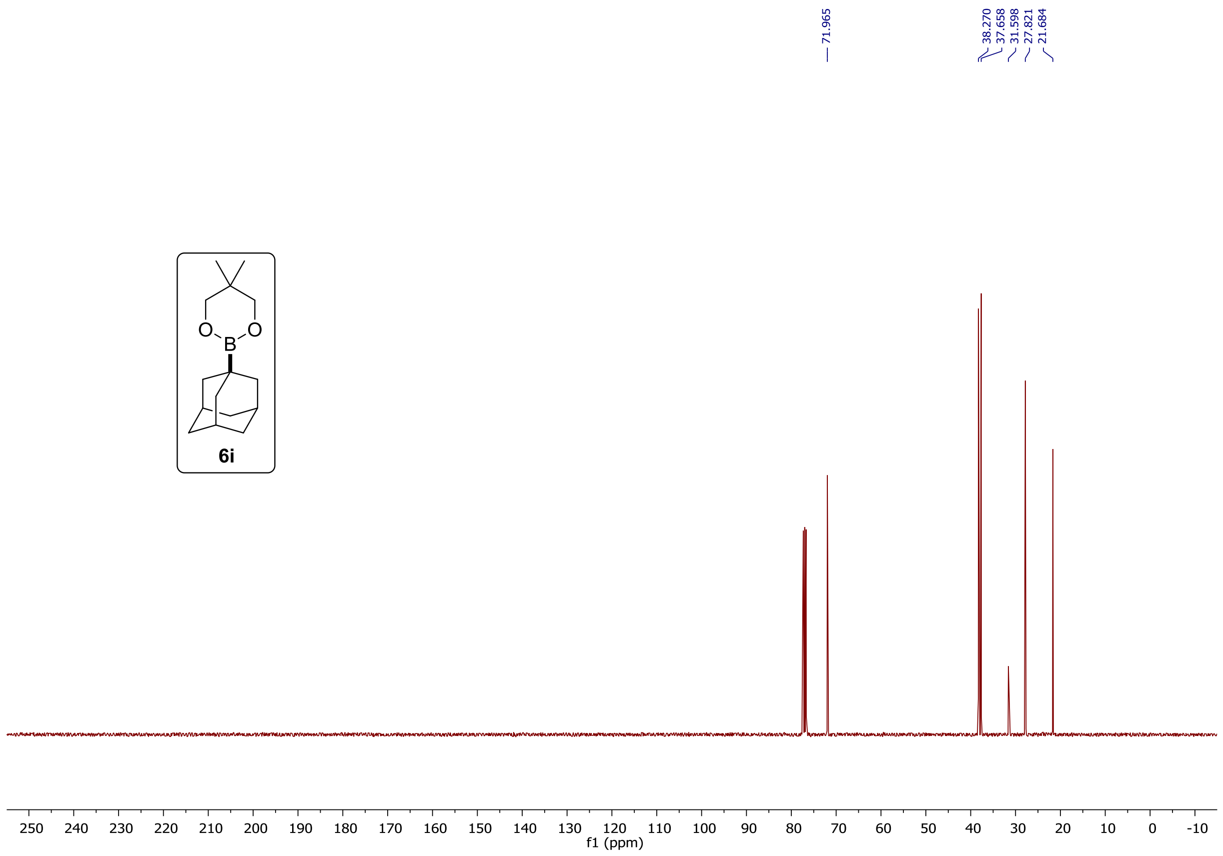
71.965
38.270
37.658
31.598
27.821
21.684
6i
250 240 230 220 210 200 190 180 170 160 150 140 130 120 110 100 90 80 70 60 50 40 30 20 10 0 -10
f1 (ppm)

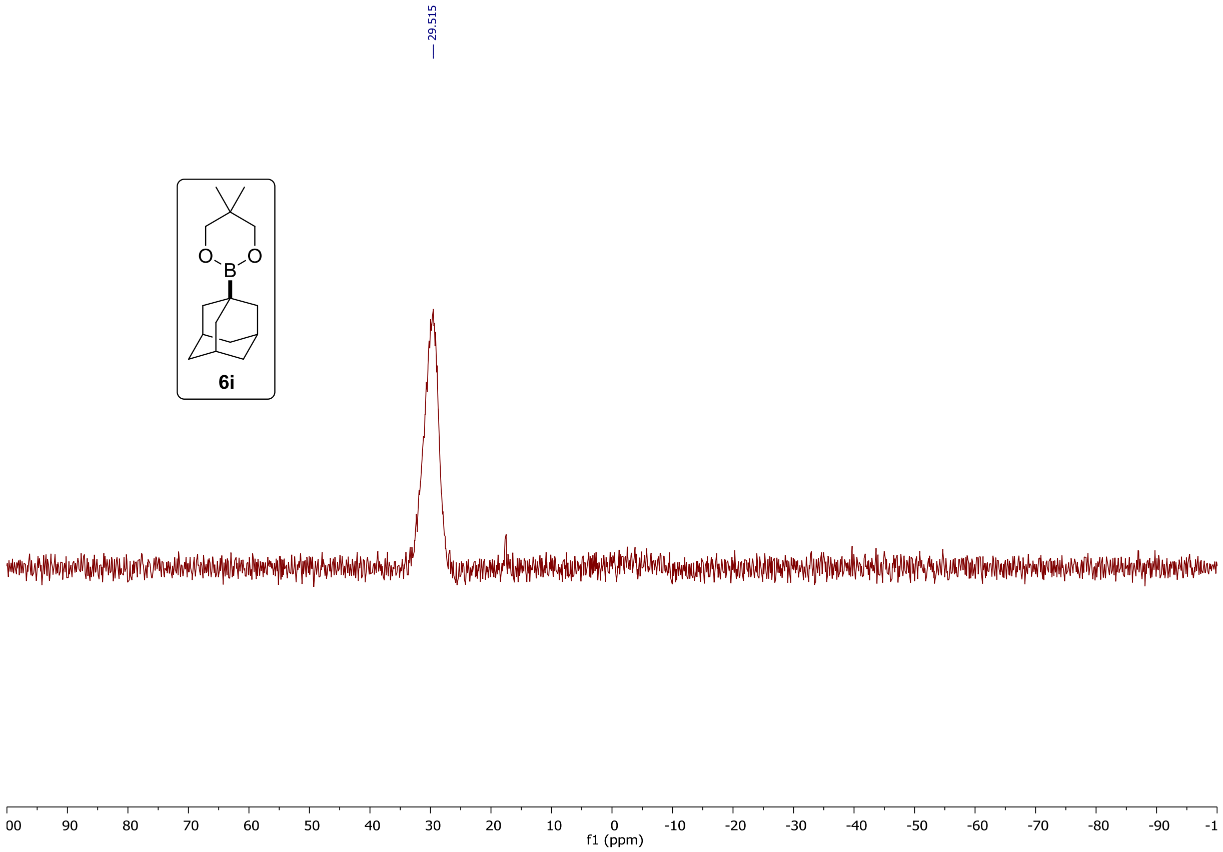
29.515
6i
00 90 80 70 60 50 40 30 20 10 0 -10 -20 -30 -40 -50 -60 -70 -80 -90 -1
f1 (ppm)

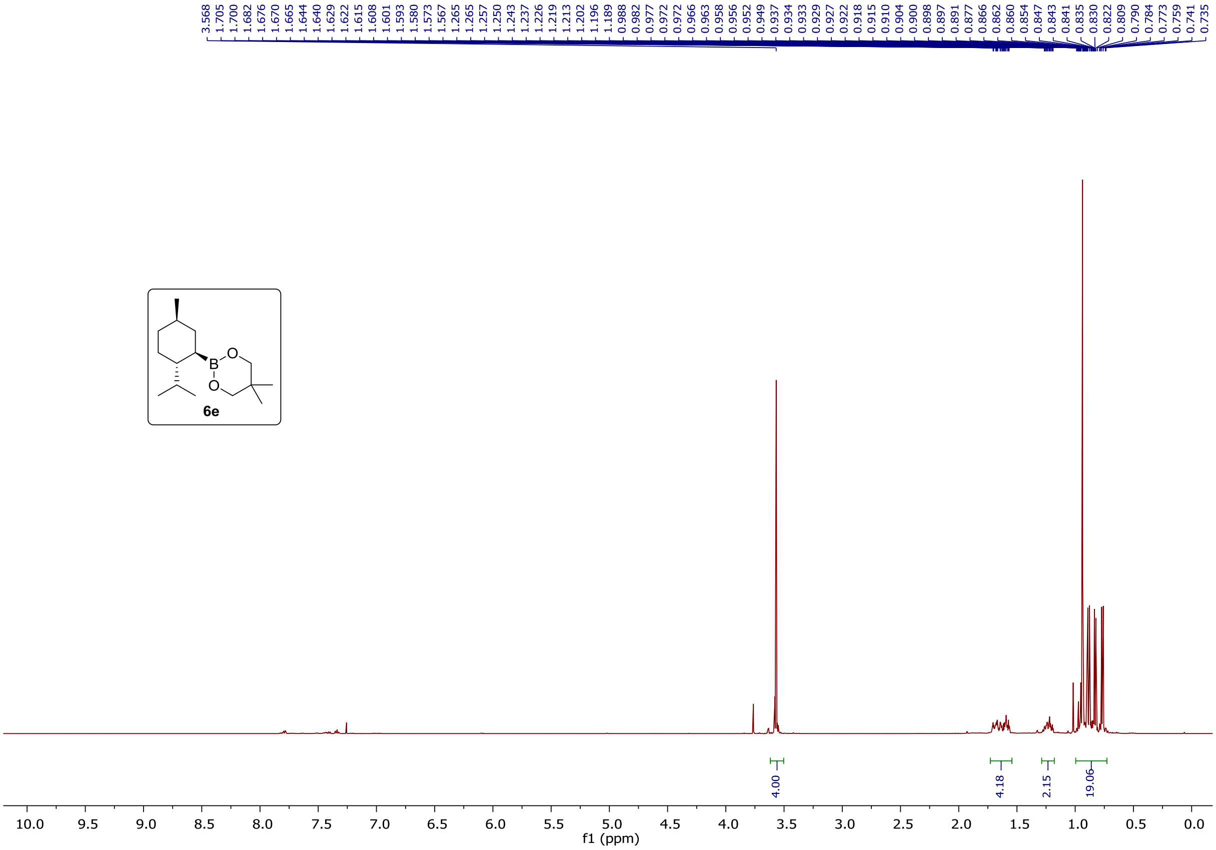
3.568 1.705 1.700 1.682 1.676 1.670 1.665 1.644 1.640 1.629 1.622 1.615 1.608 1.601 1.593 1.580 1.573 1.567 1.265 1.265 1.257 1.250 1.243 1.237 1.226 1.219 1.213 1.202 1.196 1.189 0.988 0.982 0.977 0.972 0.972 0.966 0.963 0.958 0.956 0.952 0.949 0.937 0.934 0.933 0.929 0.927 0.922 0.918 0.915 0.910 0.904 0.900 0.898 0.897 0.891 0.877 0.866 0.862 0.860 0.854 0.847 0.843 0.841 0.835 0.830 0.822 0.809 0.790 0.784 0.773 0.759 0.741 0.735
6e
4.00
4.18
2.15
19.06
10.0 9.5 9.0 8.5 8.0 7.5 7.0 6.5 6.0 5.5 5.0 4.5 4.0 3.5 3.0 2.5 2.0 1.5 1.0 0.5 0.0
f1 (ppm)

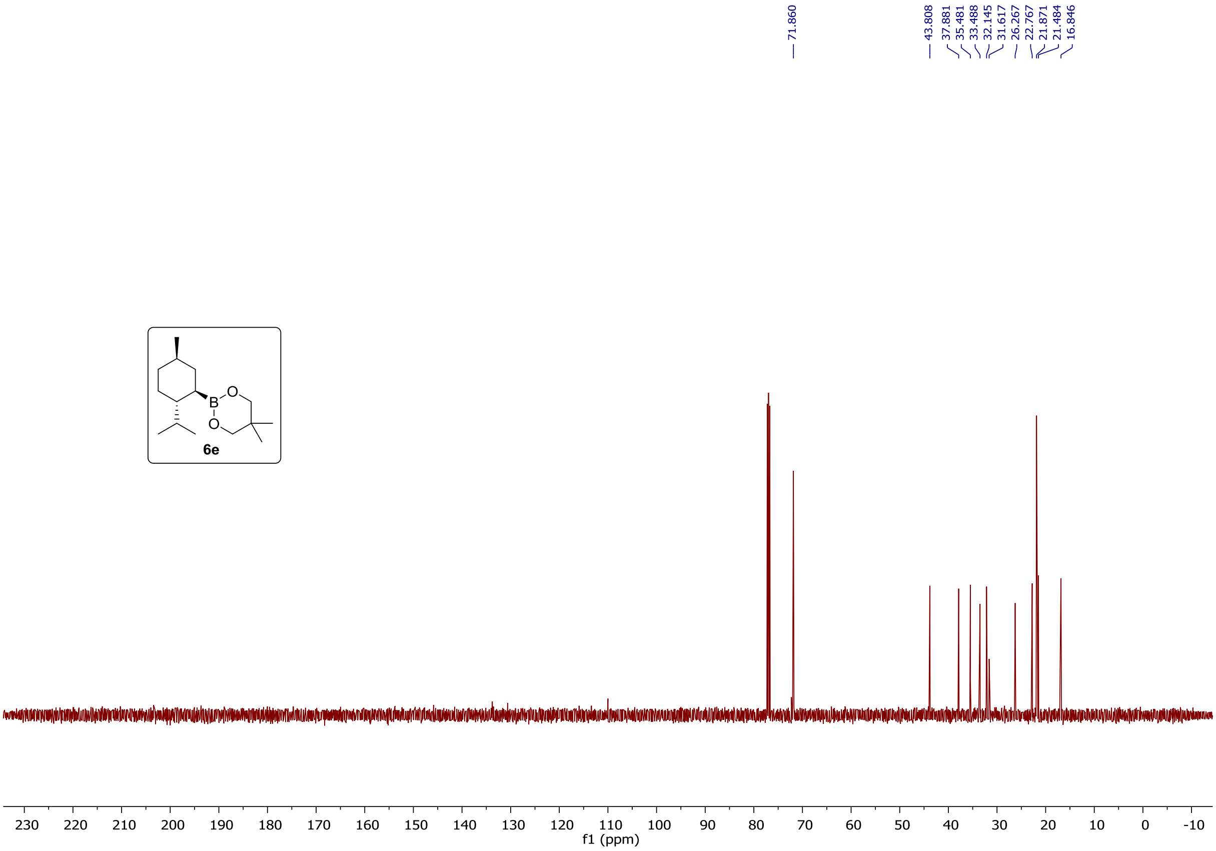
71.860
43.808 37.881 35.481 33.488 32.145 31.617 26.267 22.767 21.871 21.484 16.846
6e
230 220 210 200 190 180 170 160 150 140 130 120 110 100 90 80 70 60 50 40 30 20 10 0 -10
f1 (ppm)

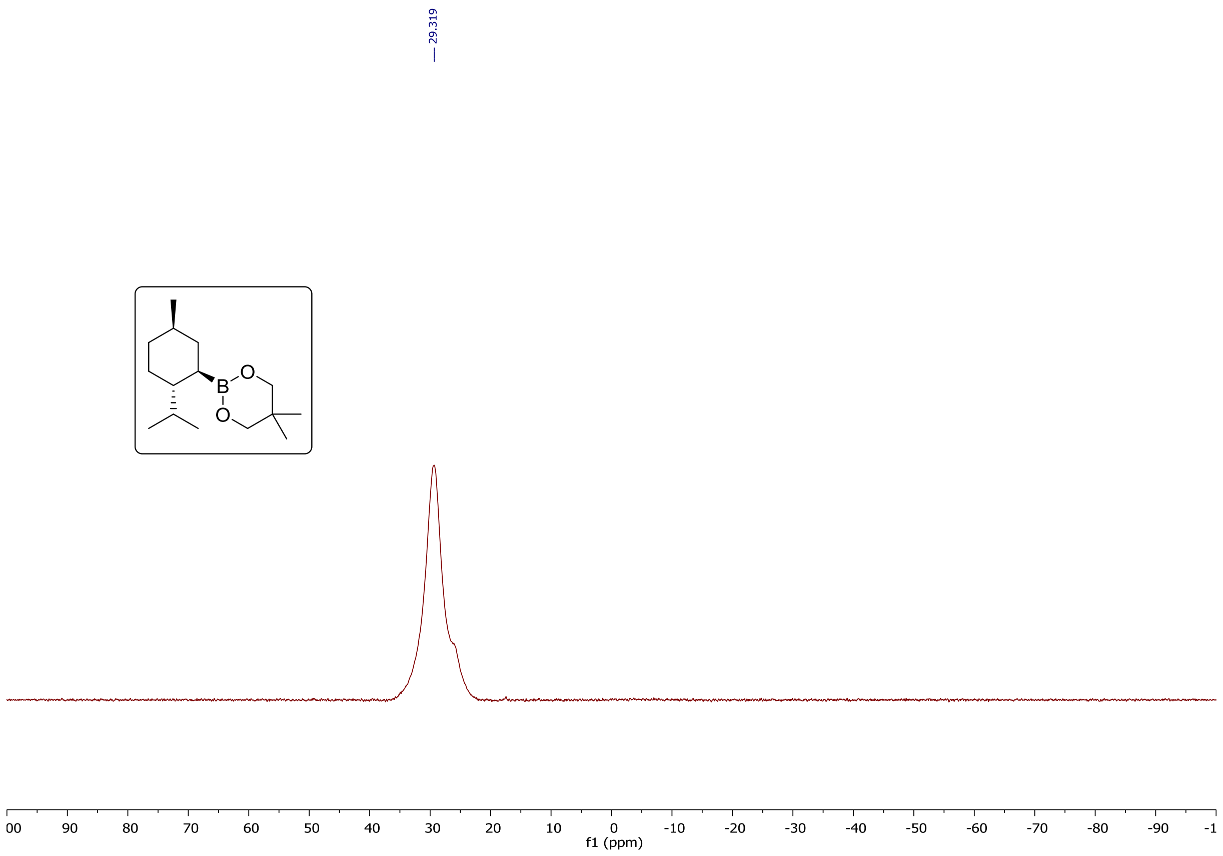
29.319
f1 (ppm)

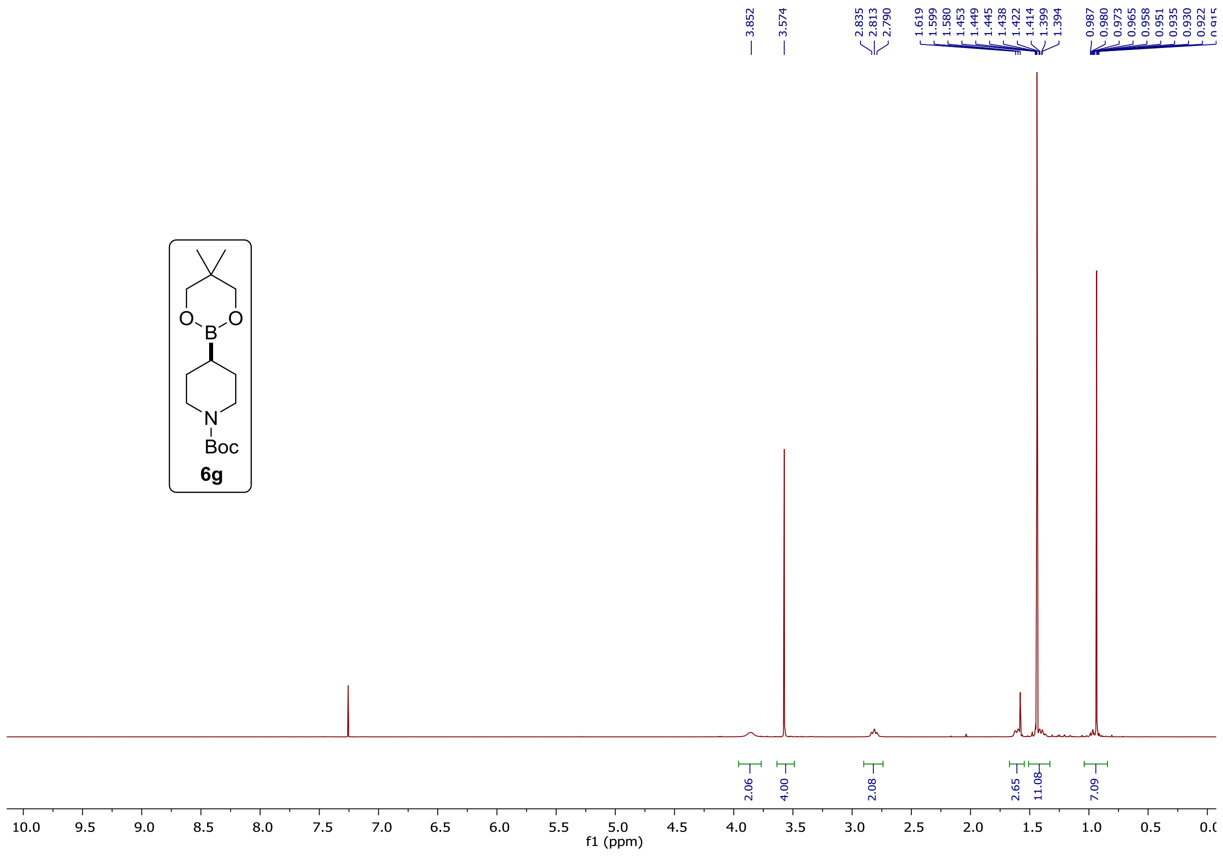
3.852
3.574
2.835
2.813
2.790
1.619
1.599
1.580
1.453
1.449
1.445
1.438
1.422
1.414
1.399
1.394
0.987
0.980
0.973
0.965
0.958
0.951
0.935
0.930
0.922
2.06
4.00
2.08
2.65
11.08
7.09
6g
f1 (ppm)

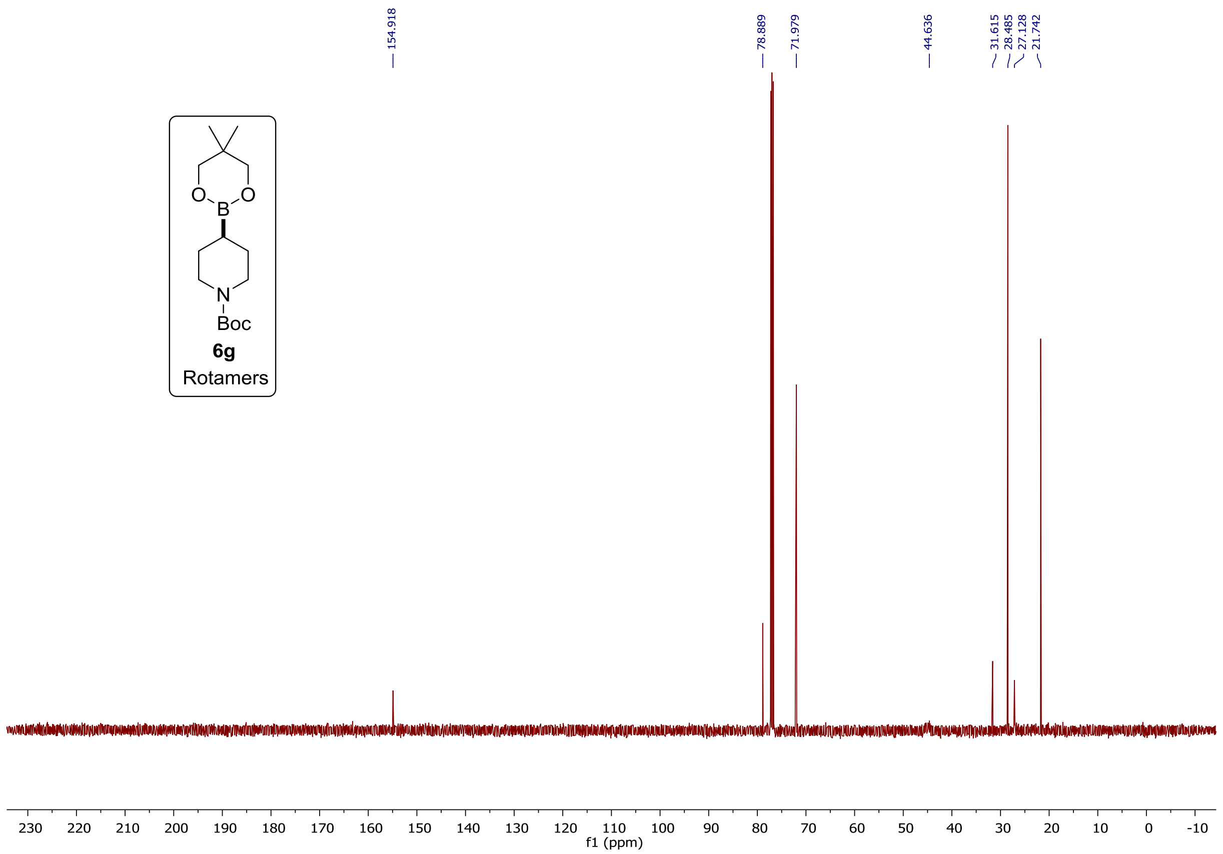

154.918
78.889
71.979
44.636
31.615
28.485
27.128
21.742
6g
Rotamers
230 220 210 200 190 180 170 160 150 140 130 120 110 100 90 80 70 60 50 40 30 20 10 0 -10
f1 (ppm)

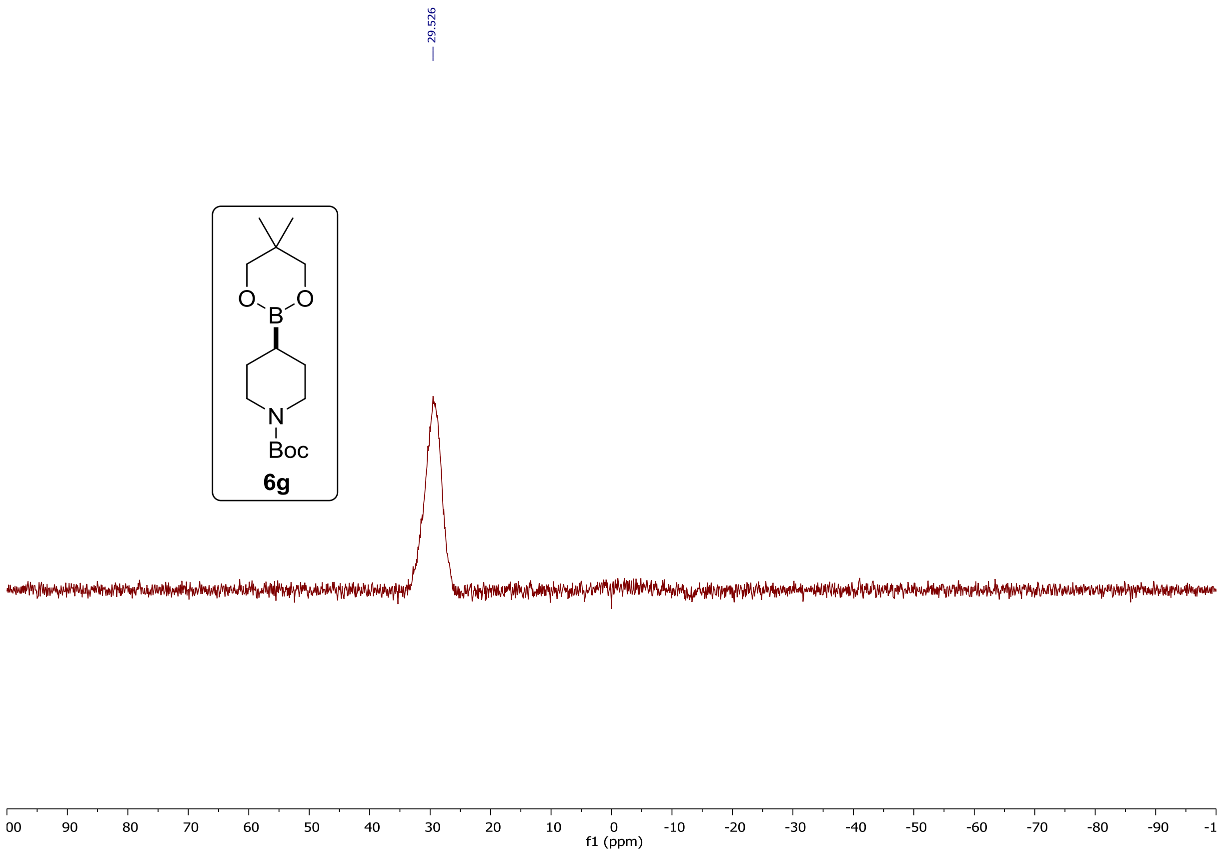

29.526
6g
00 90 80 70 60 50 40 30 20 10 0 -10 -20 -30 -40 -50 -60 -70 -80 -90 -1
f1 (ppm)

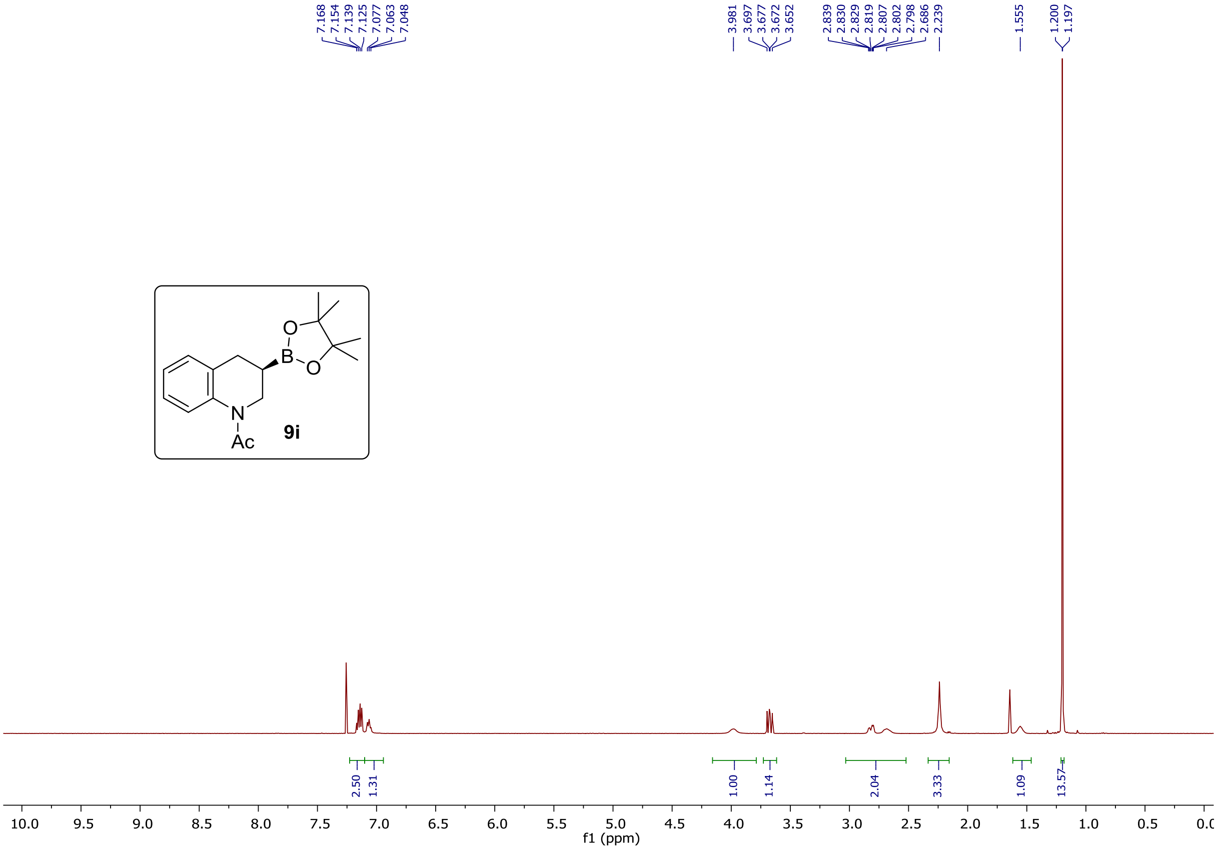
7.168
7.154
7.139
7.125
7.077
7.063
7.048
3.981
3.697
3.677
3.672
3.652
2.839
2.830
2.829
2.819
2.807
2.802
2.798
2.686
2.239
1.555
1.200
1.197
9i
Ac
2.50
1.31
1.00
1.14
2.04
3.33
1.09
13.57
f1 (ppm)

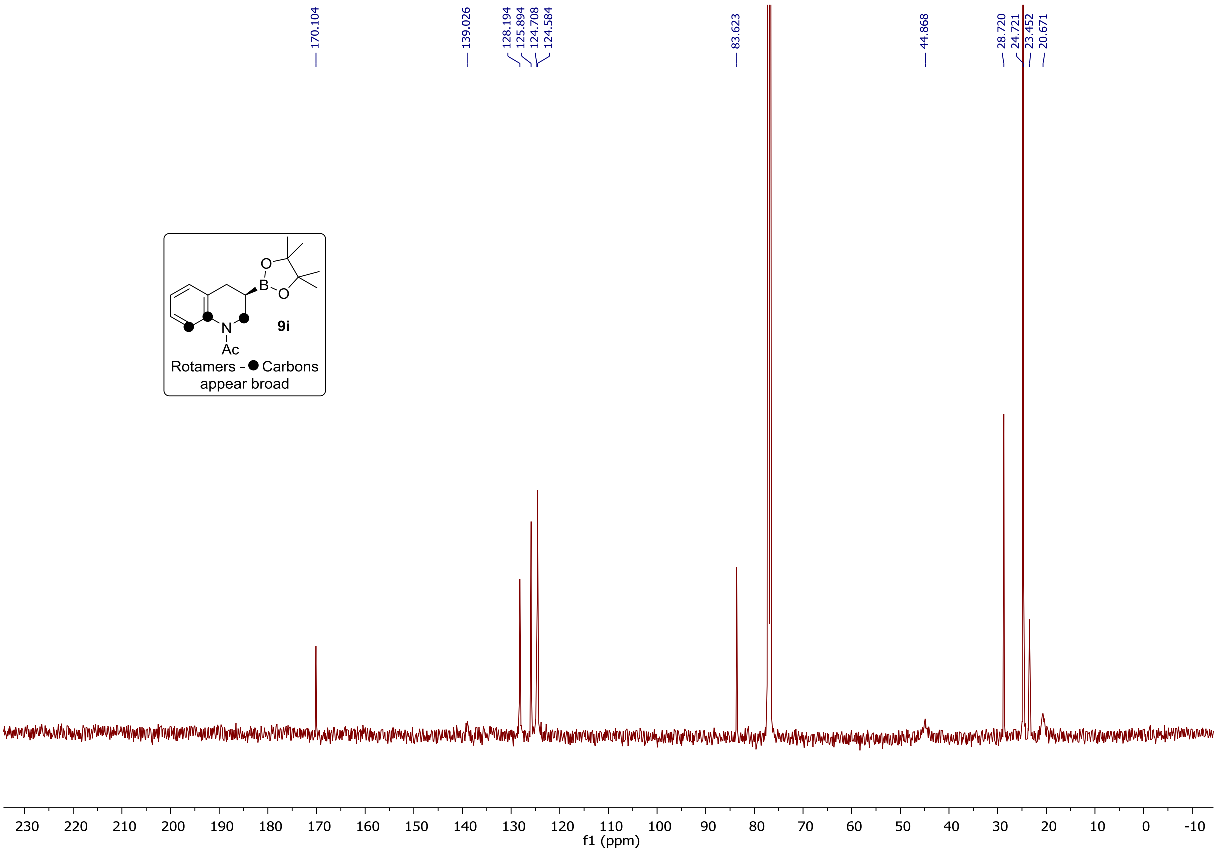
170.104
139.026
128.194
125.894
124.708
124.584
83.623
44.868
28.720
24.721
23.452
20.671
9i
Ac
Rotamers - ● Carbons
appear broad
f1 (ppm)

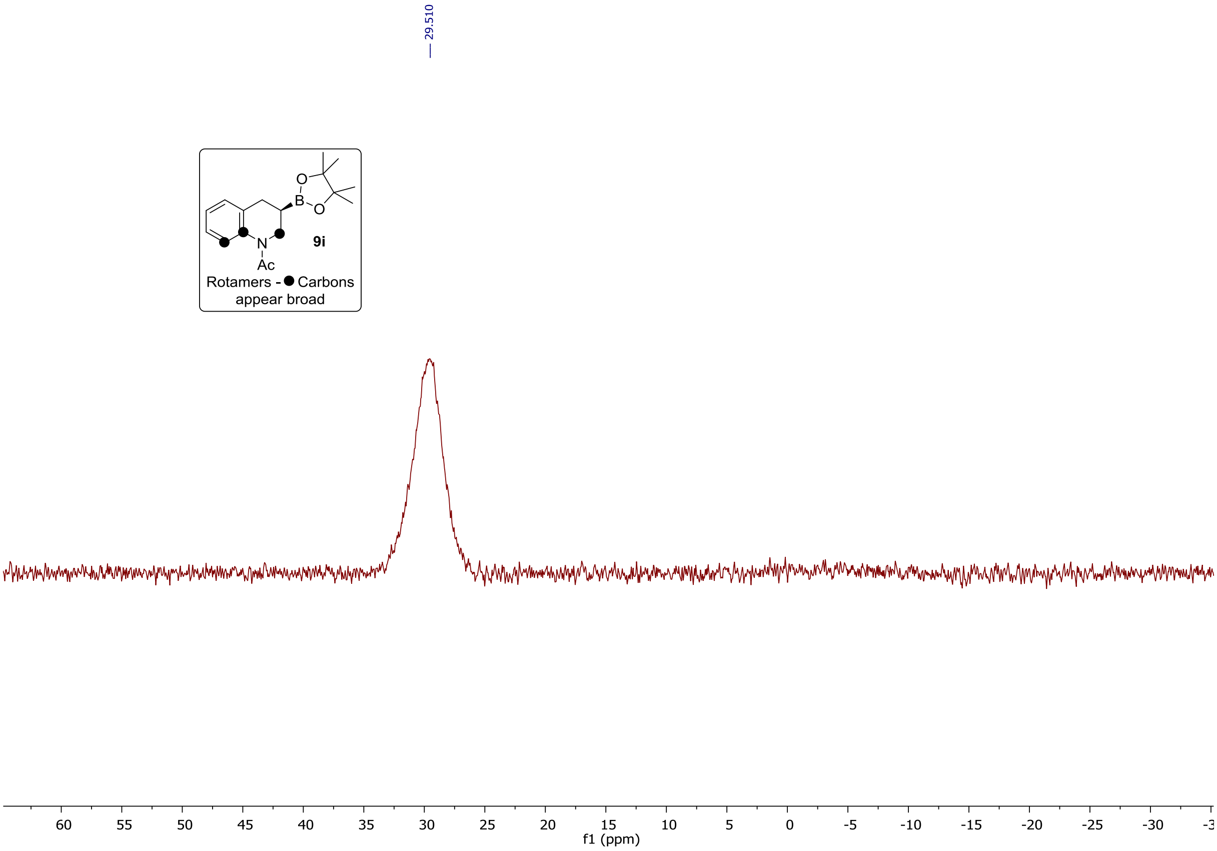
29.510
9i
N
Ac
Rotamers - ● Carbons appear broad
60
55
50
45
40
35
30
25
20
15
10
5
0
-5
-10
-15
-20
-25
-30
f1 (ppm)

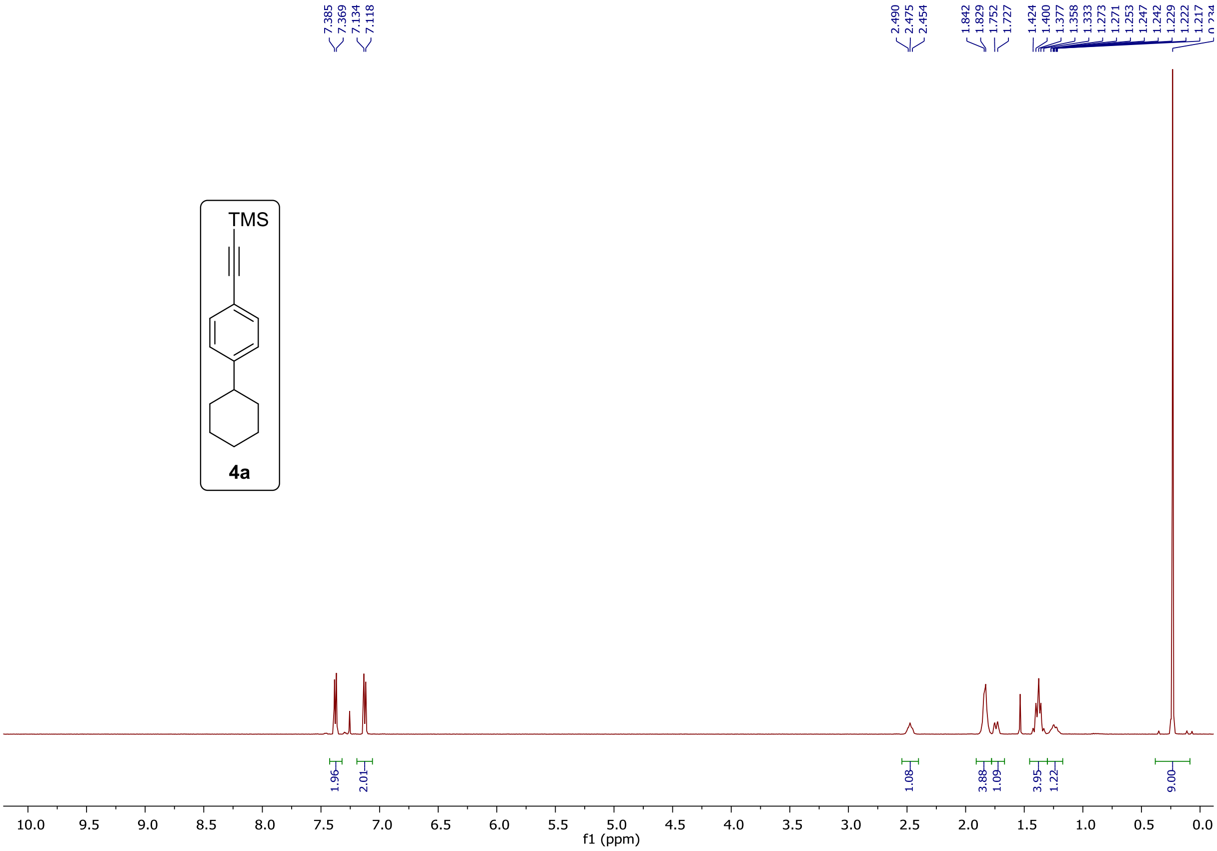
7.385
7.369
7.134
7.118
2.490
2.475
2.454
1.842
1.829
1.752
1.727
1.424
1.400
1.377
1.358
1.333
1.273
1.271
1.253
1.247
1.242
1.229
1.222
1.217
TMS
4a
1.96
2.01
1.08
3.88
1.09
3.95
1.22
9.00
10.0
9.5
9.0
8.5
8.0
7.5
7.0
6.5
6.0
5.5
5.0
4.5
4.0
3.5
3.0
2.5
2.0
1.5
1.0
0.5
0.0
f1 (ppm)

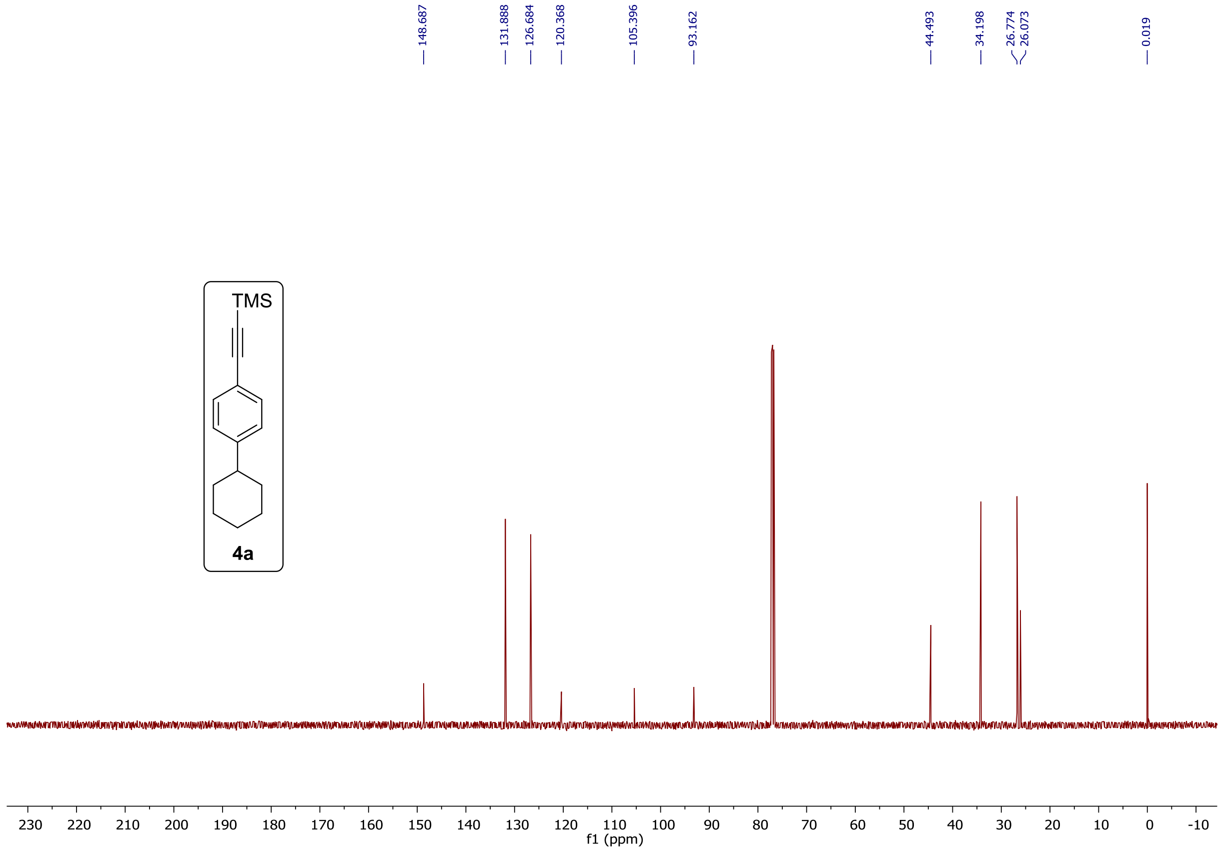
148.687
131.888
126.684
120.368
105.396
93.162
44.493
34.198
26.774
26.073
0.019
TMS
4a
230 220 210 200 190 180 170 160 150 140 130 120 110 100 90 80 70 60 50 40 30 20 10 0 -10
f1 (ppm)

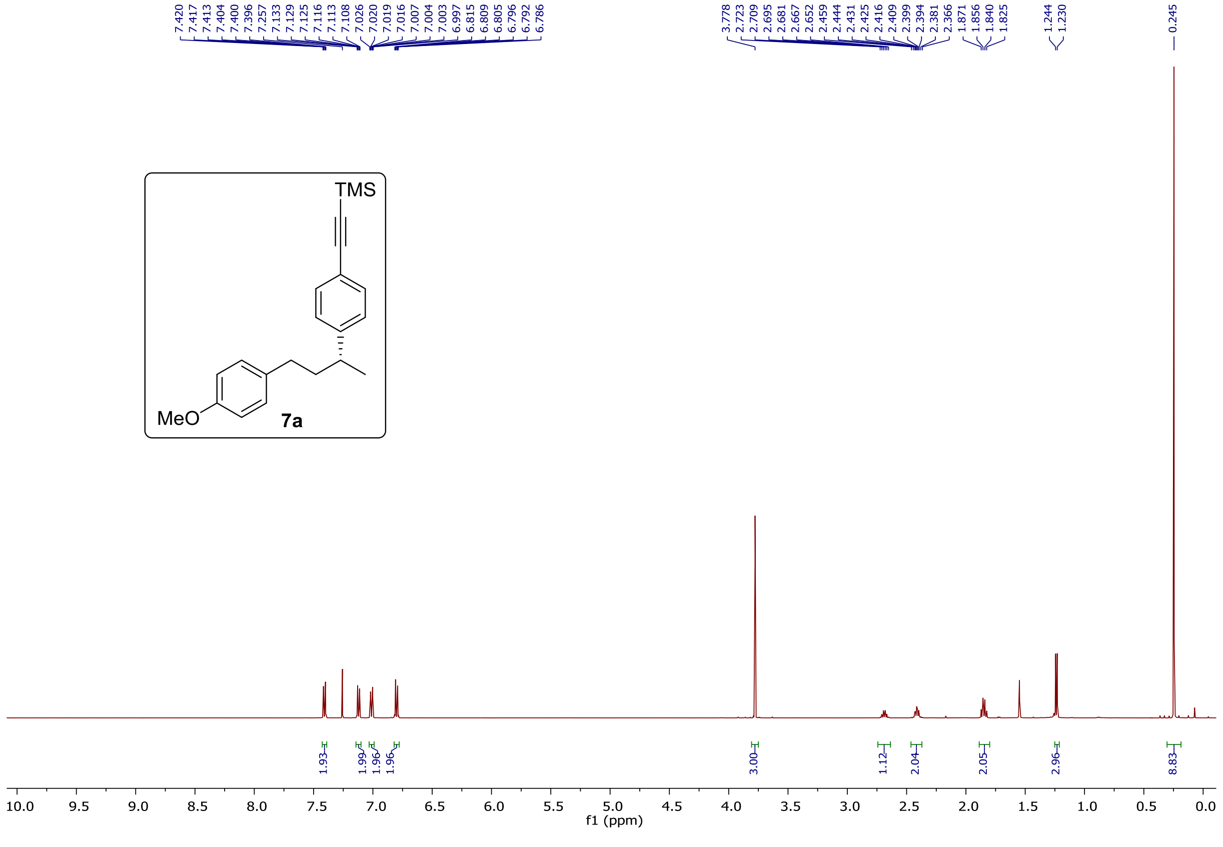
7.420
7.417
7.413
7.404
7.400
7.396
7.257
7.133
7.129
7.125
7.116
7.113
7.108
7.026
7.020
7.019
7.016
7.007
7.004
7.003
6.997
6.815
6.809
6.805
6.796
6.792
6.786
3.778
2.723
2.709
2.695
2.681
2.667
2.652
2.459
2.444
2.431
2.425
2.416
2.409
2.399
2.394
2.381
2.366
1.871
1.856
1.840
1.825
1.244
1.230
0.245
TMS
MeO
7a
1.93
1.99
1.96
1.96
3.00
1.12
2.04
2.05
2.96
8.83
10.0 9.5 9.0 8.5 8.0 7.5 7.0 6.5 6.0 5.5 5.0 4.5 4.0 3.5 3.0 2.5 2.0 1.5 1.0 0.5 0.0
f1 (ppm)

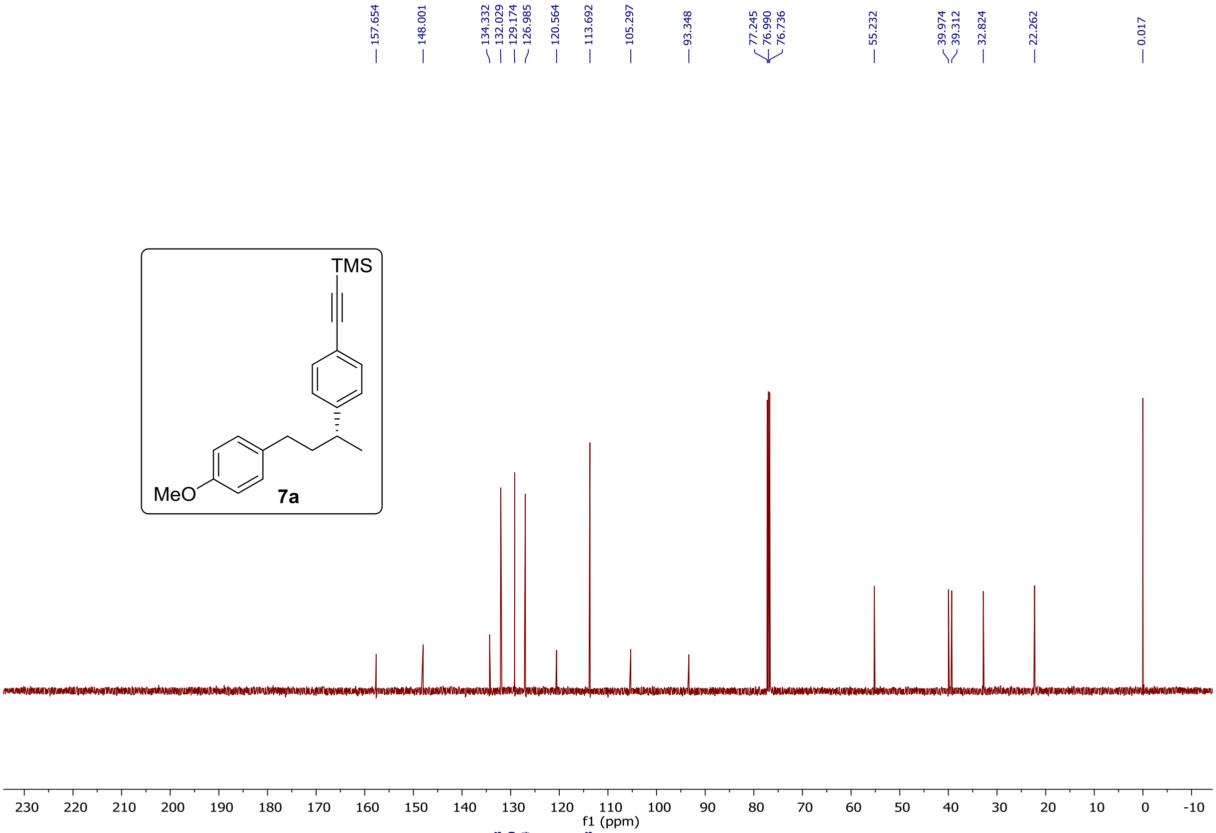
157.654
148.001
134.332
132.029
129.174
126.985
120.564
113.692
105.297
93.348
77.245
76.990
76.736
55.232
39.974
39.312
32.824
22.262
0.017
TMS
MeO
7a
230
220
210
200
190
180
170
160
150
140
130
120
110
100
90
80
70
60
50
40
30
20
10
0
-10
f1 (ppm)

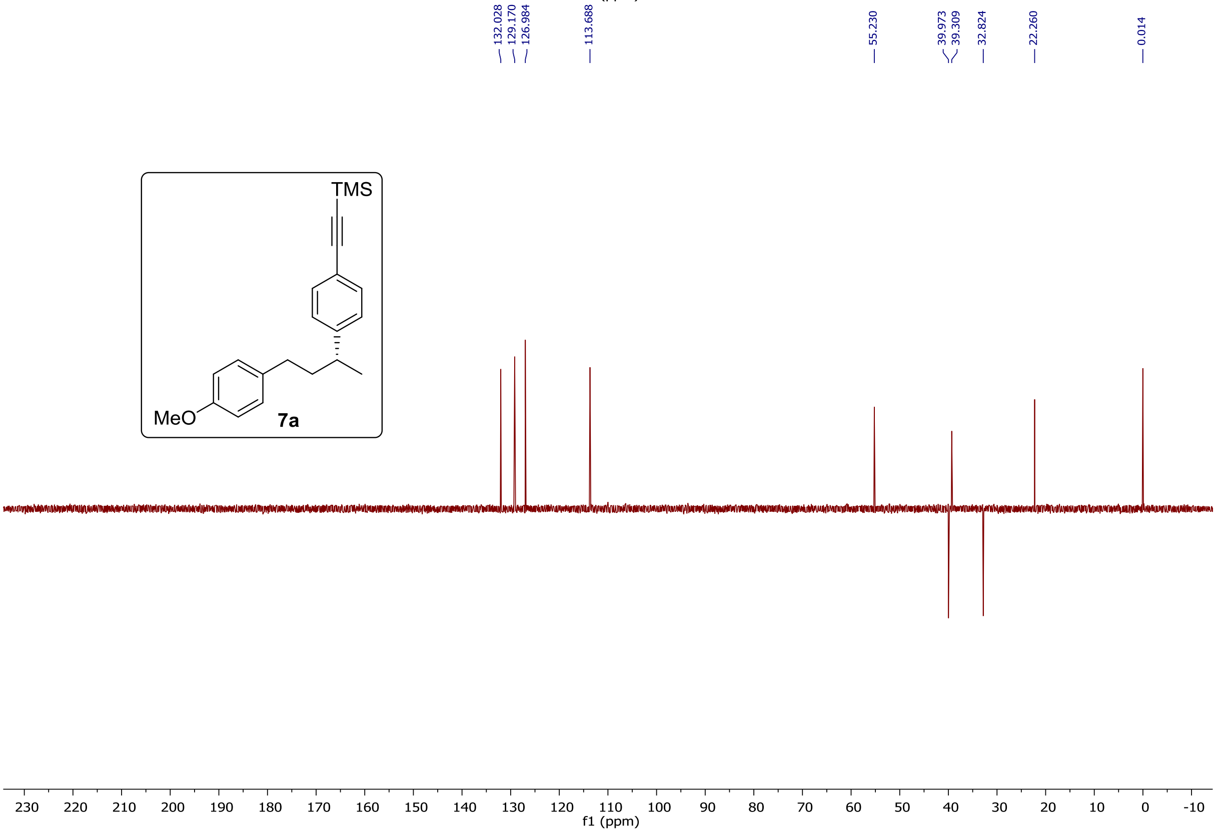
132.028
129.170
126.984
113.688
55.230
39.973
39.309
32.824
22.260
0.014
TMS
MeO
7a
230
220
210
200
190
180
170
160
150
140
130
120
110
100
90
80
70
60
50
40
30
20
10
0
-10
f1 (ppm)

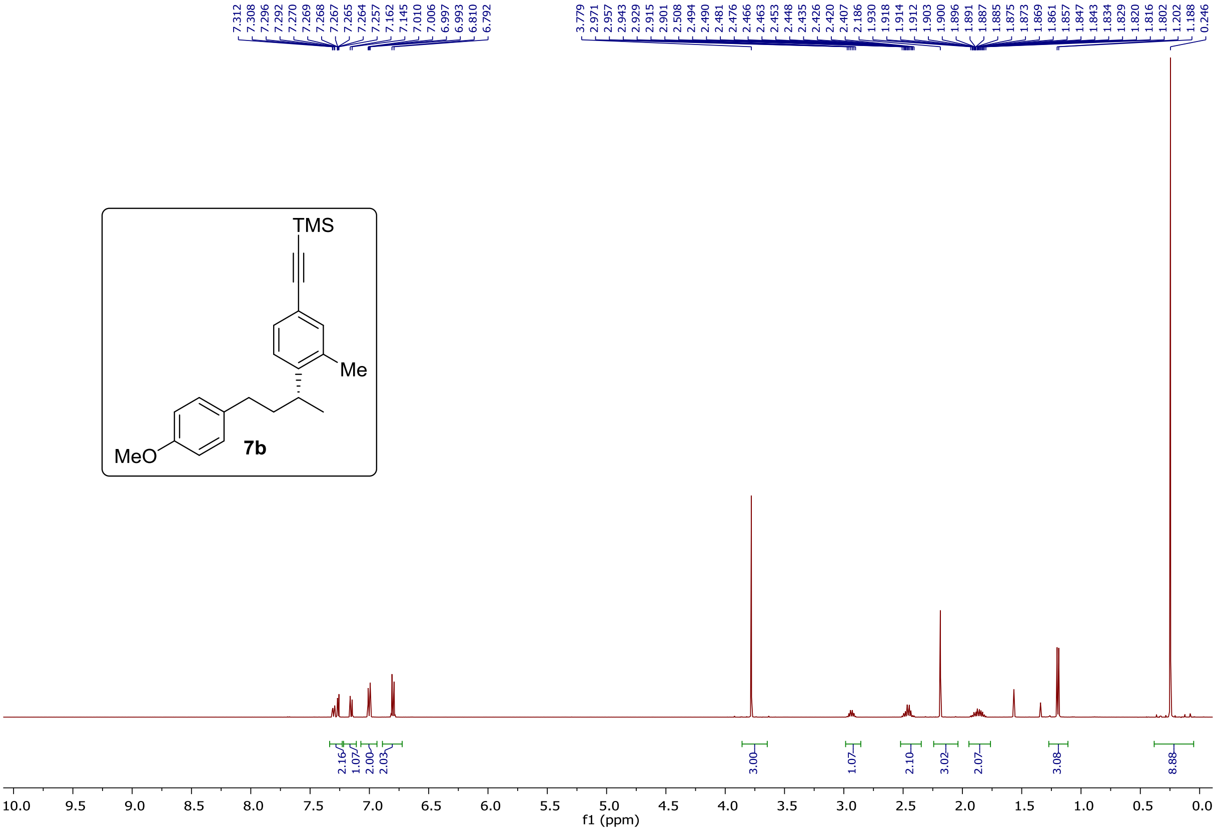
7.312 7.308 7.296 7.292 7.270 7.269 7.268 7.267 7.265 7.264 7.257 7.162 7.145 7.010 7.006 6.997 6.993 6.810 6.792
3.779 2.971 2.957 2.943 2.929 2.915 2.901 2.508 2.494 2.490 2.481 2.476 2.466 2.463 2.453 2.448 2.435 2.426 2.420 2.407 2.186 1.930 1.918 1.914 1.912 1.903 1.900 1.896 1.891 1.887 1.885 1.875 1.873 1.869 1.861 1.857 1.847 1.843 1.834 1.829 1.820 1.816 1.802 1.202 1.188 0.246
TMS
Me
MeO
7b
2.16 1.07 2.00 2.03 3.00 1.07 2.10 3.02 2.07 3.08 8.88
f1 (ppm)

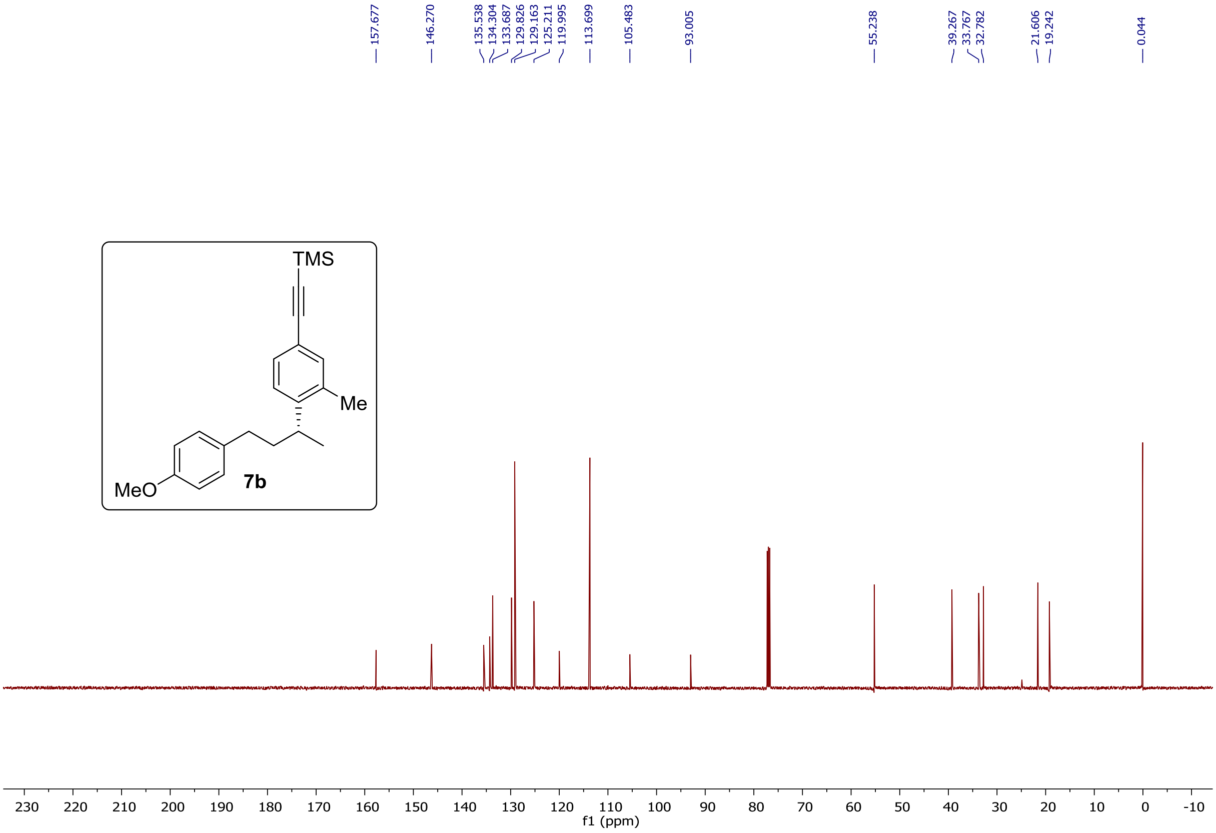
157.677 146.270 135.538 134.304 133.687 129.826 129.163 125.211 119.995 113.699 105.483 93.005 55.238 39.267 33.767 32.782 21.606 19.242 0.044
TMS
Me
MeO
7b
f1 (ppm)

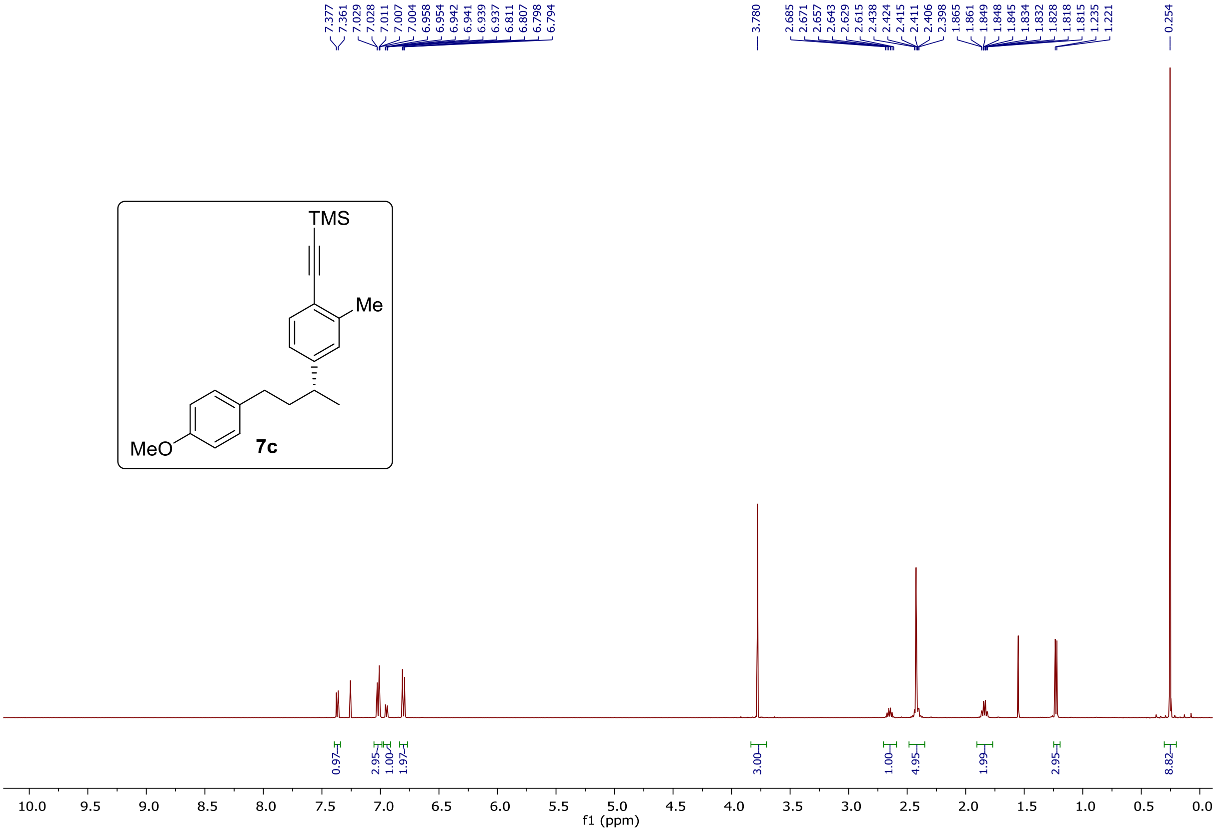
7.377
7.361
7.029
7.028
7.011
7.007
7.004
6.958
6.954
6.942
6.941
6.939
6.937
6.811
6.807
6.798
6.794
3.780
2.685
2.671
2.657
2.643
2.629
2.615
2.438
2.424
2.415
2.411
2.406
2.398
1.865
1.861
1.849
1.848
1.845
1.834
1.832
1.828
1.818
1.815
1.235
1.221
0.254
TMS
Me
MeO
7c
0.97
2.95
1.00
1.97
3.00
1.00
4.95
1.99
2.95
8.82
10.0 9.5 9.0 8.5 8.0 7.5 7.0 6.5 6.0 5.5 5.0 4.5 4.0 3.5 3.0 2.5 2.0 1.5 1.0 0.5 0.0
f1 (ppm)

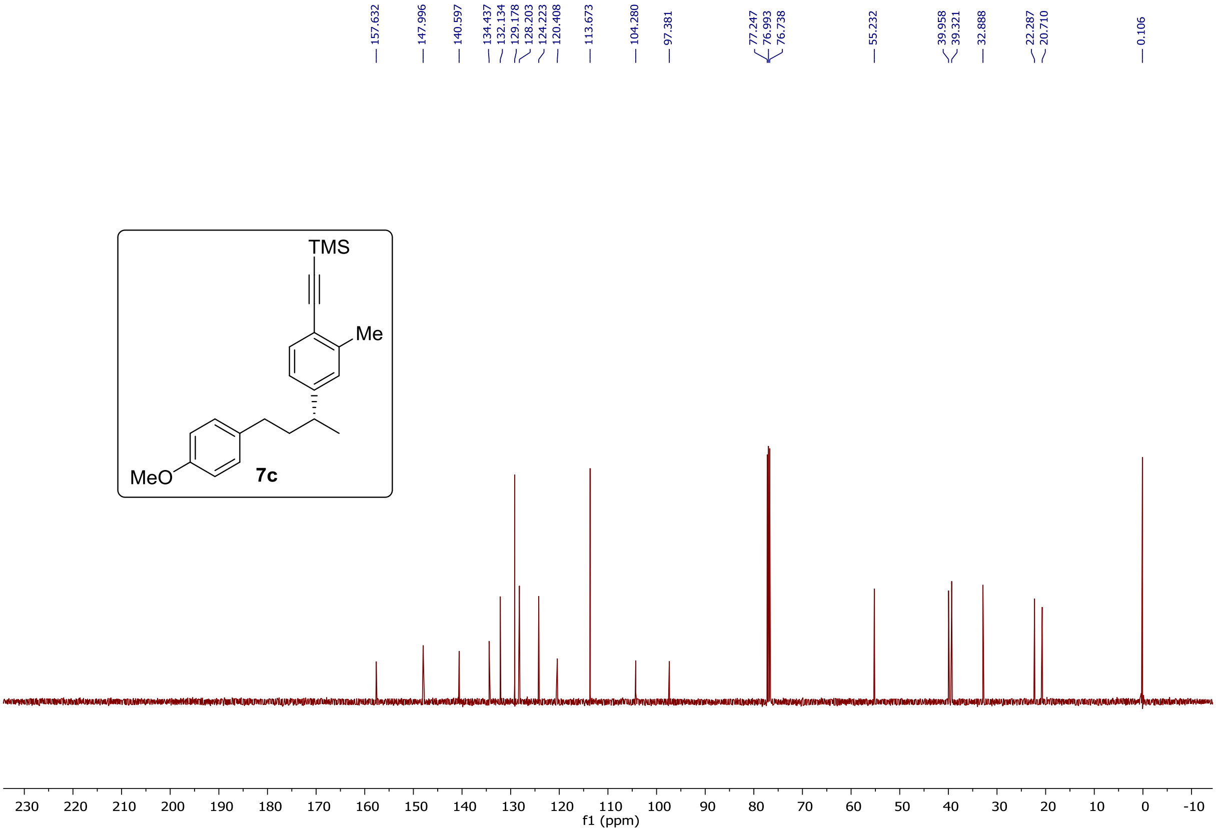
157.632
147.996
140.597
134.437
132.134
129.178
128.203
124.223
120.408
113.673
104.280
97.381
77.247
76.993
76.738
55.232
39.958
39.321
32.888
22.287
20.710
0.106
TMS
Me
MeO
7c
230 220 210 200 190 180 170 160 150 140 130 120 110 100 90 80 70 60 50 40 30 20 10 0 -10
f1 (ppm)

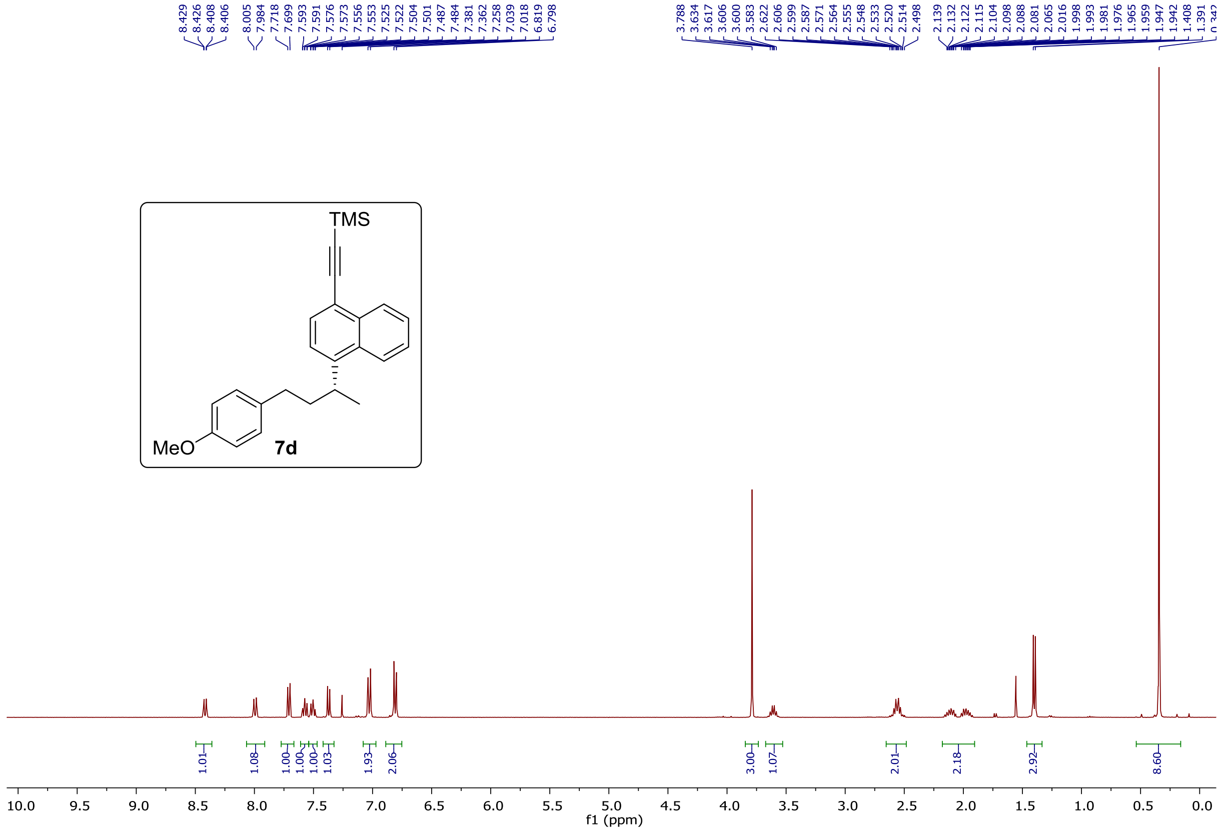
TMS
MeO
7d
f1 (ppm)

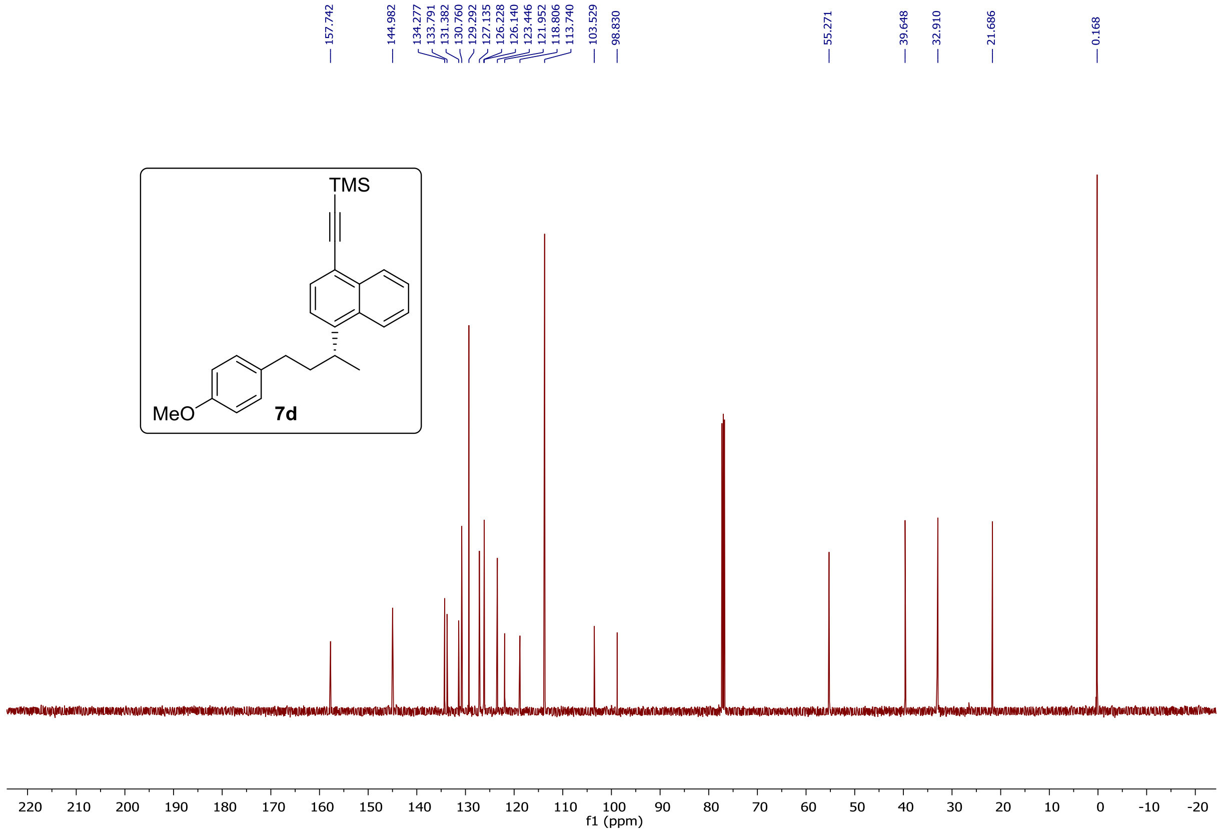
157.742
144.982
134.277
133.791
131.382
130.760
129.292
127.135
126.228
126.140
123.446
121.952
118.806
113.740
103.529
98.830
55.271
39.648
32.910
21.686
0.168
TMS
MeO
7d
f1 (ppm)

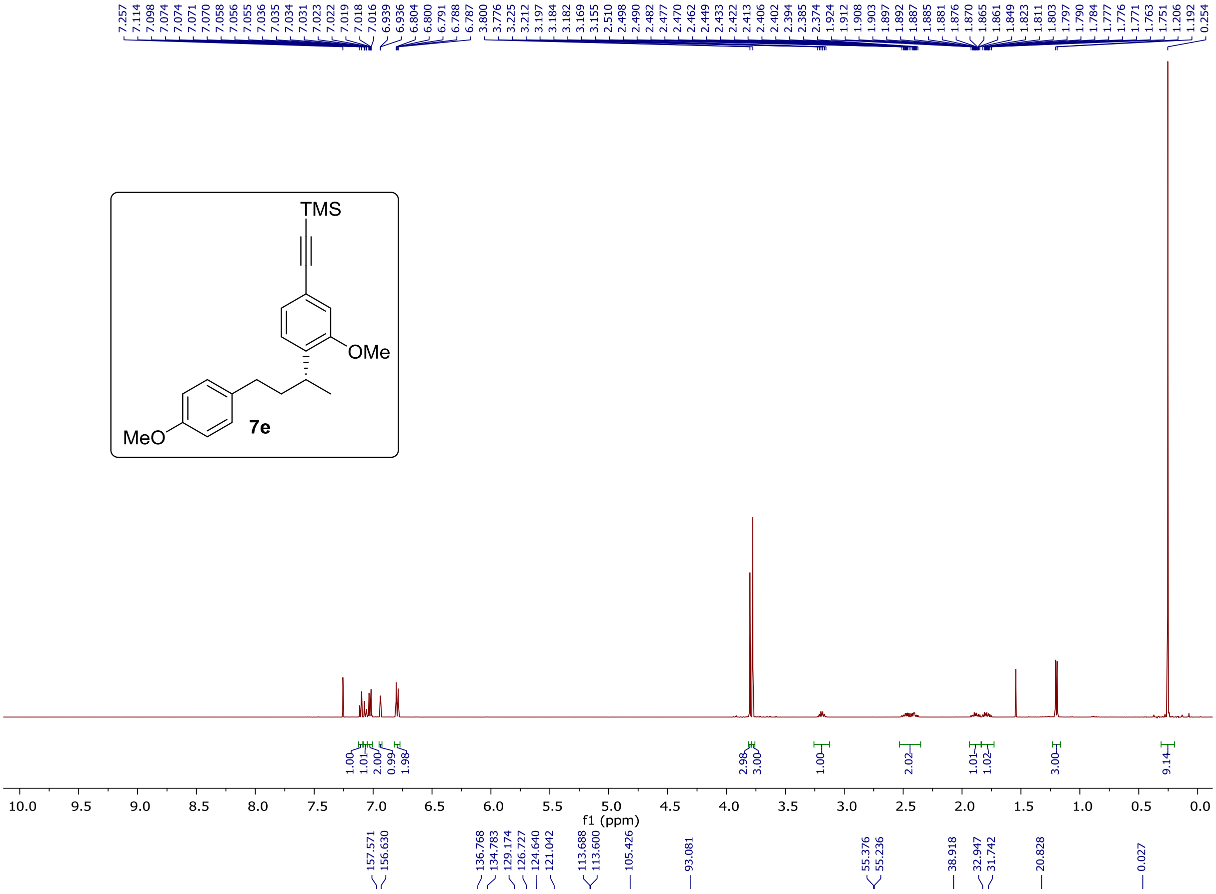
7.257
7.114
7.098
7.074
7.074
7.071
7.070
7.058
7.056
7.055
7.036
7.035
7.034
7.031
7.023
7.022
7.019
7.018
7.016
6.939
6.936
6.804
6.800
6.791
6.788
6.787
3.800
3.776
3.225
3.212
3.197
3.184
3.182
3.169
3.155
2.510
2.498
2.490
2.482
2.477
2.470
2.462
2.449
2.433
2.422
2.413
2.406
2.402
2.394
2.385
2.374
1.924
1.912
1.908
1.903
1.897
1.892
1.887
1.885
1.881
1.876
1.870
1.865
1.861
1.849
1.823
1.811
1.803
1.797
1.790
1.784
1.777
1.776
1.771
1.763
1.751
1.206
1.192
0.254
TMS
OMe
MeO
7e
1.00
1.01
2.00
0.99
1.98
2.98
3.00
1.00
2.02
1.01
1.02
3.00
9.14
10.0 9.5 9.0 8.5 8.0 7.5 7.0 6.5 6.0 5.5 5.0 4.5 4.0 3.5 3.0 2.5 2.0 1.5 1.0 0.5 0.0
f1 (ppm)

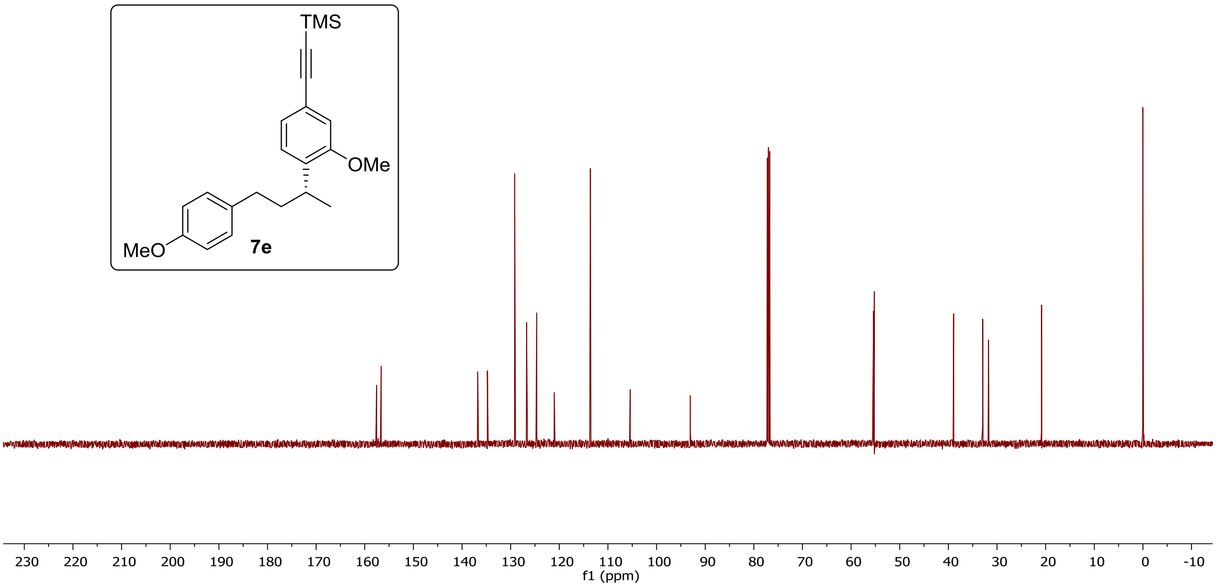
157.571
156.630
136.768
134.783
129.174
126.727
124.640
121.042
113.688
113.600
105.426
93.081
55.376
55.236
38.918
32.947
31.742
20.828
0.027
TMS
OMe
MeO
7e
230 220 210 200 190 180 170 160 150 140 130 120 110 100 90 80 70 60 50 40 30 20 10 0 -10
f1 (ppm)

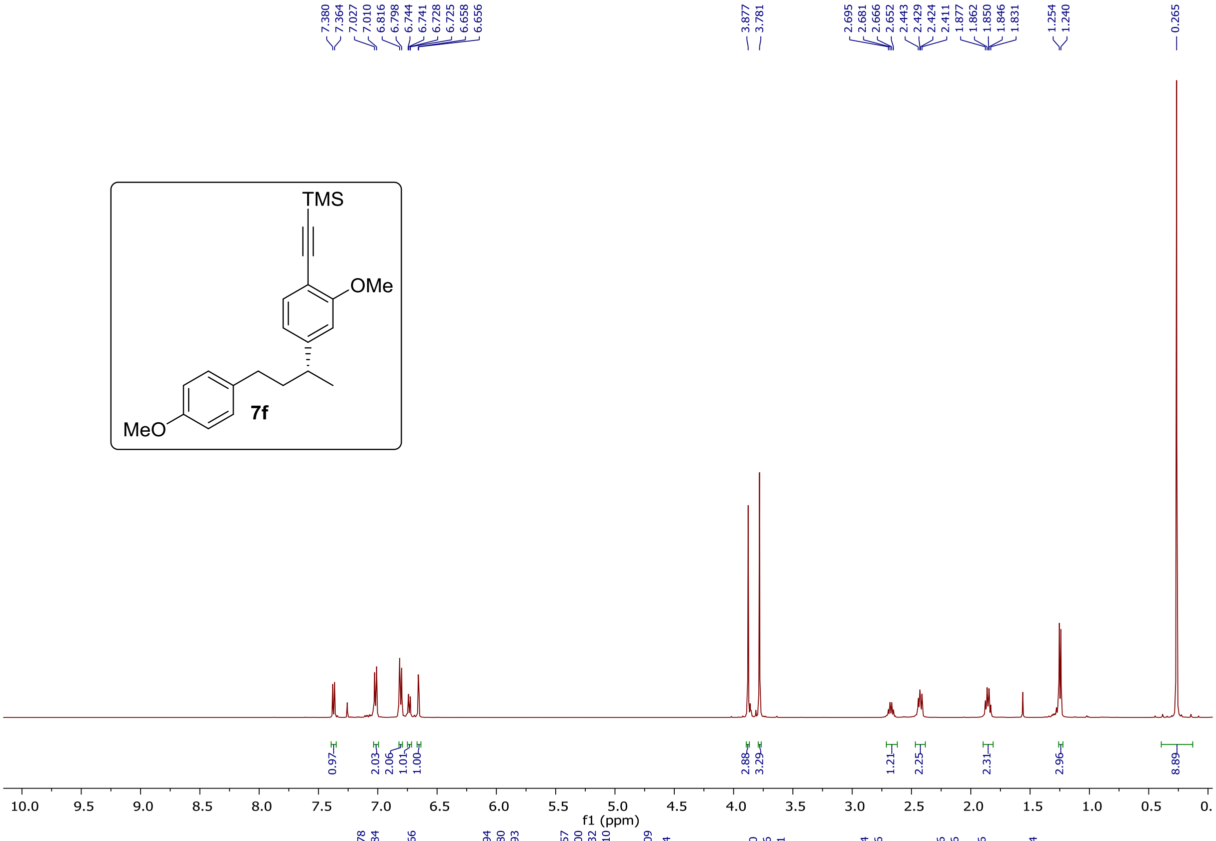
7.380
7.364
7.027
7.010
6.816
6.798
6.744
6.741
6.728
6.725
6.658
6.656
3.877
3.781
2.695
2.681
2.666
2.652
2.443
2.429
2.424
2.411
1.877
1.862
1.850
1.846
1.831
1.254
1.240
0.265
TMS
OMe
MeO
7f
0.97
2.03
2.06
1.01
1.00
2.88
3.29
1.21
2.25
2.31
2.96
8.89
f1 (ppm)

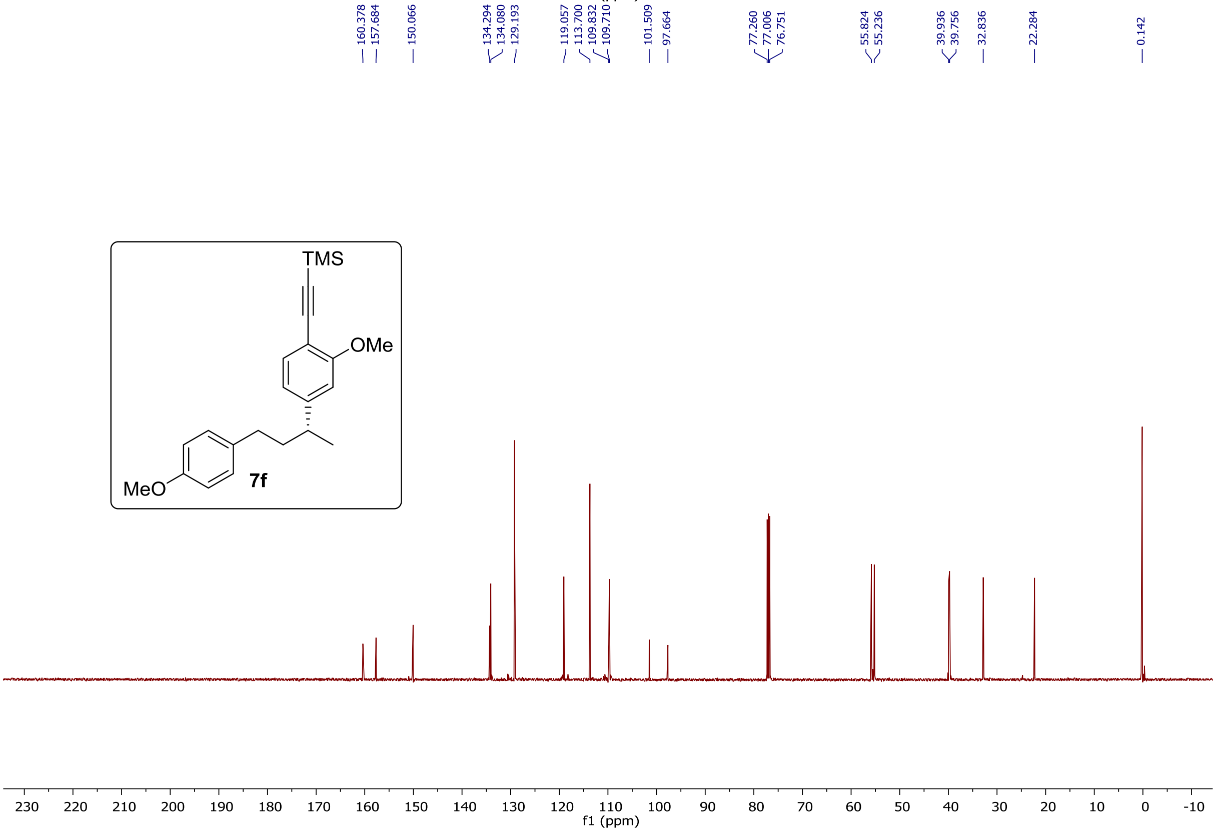
160.378
157.684
150.066
134.294
134.080
129.193
119.057
113.700
109.832
109.710
101.509
97.664
77.260
77.006
76.751
55.824
55.236
39.936
39.756
32.836
22.284
0.142
TMS
OMe
MeO
7f
f1 (ppm)

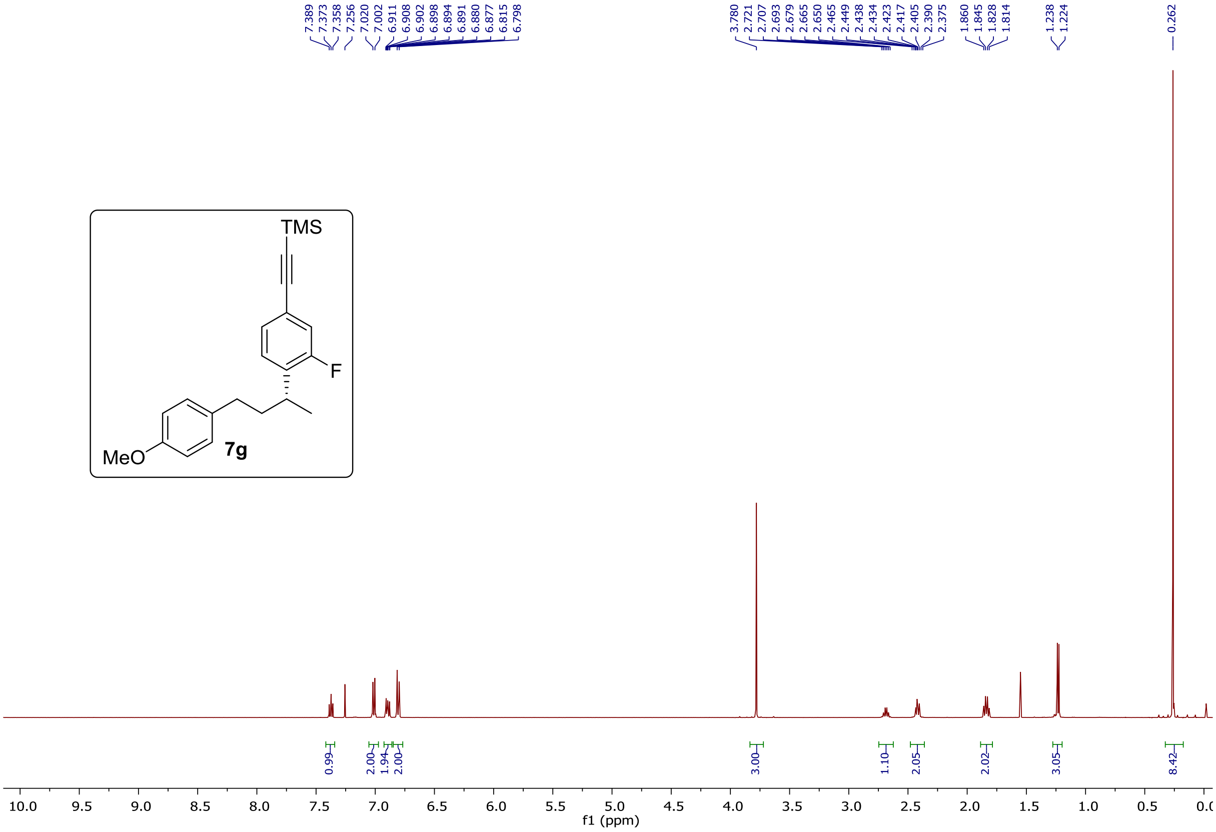
7.389
7.373
7.358
7.256
7.020
7.002
6.911
6.908
6.902
6.898
6.894
6.891
6.880
6.877
6.815
6.798
3.780
2.721
2.707
2.693
2.679
2.665
2.650
2.465
2.449
2.438
2.434
2.423
2.417
2.405
2.390
2.375
1.860
1.845
1.828
1.814
1.238
1.224
0.262
TMS
F
MeO
7g
0.99
2.00
1.94
2.00
3.00
1.10
2.05
2.02
3.05
8.42
10.0 9.5 9.0 8.5 8.0 7.5 7.0 6.5 6.0 5.5 5.0 4.5 4.0 3.5 3.0 2.5 2.0 1.5 1.0 0.5
f1 (ppm)

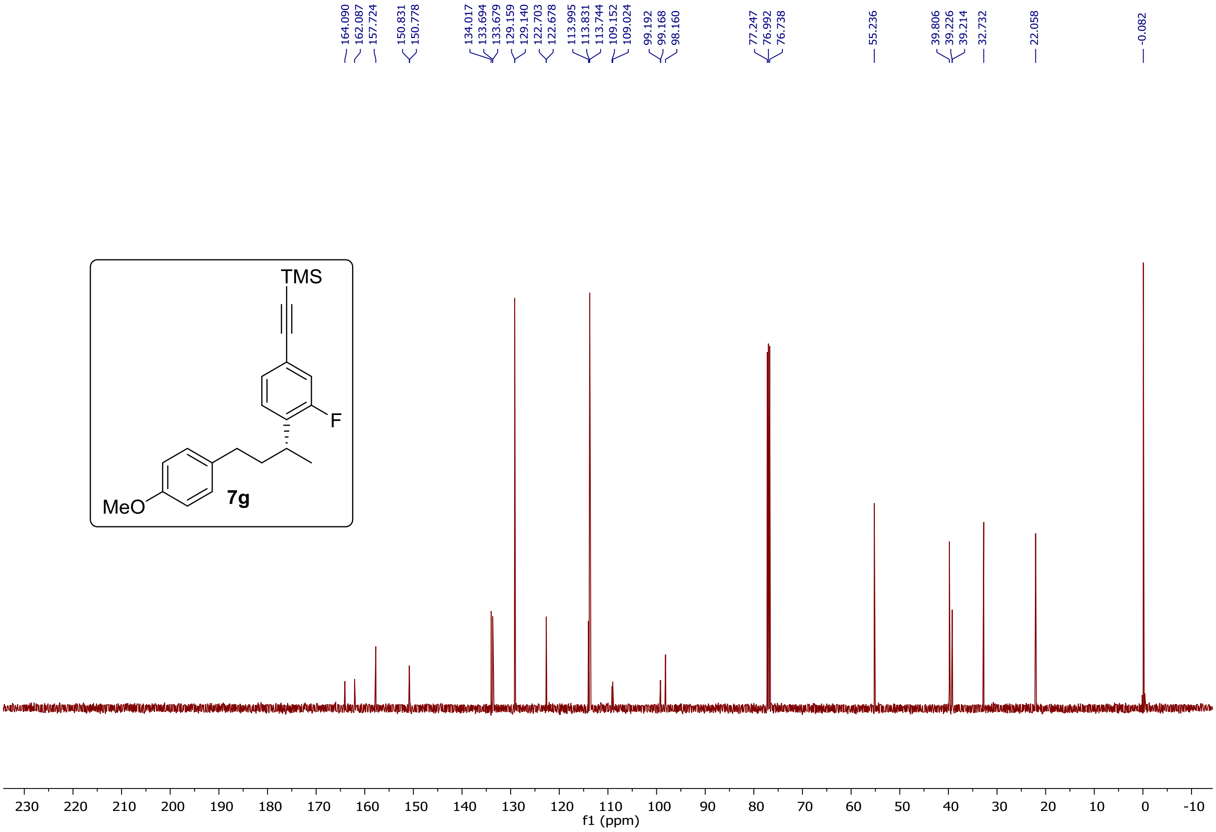
164.090
162.087
157.724
150.831
150.778
134.017
133.694
133.679
129.159
129.140
122.703
122.678
113.995
113.831
113.744
109.152
109.024
99.192
99.168
98.160
77.247
76.992
76.738
55.236
39.806
39.226
39.214
32.732
22.058
-0.082
TMS
F
MeO
7g
230 220 210 200 190 180 170 160 150 140 130 120 110 100 90 80 70 60 50 40 30 20 10 0 -10
f1 (ppm)

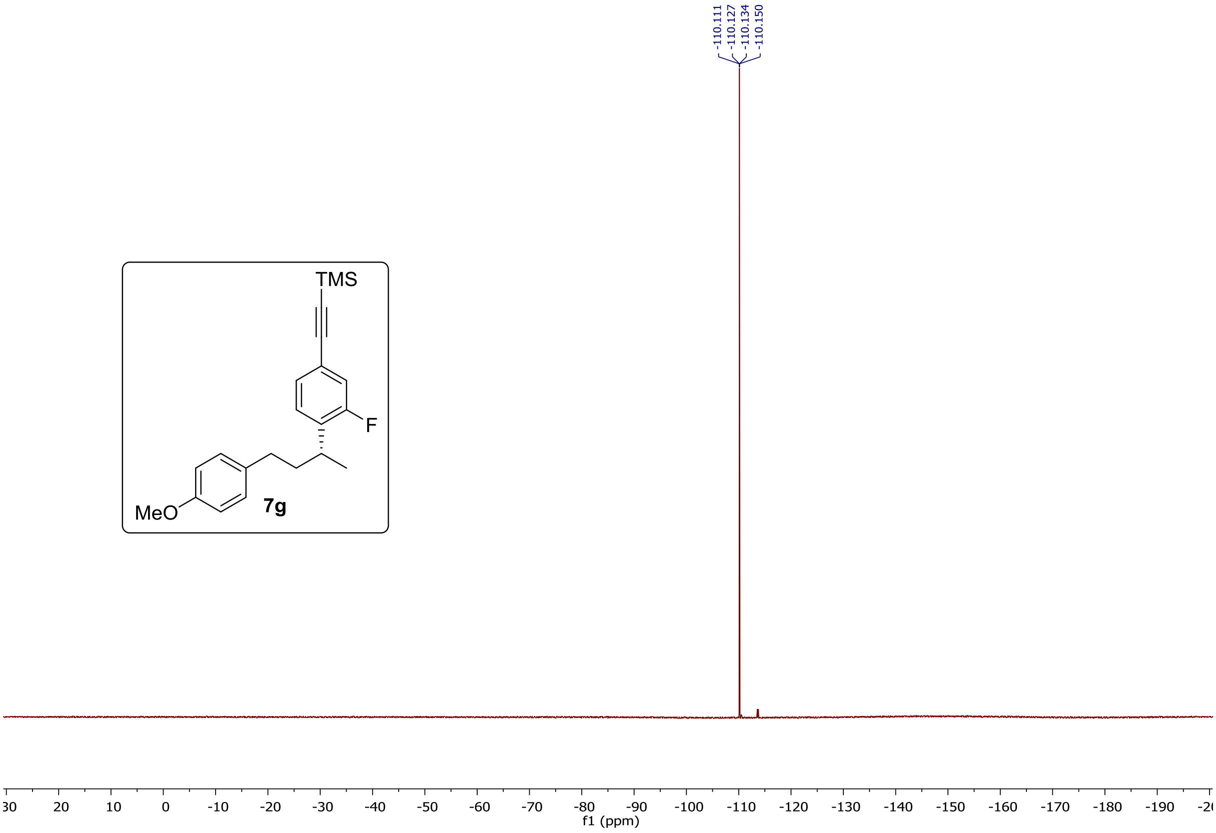
-110.111
-110.127
-110.134
-110.150
TMS
F
MeO
7g
f1 (ppm)

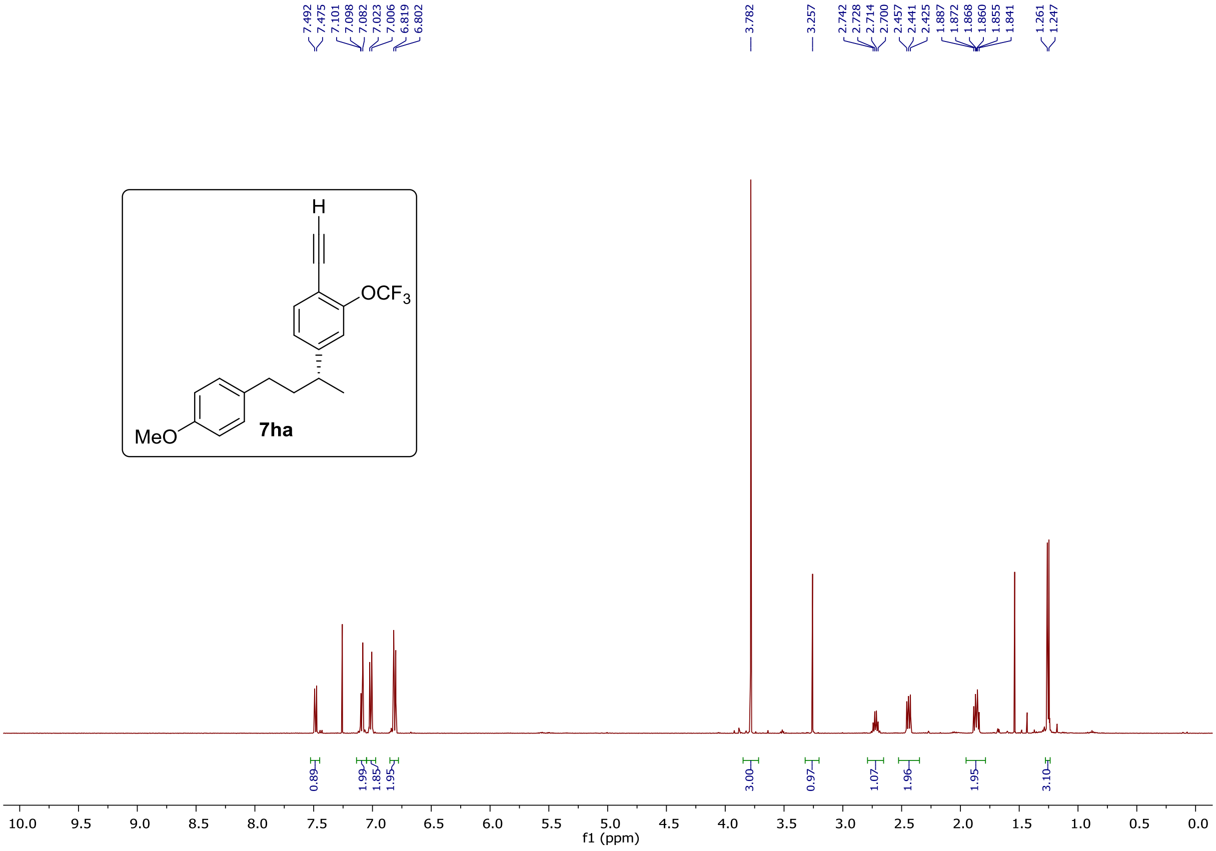
7.492
7.475
7.101
7.098
7.082
7.023
7.006
6.819
6.802
3.782
3.257
2.742
2.728
2.714
2.700
2.457
2.441
2.425
1.887
1.872
1.868
1.860
1.855
1.841
1.261
1.247
H
OCF3
MeO
7ha
0.89
1.99
1.85
1.95
3.00
0.97
1.07
1.96
1.95
3.10
f1 (ppm)

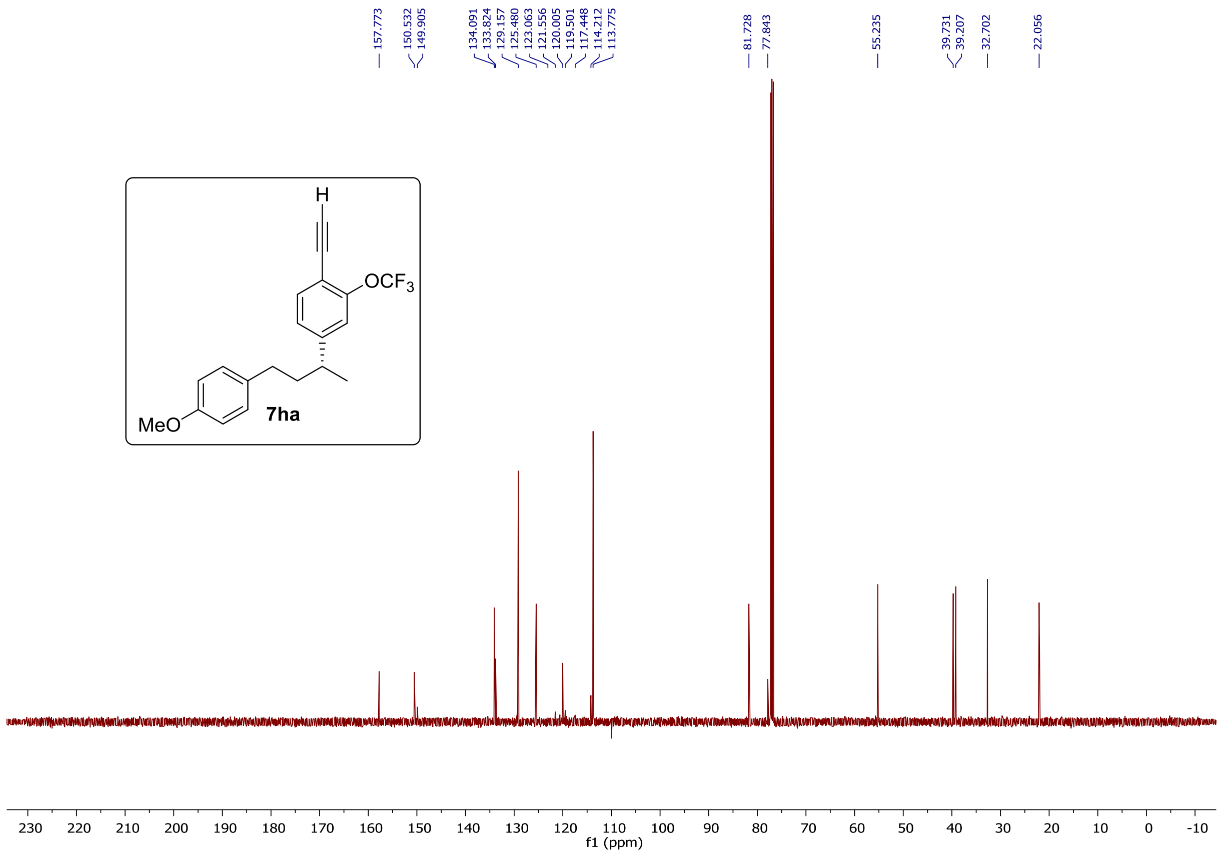
157.773
150.532
149.905
134.091
133.824
129.157
125.480
123.063
121.556
120.005
119.501
117.448
114.212
113.775
81.728
77.843
55.235
39.731
39.207
32.702
22.056
H
OCF3
MeO
7ha
230 220 210 200 190 180 170 160 150 140 130 120 110 100 90 80 70 60 50 40 30 20 10 0 -10
f1 (ppm)

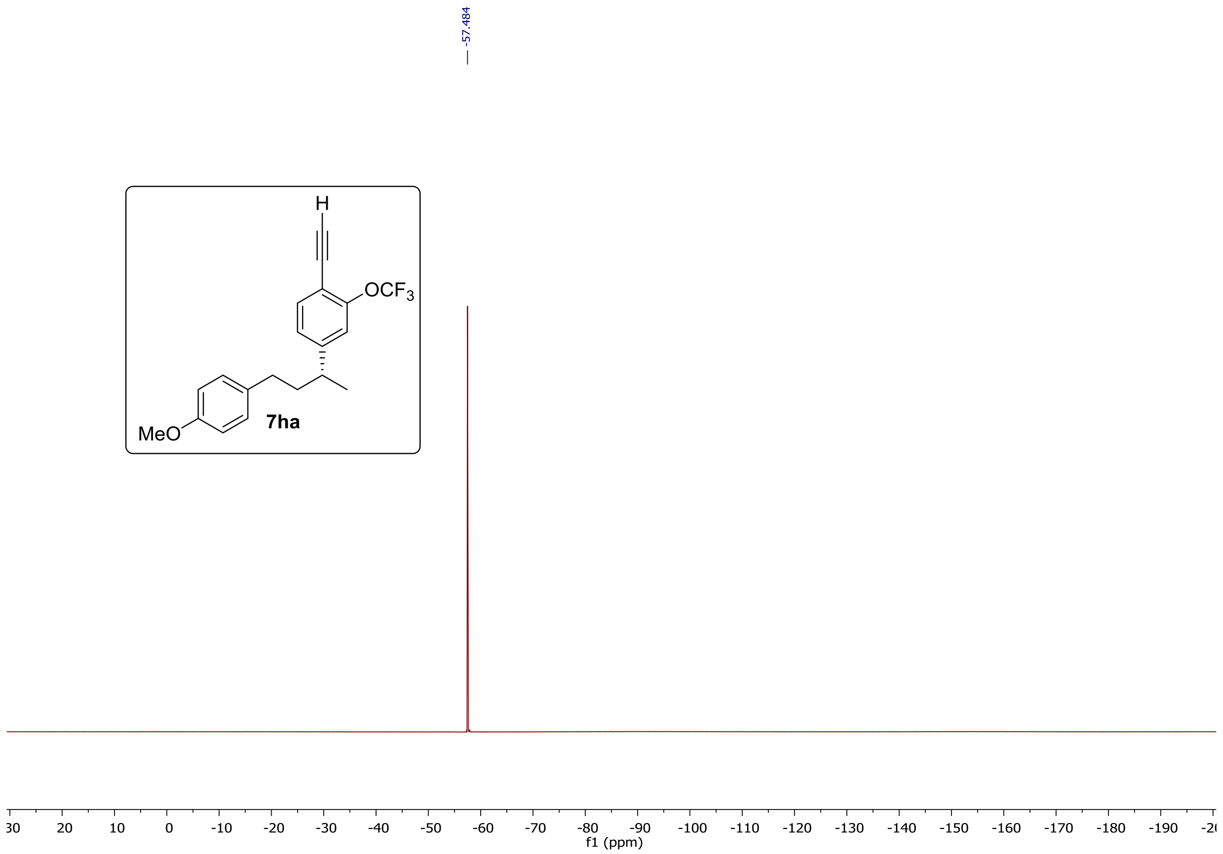
-57.484
H
OCF3
MeO
7ha
30 20 10 0 -10 -20 -30 -40 -50 -60 -70 -80 -90 -100 -110 -120 -130 -140 -150 -160 -170 -180 -190
f1 (ppm)

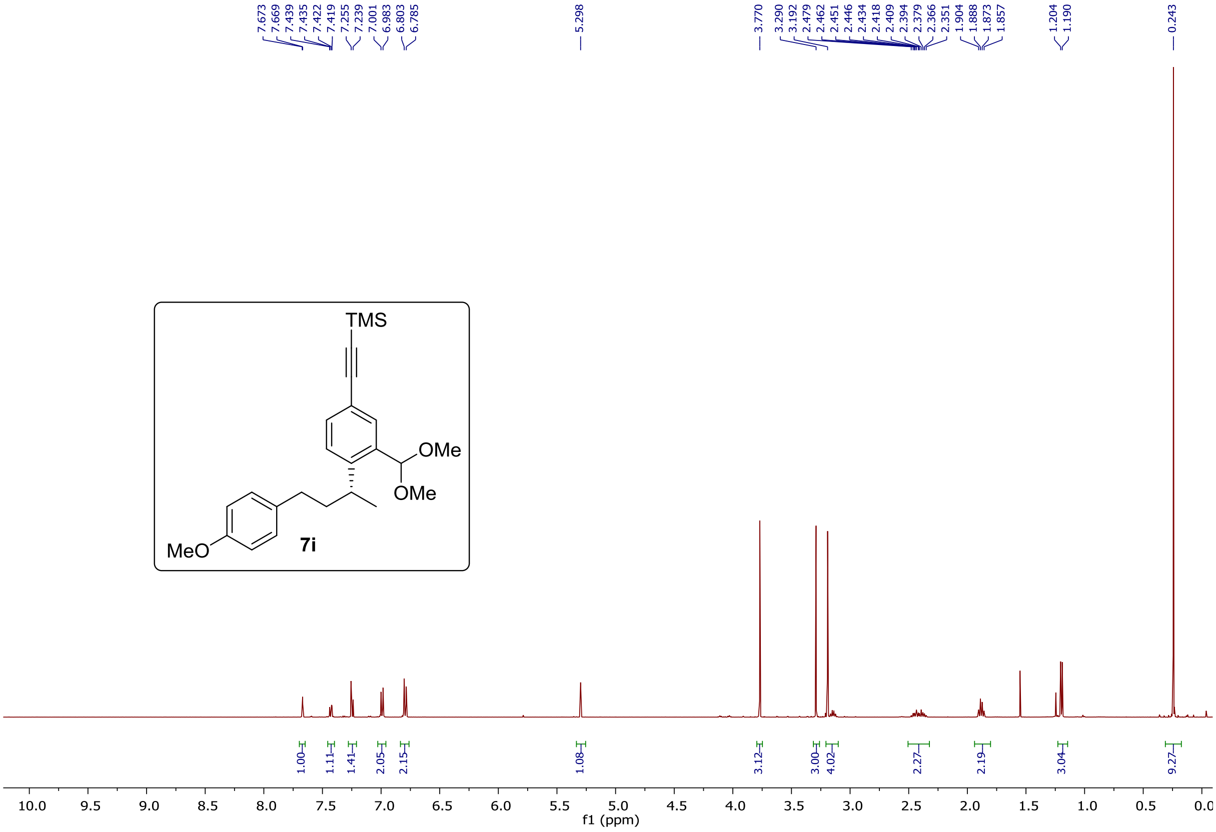
7.673 7.669 7.439 7.435 7.422 7.419 7.255 7.239 7.001 6.983 6.803 6.785
5.298
3.770 3.290 3.192 2.479 2.462 2.451 2.446 2.434 2.418 2.409 2.394 2.379 2.366 2.351 1.904 1.888 1.873 1.857
1.204 1.190
0.243
TMS
OMe
OMe
MeO
7i
1.00 1.11 1.41 2.05 2.15 1.08 3.12 3.00 4.02 2.27 2.19 3.04 9.27
10.0 9.5 9.0 8.5 8.0 7.5 7.0 6.5 6.0 5.5 5.0 4.5 4.0 3.5 3.0 2.5 2.0 1.5 1.0 0.5
f1 (ppm)

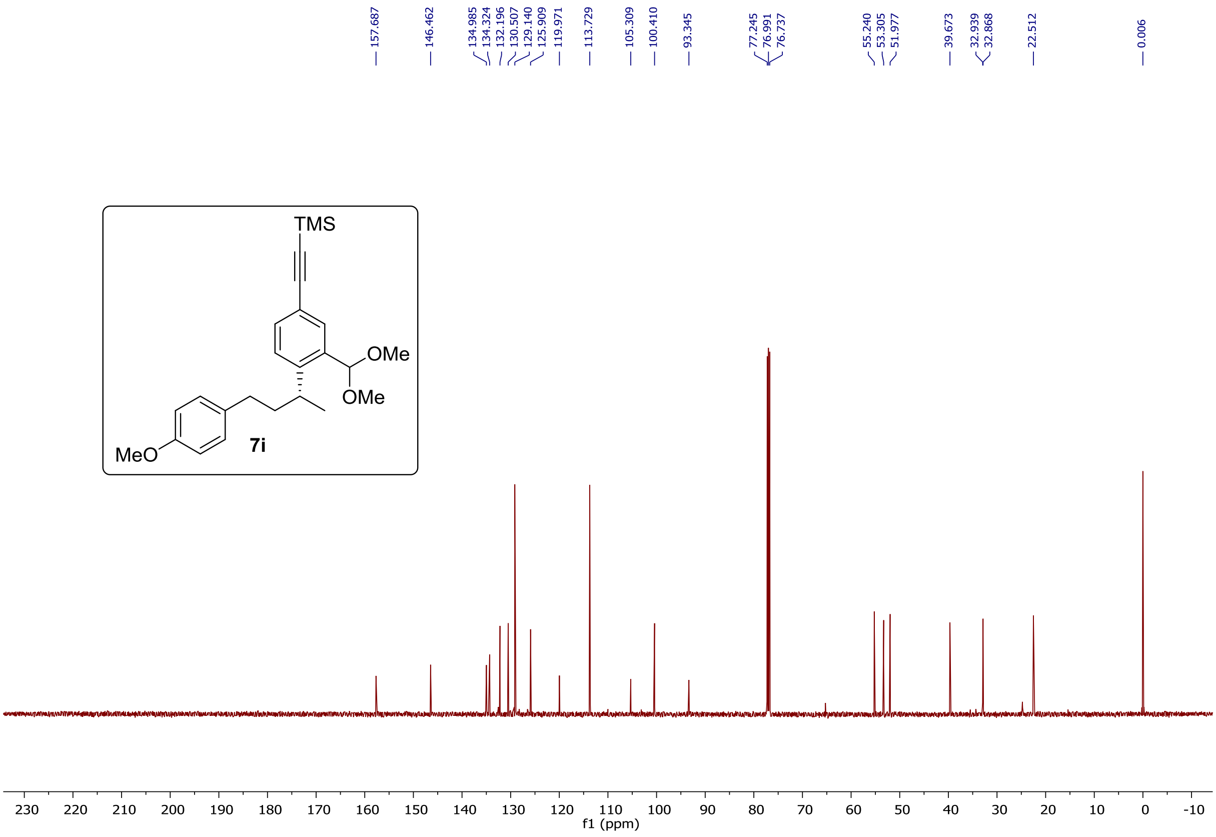
157.687
146.462
134.985 134.324 132.196 130.507 129.140 125.909 119.971
113.729
105.309
100.410
93.345
77.245 76.991 76.737
55.240 53.305 51.977
39.673
32.939 32.868
22.512
0.006
TMS
OMe
OMe
MeO
7i
230 220 210 200 190 180 170 160 150 140 130 120 110 100 90 80 70 60 50 40 30 20 10 0 -10
f1 (ppm)

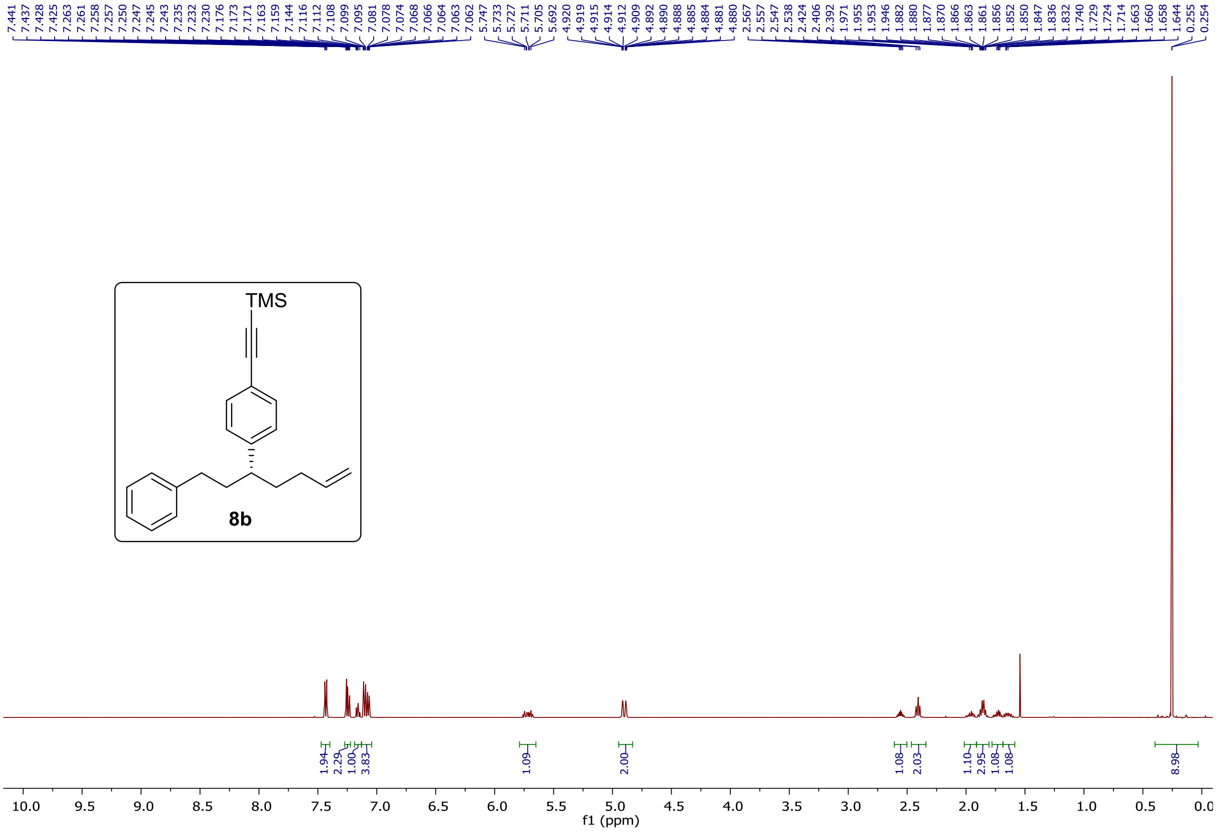
7.441
7.437
7.428
7.425
7.263
7.261
7.258
7.257
7.250
7.247
7.245
7.243
7.235
7.232
7.230
7.176
7.173
7.171
7.163
7.159
7.144
7.116
7.112
7.108
7.099
7.095
7.081
7.078
7.074
7.068
7.066
7.064
7.063
7.062
5.747
5.733
5.727
5.711
5.705
5.692
4.920
4.919
4.915
4.914
4.912
4.909
4.892
4.890
4.888
4.885
4.884
4.881
4.880
2.567
2.557
2.547
2.538
2.424
2.406
2.392
1.971
1.955
1.953
1.946
1.882
1.880
1.877
1.870
1.866
1.863
1.861
1.856
1.852
1.850
1.847
1.836
1.832
1.740
1.729
1.724
1.714
1.663
1.660
1.658
1.644
0.255
0.254
TMS
8b
1.94
2.29
1.00
3.83
1.09
2.00
1.08
2.03
1.10
2.95
1.08
1.08
8.98
10.0 9.5 9.0 8.5 8.0 7.5 7.0 6.5 6.0 5.5 5.0 4.5 4.0 3.5 3.0 2.5 2.0 1.5 1.0 0.5 0.0
f1 (ppm)

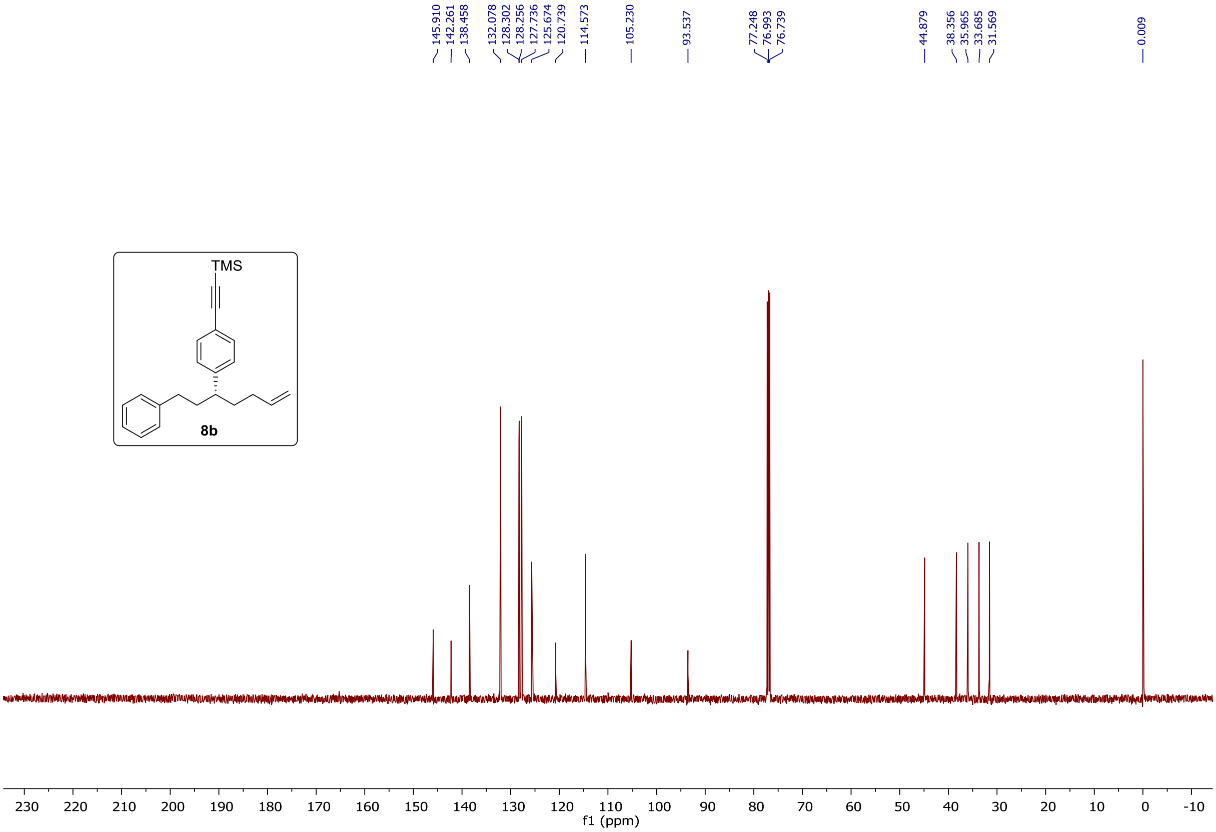
145.910
142.261
138.458
132.078
128.302
128.256
127.736
125.674
120.739
114.573
105.230
93.537
77.248
76.993
76.739
44.879
38.356
35.965
33.685
31.569
0.009
TMS
8b
230 220 210 200 190 180 170 160 150 140 130 120 110 100 90 80 70 60 50 40 30 20 10 0 -10
f1 (ppm)

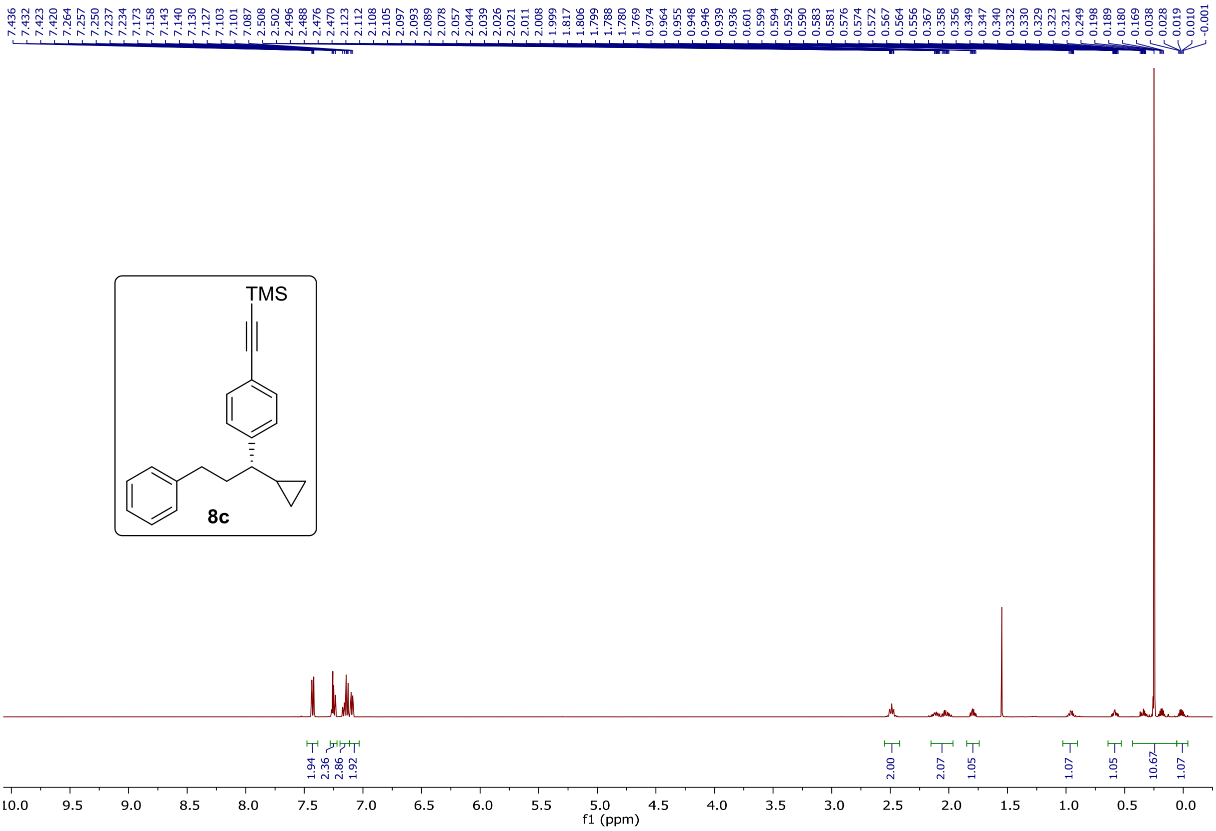
7.436
7.432
7.423
7.420
7.264
7.257
7.250
7.237
7.234
7.173
7.158
7.143
7.140
7.130
7.127
7.103
7.101
7.087
2.508
2.502
2.496
2.488
2.476
2.470
2.123
2.112
2.108
2.105
2.097
2.093
2.089
2.078
2.057
2.044
2.039
2.026
2.021
2.011
2.008
1.999
1.817
1.806
1.799
1.788
1.780
1.769
0.974
0.964
0.955
0.948
0.946
0.939
0.936
0.601
0.599
0.594
0.592
0.590
0.583
0.581
0.576
0.574
0.572
0.567
0.564
0.556
0.367
0.358
0.356
0.349
0.347
0.340
0.332
0.330
0.329
0.323
0.321
0.249
0.198
0.189
0.180
0.169
0.038
0.028
0.019
0.010
-0.001
TMS
8c
1.94
2.36
2.86
1.92
2.00
2.07
1.05
1.07
1.05
10.67
1.07
f1 (ppm)

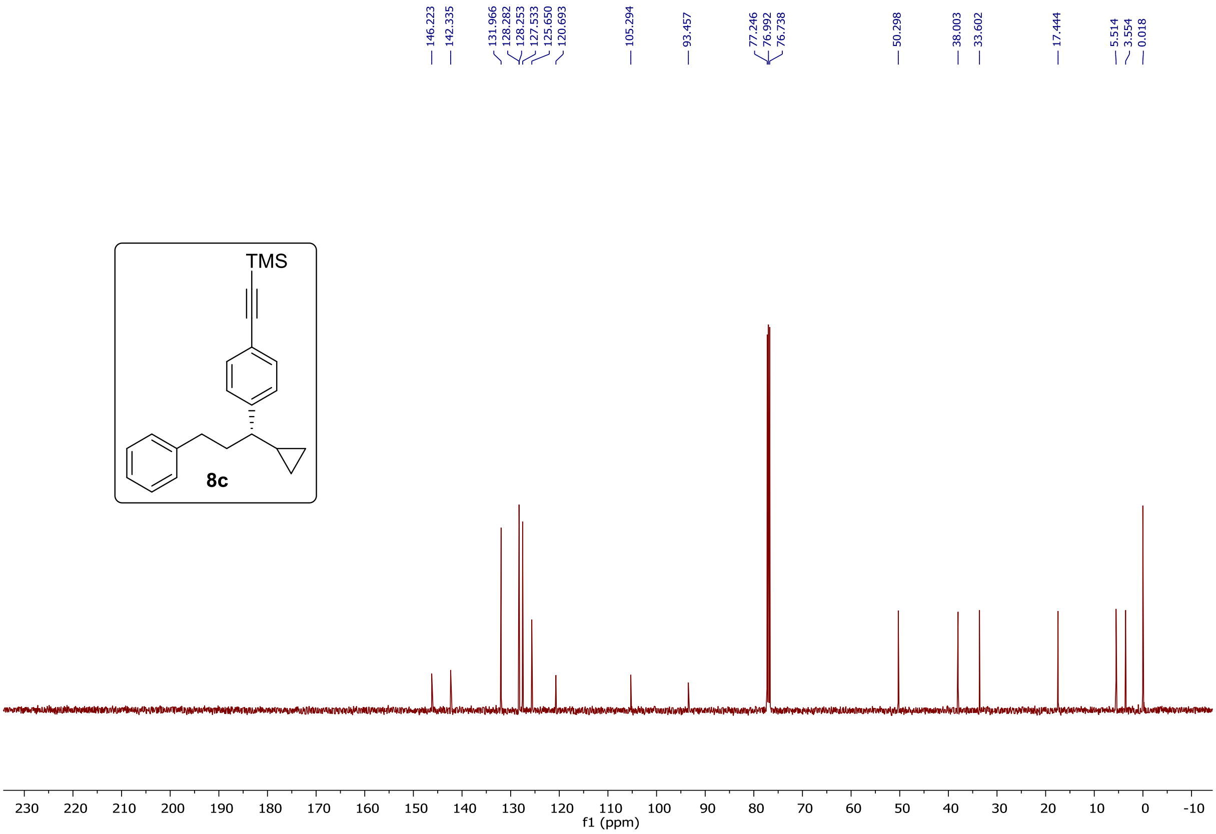
146.223
142.335
131.966
128.282
128.253
127.533
125.650
120.693
105.294
93.457
77.246
76.992
76.738
50.298
38.003
33.602
17.444
5.514
3.554
0.018
TMS
8c
f1 (ppm)

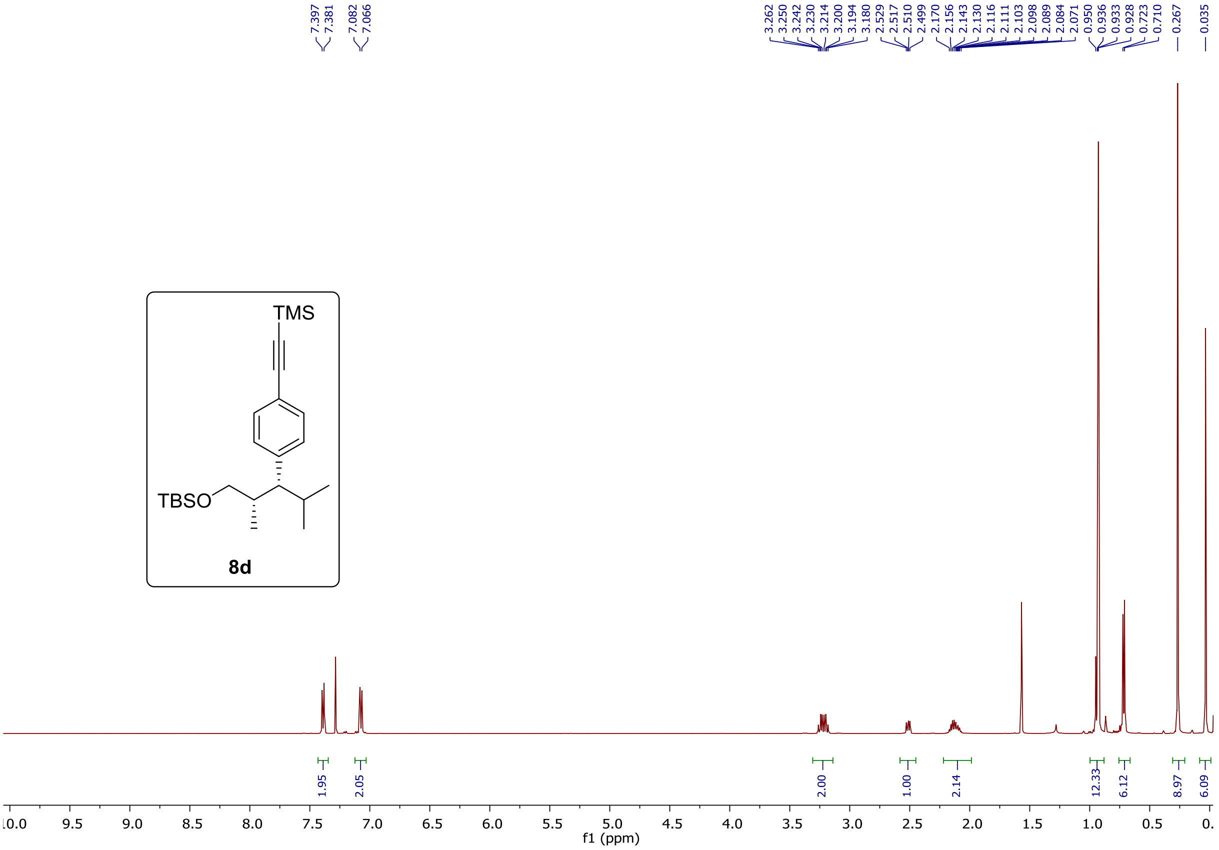

7.397
7.381
7.082
7.066
3.262
3.250
3.242
3.230
3.214
3.200
3.194
3.180
2.529
2.517
2.510
2.499
2.170
2.156
2.143
2.130
2.116
2.111
2.103
2.098
2.089
2.084
2.071
0.950
0.936
0.933
0.928
0.723
0.710
0.267
0.035
TMS
TBSO
8d
1.95
2.05
2.00
1.00
2.14
12.33
6.12
8.97
6.09
f1 (ppm)

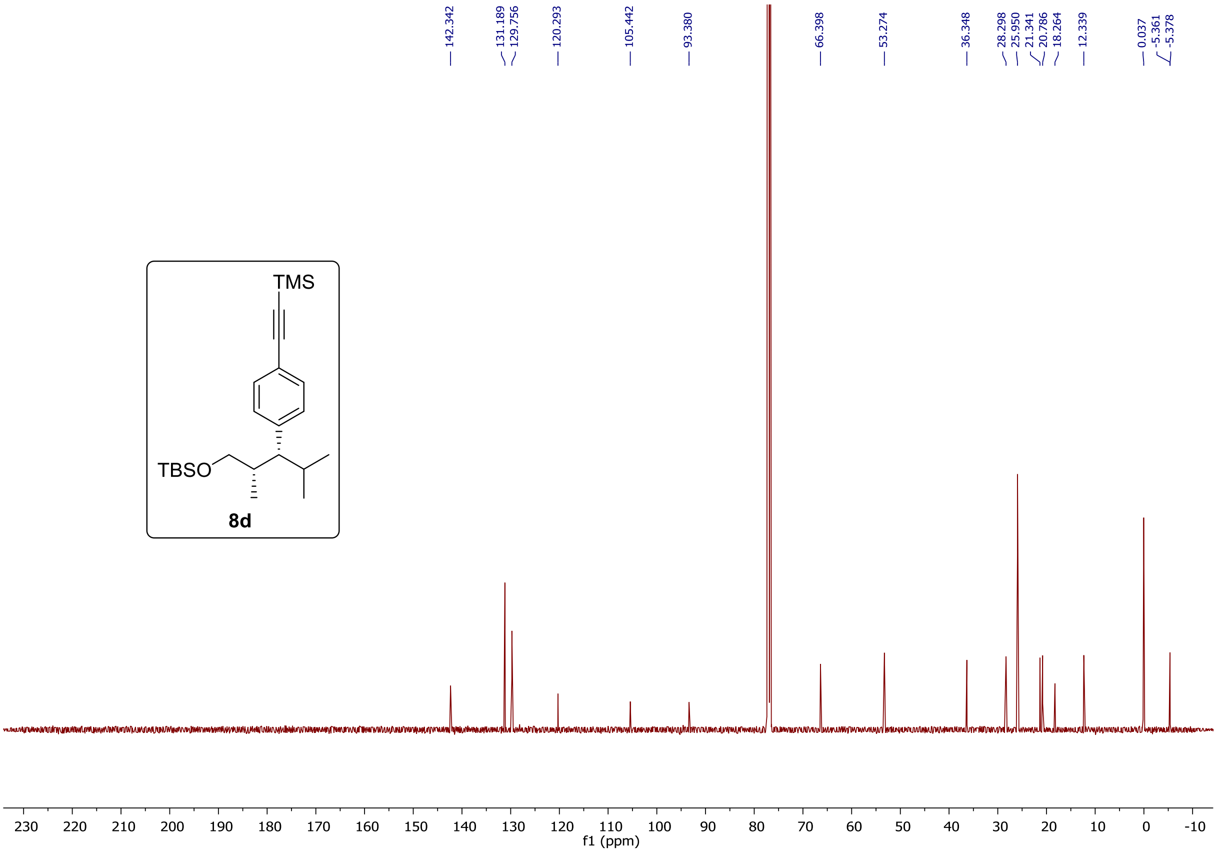

142.342
131.189
129.756
120.293
105.442
93.380
66.398
53.274
36.348
28.298
25.950
21.341
20.786
18.264
12.339
0.037
-5.361
-5.378
TMS
TBSO
8d
f1 (ppm)

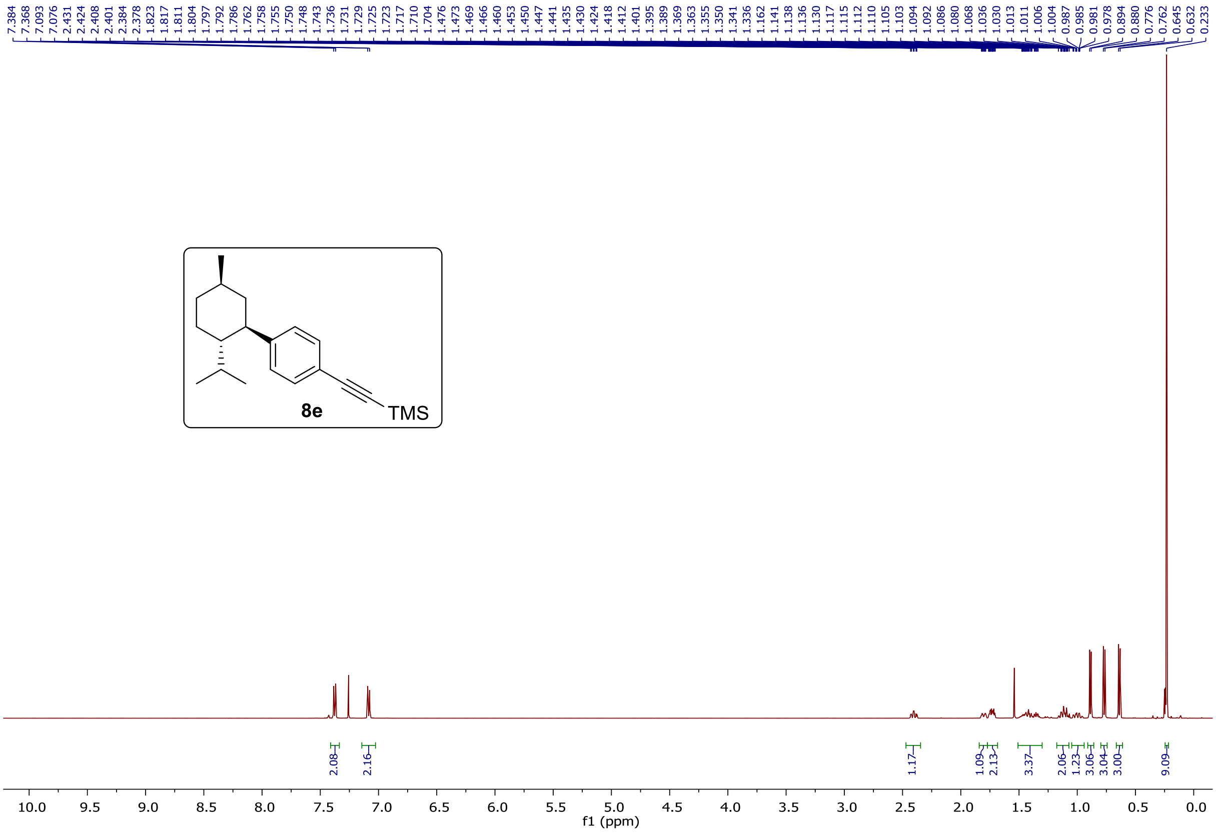
7.384
7.368
7.093
7.076
2.431
2.424
2.408
2.401
2.384
2.378
1.823
1.817
1.811
1.804
1.797
1.792
1.786
1.762
1.758
1.755
1.750
1.748
1.743
1.736
1.731
1.729
1.725
1.723
1.717
1.710
1.704
1.476
1.473
1.469
1.466
1.460
1.453
1.450
1.447
1.441
1.435
1.430
1.424
1.418
1.412
1.401
1.395
1.389
1.369
1.363
1.355
1.350
1.341
1.336
1.162
1.141
1.138
1.136
1.130
1.117
1.115
1.112
1.110
1.105
1.103
1.094
1.092
1.086
1.080
1.068
1.036
1.030
1.013
1.011
1.006
1.004
0.987
0.985
0.981
0.978
0.894
0.880
0.776
0.762
0.645
0.632
0.233
8e
TMS
2.08
2.16
1.17
1.09
2.13
3.37
2.06
1.23
3.06
3.04
3.00
9.09
f1 (ppm)

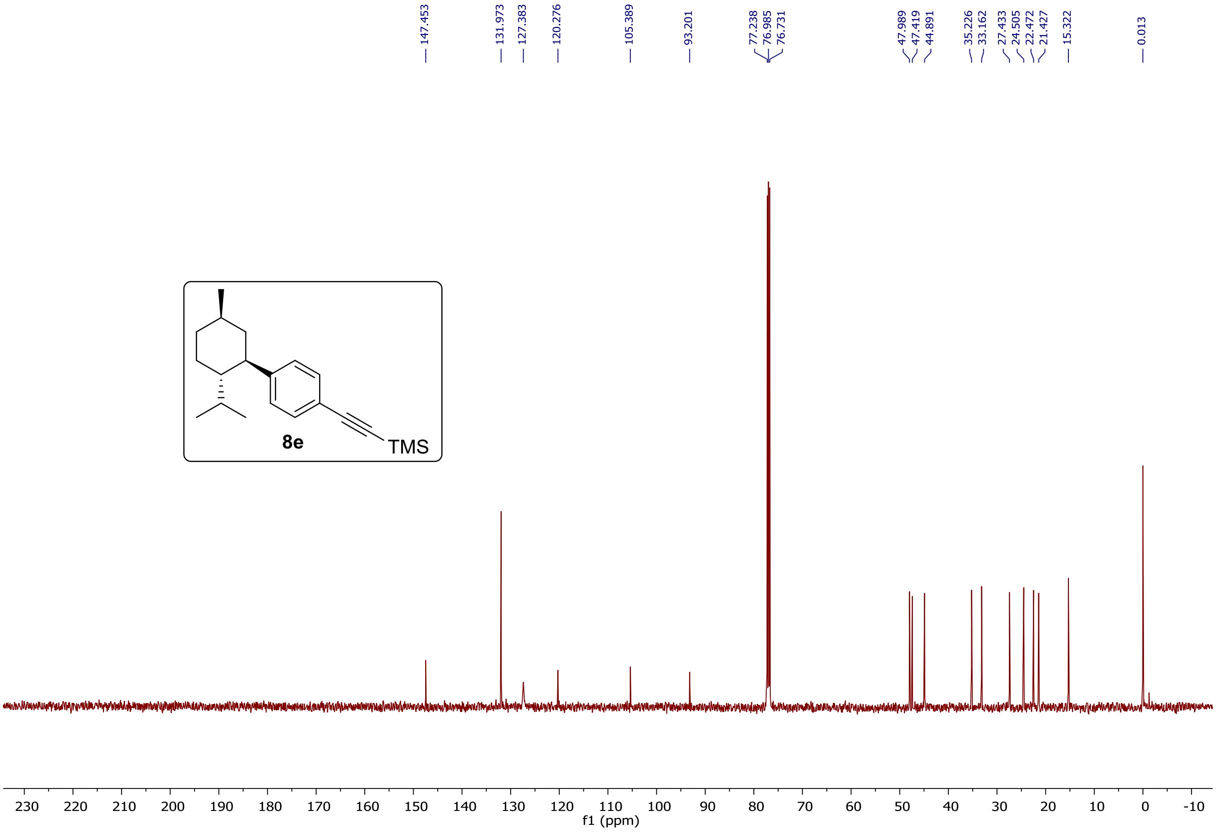
147.453
131.973
127.383
120.276
105.389
93.201
77.238
76.985
76.731
47.989
47.419
44.891
35.226
33.162
27.433
24.505
22.472
21.427
15.322
0.013
8e
TMS
f1 (ppm)

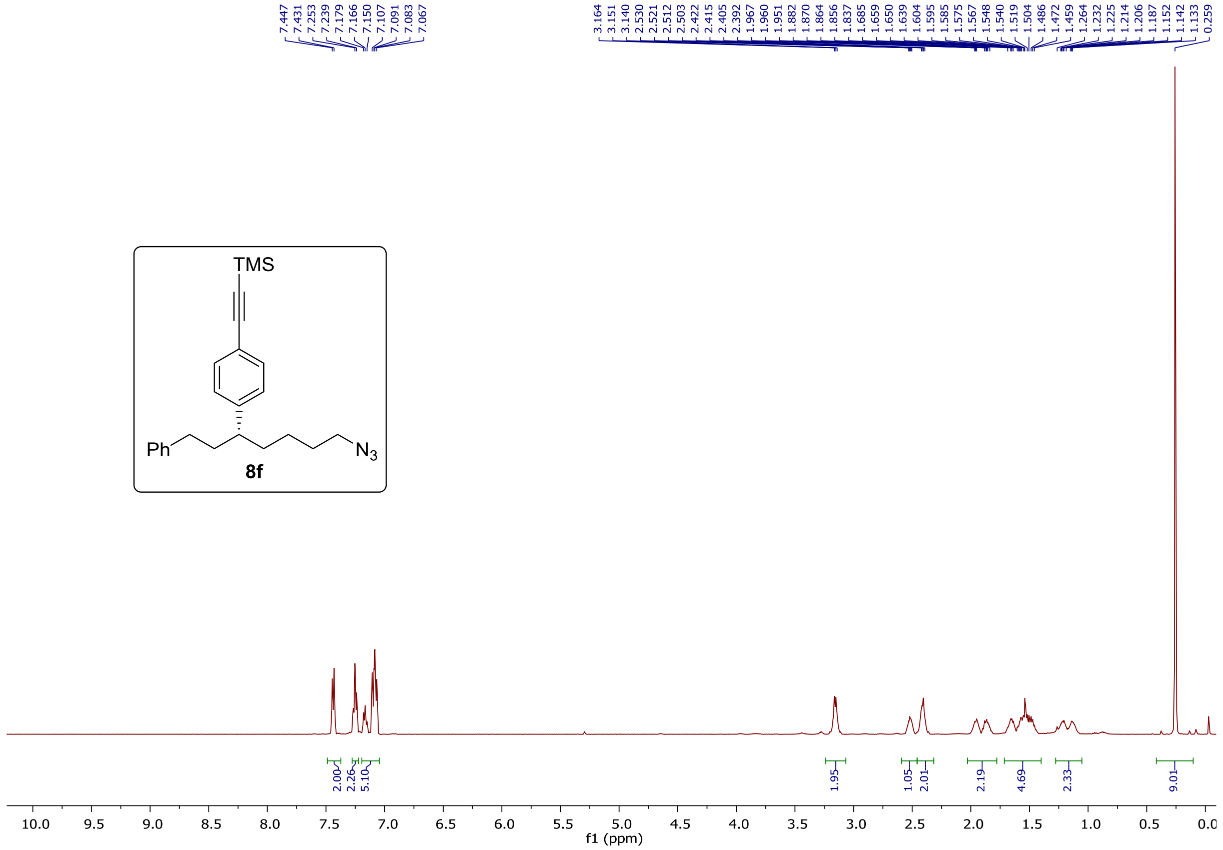
7.447 7.431 7.253 7.239 7.179 7.166 7.150 7.107 7.091 7.083 7.067
3.164 3.151 3.140 2.530 2.521 2.512 2.503 2.422 2.415 2.405 2.392 1.967 1.960 1.951 1.882 1.870 1.864 1.856 1.837 1.685 1.659 1.650 1.639 1.604 1.595 1.585 1.575 1.567 1.548 1.540 1.519 1.504 1.486 1.472 1.459 1.264 1.232 1.225 1.214 1.206 1.187 1.152 1.142 1.133 0.259
TMS
Ph
N3
8f
2.00 2.26 5.10 1.95 1.05 2.01 2.19 4.69 2.33 9.01
f1 (ppm)

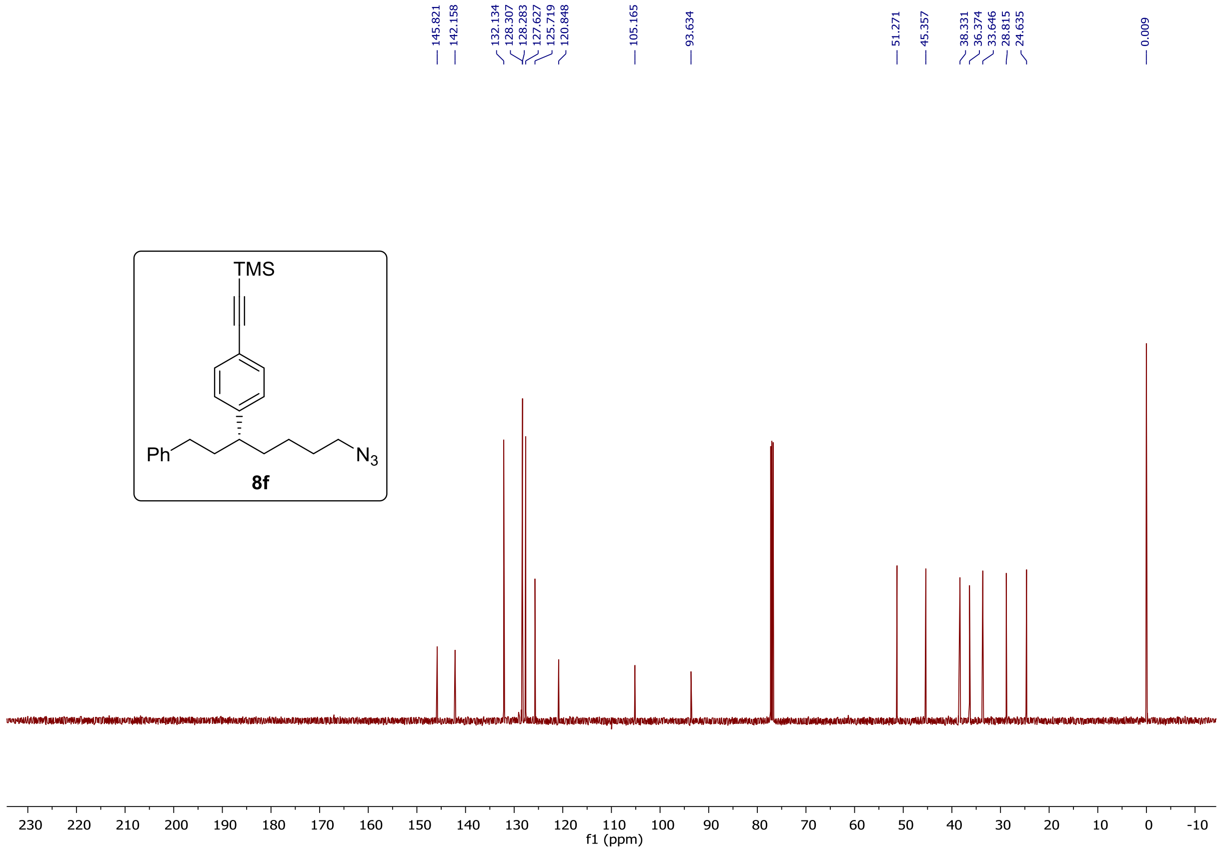
145.821 142.158 132.134 128.307 128.283 127.627 125.719 120.848 105.165 93.634 51.271 45.357 38.331 36.374 33.646 28.815 24.635 0.009
TMS
Ph
N3
8f
f1 (ppm)

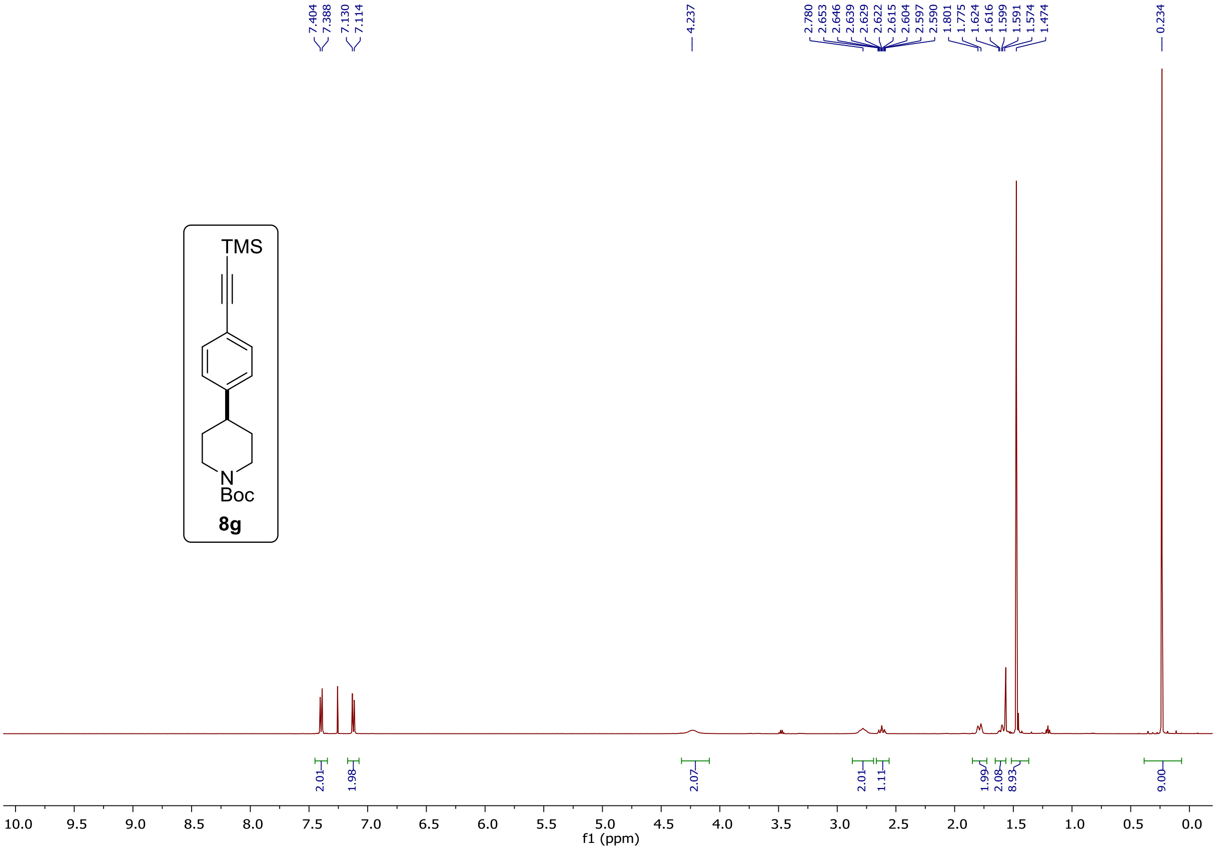
7.404
7.388
7.130
7.114
4.237
2.780
2.653
2.646
2.639
2.629
2.622
2.615
2.604
2.597
2.590
1.801
1.775
1.624
1.616
1.599
1.591
1.574
1.474
0.234
TMS
N
Boc
8g
2.01
1.98
2.07
2.01
1.11
1.99
2.08
8.93
9.00
f1 (ppm)

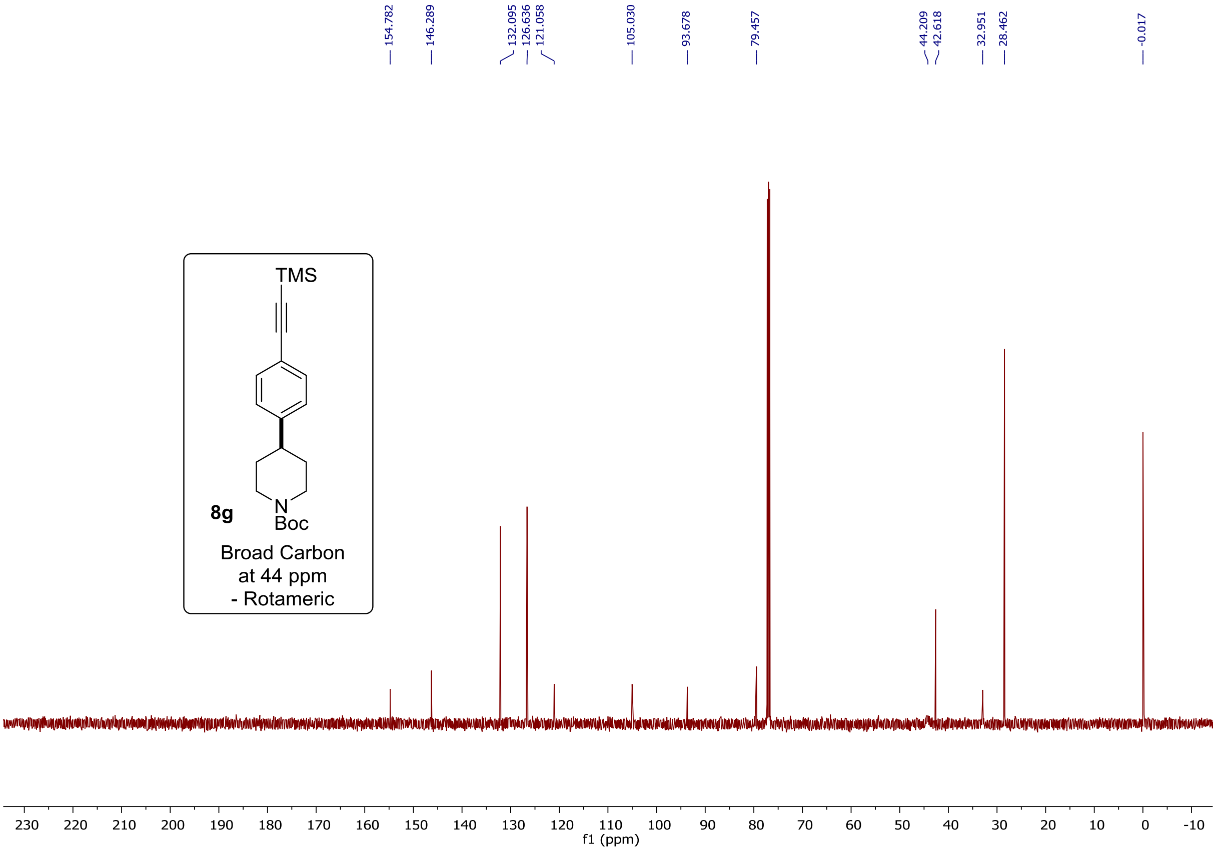
154.782
146.289
132.095
126.636
121.058
105.030
93.678
79.457
44.209
42.618
32.951
28.462
-0.017
TMS
8g
N
Boc
Broad Carbon
at 44 ppm
- Rotameric
f1 (ppm)

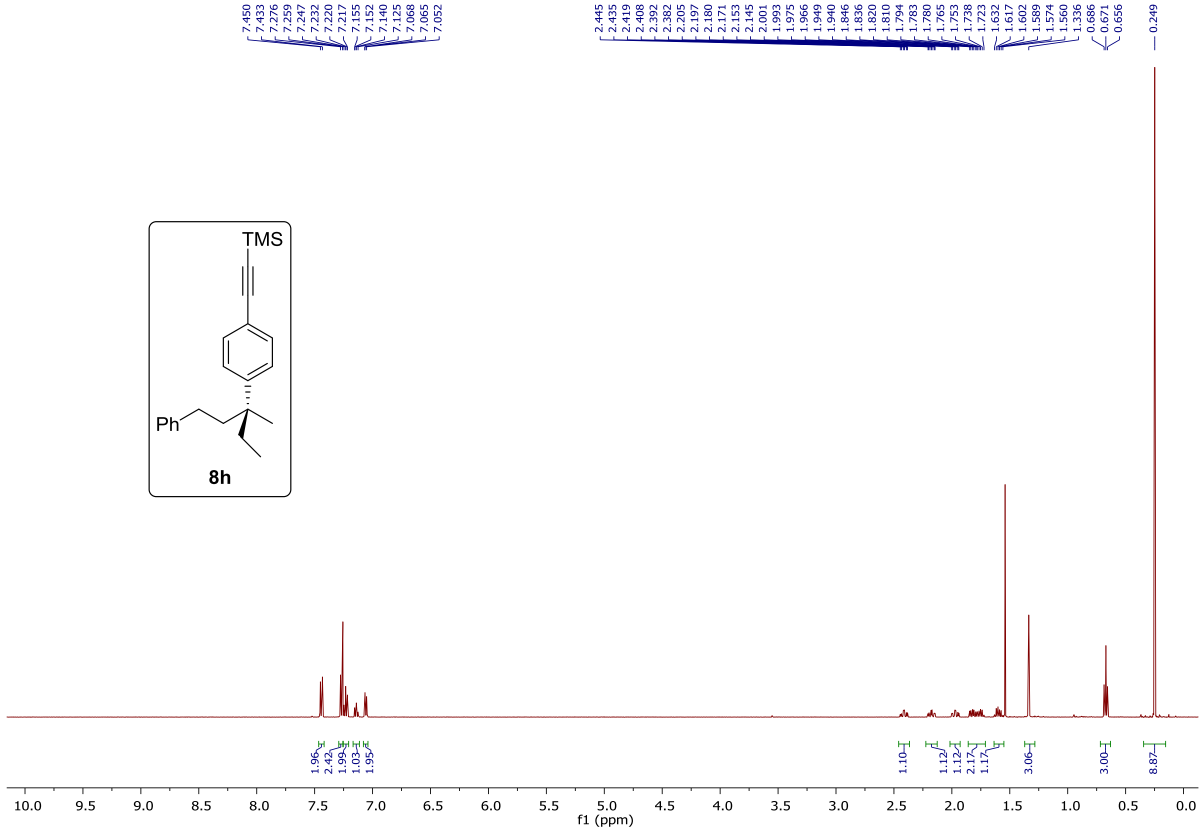
7.450
7.433
7.276
7.259
7.247
7.232
7.220
7.217
7.155
7.152
7.140
7.125
7.068
7.065
7.052
2.445
2.435
2.419
2.408
2.392
2.382
2.205
2.197
2.180
2.171
2.153
2.145
2.001
1.993
1.975
1.966
1.949
1.940
1.846
1.836
1.820
1.810
1.794
1.783
1.780
1.765
1.753
1.738
1.723
1.632
1.617
1.602
1.589
1.574
1.560
1.336
0.686
0.671
0.656
0.249
TMS
Ph
8h
1.96
2.42
1.99
1.03
1.95
1.10
1.12
1.12
2.17
1.17
3.06
3.00
8.87
10.0
9.5
9.0
8.5
8.0
7.5
7.0
6.5
6.0
5.5
5.0
4.5
4.0
3.5
3.0
2.5
2.0
1.5
1.0
0.5
0.0
f1 (ppm)

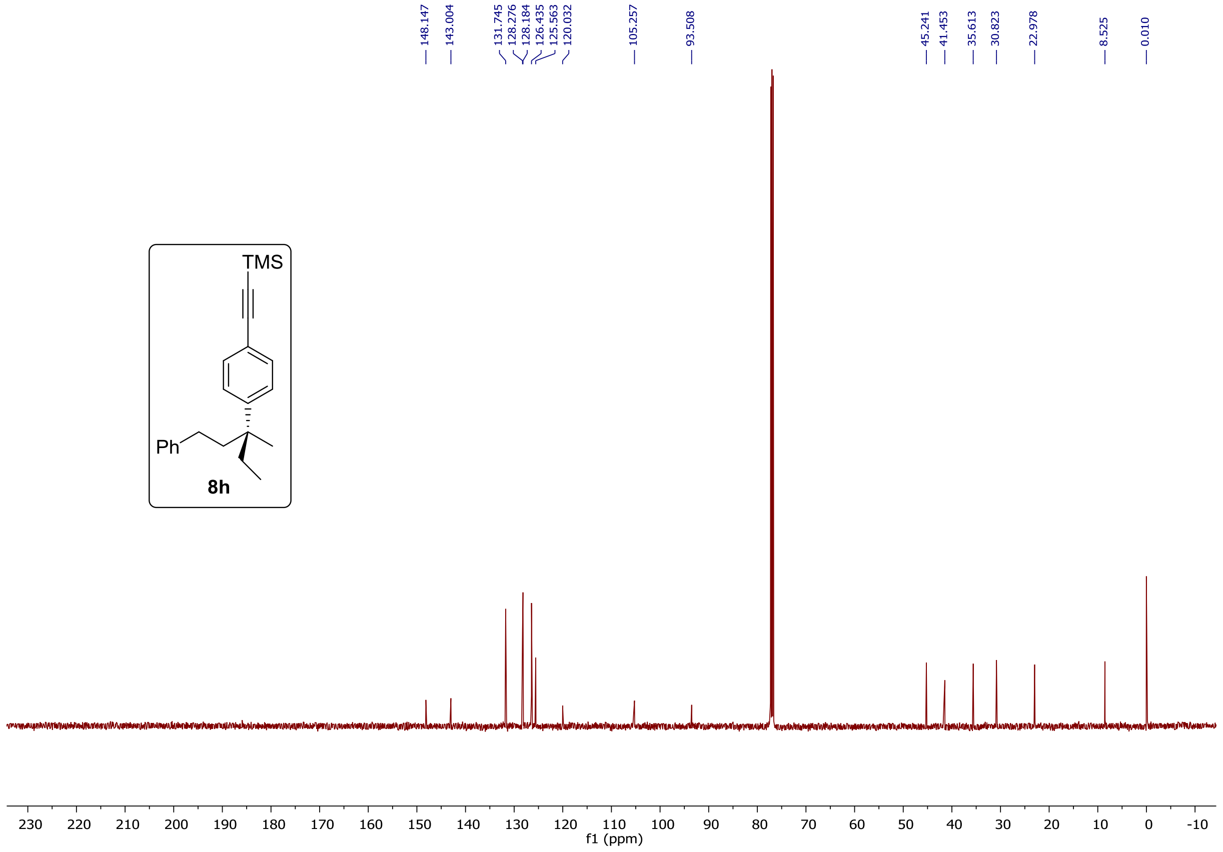
148.147
143.004
131.745
128.276
128.184
126.435
125.563
120.032
105.257
93.508
45.241
41.453
35.613
30.823
22.978
8.525
0.010
TMS
Ph
8h
230
220
210
200
190
180
170
160
150
140
130
120
110
100
90
80
70
60
50
40
30
20
10
0
-10
f1 (ppm)

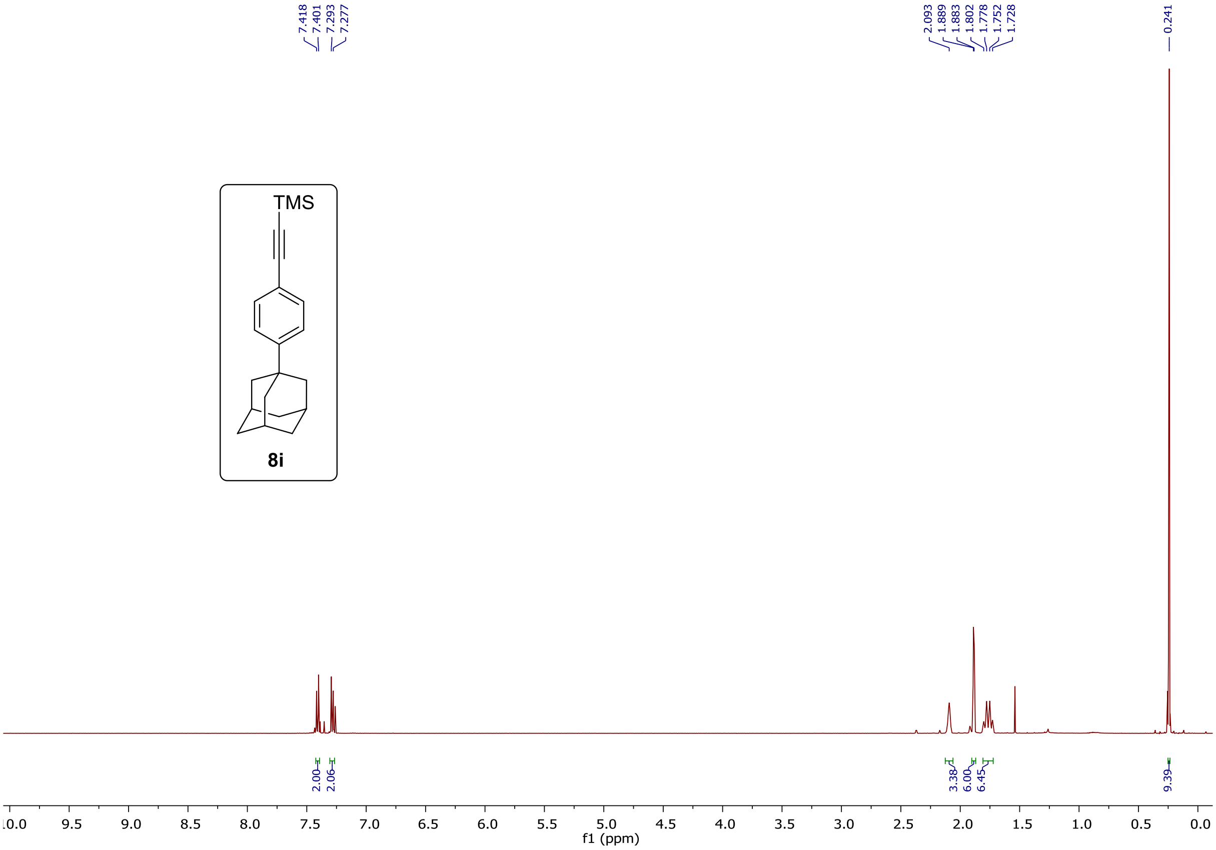
7.418
7.401
7.293
7.277
2.093
1.889
1.883
1.802
1.778
1.752
1.728
0.241
TMS
8i
2.00
2.06
3.38
6.00
6.45
9.39
f1 (ppm)

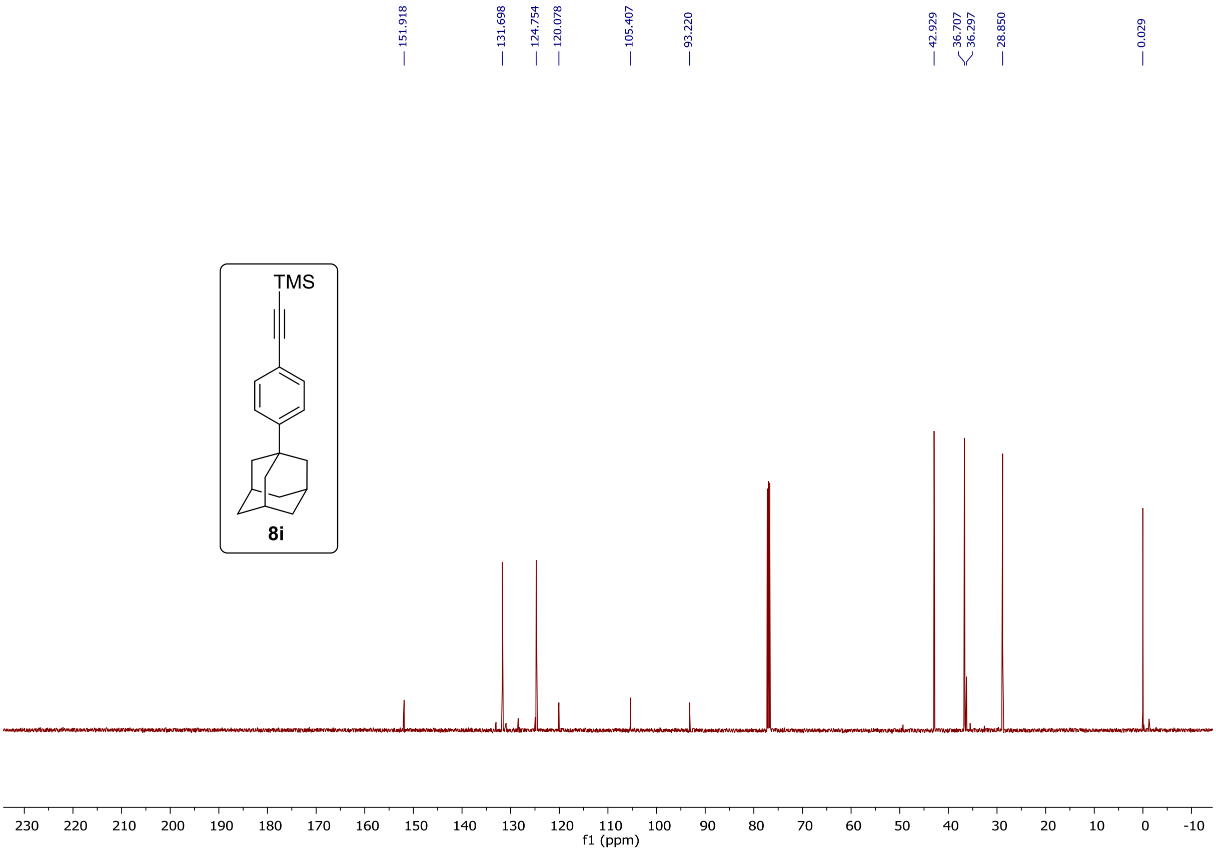
151.918
131.698
124.754
120.078
105.407
93.220
42.929
36.707
36.297
28.850
0.029
TMS
8i
f1 (ppm)

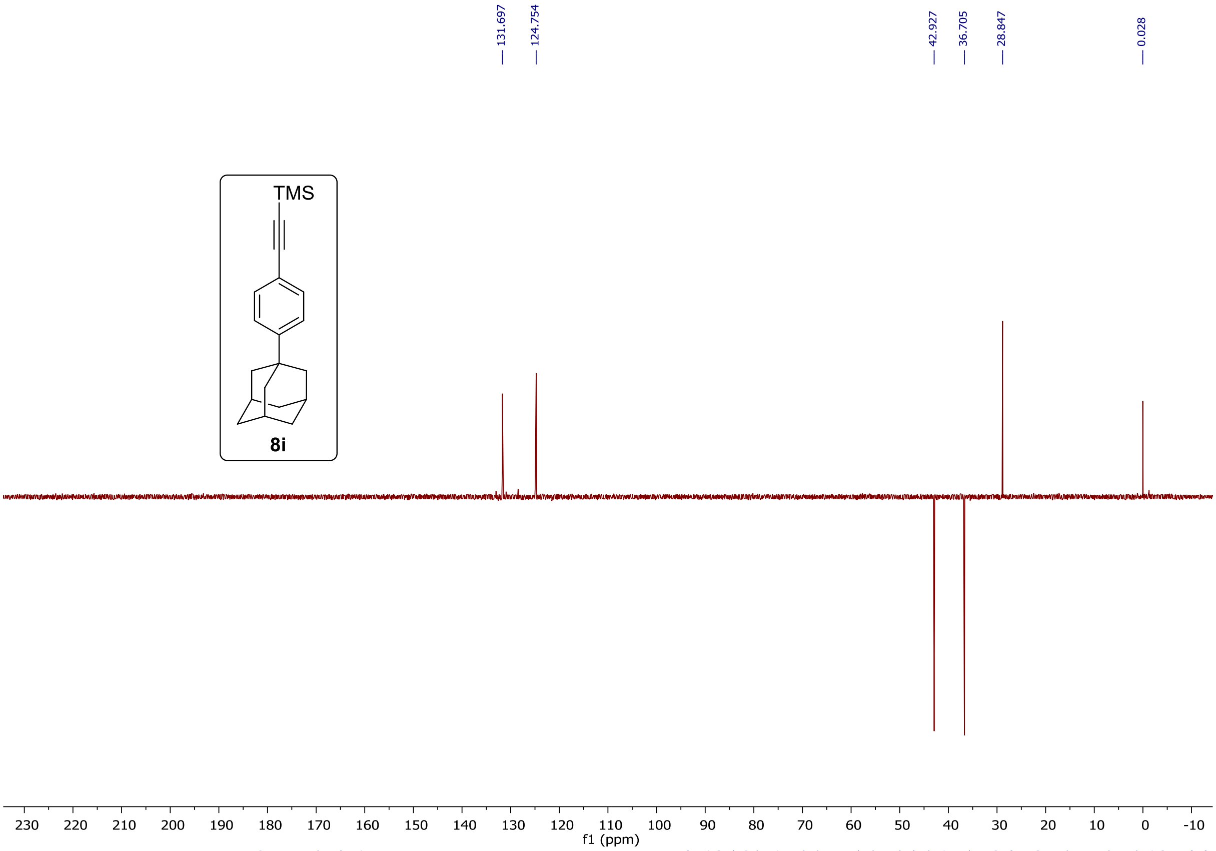
131.697
124.754
42.927
36.705
28.847
0.028
TMS
8i
f1 (ppm)

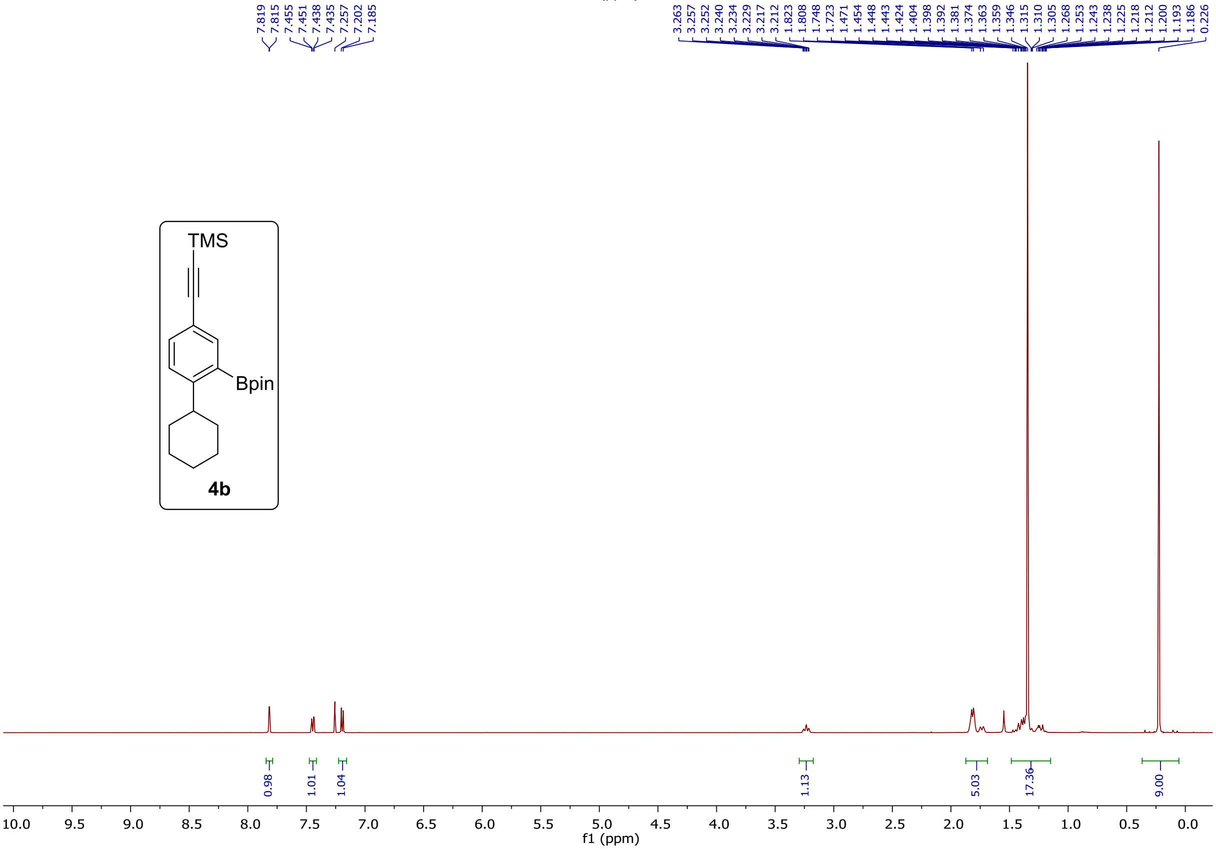
TMS
Bpin
4b
0.98
1.01
1.04
1.13
5.03
17.36
9.00
f1 (ppm)

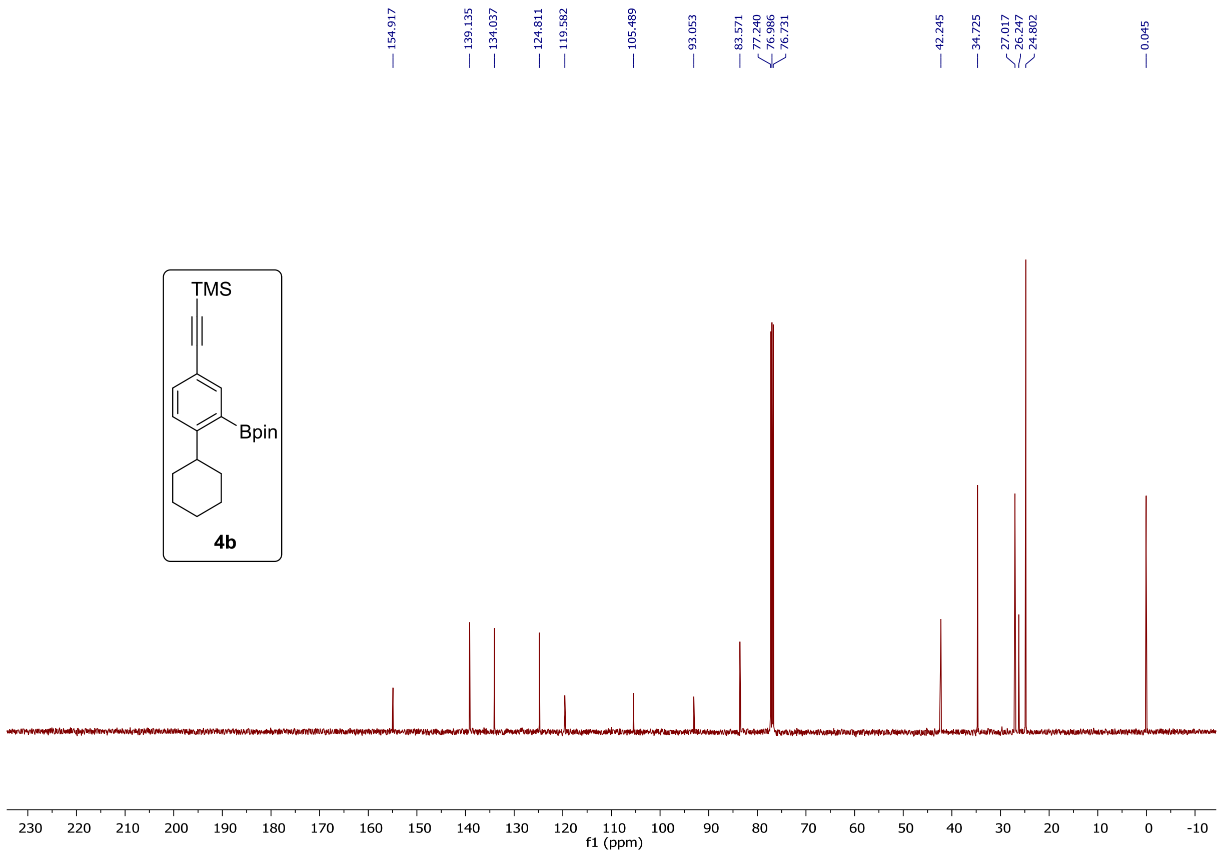
154.917
139.135
134.037
124.811
119.582
105.489
93.053
83.571
77.240
76.986
76.731
42.245
34.725
27.017
26.247
24.802
0.045
TMS
Bpin
4b
230 220 210 200 190 180 170 160 150 140 130 120 110 100 90 80 70 60 50 40 30 20 10 0 -10
f1 (ppm)

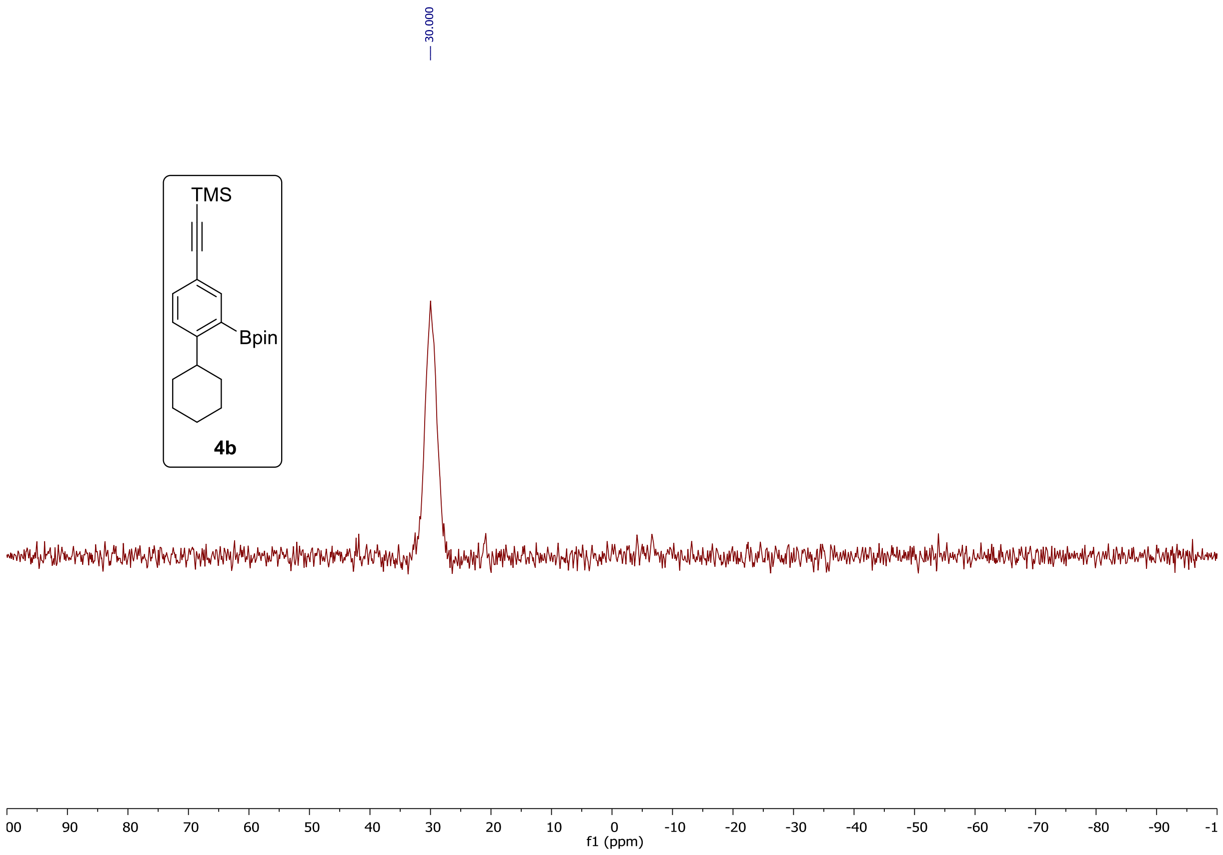
30.000
TMS
Bpin
4b
00 90 80 70 60 50 40 30 20 10 0 -10 -20 -30 -40 -50 -60 -70 -80 -90 -1
f1 (ppm)

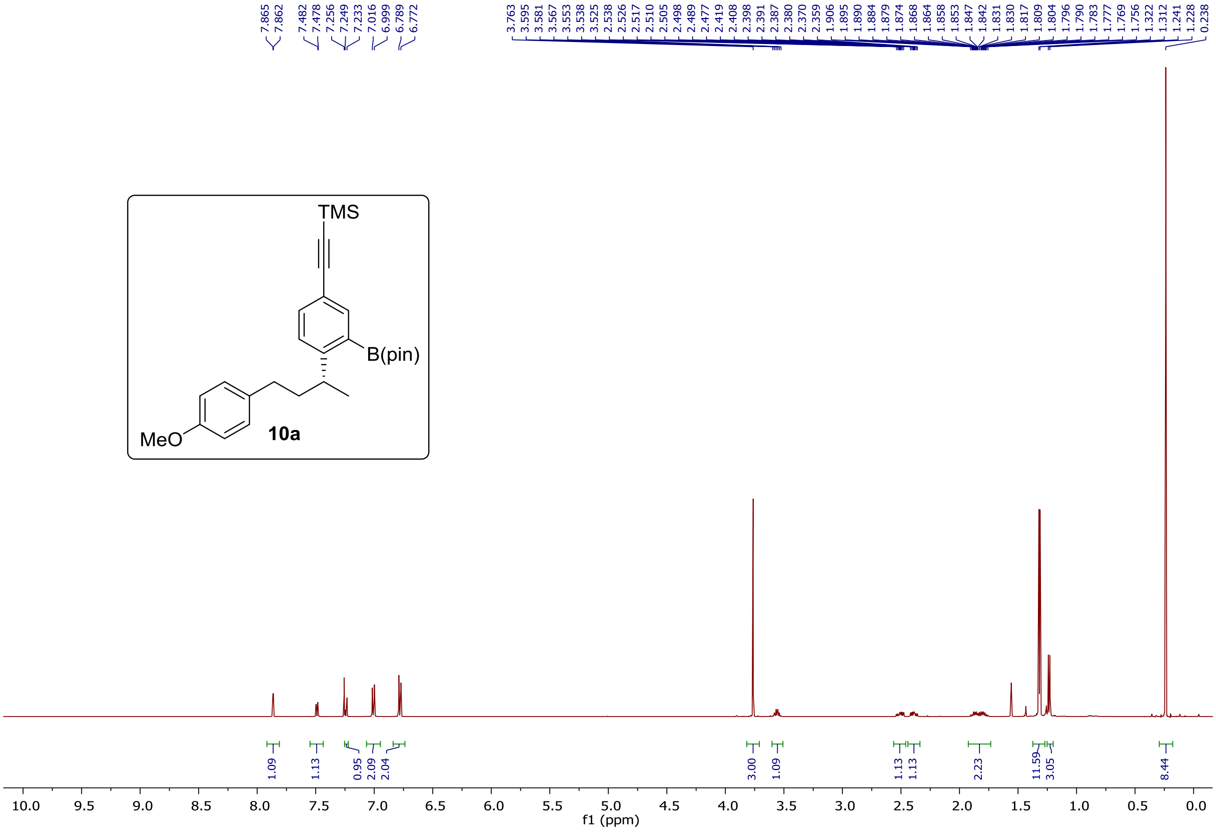
TMS
B(pin)
MeO
10a
7.865
7.862
7.482
7.478
7.256
7.249
7.233
7.016
6.999
6.789
6.772
3.763
3.595
3.581
3.567
3.553
3.538
3.525
2.538
2.526
2.517
2.510
2.505
2.498
2.489
2.477
2.419
2.408
2.398
2.391
2.387
2.380
2.370
2.359
1.906
1.895
1.890
1.884
1.879
1.874
1.868
1.864
1.858
1.853
1.847
1.842
1.831
1.830
1.817
1.809
1.804
1.796
1.790
1.783
1.777
1.769
1.756
1.322
1.312
1.241
1.228
0.238
1.09
1.13
0.95
2.09
2.04
3.00
1.09
1.13
1.13
2.23
11.59
3.05
8.44
f1 (ppm)

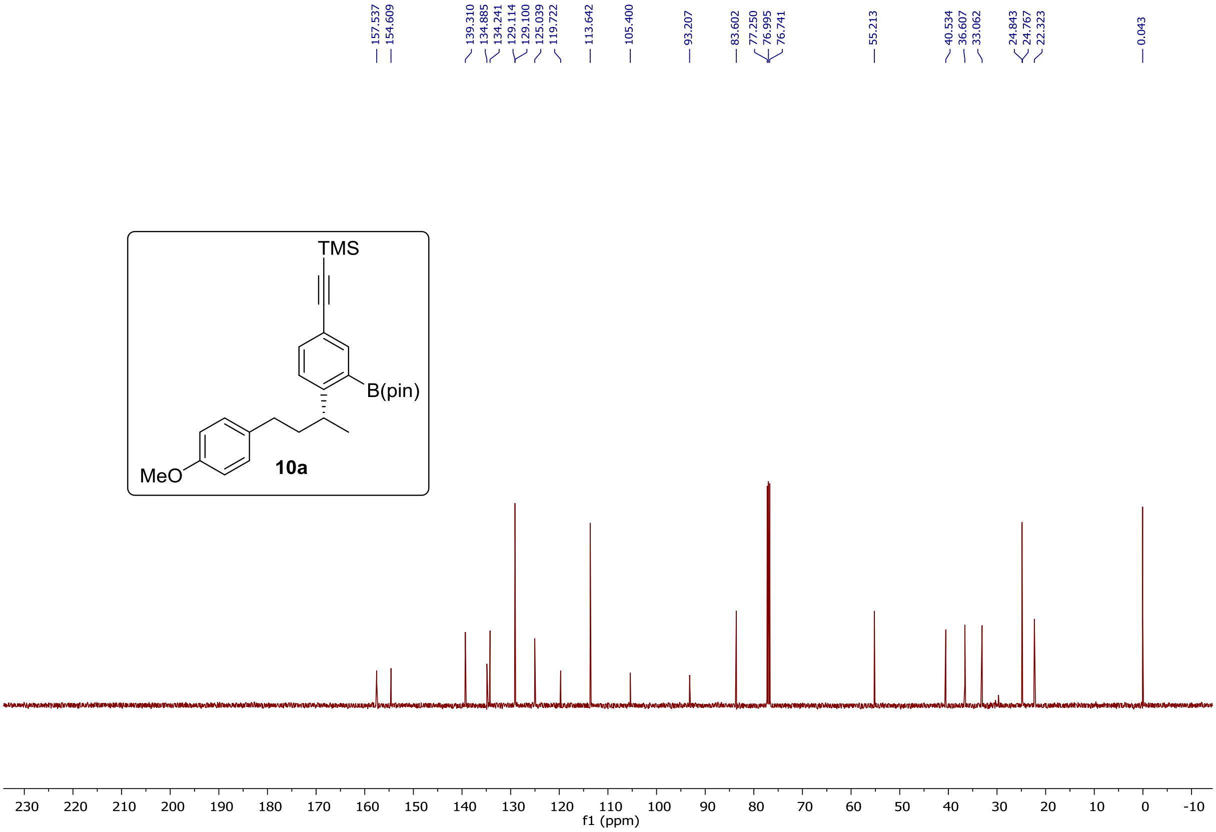
157.537
154.609
139.310
134.885
134.241
129.114
129.100
125.039
119.722
113.642
105.400
93.207
83.602
77.250
76.995
76.741
55.213
40.534
36.607
33.062
24.843
24.767
22.323
0.043
TMS
B(pin)
MeO
10a
f1 (ppm)

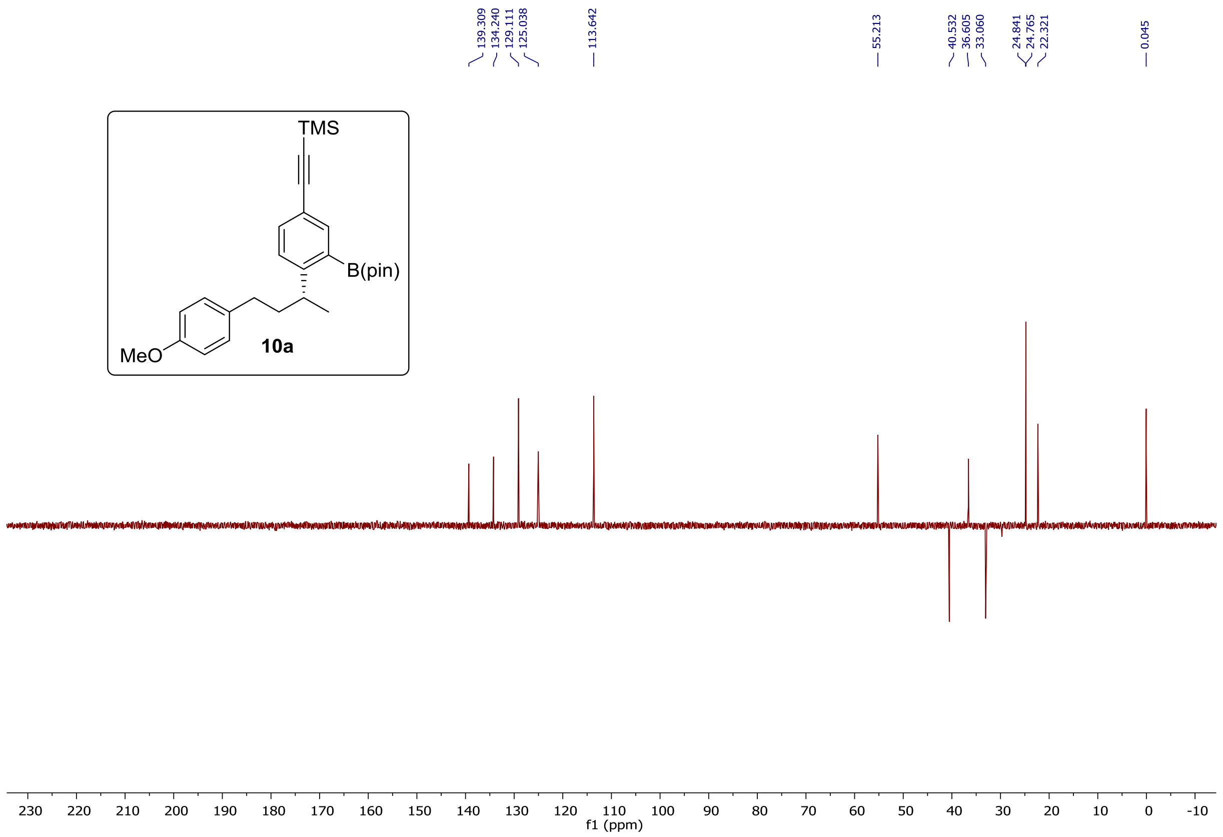
139.309
134.240
129.111
125.038
113.642
55.213
40.532
36.605
33.060
24.841
24.765
22.321
0.045
TMS
B(pin)
MeO
10a
230 220 210 200 190 180 170 160 150 140 130 120 110 100 90 80 70 60 50 40 30 20 10 0 -10
f1 (ppm)

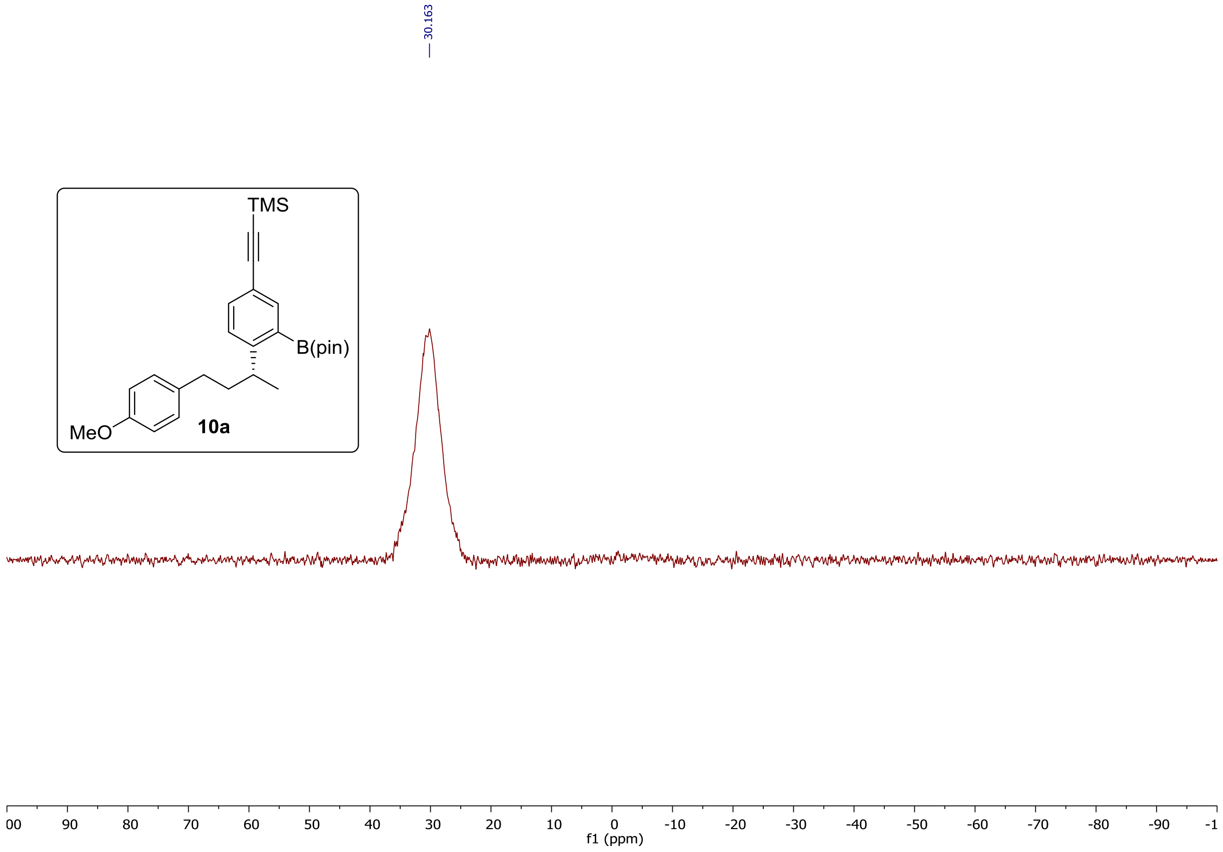
30.163
TMS
B(pin)
MeO
10a
00 90 80 70 60 50 40 30 20 10 0 -10 -20 -30 -40 -50 -60 -70 -80 -90 -1
f1 (ppm)

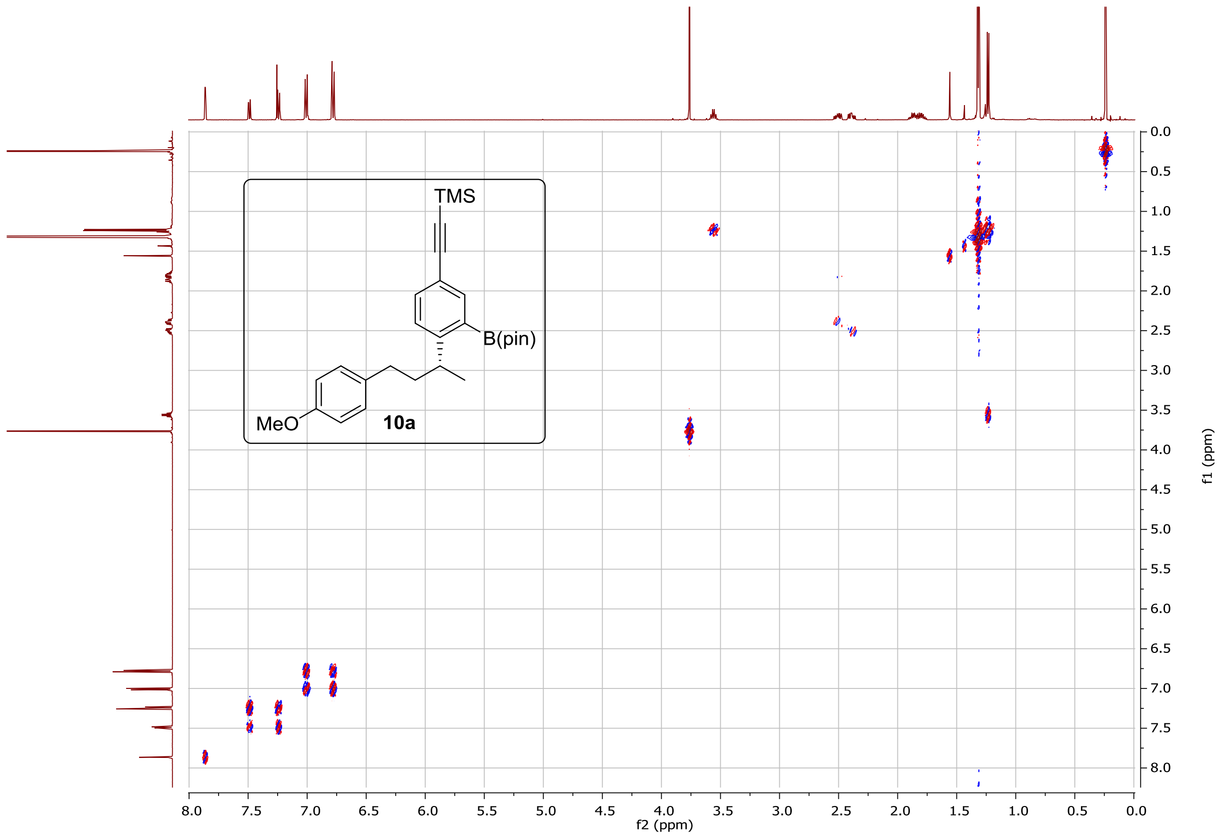
TMS
B(pin)
MeO
10a
f2 (ppm)
f1 (ppm)

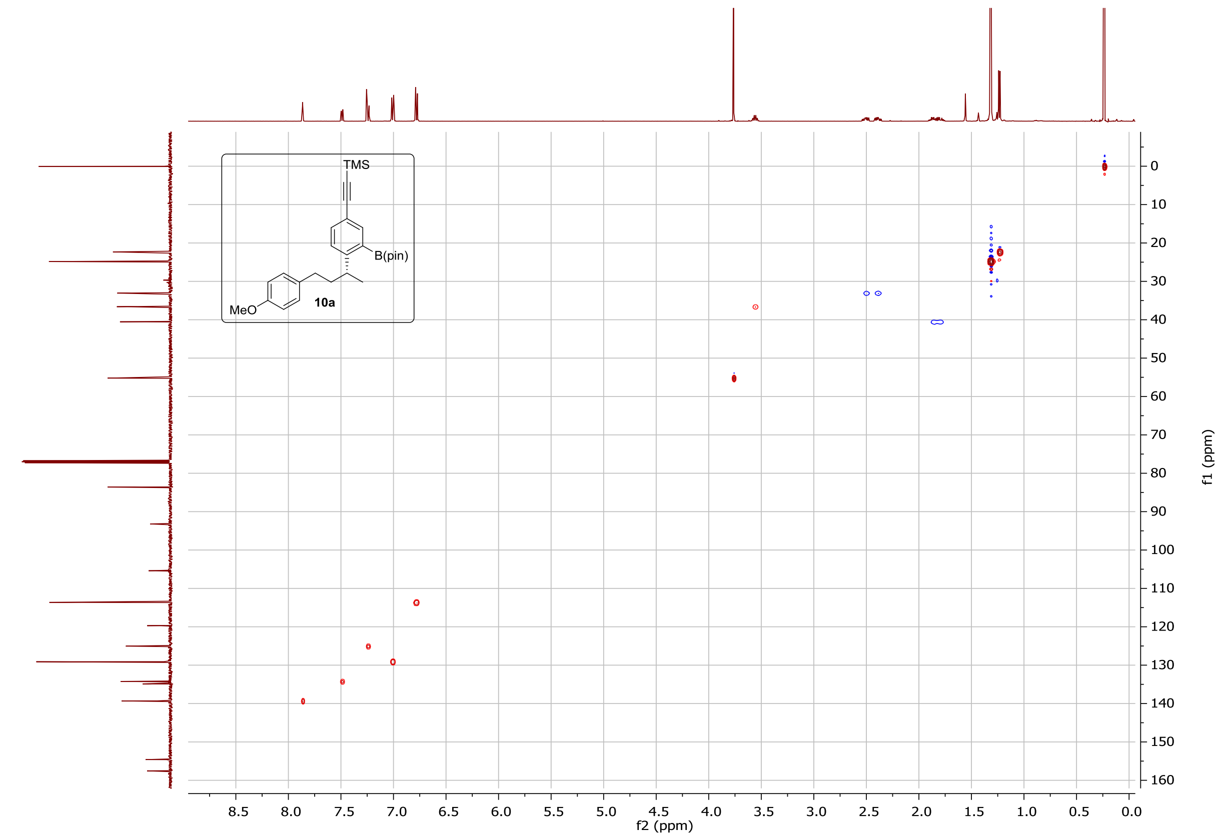
TMS
B(pin)
MeO
10a
f2 (ppm)
f1 (ppm)

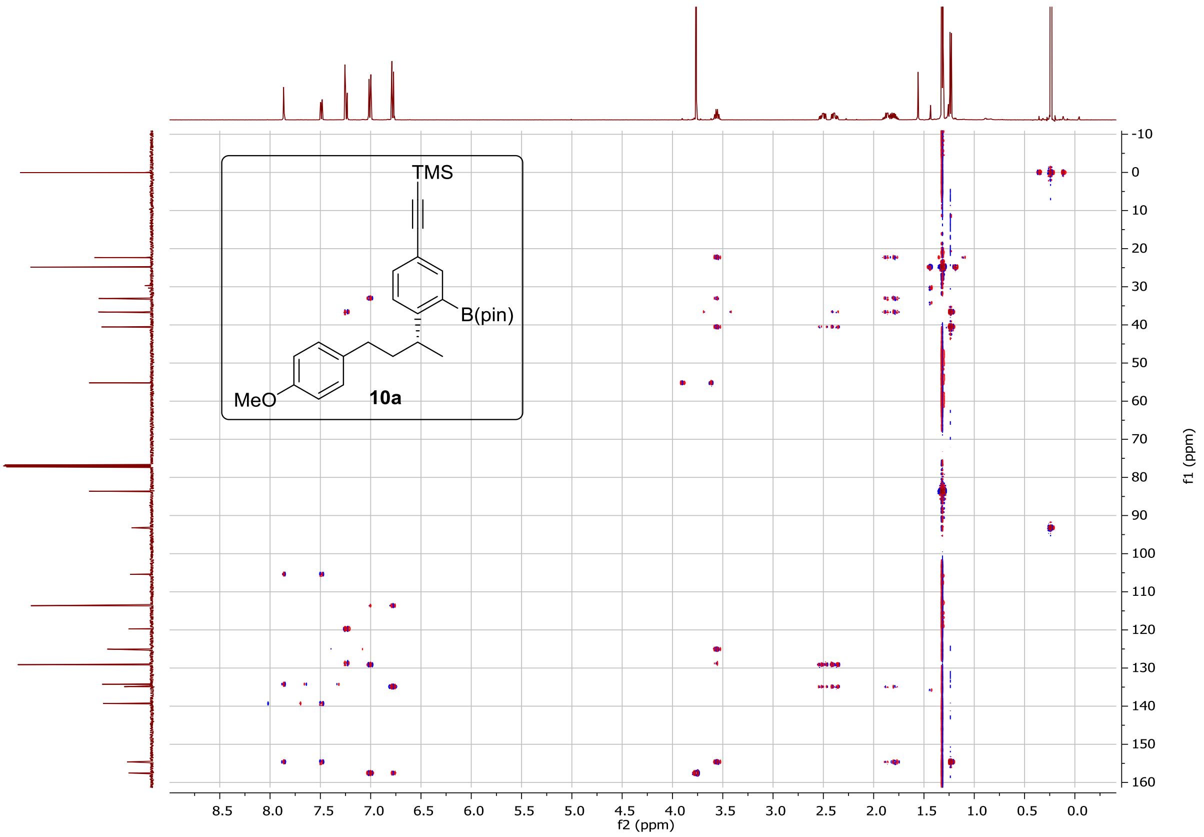
TMS
B(pin)
MeO
10a
f2 (ppm)
f1 (ppm)

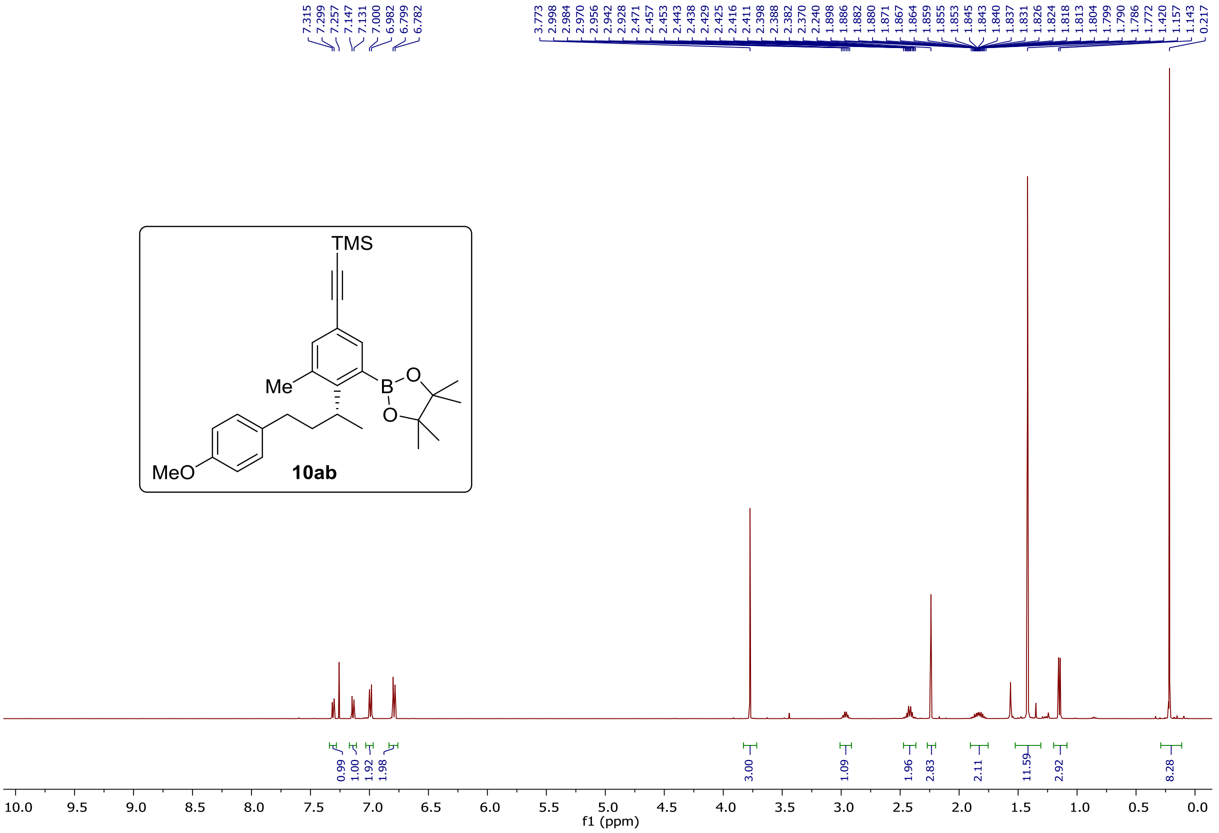
7.315
7.299
7.257
7.147
7.131
7.000
6.982
6.799
6.782
3.773
2.998
2.984
2.970
2.956
2.942
2.928
2.471
2.457
2.453
2.443
2.438
2.429
2.425
2.416
2.411
2.398
2.388
2.382
2.370
2.240
1.898
1.886
1.882
1.880
1.871
1.867
1.864
1.859
1.855
1.853
1.845
1.843
1.840
1.837
1.831
1.826
1.824
1.818
1.813
1.804
1.799
1.790
1.786
1.772
1.420
1.157
1.143
0.217
TMS
Me
B
O
O
MeO
10ab
0.99
1.00
1.92
1.98
3.00
1.09
1.96
2.83
2.11
11.59
2.92
8.28
f1 (ppm)

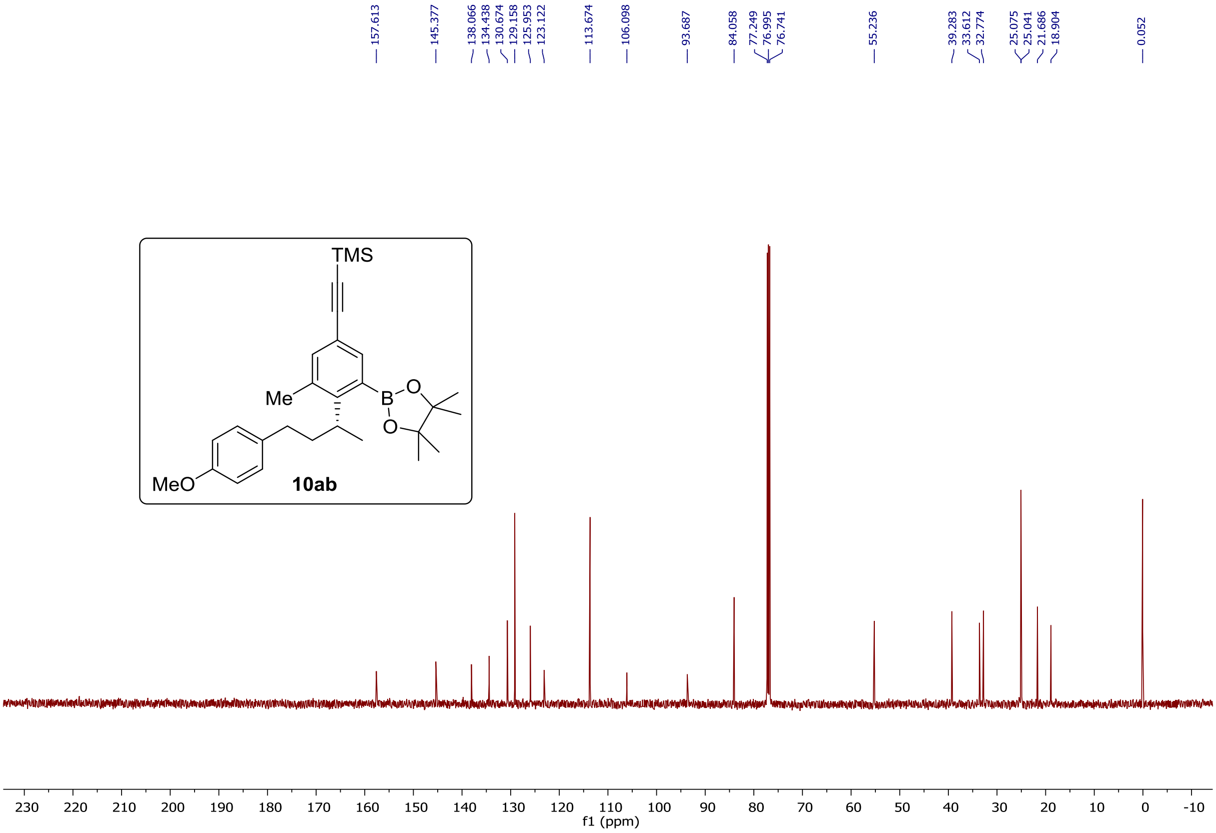
157.613
145.377
138.066
134.438
130.674
129.158
125.953
123.122
113.674
106.098
93.687
84.058
77.249
76.995
76.741
55.236
39.283
33.612
32.774
25.075
25.041
21.686
18.904
0.052
TMS
Me
B
O
O
MeO
10ab
230 220 210 200 190 180 170 160 150 140 130 120 110 100 90 80 70 60 50 40 30 20 10 0 -10
f1 (ppm)

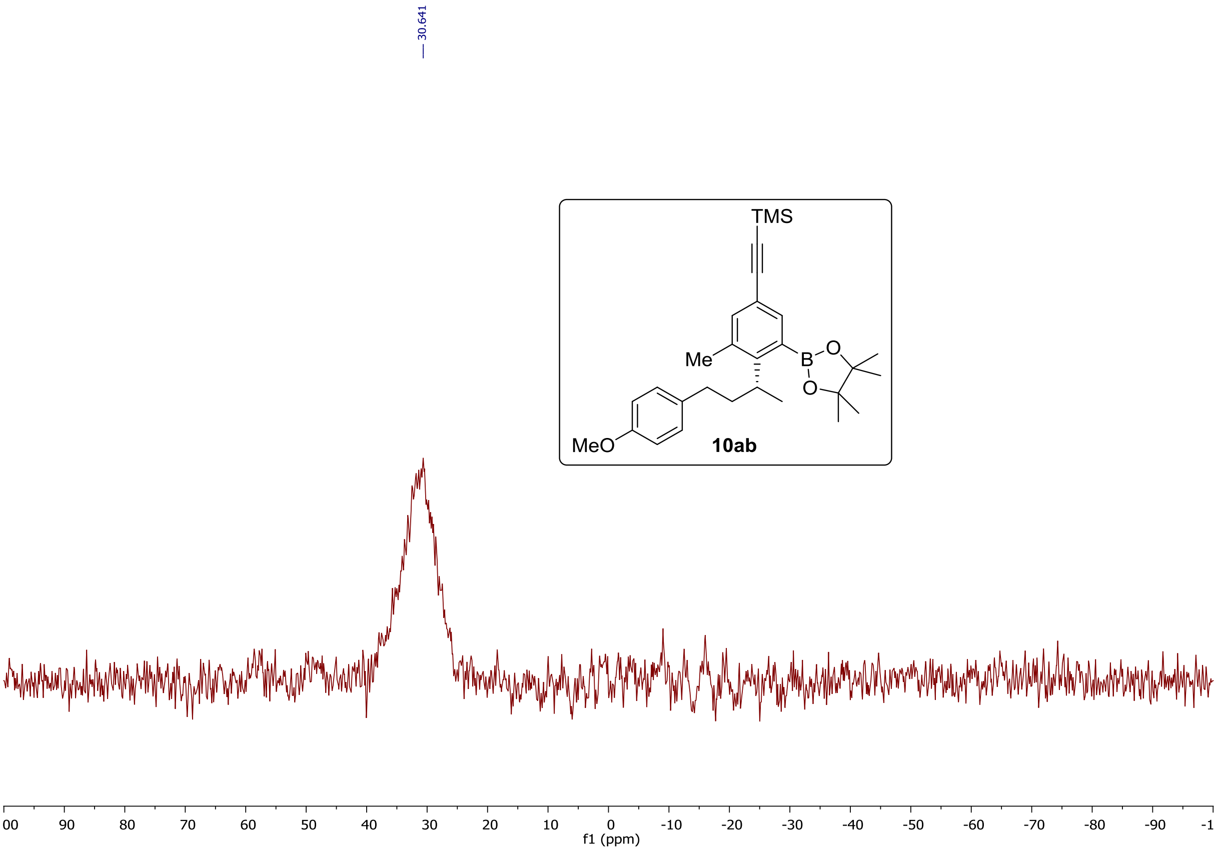
30.641
TMS
Me
B
O
O
MeO
10ab
00 90 80 70 60 50 40 30 20 10 0 -10 -20 -30 -40 -50 -60 -70 -80 -90 -1
f1 (ppm)

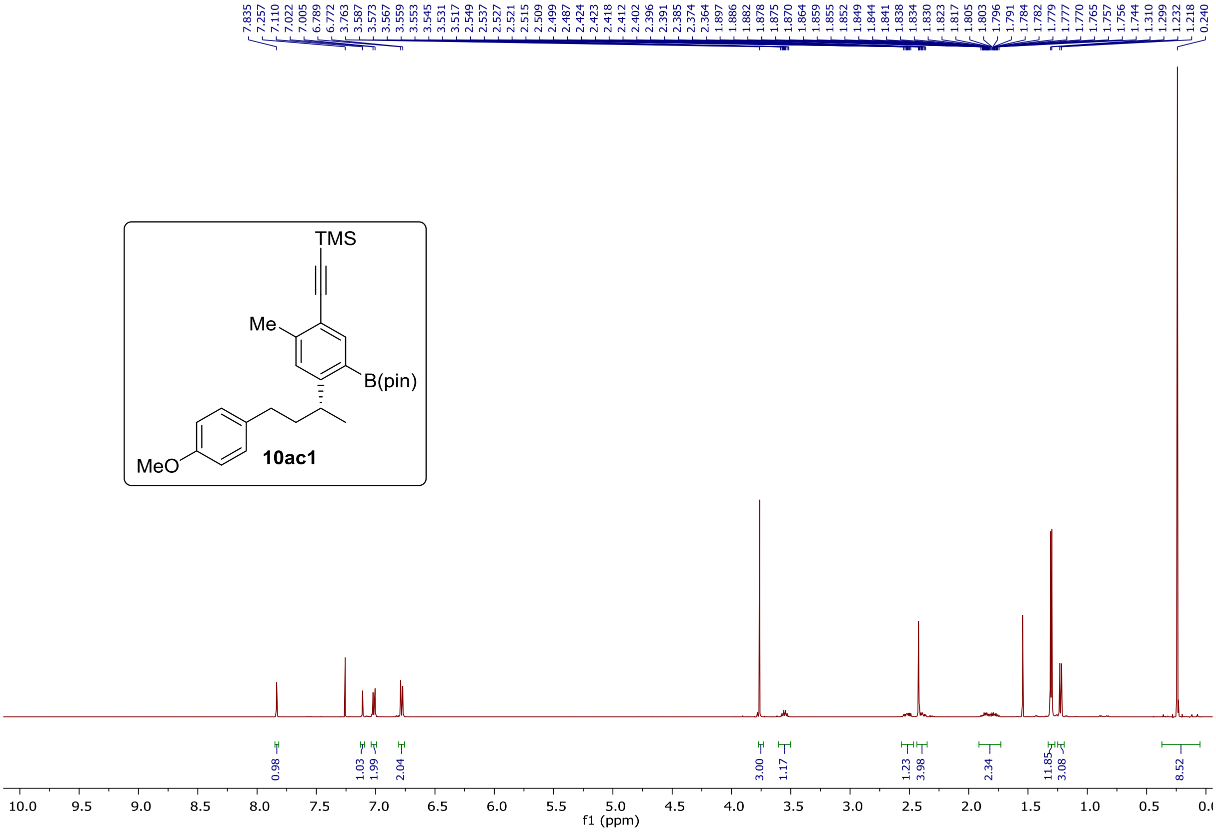
7.835
7.257
7.110
7.022
7.005
6.789
6.772
3.763
3.587
3.573
3.567
3.559
3.553
3.545
3.531
3.517
2.549
2.537
2.527
2.521
2.515
2.509
2.499
2.487
2.424
2.423
2.418
2.412
2.402
2.396
2.391
2.385
2.374
2.364
1.897
1.886
1.882
1.878
1.875
1.870
1.864
1.859
1.855
1.852
1.849
1.844
1.841
1.838
1.834
1.830
1.823
1.817
1.805
1.803
1.796
1.791
1.784
1.782
1.779
1.777
1.770
1.765
1.757
1.756
1.744
1.310
1.299
1.232
1.218
0.240
TMS
Me
B(pin)
MeO
10ac1
0.98
1.03
1.99
2.04
3.00
1.17
1.23
3.98
2.34
11.85
3.08
8.52
10.0
9.5
9.0
8.5
8.0
7.5
7.0
6.5
6.0
5.5
5.0
4.5
4.0
3.5
3.0
2.5
2.0
1.5
1.0
0.5
f1 (ppm)

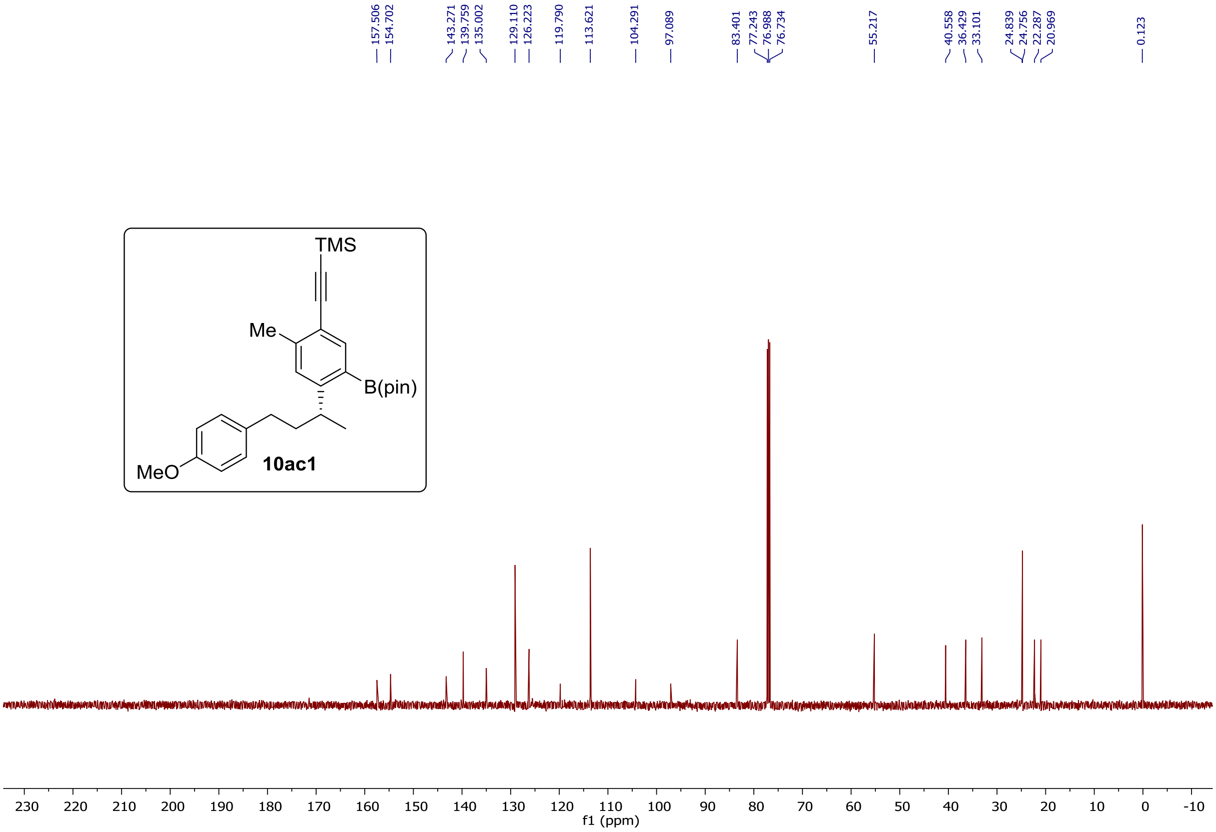
157.506
154.702
143.271
139.759
135.002
129.110
126.223
119.790
113.621
104.291
97.089
83.401
77.243
76.988
76.734
55.217
40.558
36.429
33.101
24.839
24.756
22.287
20.969
0.123
TMS
Me
B(pin)
MeO
10ac1
230
220
210
200
190
180
170
160
150
140
130
120
110
100
90
80
70
60
50
40
30
20
10
0
-10
f1 (ppm)

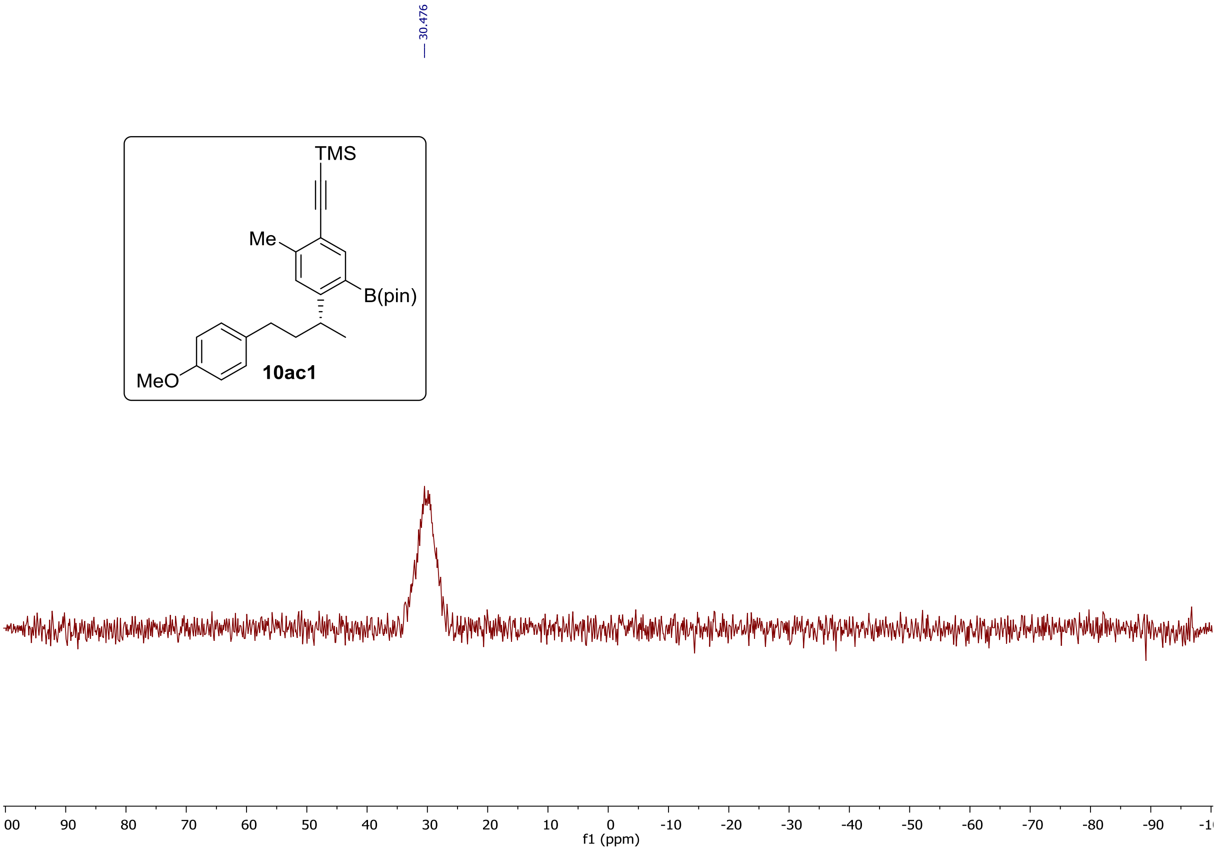
30.476
TMS
Me
B(pin)
MeO
10ac1
f1 (ppm)

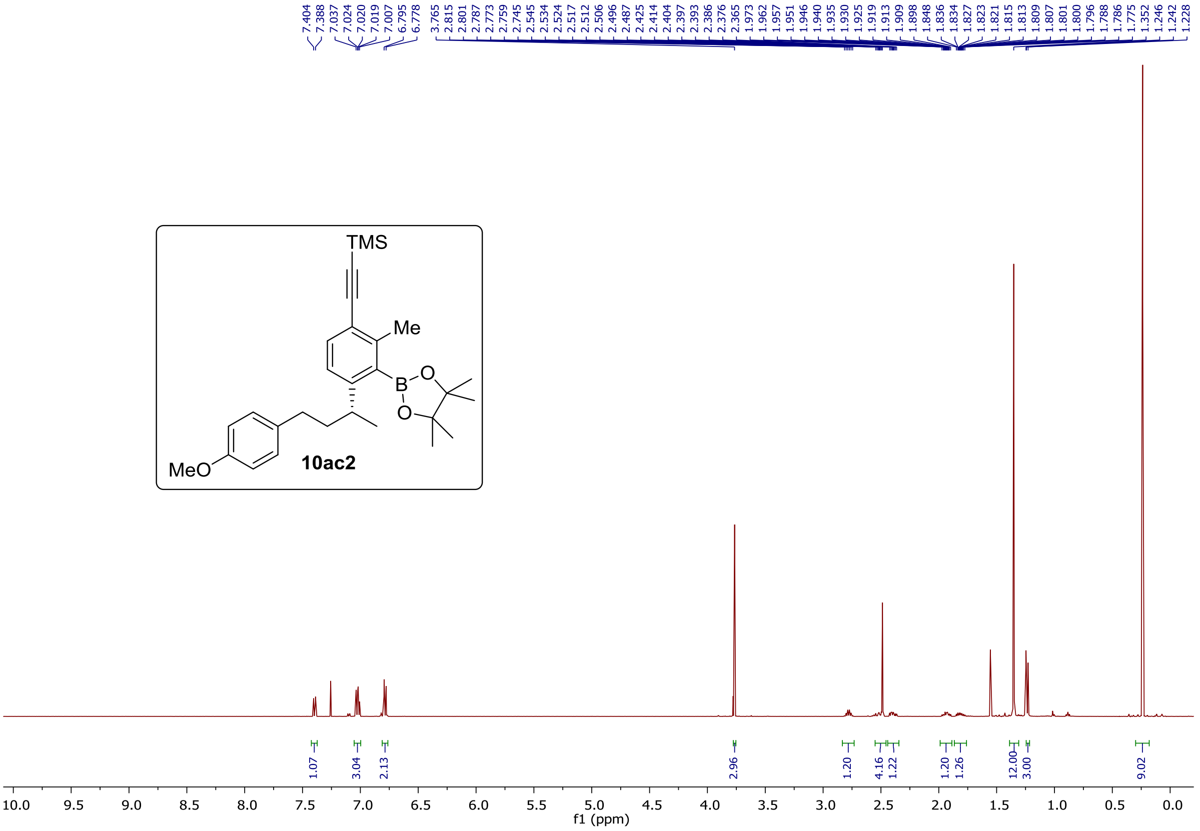
TMS
Me
B
O
O
MeO
10ac2
1.07
3.04
2.13
2.96
1.20
4.16
1.22
1.20
1.26
12.00
3.00
9.02
f1 (ppm)

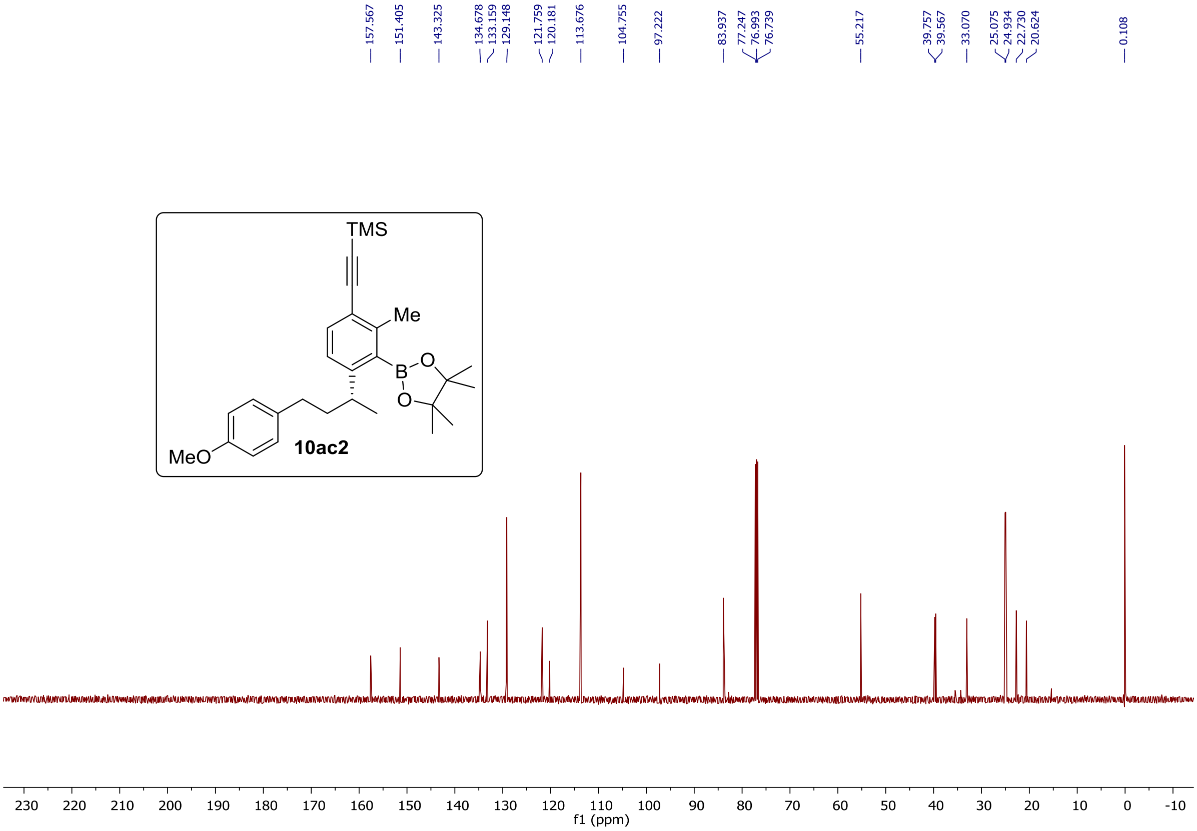
157.567
151.405
143.325
134.678
133.159
129.148
121.759
120.181
113.676
104.755
97.222
83.937
77.247
76.993
76.739
55.217
39.757
39.567
33.070
25.075
24.934
22.730
20.624
0.108
TMS
Me
B
O
O
MeO
10ac2
230 220 210 200 190 180 170 160 150 140 130 120 110 100 90 80 70 60 50 40 30 20 10 0 -10
f1 (ppm)

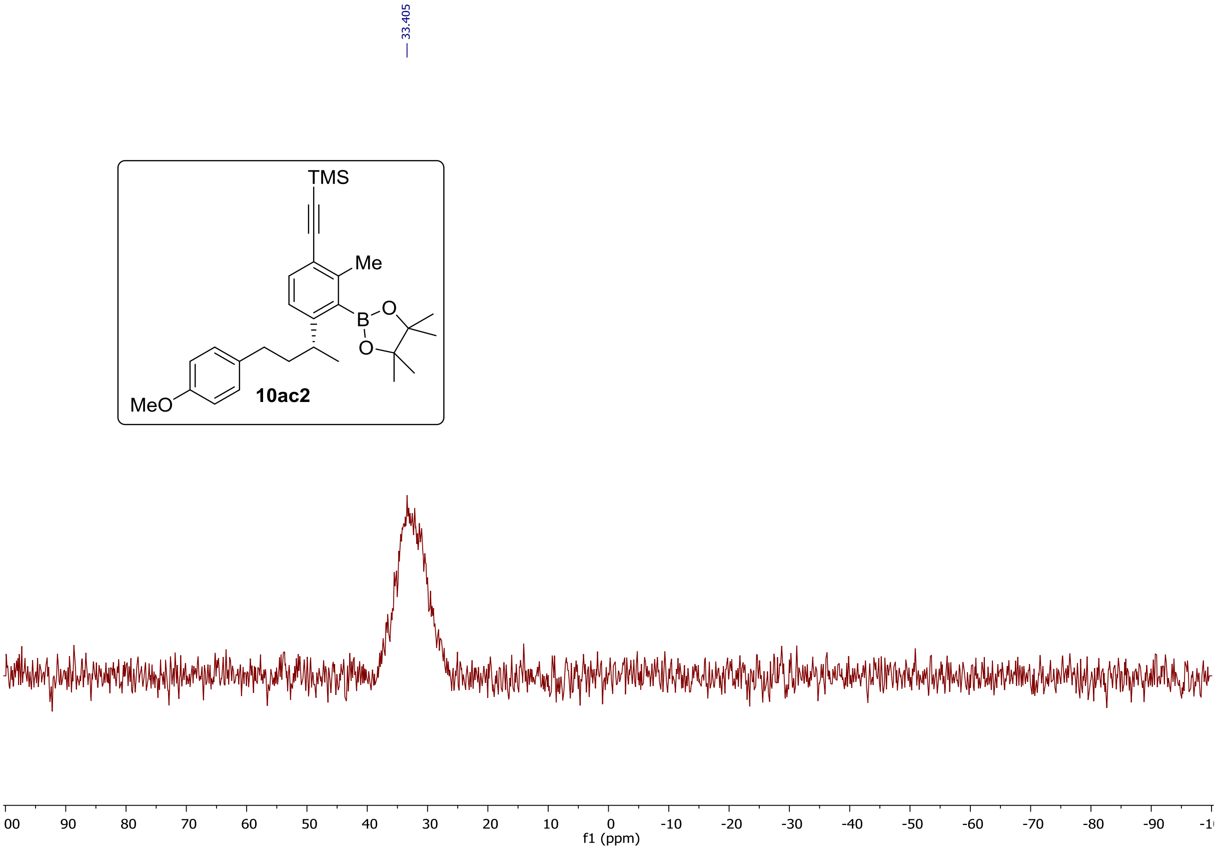
33.405
TMS
Me
B
O
O
MeO
10ac2
90 80 70 60 50 40 30 20 10 0 -10 -20 -30 -40 -50 -60 -70 -80 -90
f1 (ppm)

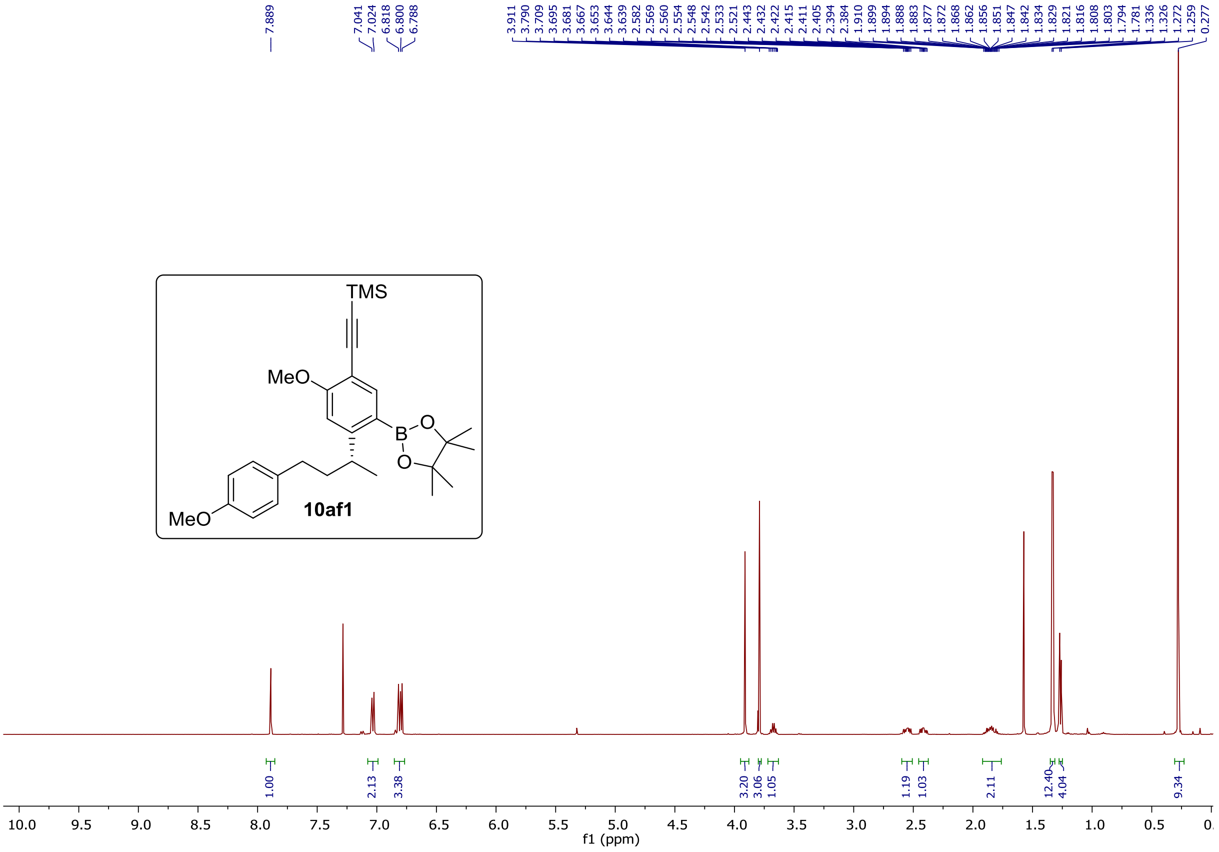
7.889
7.041
7.024
6.818
6.800
6.788
3.911
3.790
3.709
3.695
3.681
3.667
3.653
3.644
3.639
2.582
2.569
2.560
2.554
2.548
2.542
2.533
2.521
2.443
2.432
2.422
2.415
2.411
2.405
2.394
2.384
1.910
1.899
1.894
1.888
1.883
1.877
1.872
1.868
1.862
1.856
1.851
1.847
1.842
1.834
1.829
1.821
1.816
1.808
1.803
1.794
1.781
1.336
1.326
1.272
1.259
0.277
TMS
MeO
B
O
O
MeO
10af1
1.00
2.13
3.38
3.20
3.06
1.05
1.19
1.03
2.11
12.40
4.04
9.34
f1 (ppm)

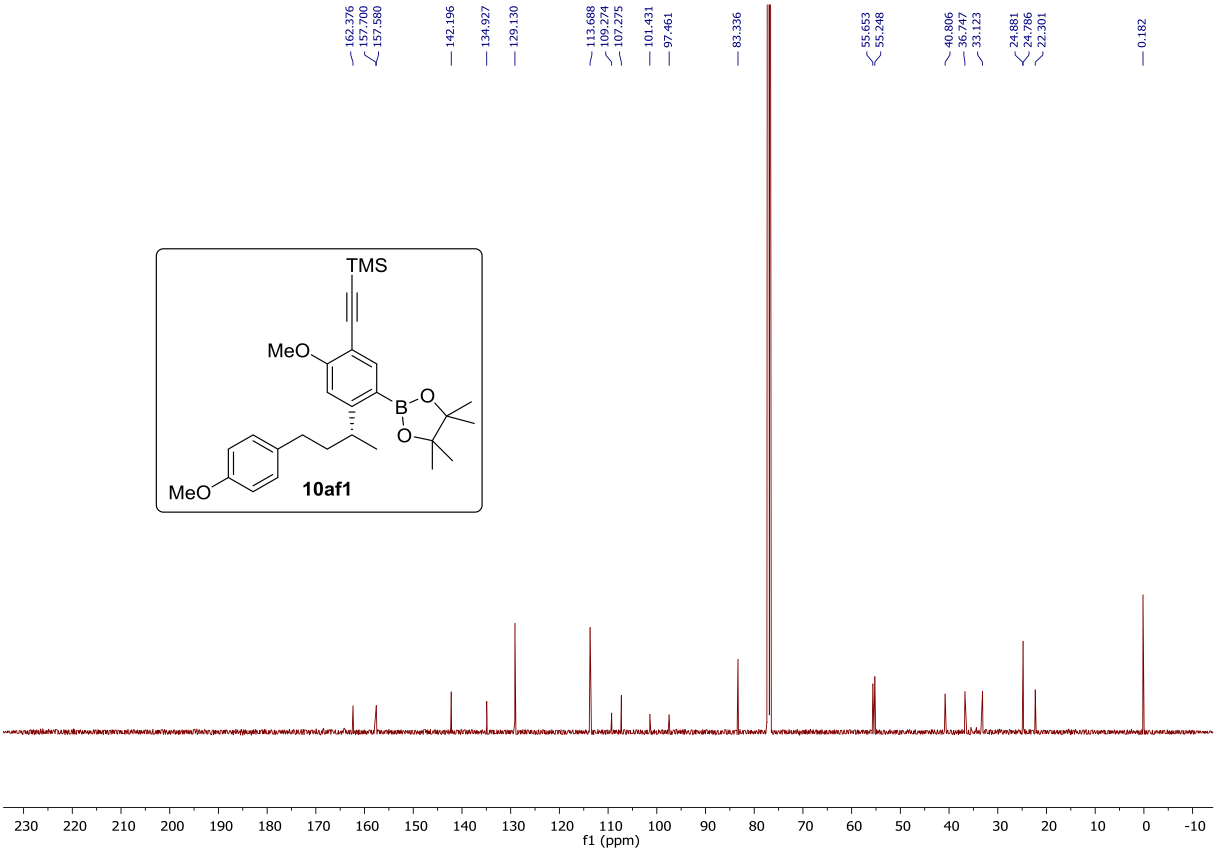
162.376
157.700
157.580
142.196
134.927
129.130
113.688
109.274
107.275
101.431
97.461
83.336
55.653
55.248
40.806
36.747
33.123
24.881
24.786
22.301
0.182
TMS
MeO
B
O
O
MeO
10af1
f1 (ppm)

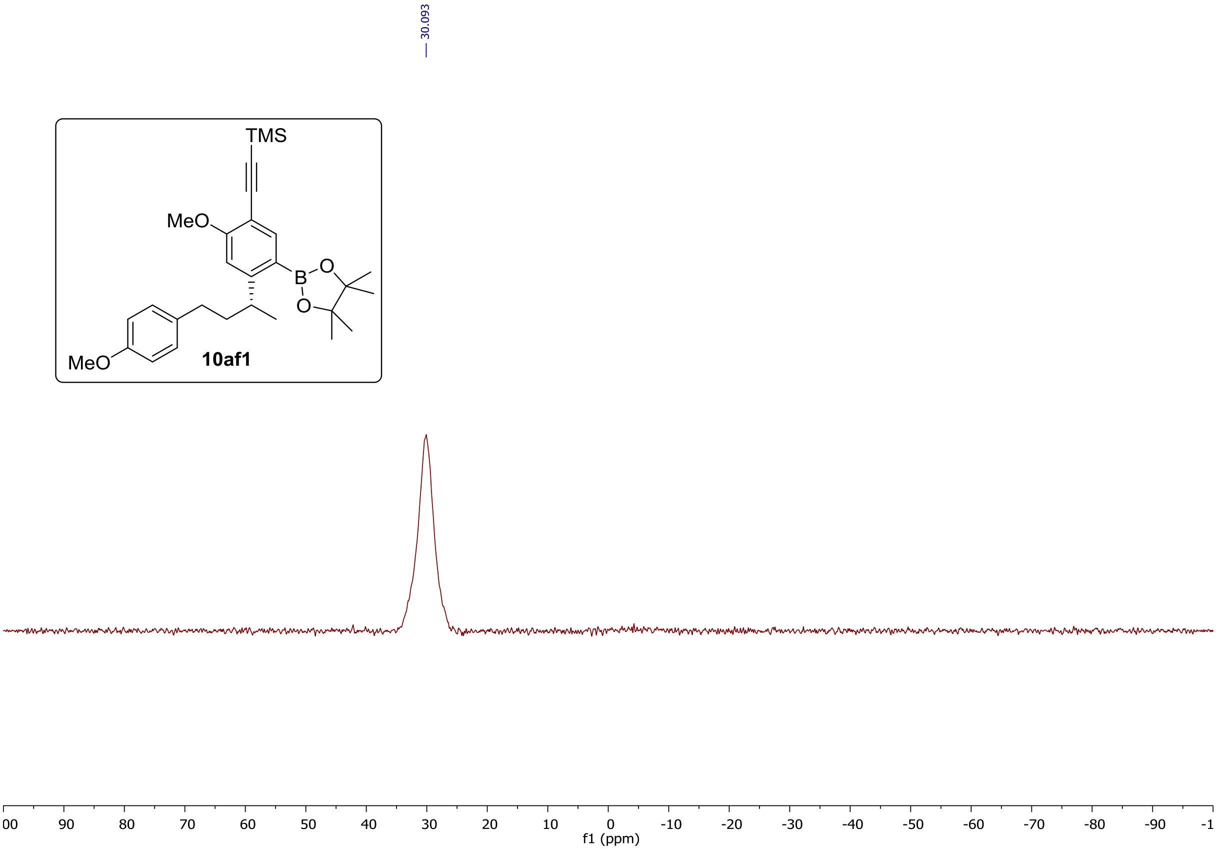
30.093
TMS
MeO
B
O
O
MeO
10af1
00 90 80 70 60 50 40 30 20 10 0 -10 -20 -30 -40 -50 -60 -70 -80 -90 -1
f1 (ppm)

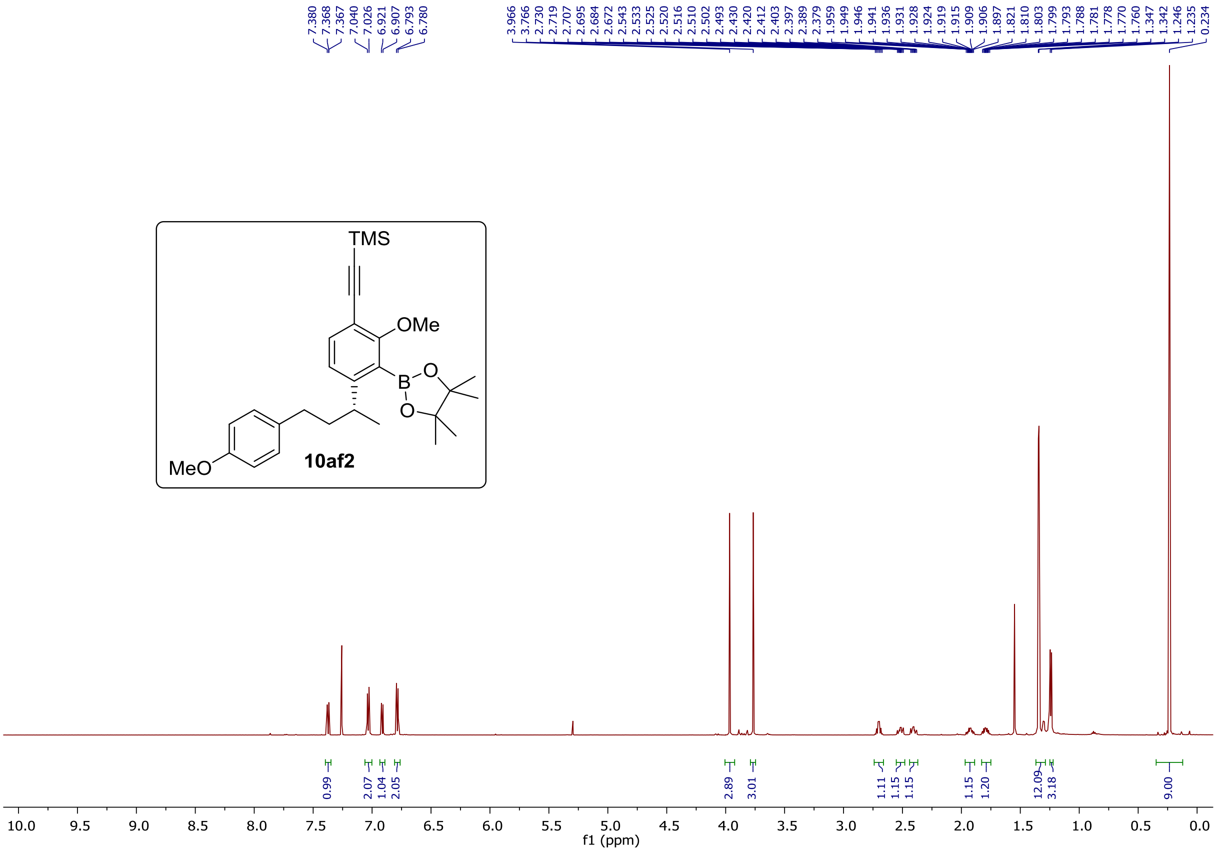
7.380 7.368 7.367 7.040 7.026 6.921 6.907 6.793 6.780
3.966 3.766 2.730 2.719 2.707 2.695 2.684 2.672 2.543 2.533 2.525 2.520 2.516 2.510 2.502 2.493 2.430 2.420 2.412 2.403 2.397 2.389 2.379 1.959 1.949 1.946 1.941 1.936 1.931 1.928 1.924 1.919 1.915 1.909 1.906 1.897 1.821 1.810 1.803 1.799 1.793 1.788 1.781 1.778 1.770 1.760 1.347 1.342 1.246 1.235 0.234
TMS
OMe
B
O
O
MeO
10af2
0.99 2.07 1.04 2.05 2.89 3.01 1.11 1.15 1.15 1.15 1.20 12.09 3.18 9.00
10.0 9.5 9.0 8.5 8.0 7.5 7.0 6.5 6.0 5.5 5.0 4.5 4.0 3.5 3.0 2.5 2.0 1.5 1.0 0.5 0.0
f1 (ppm)

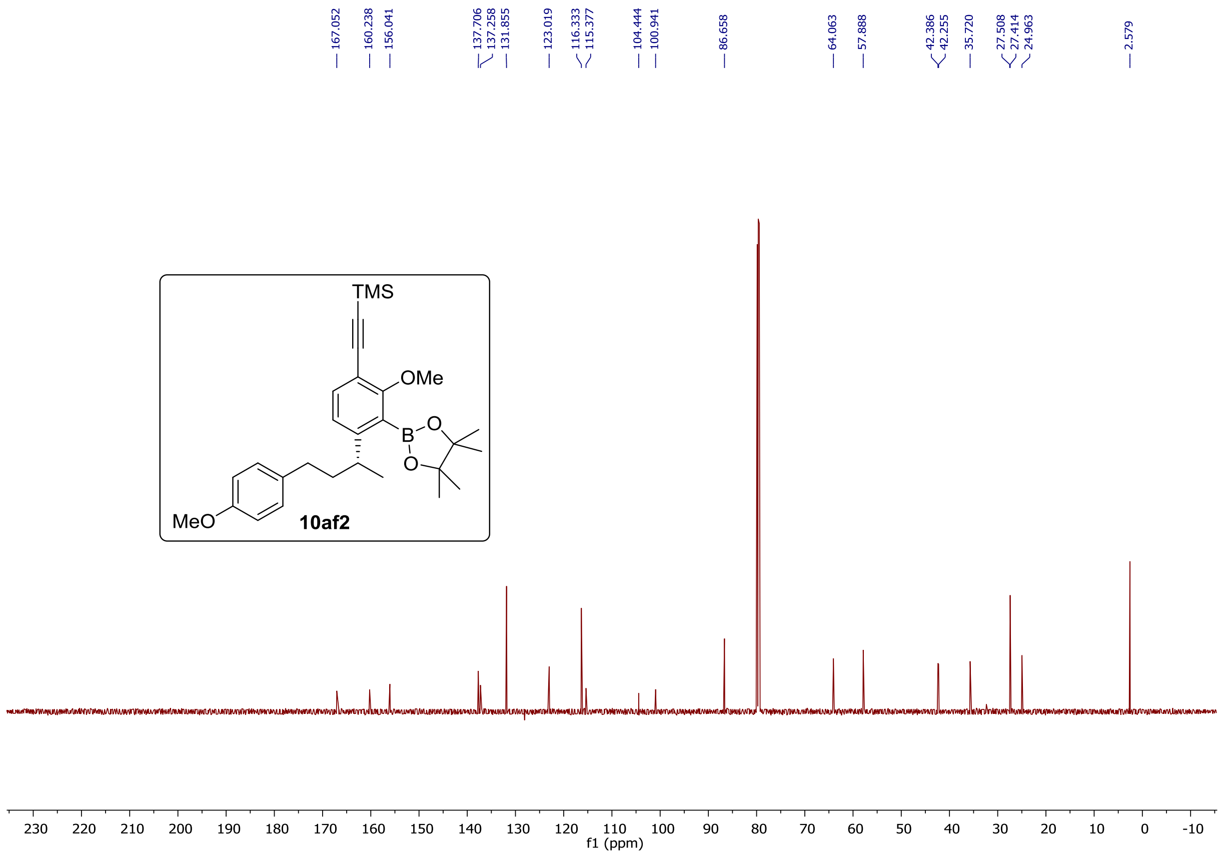
167.052
160.238
156.041
137.706
137.258
131.855
123.019
116.333
115.377
104.444
100.941
86.658
64.063
57.888
42.386
42.255
35.720
27.508
27.414
24.963
2.579
TMS
OMe
B
O
MeO
10af2
230 220 210 200 190 180 170 160 150 140 130 120 110 100 90 80 70 60 50 40 30 20 10 0 -10
f1 (ppm)

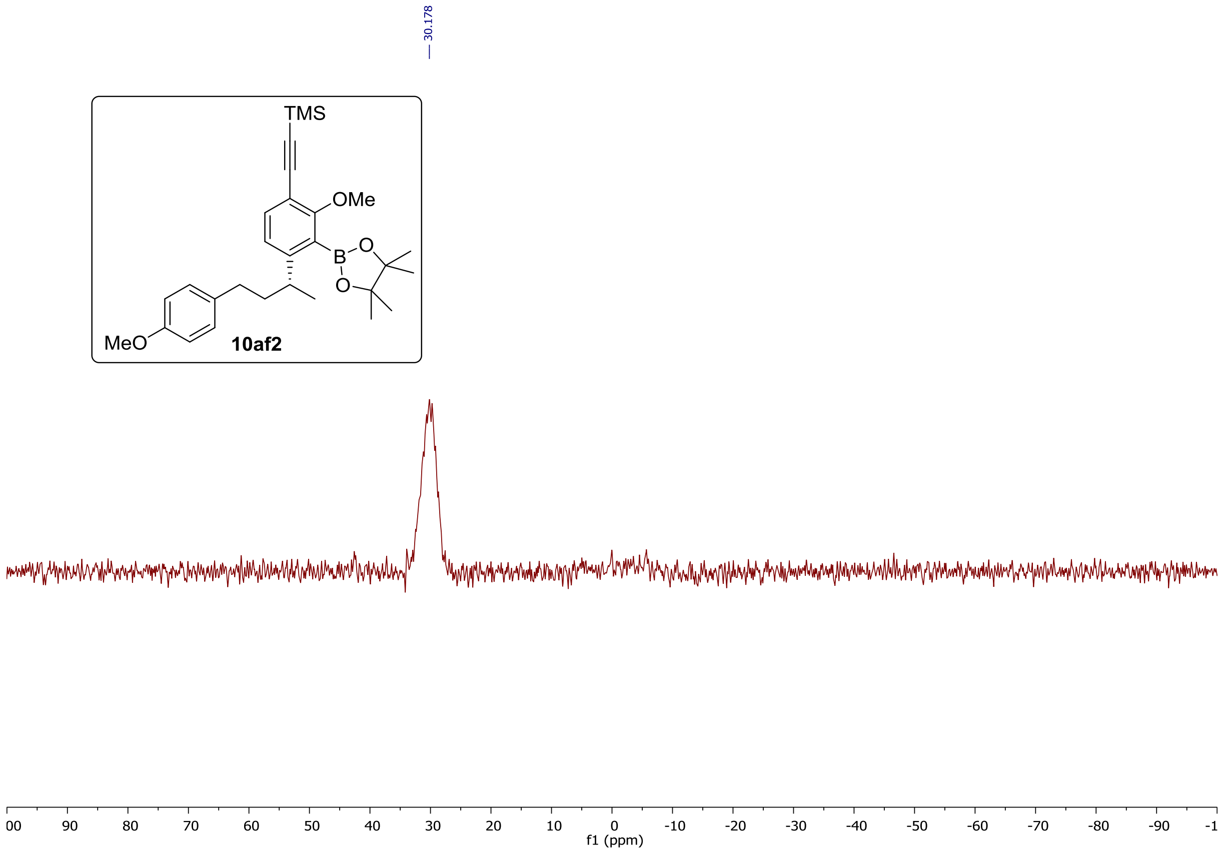
30.178
TMS
OMe
B
O
MeO
10af2
00 90 80 70 60 50 40 30 20 10 0 -10 -20 -30 -40 -50 -60 -70 -80 -90 -1
f1 (ppm)

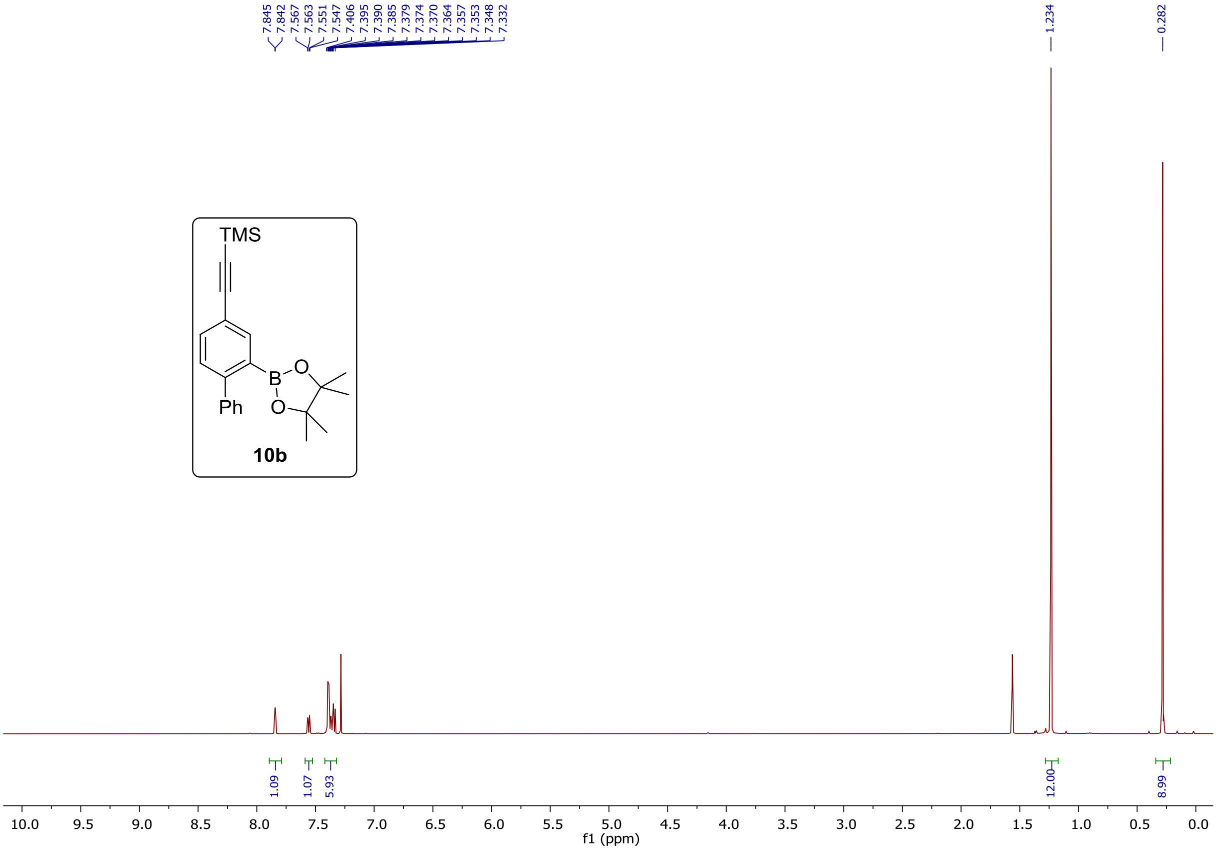
7.845
7.842
7.567
7.563
7.551
7.547
7.406
7.395
7.390
7.385
7.379
7.374
7.370
7.364
7.357
7.353
7.348
7.332
1.234
0.282
TMS
B
O
O
Ph
10b
1.09
1.07
5.93
12.00
8.99
10.0 9.5 9.0 8.5 8.0 7.5 7.0 6.5 6.0 5.5 5.0 4.5 4.0 3.5 3.0 2.5 2.0 1.5 1.0 0.5 0.0
f1 (ppm)

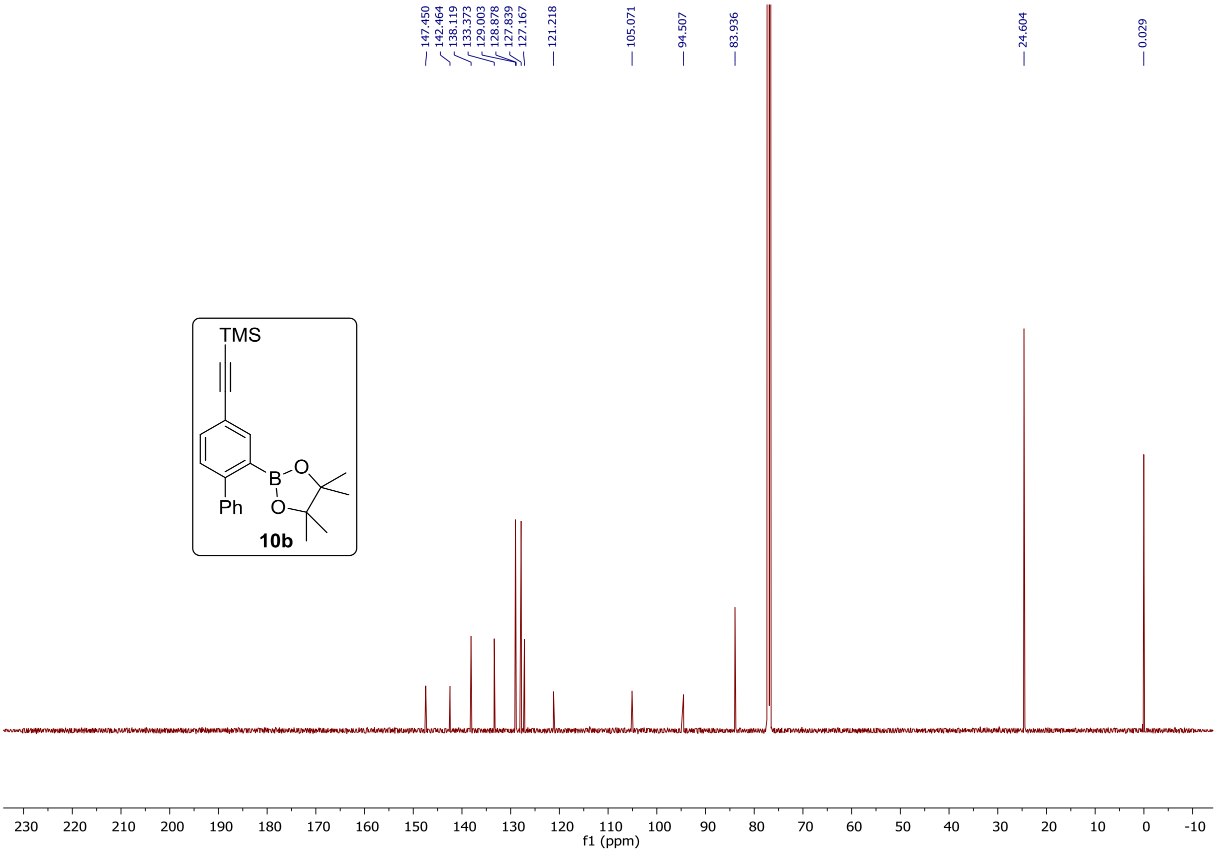
147.450
142.464
138.119
133.373
129.003
128.878
127.839
127.167
121.218
105.071
94.507
83.936
24.604
0.029
TMS
B
O
O
Ph
10b
230 220 210 200 190 180 170 160 150 140 130 120 110 100 90 80 70 60 50 40 30 20 10 0 -10
f1 (ppm)

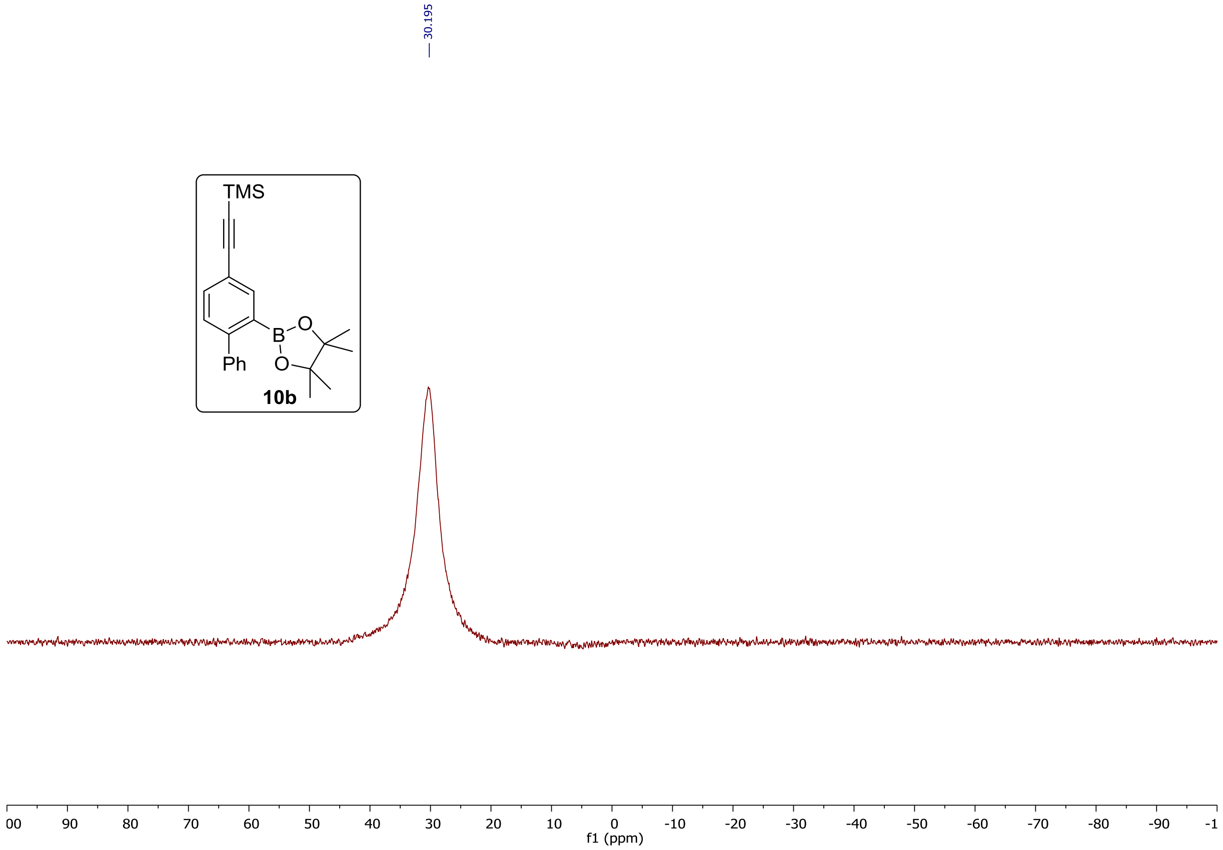

30.195
TMS
B
O
O
Ph
10b
f1 (ppm)

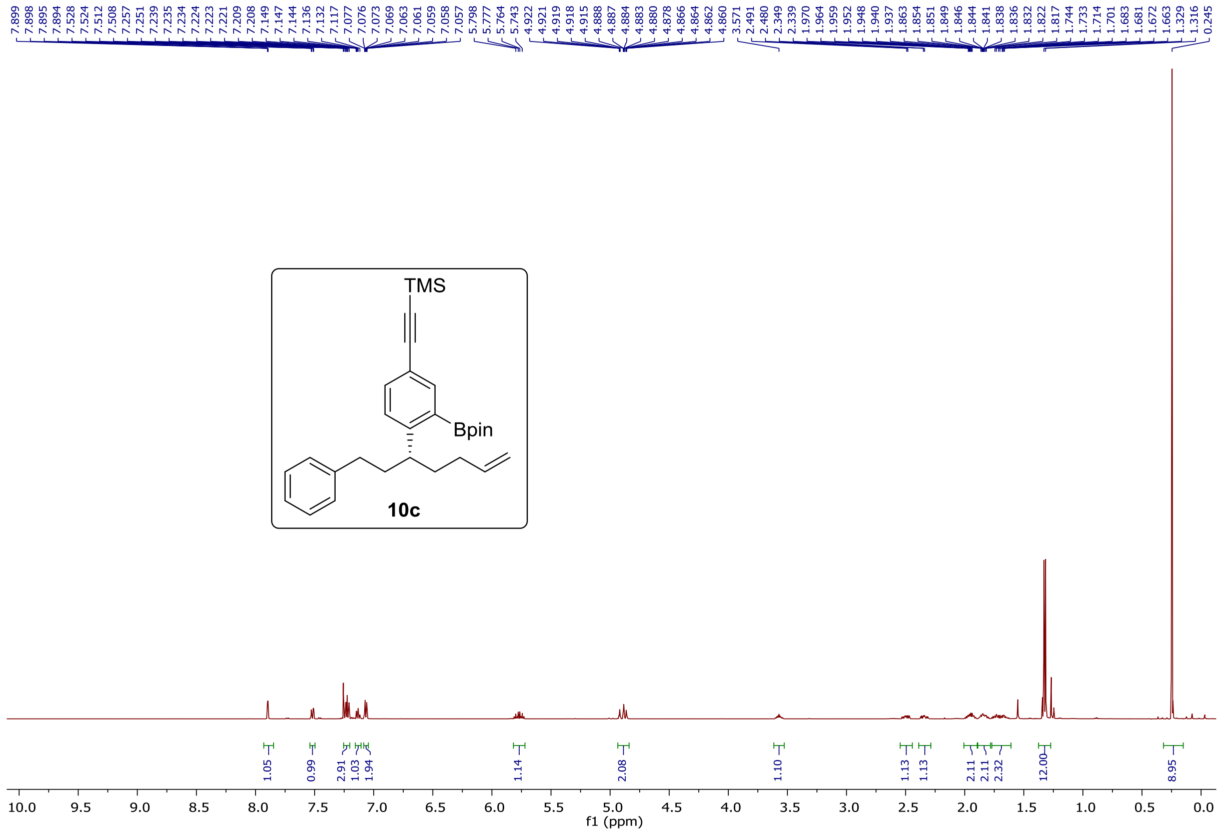

7.899 7.898 7.895 7.894 7.528 7.524 7.512 7.508 7.257 7.251 7.239 7.235 7.234 7.224 7.223 7.221 7.209 7.208 7.149 7.147 7.144 7.136 7.132 7.117 7.077 7.076 7.073 7.069 7.063 7.061 7.059 7.058 7.057 5.798 5.777 5.764 5.743 4.922 4.921 4.919 4.918 4.915 4.888 4.887 4.884 4.883 4.880 4.878 4.866 4.864 4.862 4.860 3.571 2.491 2.480 2.349 2.339 1.970 1.964 1.959 1.952 1.948 1.940 1.937 1.863 1.854 1.851 1.849 1.846 1.844 1.841 1.838 1.836 1.832 1.822 1.817 1.744 1.733 1.714 1.701 1.683 1.681 1.672 1.663 1.329 1.316 0.245
TMS
Bpin
10c
1.05 0.99 2.91 1.03 1.94 1.14 2.08 1.10 1.13 1.13 2.11 2.11 2.32 12.00 8.95
f1 (ppm)

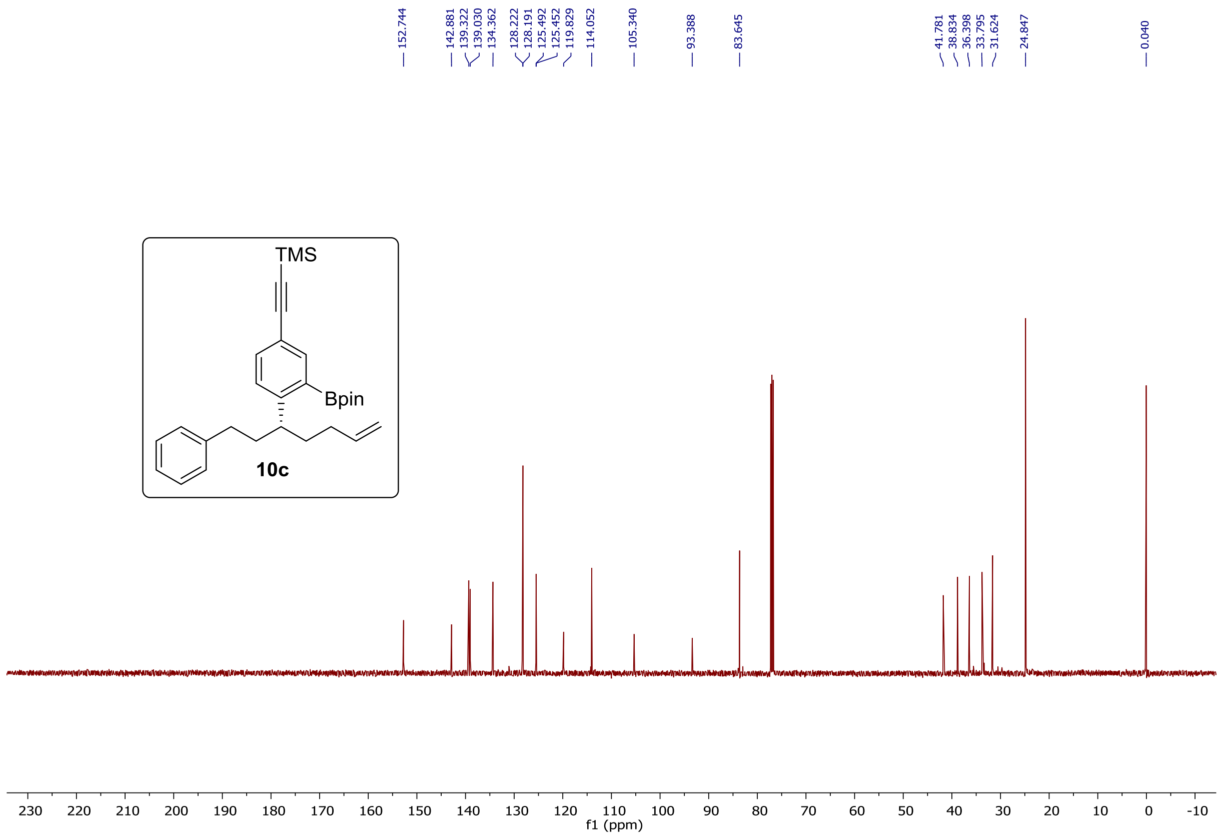
152.744
142.881
139.322
139.030
134.362
128.222
128.191
125.492
125.452
119.829
114.052
105.340
93.388
83.645
41.781
38.834
36.398
33.795
31.624
24.847
0.040
TMS
Bpin
10c
230 220 210 200 190 180 170 160 150 140 130 120 110 100 90 80 70 60 50 40 30 20 10 0 -10
f1 (ppm)

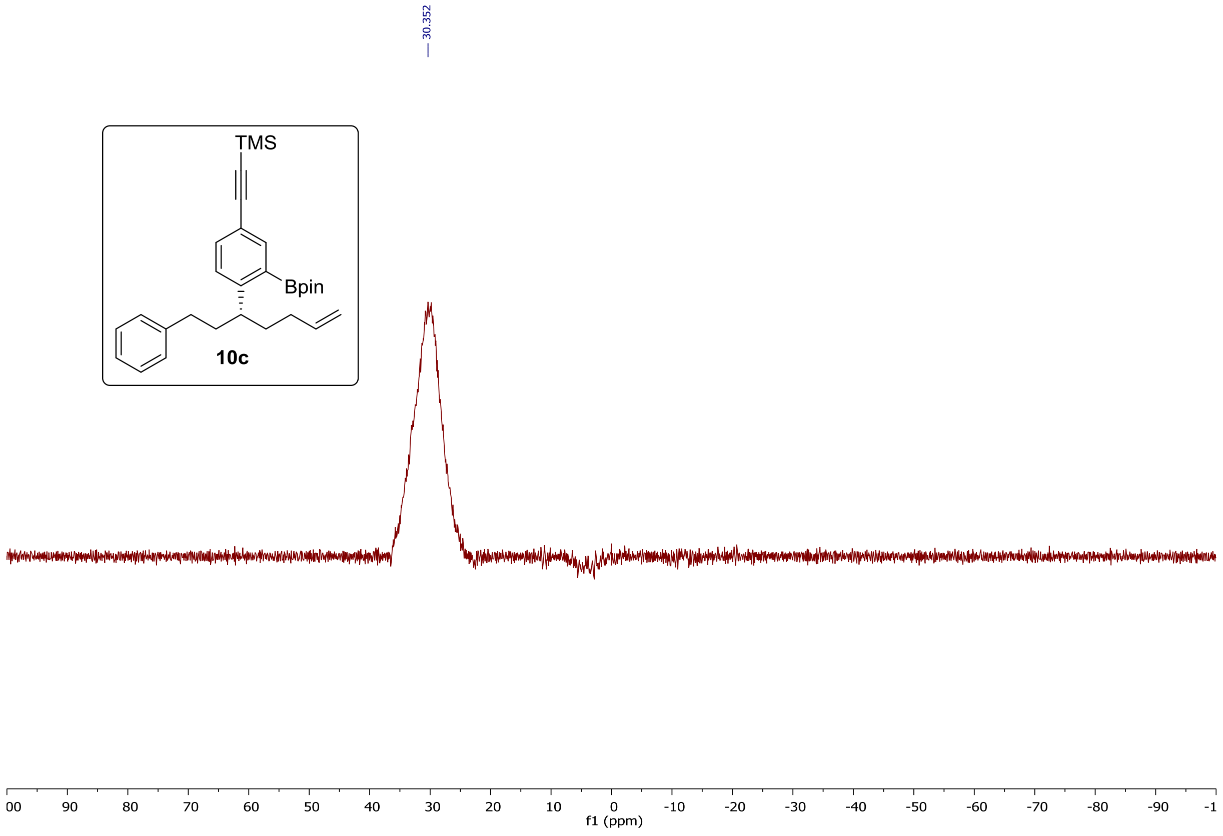
30.352
TMS
Bpin
10c
00 90 80 70 60 50 40 30 20 10 0 -10 -20 -30 -40 -50 -60 -70 -80 -90 -1
f1 (ppm)

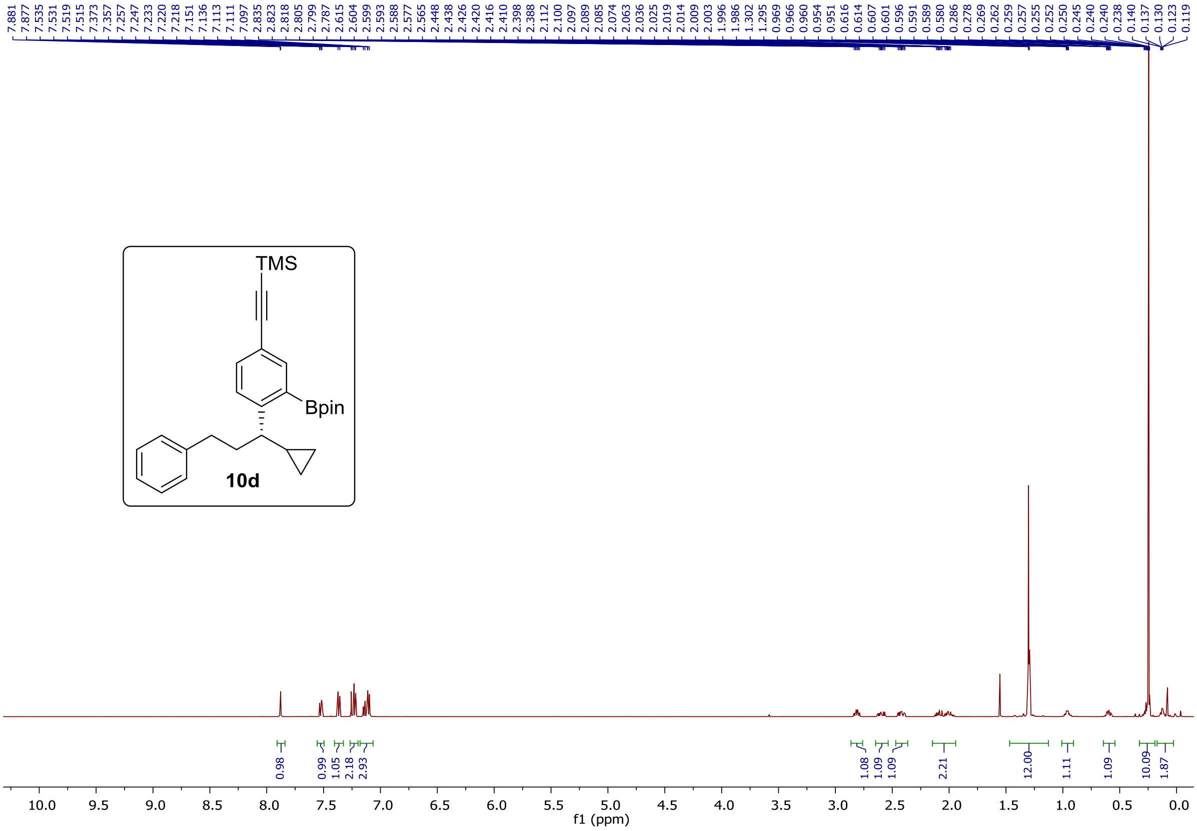
7.881 7.877 7.535 7.531 7.519 7.515 7.373 7.357 7.257 7.247 7.233 7.220 7.218 7.151 7.136 7.113 7.111 7.097 2.835 2.823 2.818 2.805 2.799 2.787 2.615 2.604 2.599 2.593 2.588 2.577 2.565 2.448 2.438 2.426 2.420 2.416 2.410 2.398 2.388 2.112 2.100 2.097 2.089 2.085 2.074 2.063 2.036 2.025 2.019 2.014 2.009 2.003 1.996 1.986 1.302 1.295 0.969 0.966 0.960 0.954 0.951 0.616 0.614 0.607 0.601 0.596 0.591 0.589 0.580 0.286 0.278 0.269 0.262 0.259 0.257 0.255 0.252 0.250 0.245 0.240 0.240 0.238 0.140 0.137 0.130 0.123 0.119
TMS
Bpin
10d
0.98
0.99
1.05
2.18
2.93
1.08
1.09
1.09
2.21
12.00
1.11
1.09
10.09
1.87
10.0 9.5 9.0 8.5 8.0 7.5 7.0 6.5 6.0 5.5 5.0 4.5 4.0 3.5 3.0 2.5 2.0 1.5 1.0 0.5 0.0
f1 (ppm)

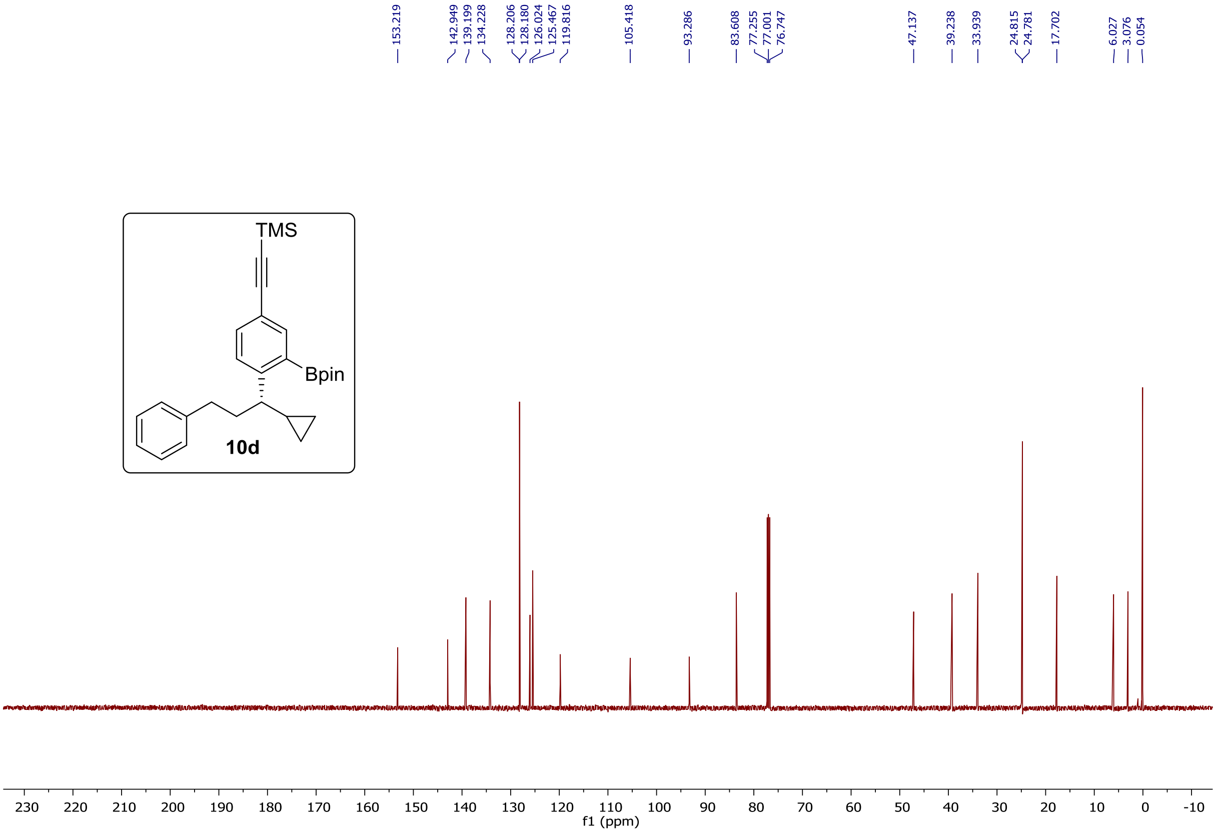
153.219
142.949 139.199 134.228
128.206 128.180 126.024 125.467 119.816
105.418
93.286
83.608 77.255 77.001 76.747
47.137
39.238
33.939
24.815 24.781
17.702
6.027 3.076 0.054
TMS
Bpin
10d
230 220 210 200 190 180 170 160 150 140 130 120 110 100 90 80 70 60 50 40 30 20 10 0 -10
f1 (ppm)

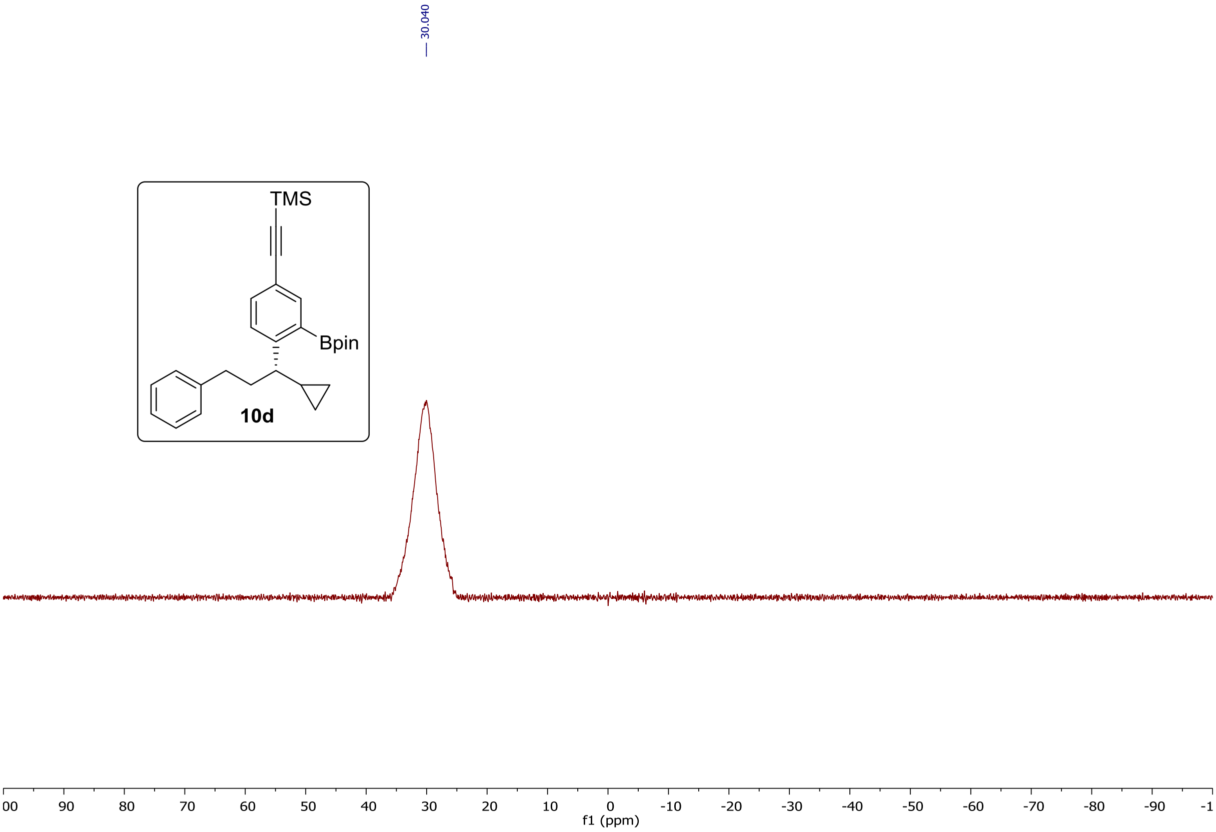
30.040
TMS
Bpin
10d
f1 (ppm)

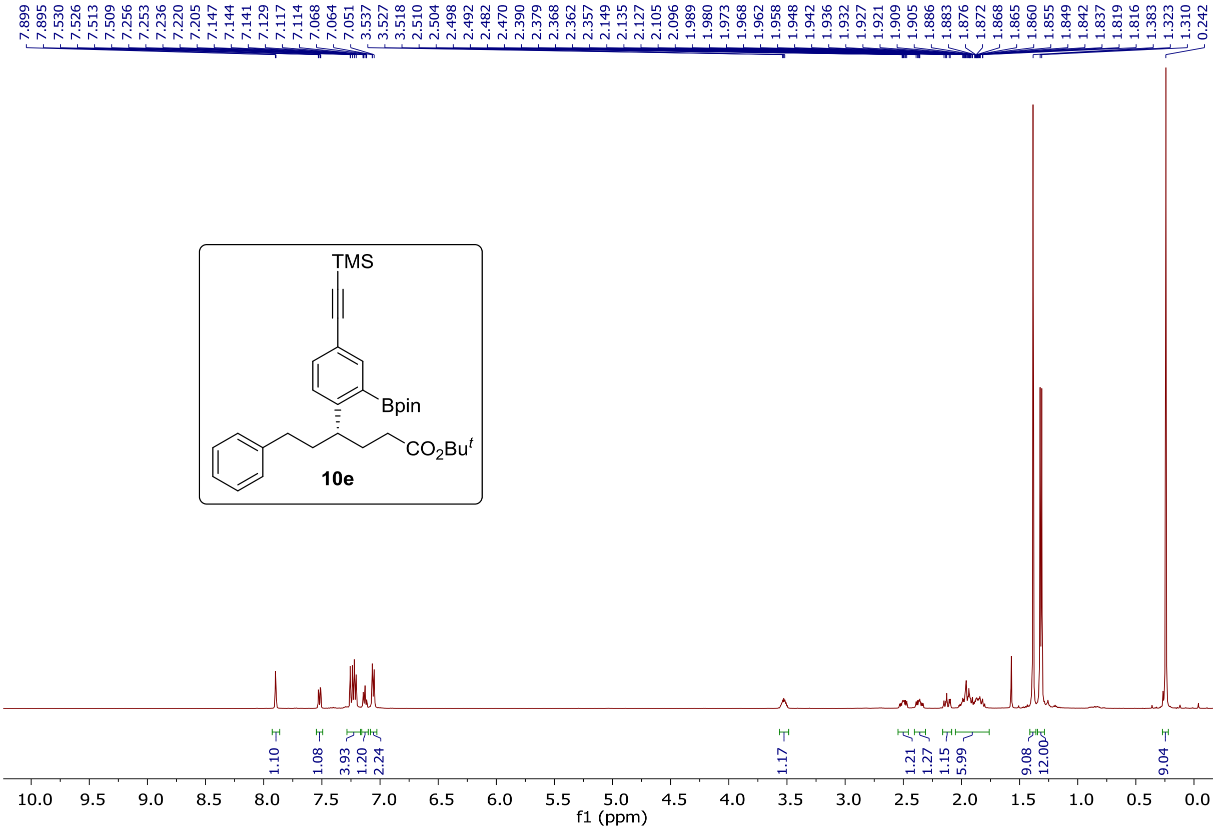
7.899
7.895
7.530
7.526
7.513
7.509
7.256
7.253
7.236
7.220
7.205
7.147
7.144
7.141
7.129
7.117
7.114
7.068
7.064
7.051
3.537
3.527
3.518
2.510
2.504
2.498
2.492
2.482
2.470
2.390
2.379
2.368
2.362
2.357
2.149
2.135
2.127
2.105
2.096
1.989
1.980
1.973
1.968
1.962
1.958
1.948
1.942
1.936
1.932
1.927
1.921
1.909
1.905
1.886
1.883
1.876
1.872
1.868
1.865
1.860
1.855
1.849
1.842
1.837
1.819
1.816
1.383
1.323
1.310
0.242
TMS
Bpin
CO_2Bu^t
10e
1.10
1.08
3.93
1.20
2.24
1.17
1.21
1.27
1.15
5.99
9.08
12.00
9.04
f1 (ppm)

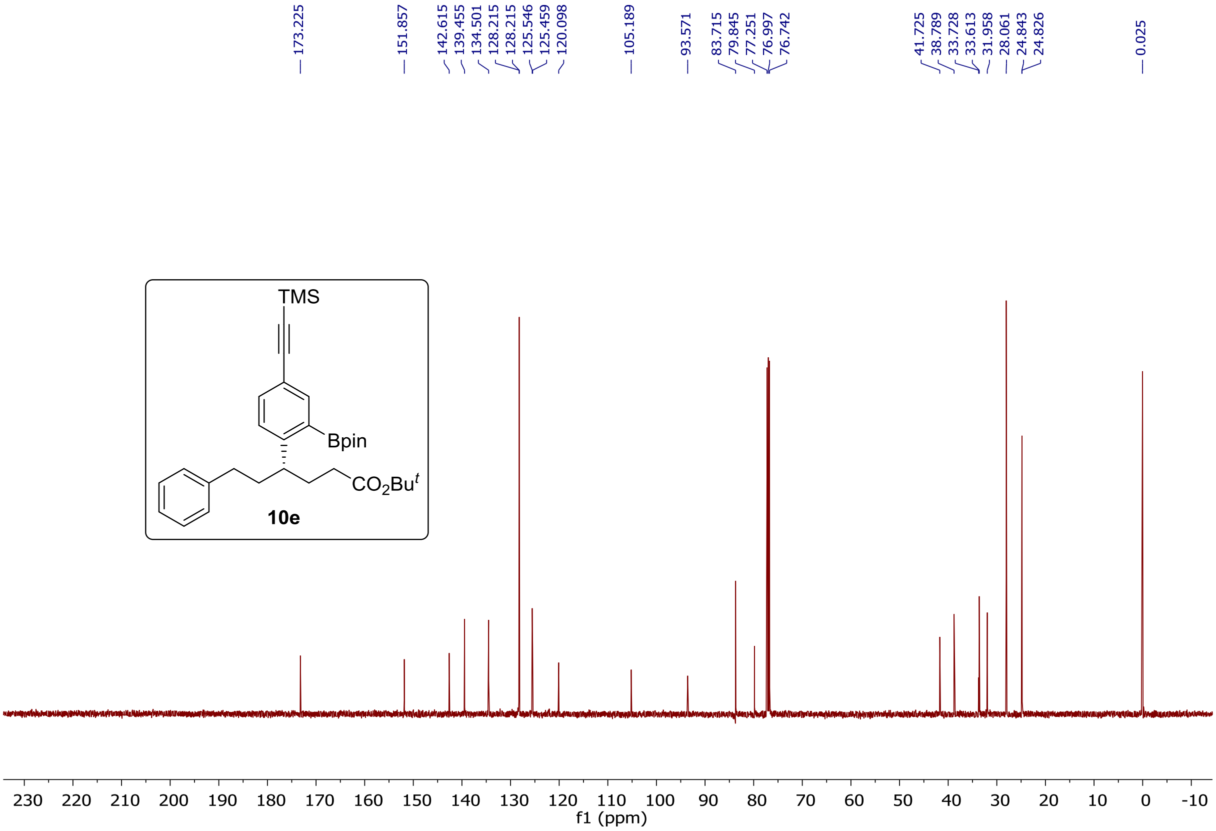
173.225
151.857
142.615
139.455
134.501
128.215
128.215
125.546
125.459
120.098
105.189
93.571
83.715
79.845
77.251
76.997
76.742
41.725
38.789
33.728
33.613
31.958
28.061
24.843
24.826
0.025
TMS
Bpin
CO_2Bu^t
10e
230 220 210 200 190 180 170 160 150 140 130 120 110 100 90 80 70 60 50 40 30 20 10 0 -10
f1 (ppm)

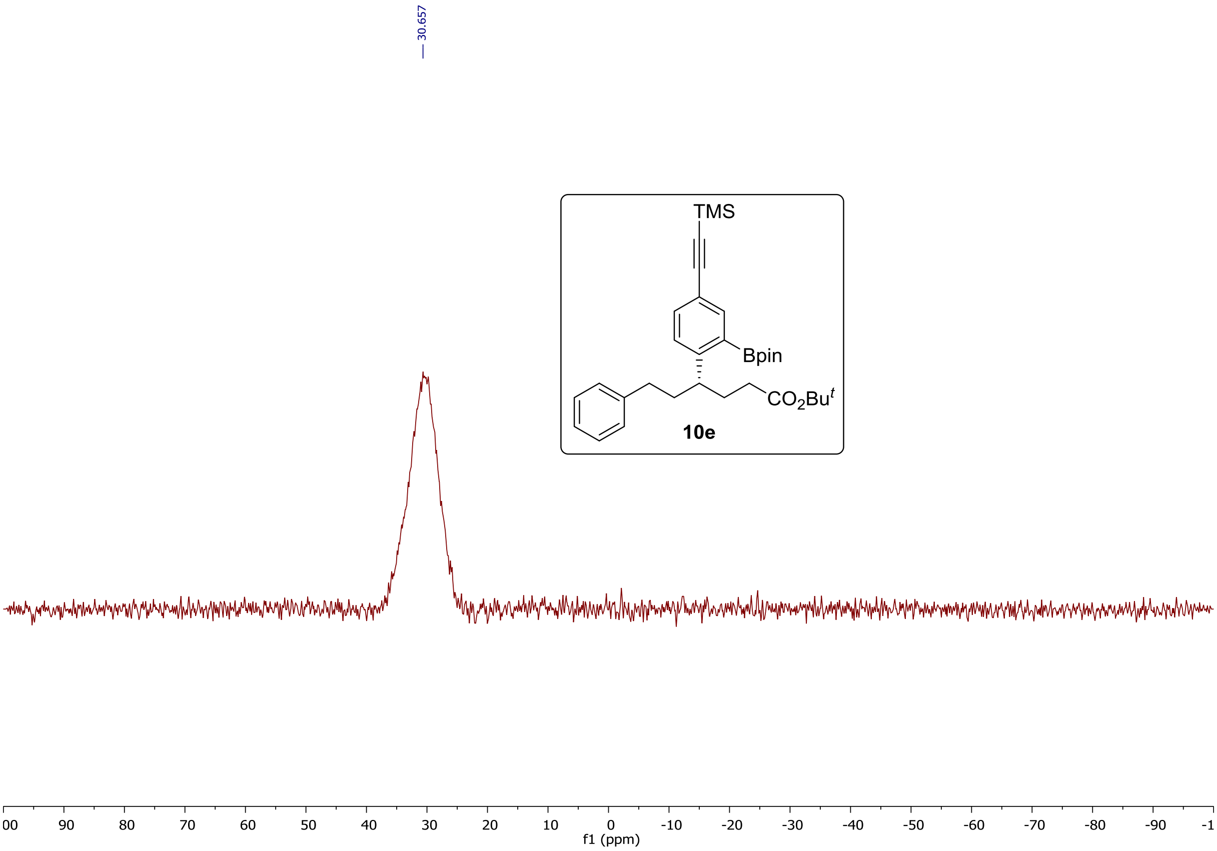
30.657
TMS
Bpin
CO_2Bu^t
10e
00 90 80 70 60 50 40 30 20 10 0 -10 -20 -30 -40 -50 -60 -70 -80 -90 -1
f1 (ppm)

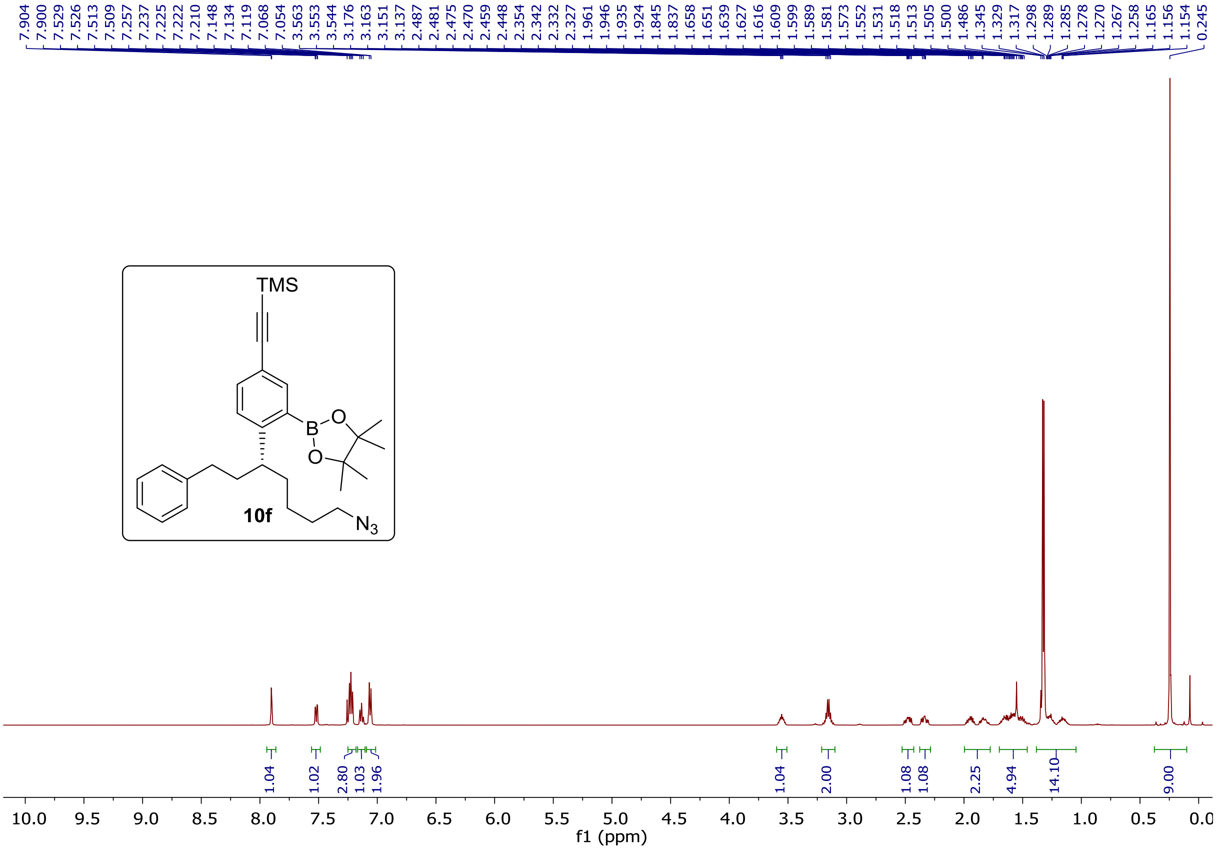
7.904 7.900 7.529 7.526 7.513 7.509 7.257 7.237 7.225 7.222 7.210 7.148 7.134 7.119 7.068 7.054 3.563 3.553 3.544 3.176 3.163 3.151 3.137 2.487 2.481 2.475 2.470 2.459 2.448 2.354 2.342 2.332 2.327 1.961 1.946 1.935 1.924 1.845 1.837 1.658 1.651 1.639 1.627 1.616 1.609 1.599 1.589 1.581 1.573 1.552 1.531 1.518 1.513 1.505 1.500 1.486 1.345 1.329 1.317 1.298 1.289 1.285 1.278 1.270 1.267 1.258 1.165 1.156 1.154 0.245
TMS
B
O
O
10f
N3
1.04
1.02
2.80
1.03
1.96
1.04
2.00
1.08
1.08
2.25
4.94
14.10
9.00
10.0 9.5 9.0 8.5 8.0 7.5 7.0 6.5 6.0 5.5 5.0 4.5 4.0 3.5 3.0 2.5 2.0 1.5 1.0 0.5 0.0
f1 (ppm)

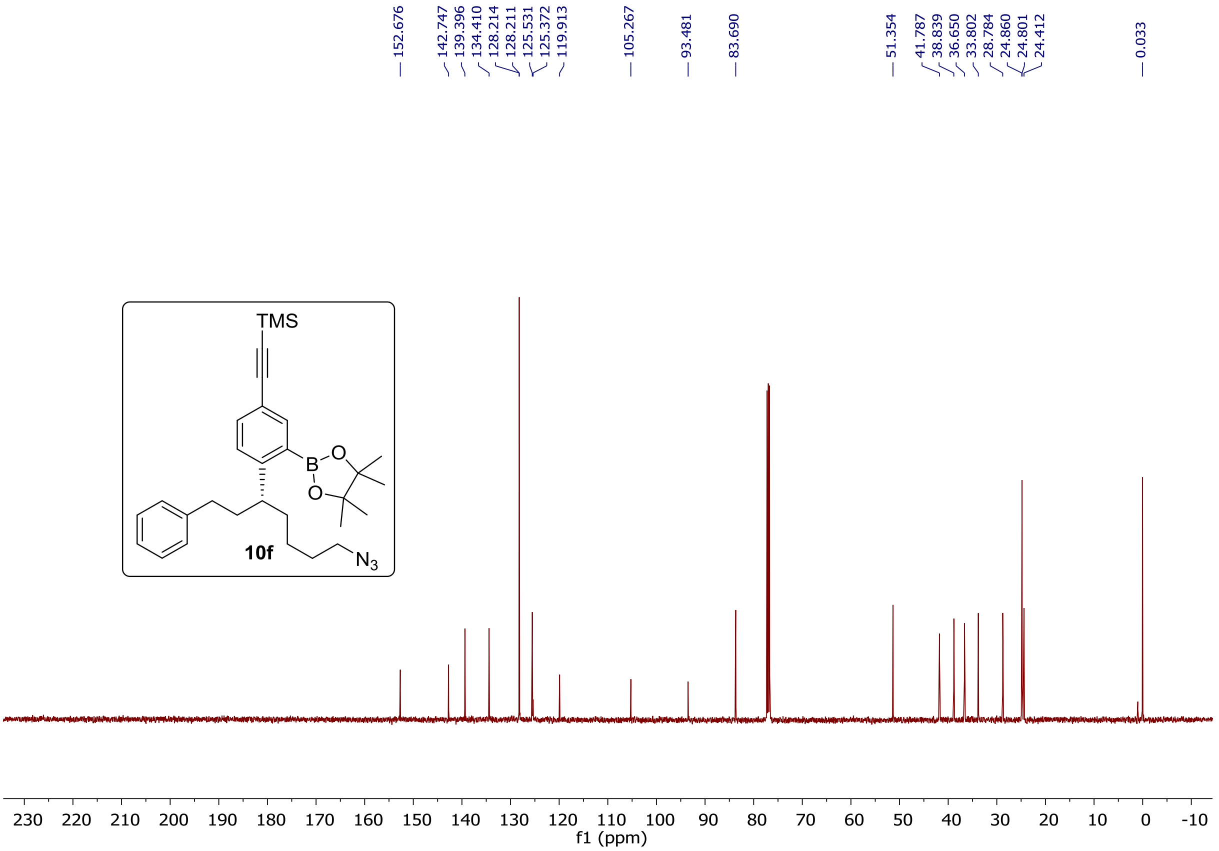
152.676
142.747 139.396 134.410 128.214 128.211 125.531 125.372 119.913
105.267
93.481
83.690
51.354
41.787 38.839 36.650 33.802 28.784 24.860 24.801 24.412
0.033
TMS
B
O
O
10f
N3
230 220 210 200 190 180 170 160 150 140 130 120 110 100 90 80 70 60 50 40 30 20 10 0 -10
f1 (ppm)

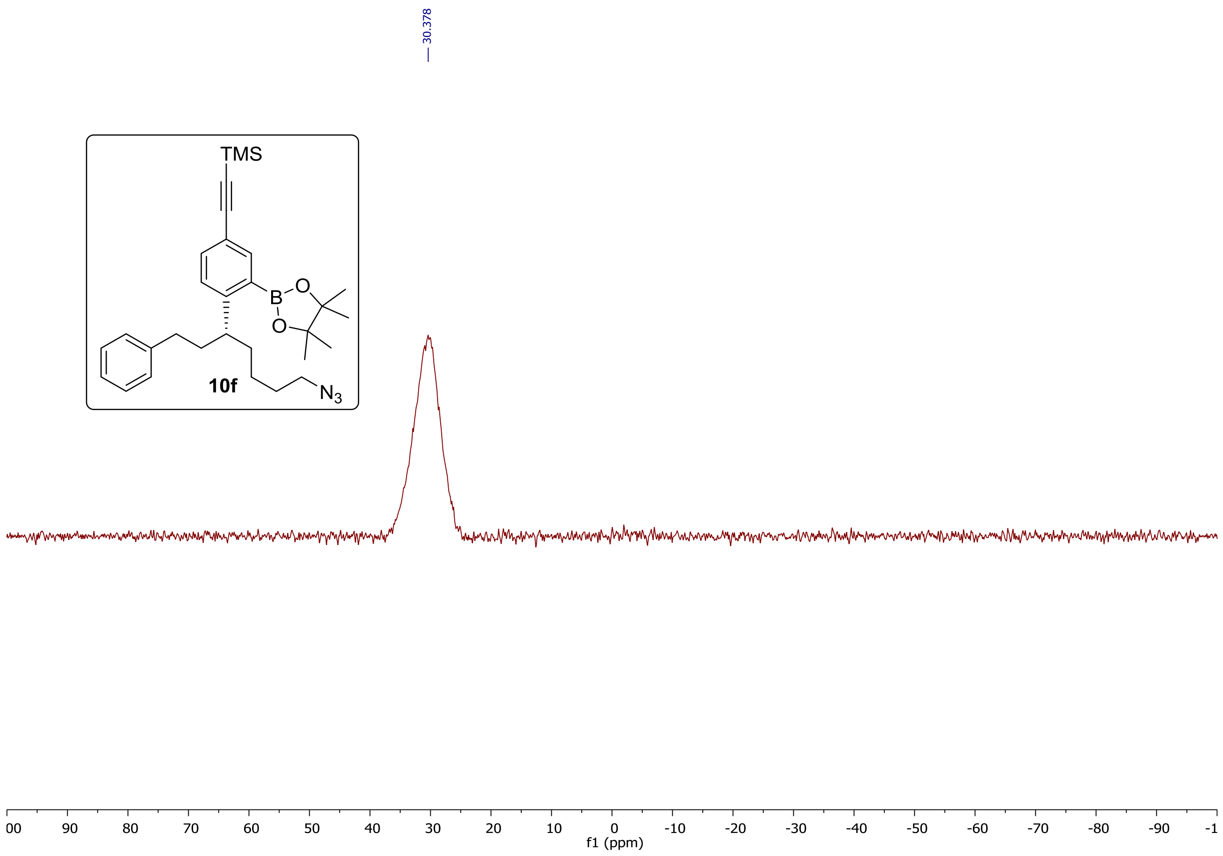
30.378
TMS
B
O
O
10f
N3
f1 (ppm)

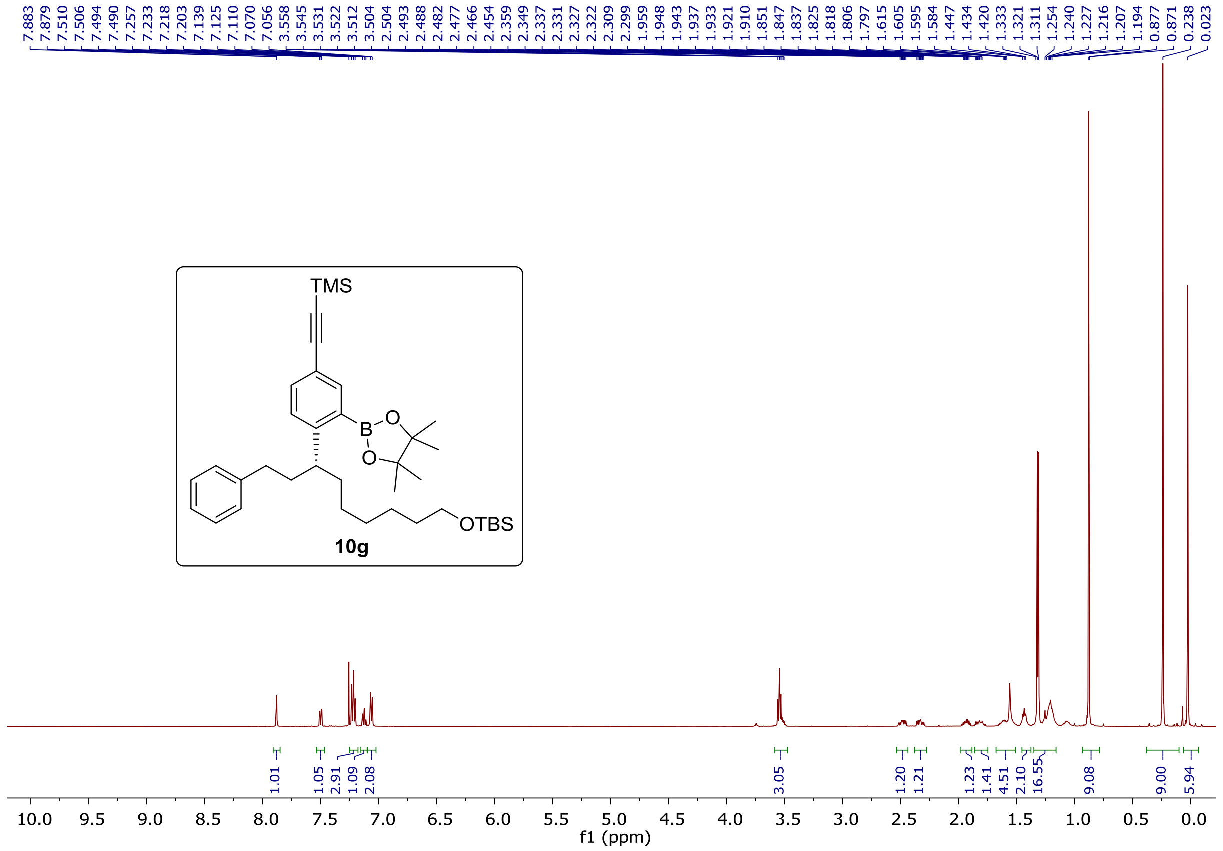
7.883
7.879
7.510
7.506
7.494
7.490
7.257
7.233
7.218
7.203
7.139
7.125
7.110
7.070
7.056
3.558
3.545
3.531
3.522
3.512
3.504
2.504
2.493
2.488
2.482
2.477
2.466
2.454
2.359
2.349
2.337
2.331
2.327
2.322
2.309
2.299
1.959
1.948
1.943
1.937
1.933
1.921
1.910
1.851
1.847
1.837
1.825
1.818
1.806
1.797
1.615
1.605
1.595
1.584
1.447
1.434
1.420
1.333
1.321
1.311
1.254
1.240
1.227
1.216
1.207
1.194
0.877
0.871
0.238
0.023
TMS
B
O
O
OTBS
10g
1.01
1.05
2.91
1.09
2.08
3.05
1.20
1.21
1.23
1.41
4.51
2.10
16.55
9.08
9.00
5.94
f1 (ppm)

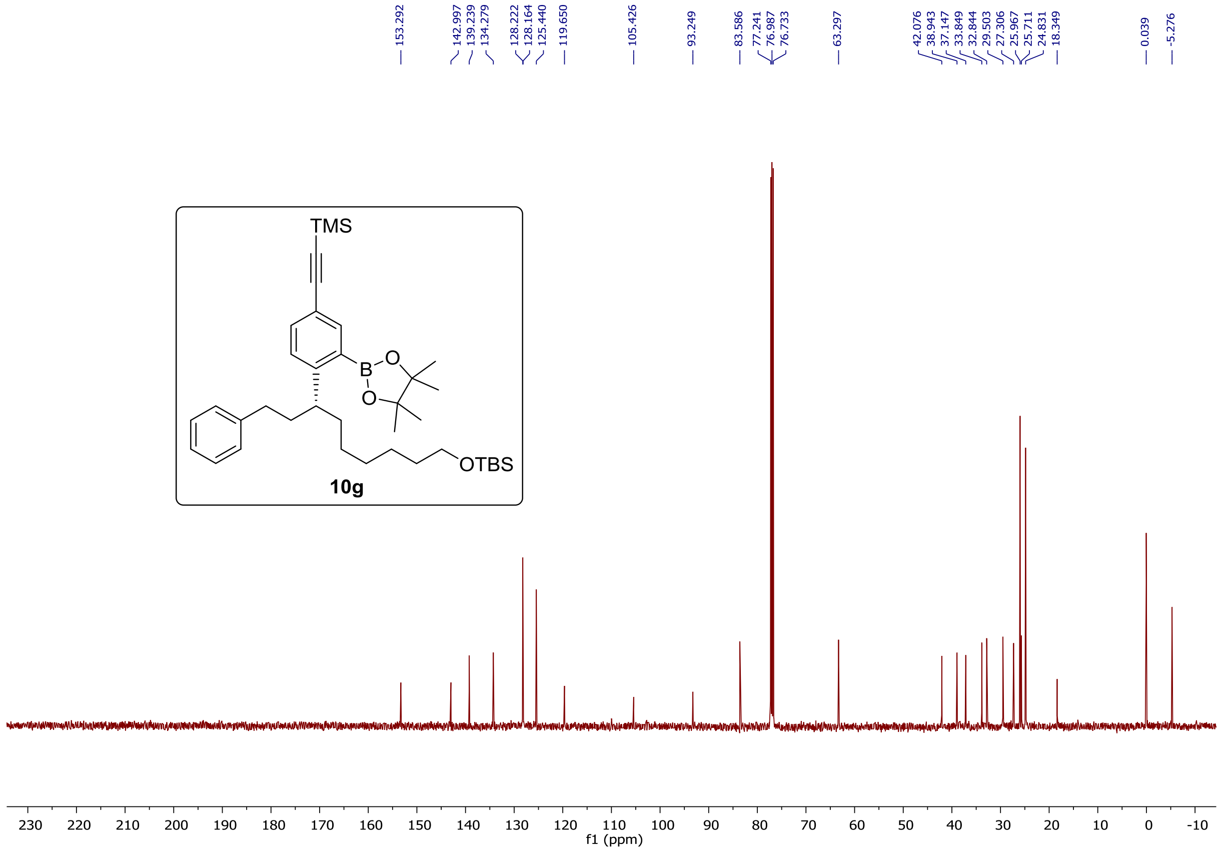
153.292
142.997
139.239
134.279
128.222
128.164
125.440
119.650
105.426
93.249
83.586
77.241
76.987
76.733
63.297
42.076
38.943
37.147
33.849
32.844
29.503
27.306
25.967
25.711
24.831
18.349
0.039
-5.276
TMS
B
O
O
OTBS
10g
f1 (ppm)

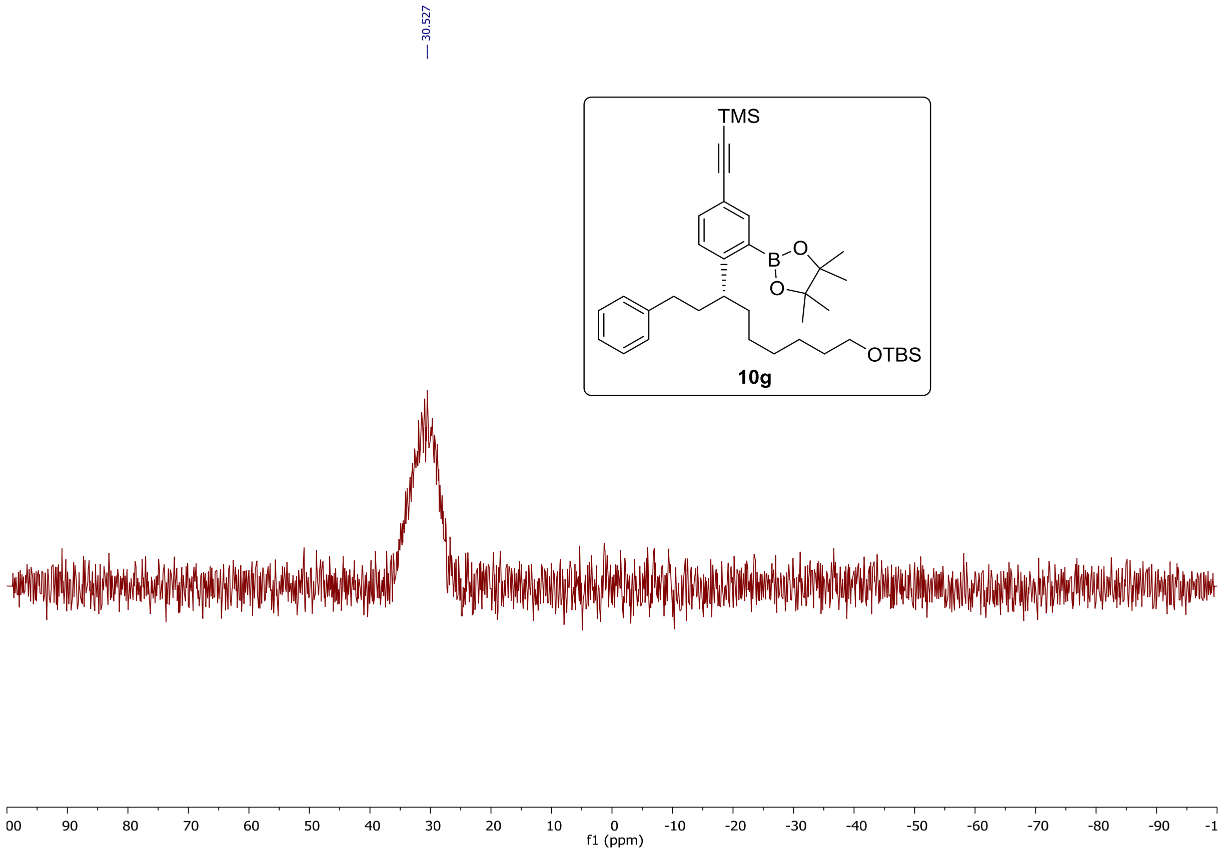
30.527
TMS
B
O
O
OTBS
10g
f1 (ppm)

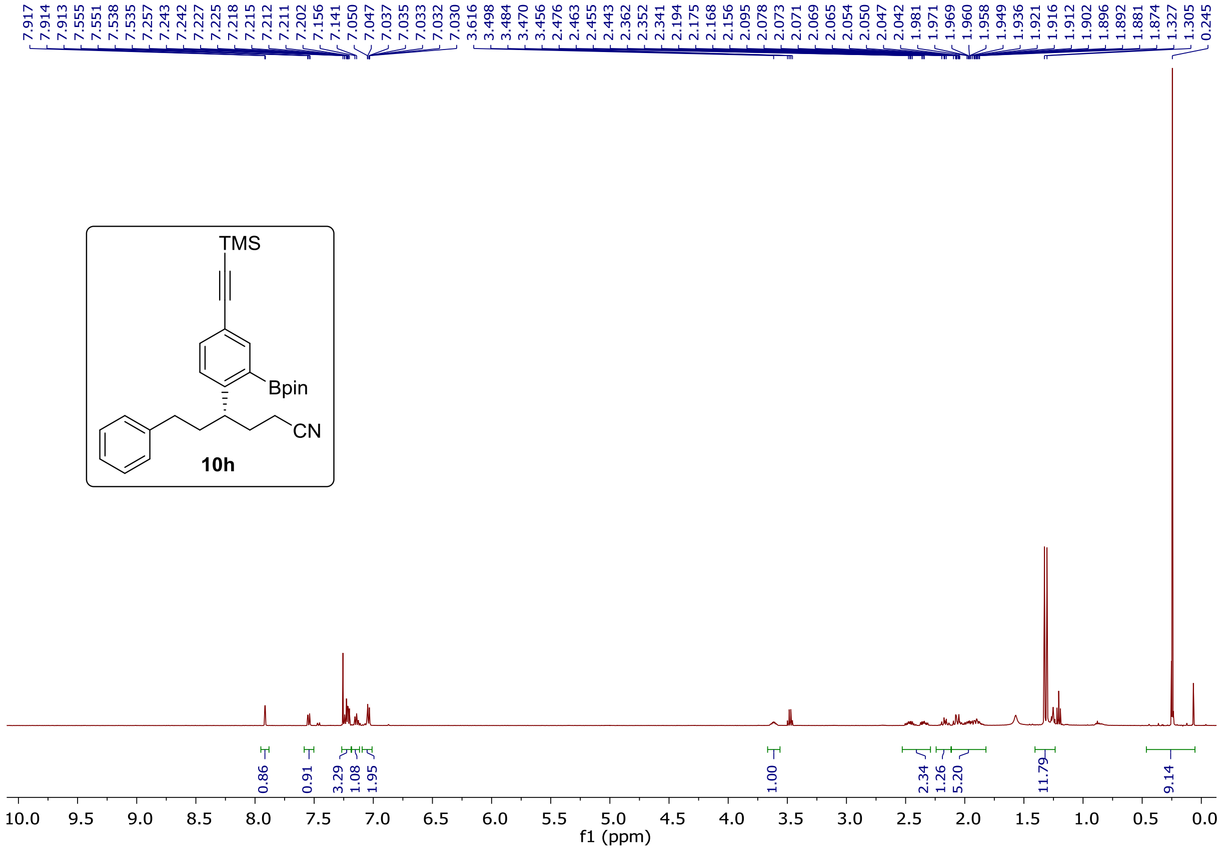
7.917
7.914
7.913
7.555
7.551
7.538
7.535
7.257
7.243
7.242
7.227
7.225
7.218
7.215
7.212
7.211
7.202
7.156
7.141
7.050
7.047
7.037
7.035
7.033
7.032
7.030
3.616
3.498
3.484
3.470
3.456
2.476
2.463
2.455
2.443
2.362
2.352
2.341
2.194
2.175
2.168
2.156
2.095
2.078
2.073
2.071
2.069
2.065
2.054
2.050
2.047
2.042
1.981
1.971
1.969
1.960
1.958
1.949
1.936
1.921
1.916
1.912
1.902
1.896
1.892
1.881
1.874
1.327
1.305
0.245
TMS
Bpin
CN
10h
0.86
0.91
3.29
1.08
1.95
1.00
2.34
1.26
5.20
11.79
9.14
10.0 9.5 9.0 8.5 8.0 7.5 7.0 6.5 6.0 5.5 5.0 4.5 4.0 3.5 3.0 2.5 2.0 1.5 1.0 0.5 0.0
f1 (ppm)

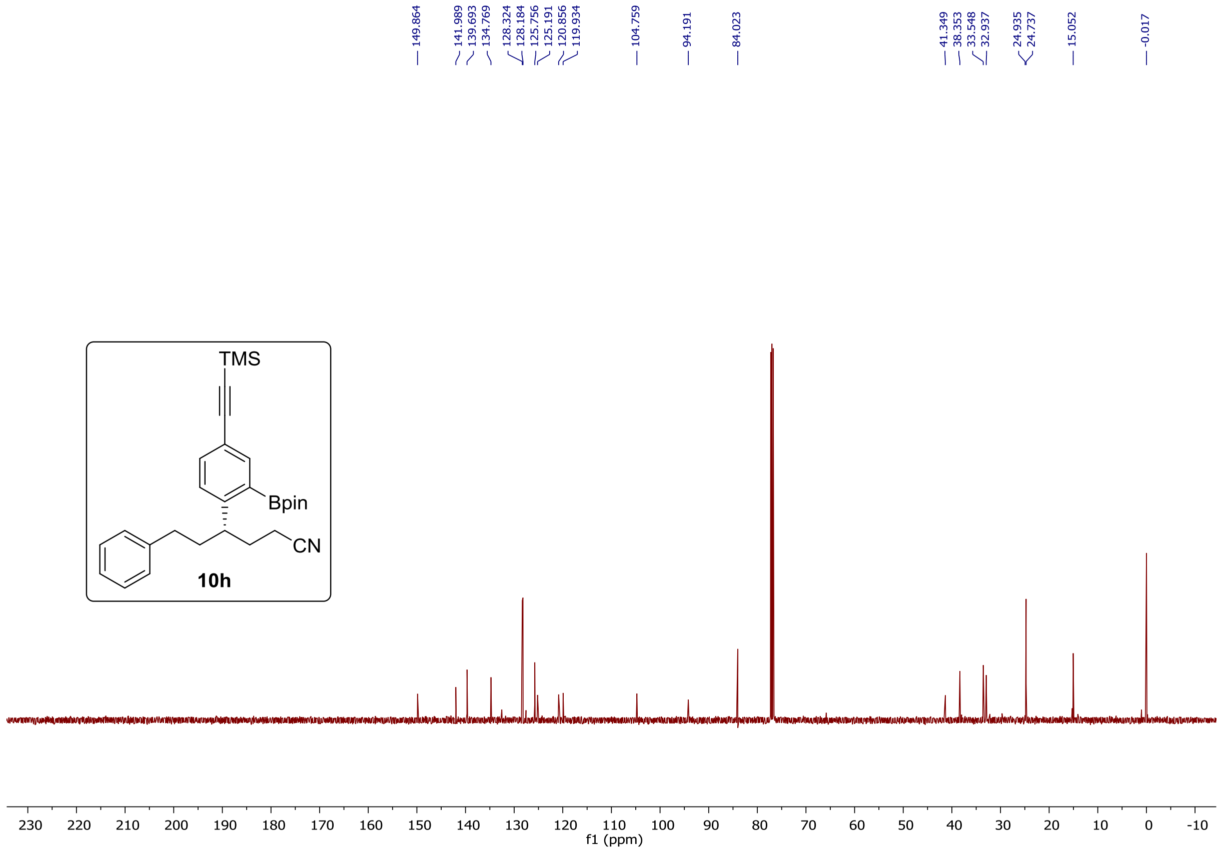
149.864
141.989
139.693
134.769
128.324
128.184
125.756
125.191
120.856
119.934
104.759
94.191
84.023
41.349
38.353
33.548
32.937
24.935
24.737
15.052
-0.017
TMS
Bpin
CN
10h
230 220 210 200 190 180 170 160 150 140 130 120 110 100 90 80 70 60 50 40 30 20 10 0 -10
f1 (ppm)

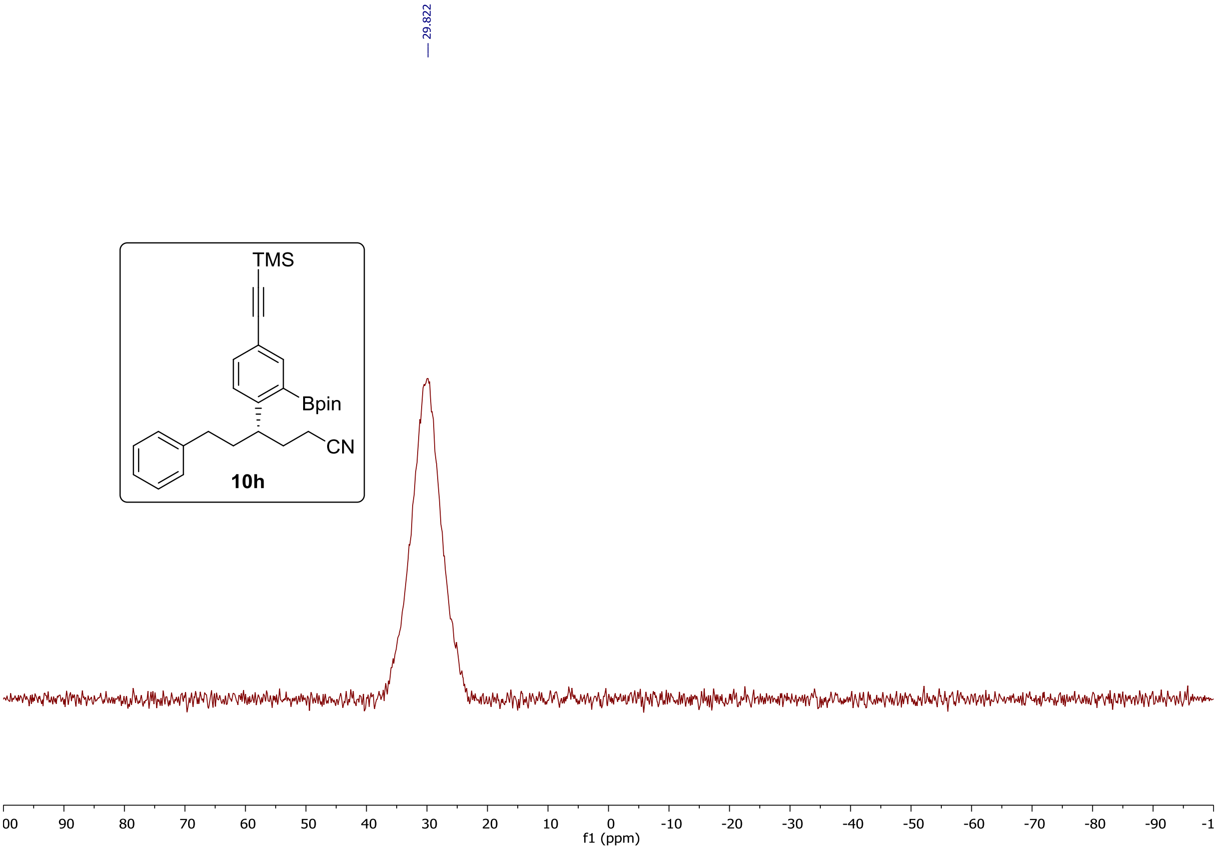
29.822
TMS
Bpin
CN
10h
00 90 80 70 60 50 40 30 20 10 0 -10 -20 -30 -40 -50 -60 -70 -80 -90 -1
f1 (ppm)

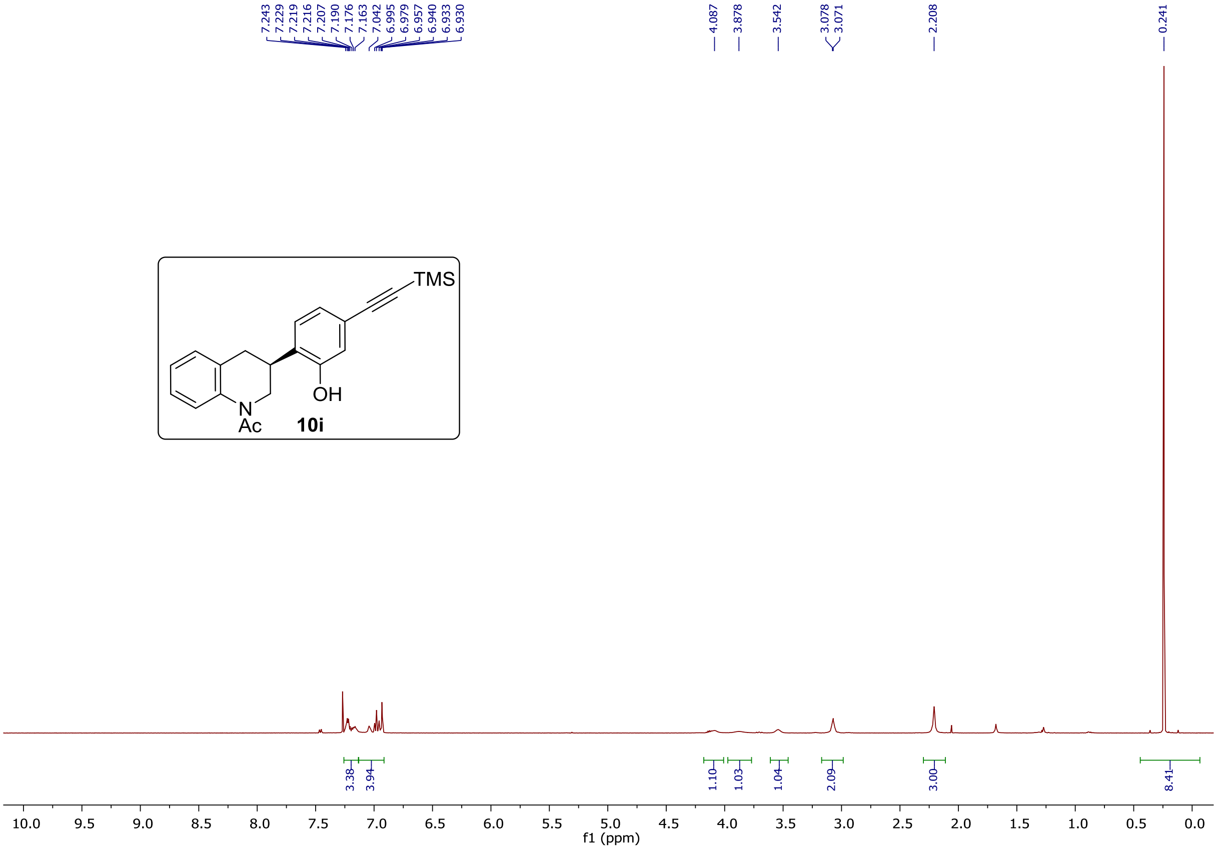
7.243 7.229 7.219 7.216 7.207 7.190 7.176 7.163 7.042 6.995 6.979 6.957 6.940 6.933 6.930
4.087
3.878
3.542
3.078 3.071
2.208
0.241
TMS
OH
N
Ac
10i
3.38 3.94 1.10 1.03 1.04 2.09 3.00 8.41
10.0 9.5 9.0 8.5 8.0 7.5 7.0 6.5 6.0 5.5 5.0 4.5 4.0 3.5 3.0 2.5 2.0 1.5 1.0 0.5 0.0
f1 (ppm)

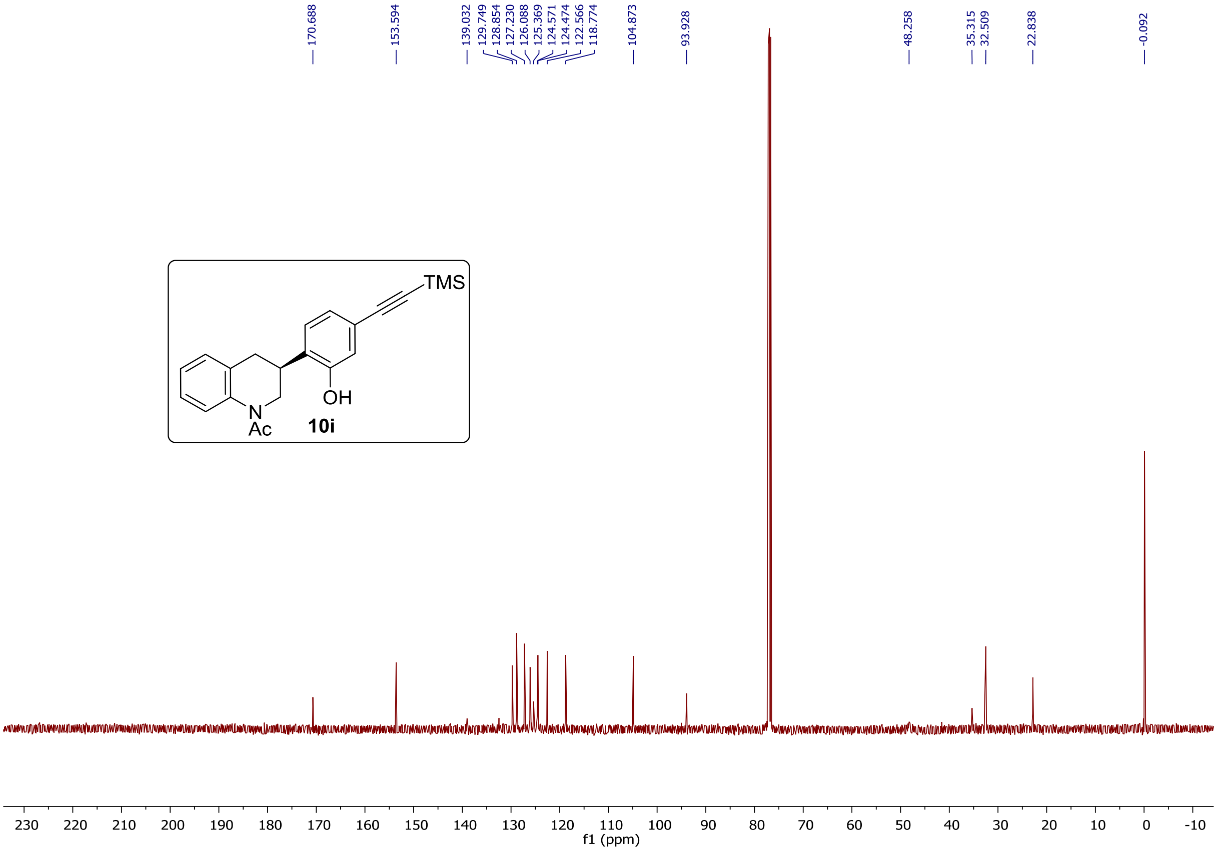
170.688
153.594
139.032
129.749
128.854
127.230
126.088
125.369
124.571
124.474
122.566
118.774
104.873
93.928
48.258
35.315
32.509
22.838
-0.092
TMS
OH
N
Ac
10i
230 220 210 200 190 180 170 160 150 140 130 120 110 100 90 80 70 60 50 40 30 20 10 0 -10
f1 (ppm)

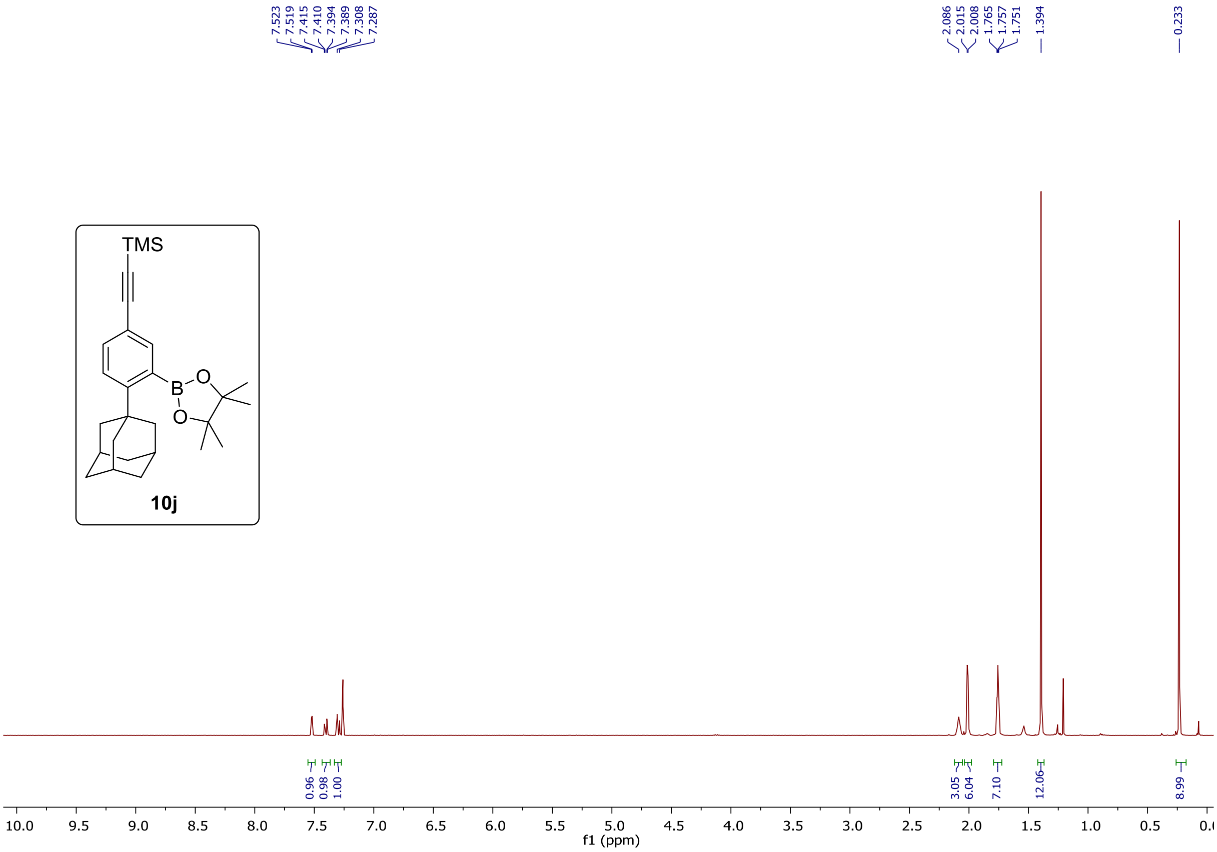
7.523
7.519
7.415
7.410
7.394
7.389
7.308
7.287
2.086
2.015
2.008
1.765
1.757
1.751
1.394
0.233
TMS
B
O
O
10j
0.96
0.98
1.00
3.05
6.04
7.10
12.06
8.99
10.0 9.5 9.0 8.5 8.0 7.5 7.0 6.5 6.0 5.5 5.0 4.5 4.0 3.5 3.0 2.5 2.0 1.5 1.0 0.5
f1 (ppm)

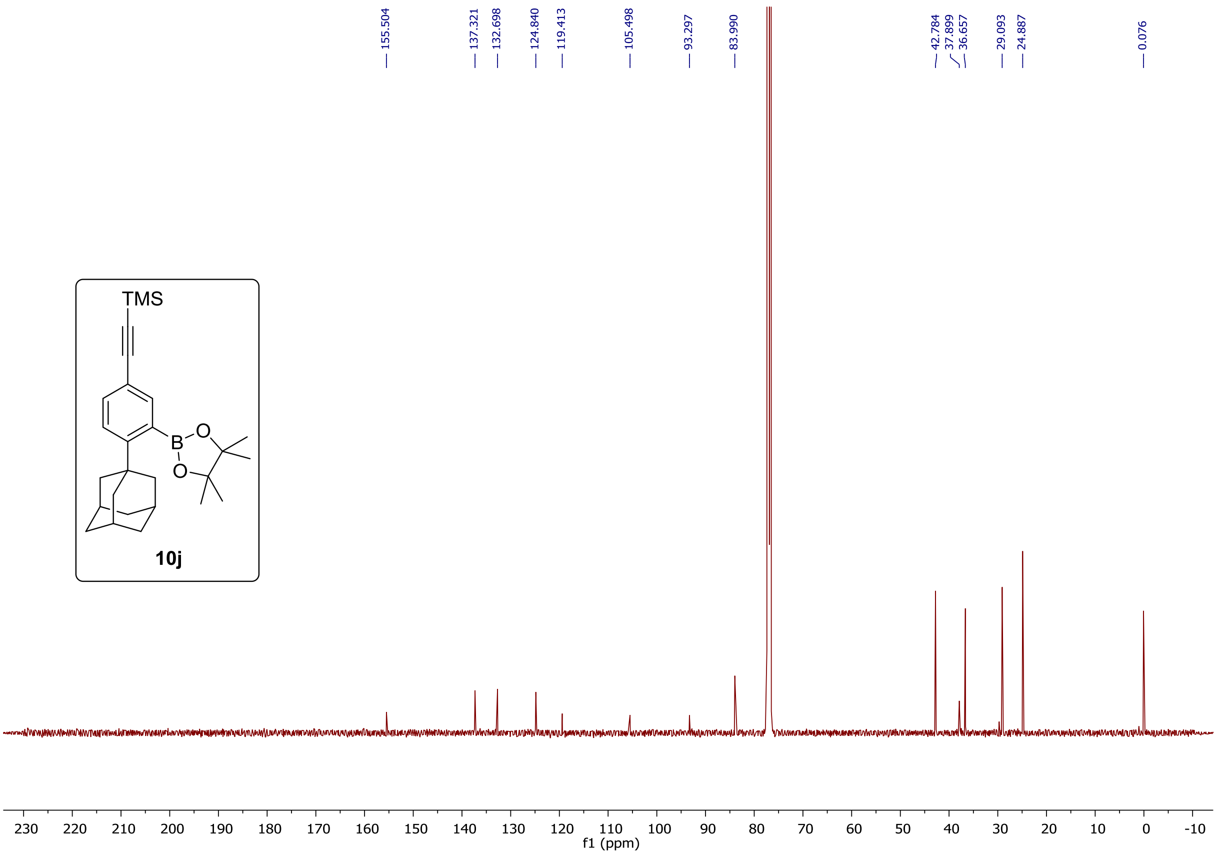
155.504
137.321
132.698
124.840
119.413
105.498
93.297
83.990
42.784
37.899
36.657
29.093
24.887
0.076
TMS
B
O
O
10j
230 220 210 200 190 180 170 160 150 140 130 120 110 100 90 80 70 60 50 40 30 20 10 0 -10
f1 (ppm)

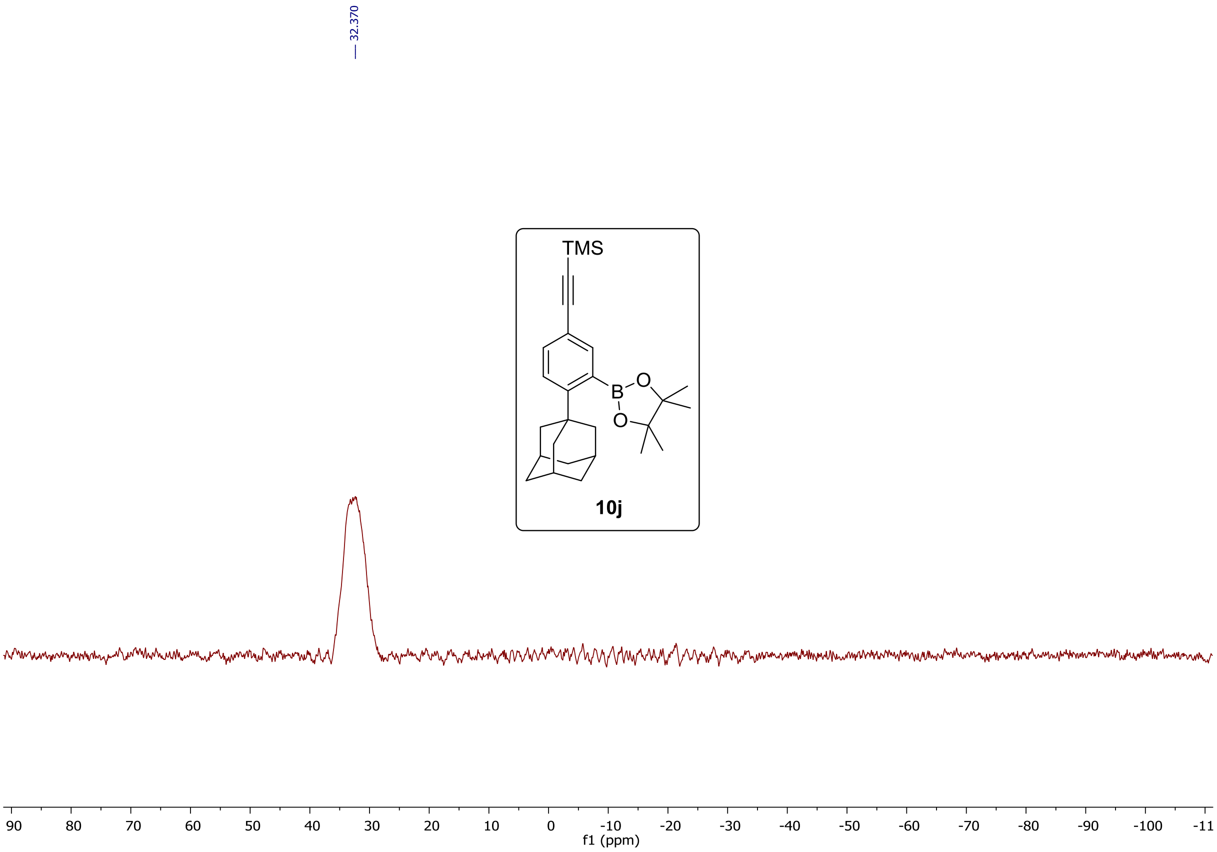
32.370
TMS
B
O
O
10j
90 80 70 60 50 40 30 20 10 0 -10 -20 -30 -40 -50 -60 -70 -80 -90 -100 -11
f1 (ppm)

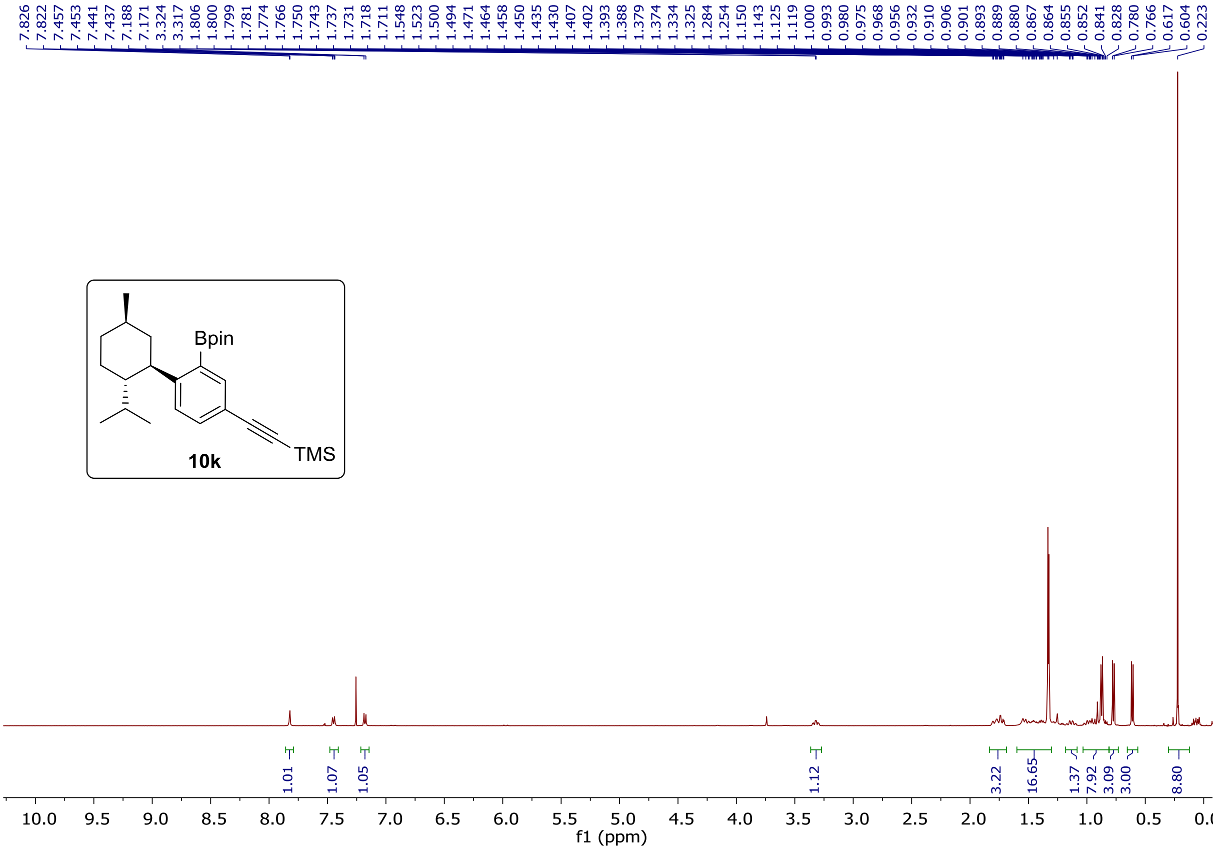
7.826
7.822
7.457
7.453
7.441
7.437
7.188
7.171
3.324
3.317
1.806
1.800
1.799
1.781
1.774
1.766
1.750
1.743
1.737
1.731
1.718
1.711
1.548
1.523
1.500
1.494
1.471
1.464
1.458
1.450
1.435
1.430
1.407
1.402
1.393
1.388
1.379
1.374
1.334
1.325
1.284
1.254
1.150
1.143
1.125
1.119
1.000
0.993
0.980
0.975
0.968
0.956
0.932
0.910
0.906
0.901
0.893
0.889
0.880
0.867
0.864
0.855
0.852
0.841
0.828
0.780
0.766
0.617
0.604
0.223
Bpin
TMS
10k
1.01
1.07
1.05
1.12
3.22
16.65
1.37
7.92
3.09
3.00
8.80
10.0
9.5
9.0
8.5
8.0
7.5
7.0
6.5
6.0
5.5
5.0
4.5
4.0
3.5
3.0
2.5
2.0
1.5
1.0
0.5
f1 (ppm)

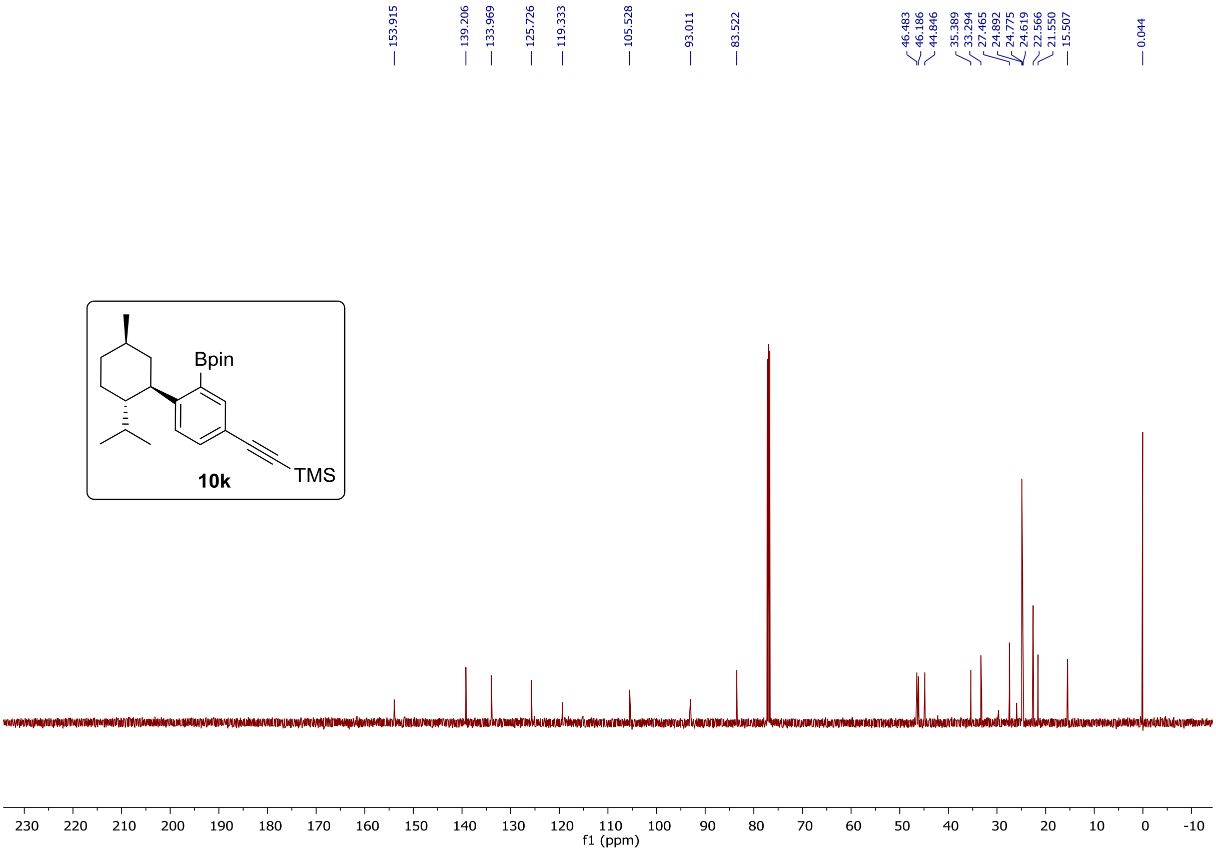
153.915
139.206
133.969
125.726
119.333
105.528
93.011
83.522
46.483
46.186
44.846
35.389
33.294
27.465
24.892
24.775
24.619
22.566
21.550
15.507
0.044
Bpin
TMS
10k
230
220
210
200
190
180
170
160
150
140
130
120
110
100
90
80
70
60
50
40
30
20
10
0
-10
f1 (ppm)

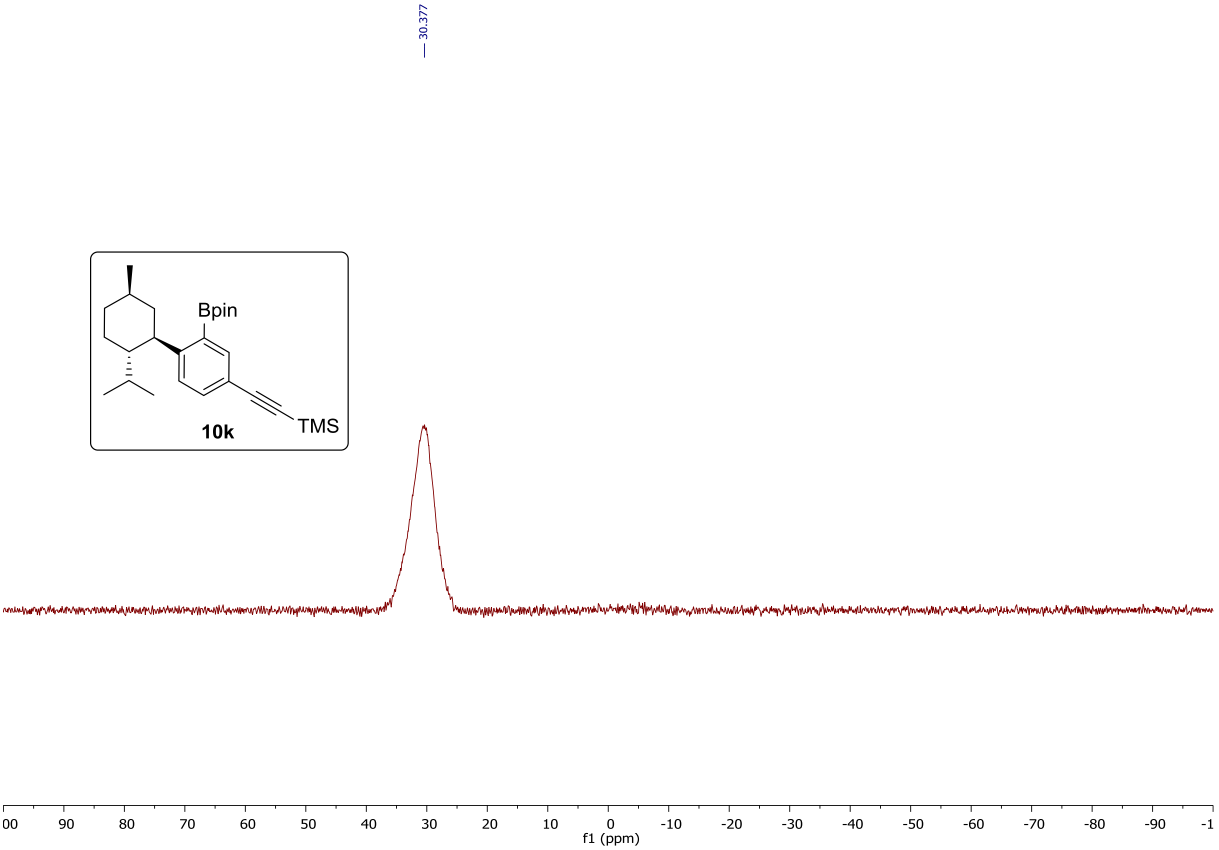
30.377
Bpin
TMS
10k
f1 (ppm)

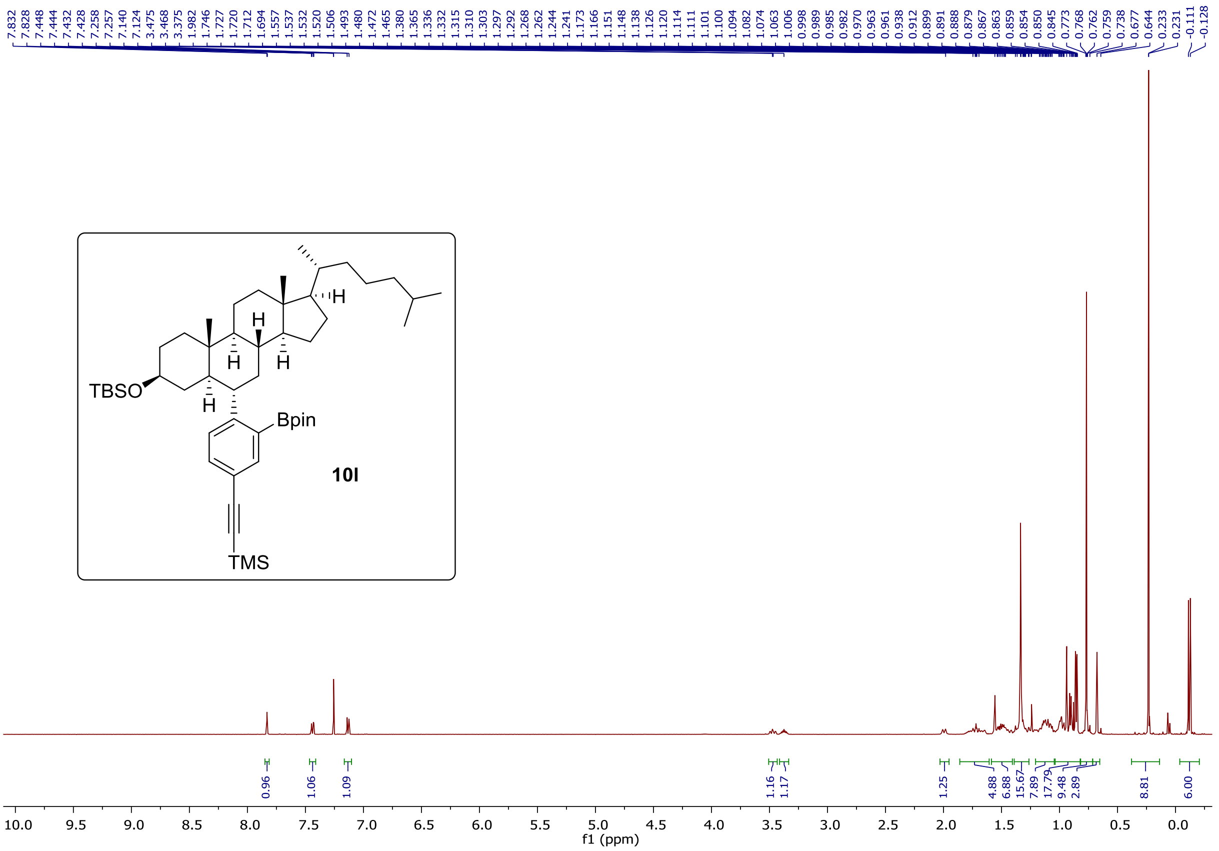
7.832 7.828 7.448 7.444 7.432 7.428 7.258 7.257 7.140 7.124 3.475 3.468 3.375 1.982 1.746 1.727 1.720 1.712 1.694 1.557 1.537 1.532 1.520 1.506 1.493 1.480 1.472 1.465 1.380 1.365 1.336 1.332 1.315 1.310 1.303 1.297 1.292 1.268 1.262 1.244 1.241 1.173 1.166 1.151 1.148 1.138 1.126 1.120 1.114 1.111 1.101 1.100 1.094 1.082 1.074 1.063 1.006 0.998 0.989 0.985 0.982 0.970 0.963 0.961 0.938 0.912 0.899 0.891 0.888 0.879 0.867 0.863 0.859 0.854 0.850 0.845 0.773 0.768 0.762 0.759 0.738 0.677 0.644 0.233 0.231 -0.111 -0.128
TBSO
Bpin
TMS
10l
0.96
1.06
1.09
1.16
1.17
1.25
4.88
6.88
15.67
7.89
17.79
9.48
2.89
8.81
6.00
f1 (ppm)

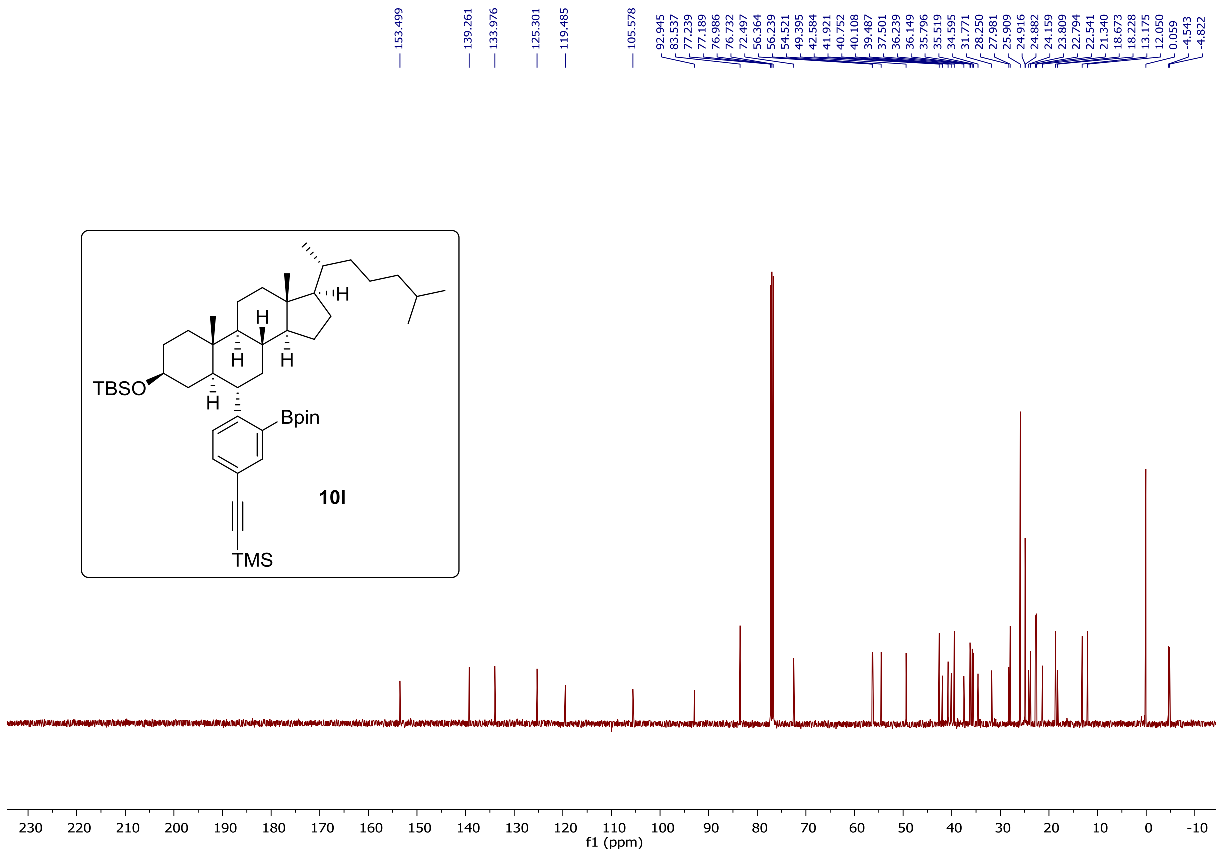
153.499
139.261
133.976
125.301
119.485
105.578
92.945
83.537
77.239
77.189
76.986
76.732
72.497
56.364
56.239
54.521
49.395
42.584
41.921
40.752
40.108
39.487
37.501
36.239
36.149
35.796
35.519
34.595
31.771
28.250
27.981
25.909
24.916
24.882
24.159
23.809
22.794
22.541
21.340
18.673
18.228
13.175
12.050
0.059
-4.543
-4.822
TBSO
Bpin
10l
TMS
230 220 210 200 190 180 170 160 150 140 130 120 110 100 90 80 70 60 50 40 30 20 10 0 -10
f1 (ppm)

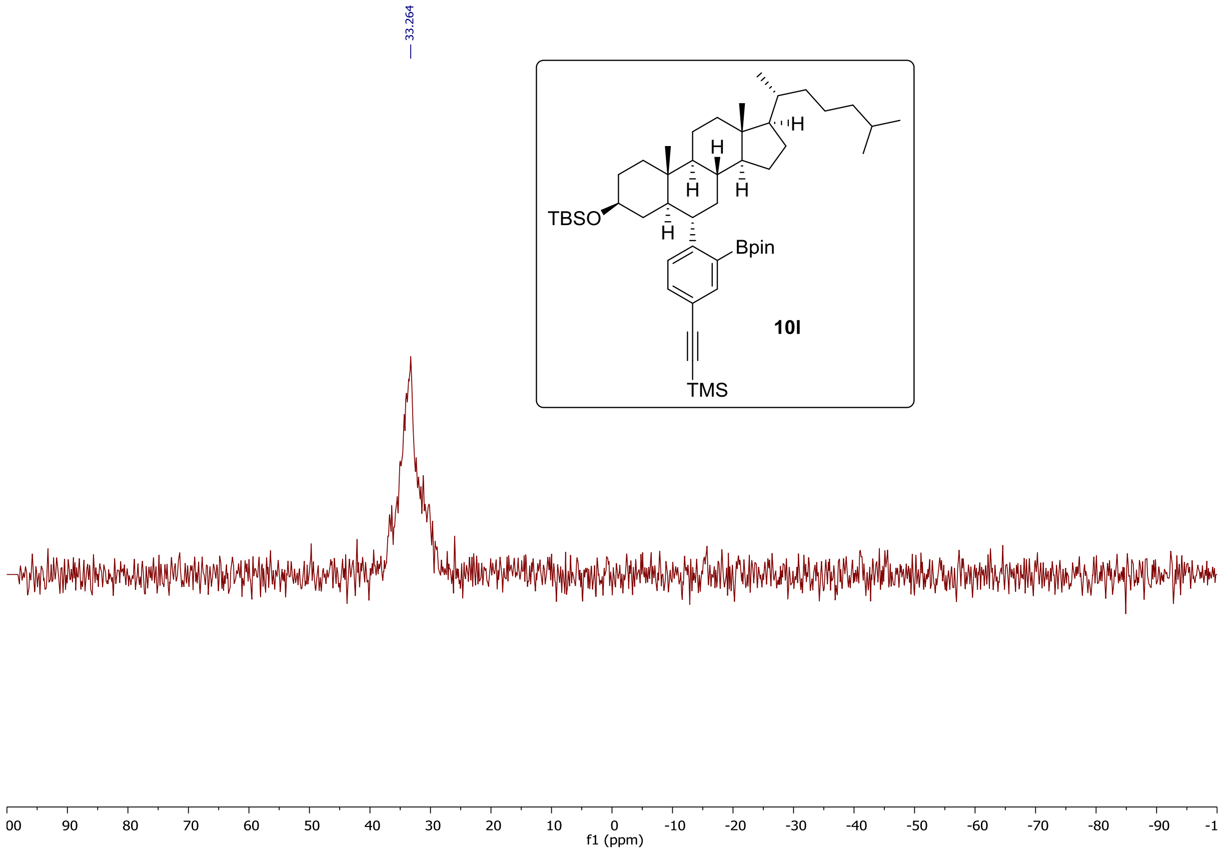
33.264
TBSO
Bpin
10l
TMS
00 90 80 70 60 50 40 30 20 10 0 -10 -20 -30 -40 -50 -60 -70 -80 -90 -1
f1 (ppm)

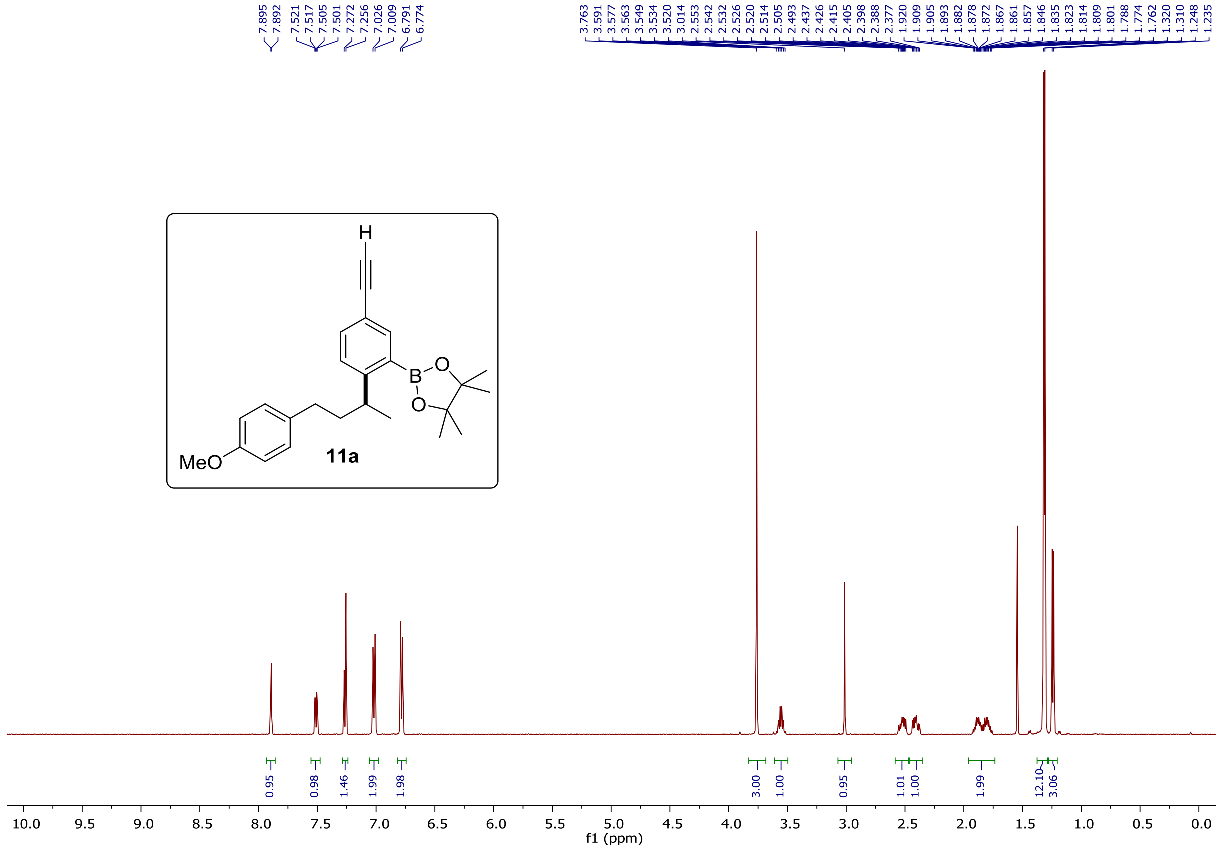
7.895
7.892
7.521
7.517
7.505
7.501
7.272
7.256
7.026
7.009
6.791
6.774
3.763
3.591
3.577
3.563
3.549
3.534
3.520
3.014
2.553
2.542
2.532
2.526
2.520
2.514
2.505
2.493
2.437
2.426
2.415
2.405
2.398
2.388
2.377
1.920
1.909
1.905
1.893
1.882
1.878
1.872
1.867
1.861
1.857
1.846
1.835
1.823
1.814
1.809
1.801
1.788
1.774
1.762
1.320
1.310
1.248
1.235
H
B
O
O
MeO
11a
0.95
0.98
1.46
1.99
1.98
3.00
1.00
0.95
1.01
1.00
1.99
12.10
3.06
f1 (ppm)

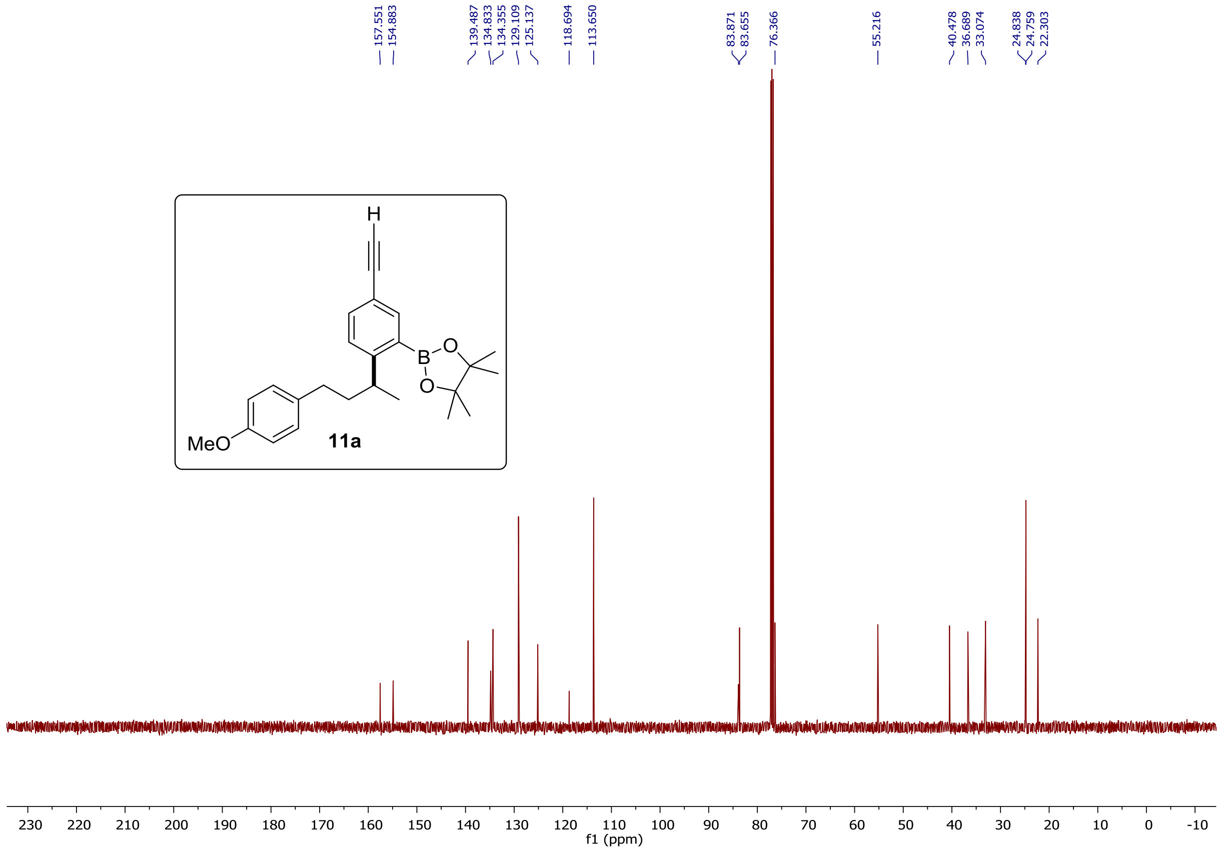
157.551
154.883
139.487
134.833
134.355
129.109
125.137
118.694
113.650
83.871
83.655
76.366
55.216
40.478
36.689
33.074
24.838
24.759
22.303
H
B
O
O
MeO
11a
f1 (ppm)

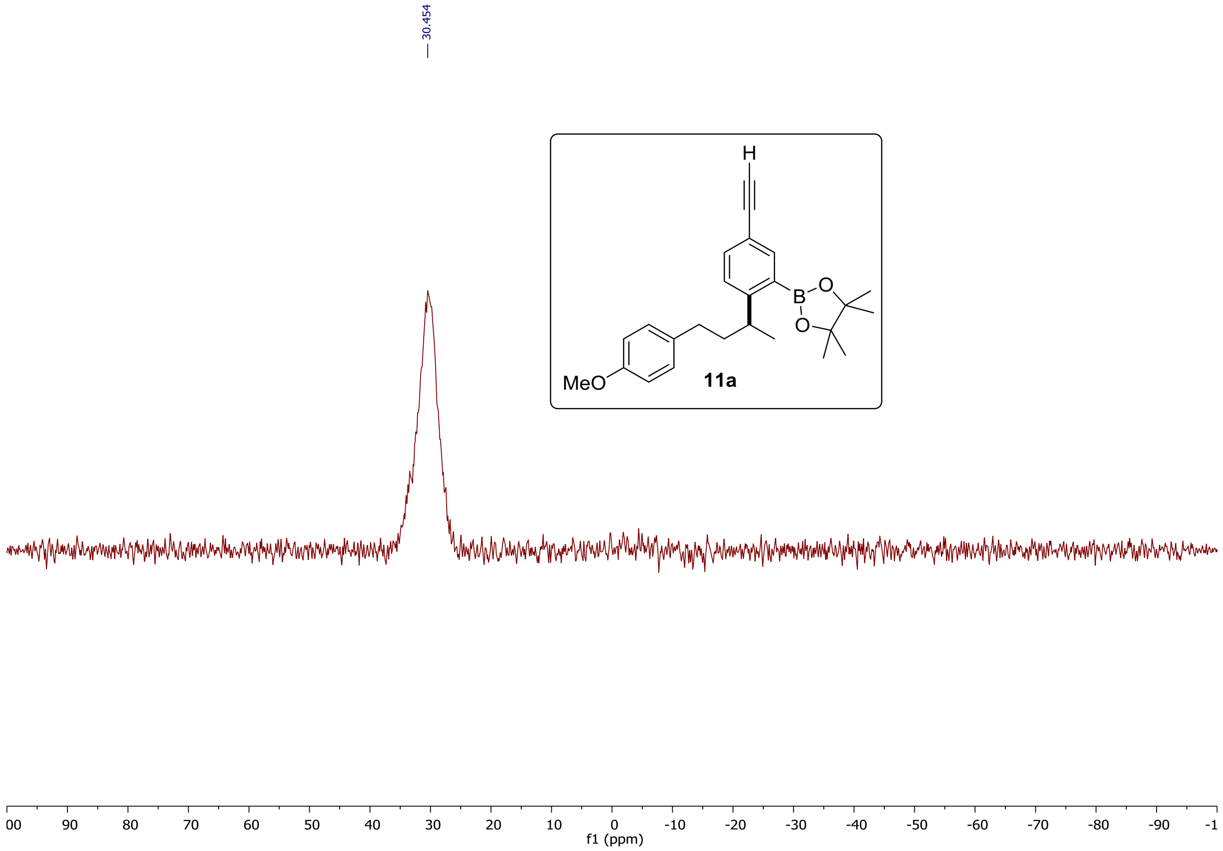

30.454
H
B
O
O
MeO
11a
f1 (ppm)

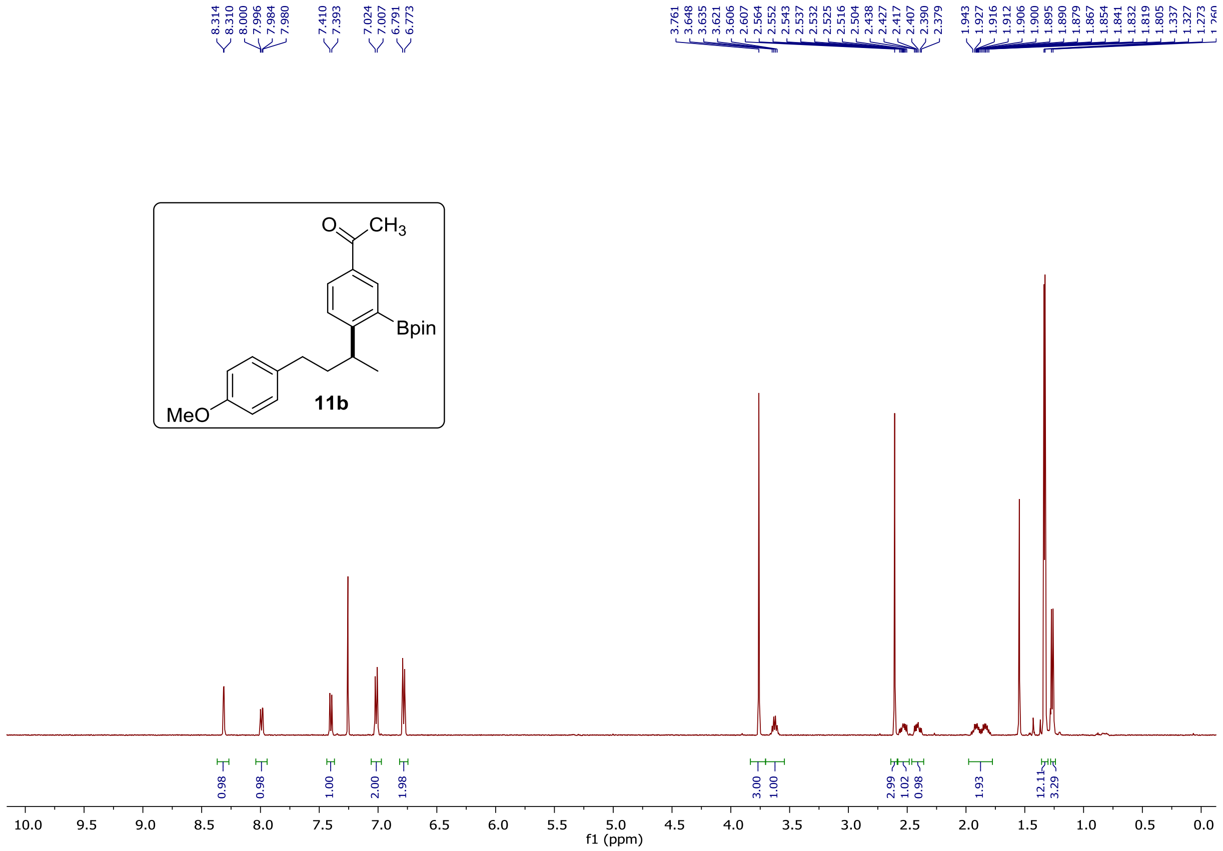

8.314
8.310
8.000
7.996
7.984
7.980
7.410
7.393
7.024
7.007
6.791
6.773
3.761
3.648
3.635
3.621
3.606
2.607
2.564
2.552
2.543
2.537
2.532
2.525
2.516
2.504
2.438
2.427
2.417
2.407
2.390
2.379
1.943
1.927
1.916
1.912
1.906
1.900
1.895
1.890
1.879
1.867
1.854
1.841
1.832
1.819
1.805
1.337
1.327
1.273
O
CH3
Bpin
MeO
11b
0.98
0.98
1.00
2.00
1.98
3.00
1.00
2.99
1.02
0.98
1.93
12.11
3.29
f1 (ppm)

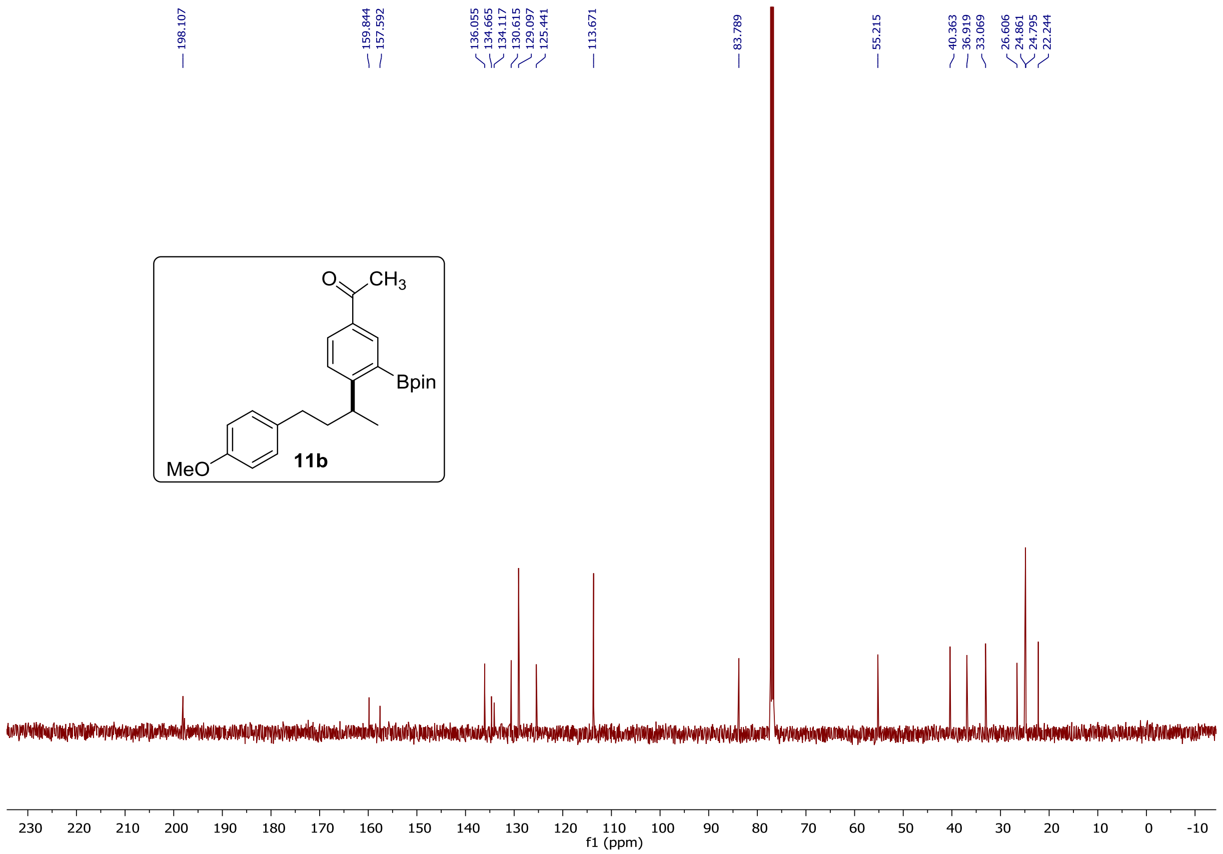
198.107
159.844
157.592
136.055
134.665
134.117
130.615
129.097
125.441
113.671
83.789
55.215
40.363
36.919
33.069
26.606
24.861
24.795
22.244
CH3
O
Bpin
MeO
11b
230 220 210 200 190 180 170 160 150 140 130 120 110 100 90 80 70 60 50 40 30 20 10 0 -10
f1 (ppm)

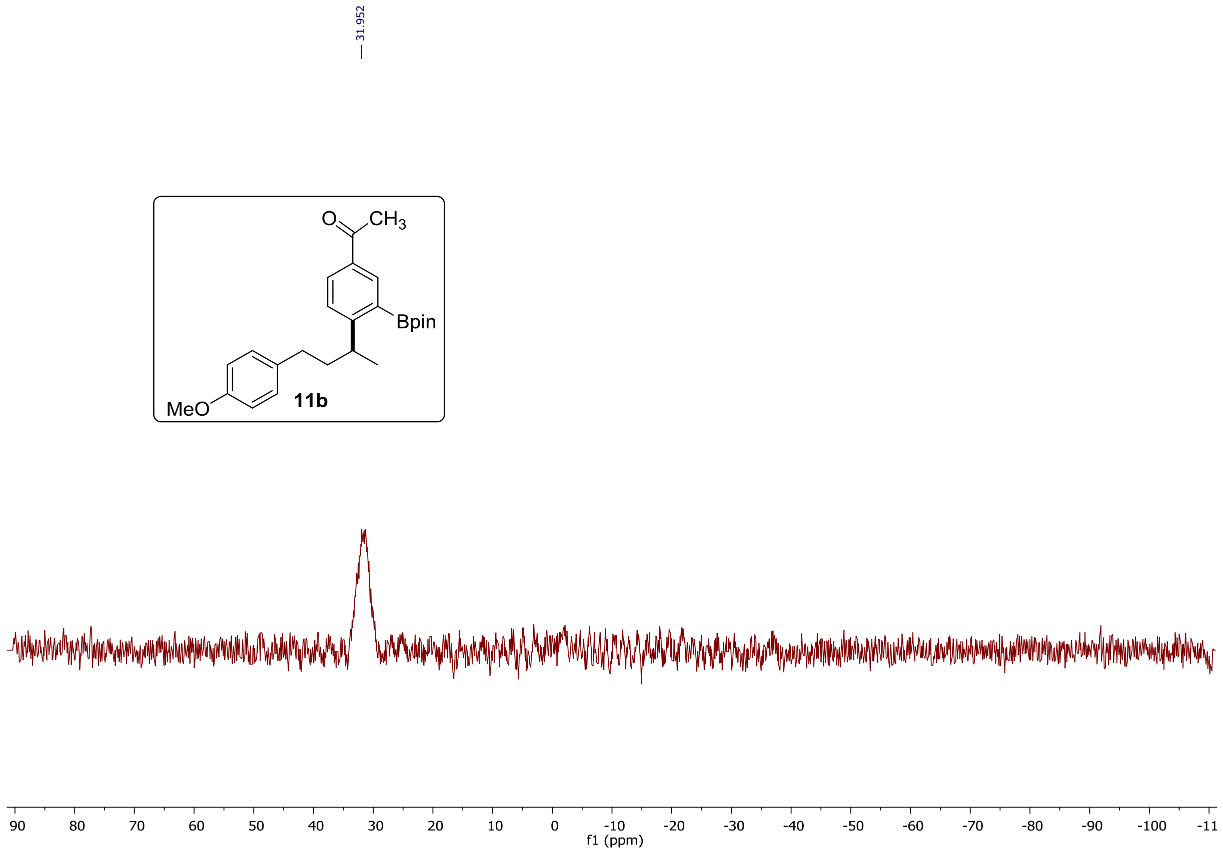
31.952
CH3
O
Bpin
MeO
11b
90 80 70 60 50 40 30 20 10 0 -10 -20 -30 -40 -50 -60 -70 -80 -90 -100 -11
f1 (ppm)

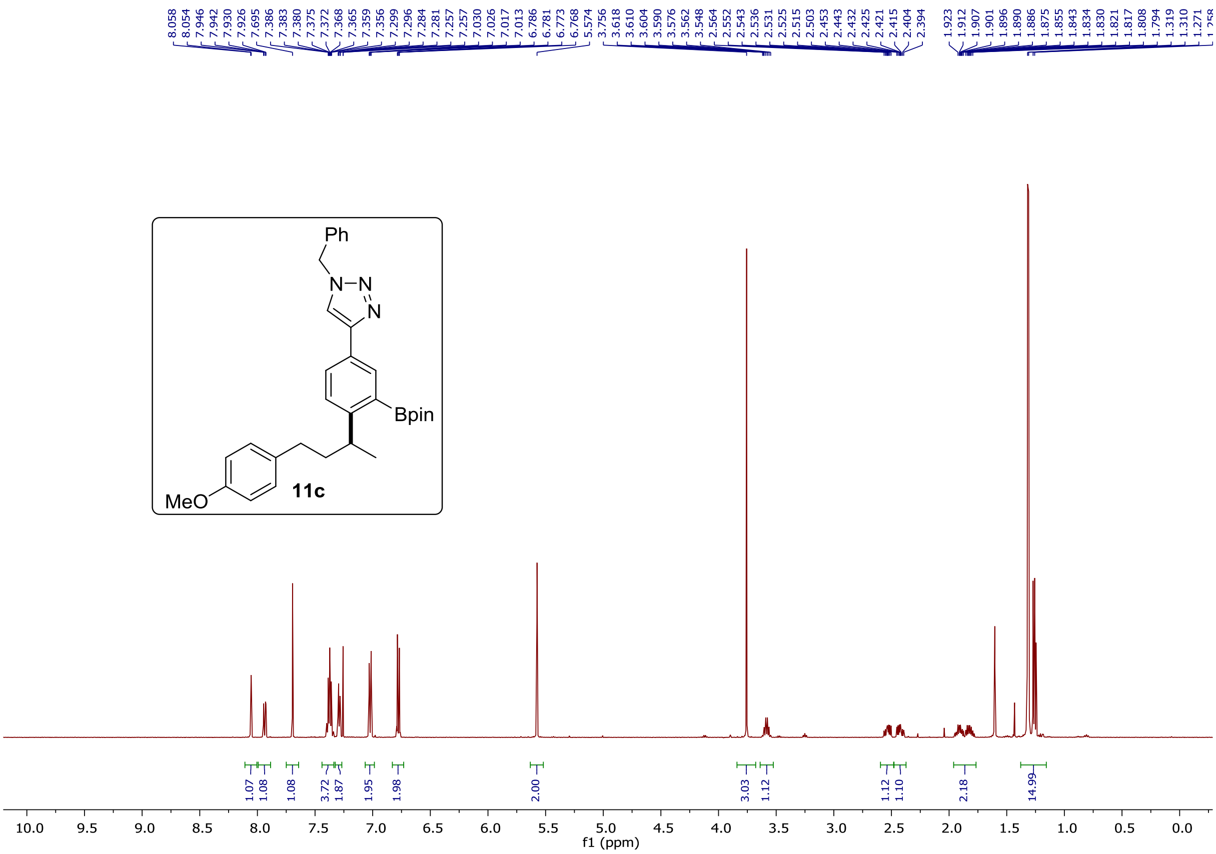
8.058 8.054 7.946 7.942 7.930 7.926 7.695 7.386 7.383 7.380 7.375 7.372 7.368 7.365 7.359 7.356 7.299 7.296 7.284 7.281 7.257 7.257 7.030 7.026 7.017 7.013 6.786 6.781 6.773 6.768 5.574 3.756 3.618 3.610 3.604 3.590 3.576 3.562 3.548 2.564 2.552 2.543 2.536 2.531 2.525 2.515 2.503 2.453 2.443 2.432 2.425 2.421 2.415 2.404 2.394
1.923 1.912 1.907 1.901 1.896 1.890 1.886 1.875 1.855 1.843 1.834 1.830 1.821 1.817 1.808 1.794 1.319 1.310 1.271
Ph
N
N
N
Bpin
MeO
11c
1.07 1.08 1.08 3.72 1.87 1.95 1.98 2.00 3.03 1.12 1.12 1.10 2.18 14.99
10.0 9.5 9.0 8.5 8.0 7.5 7.0 6.5 6.0 5.5 5.0 4.5 4.0 3.5 3.0 2.5 2.0 1.5 1.0 0.5 0.0
f1 (ppm)

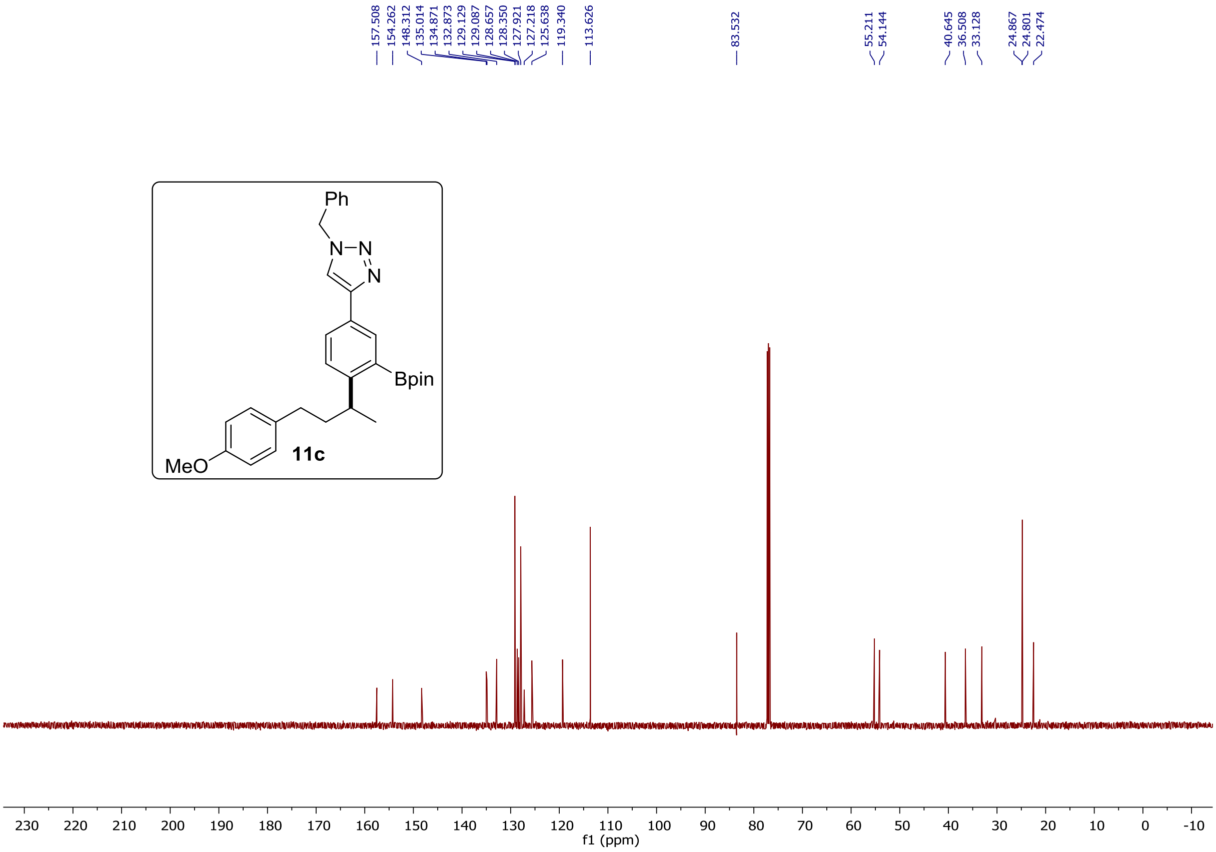
157.508 154.262 148.312 135.014 134.871 132.873 129.129 129.087 128.657 128.350 127.921 127.218 125.638 119.340 113.626
83.532
55.211 54.144
40.645 36.508 33.128
24.867 24.801 22.474
Ph
N
N
N
Bpin
MeO
11c
230 220 210 200 190 180 170 160 150 140 130 120 110 100 90 80 70 60 50 40 30 20 10 0 -10
f1 (ppm)

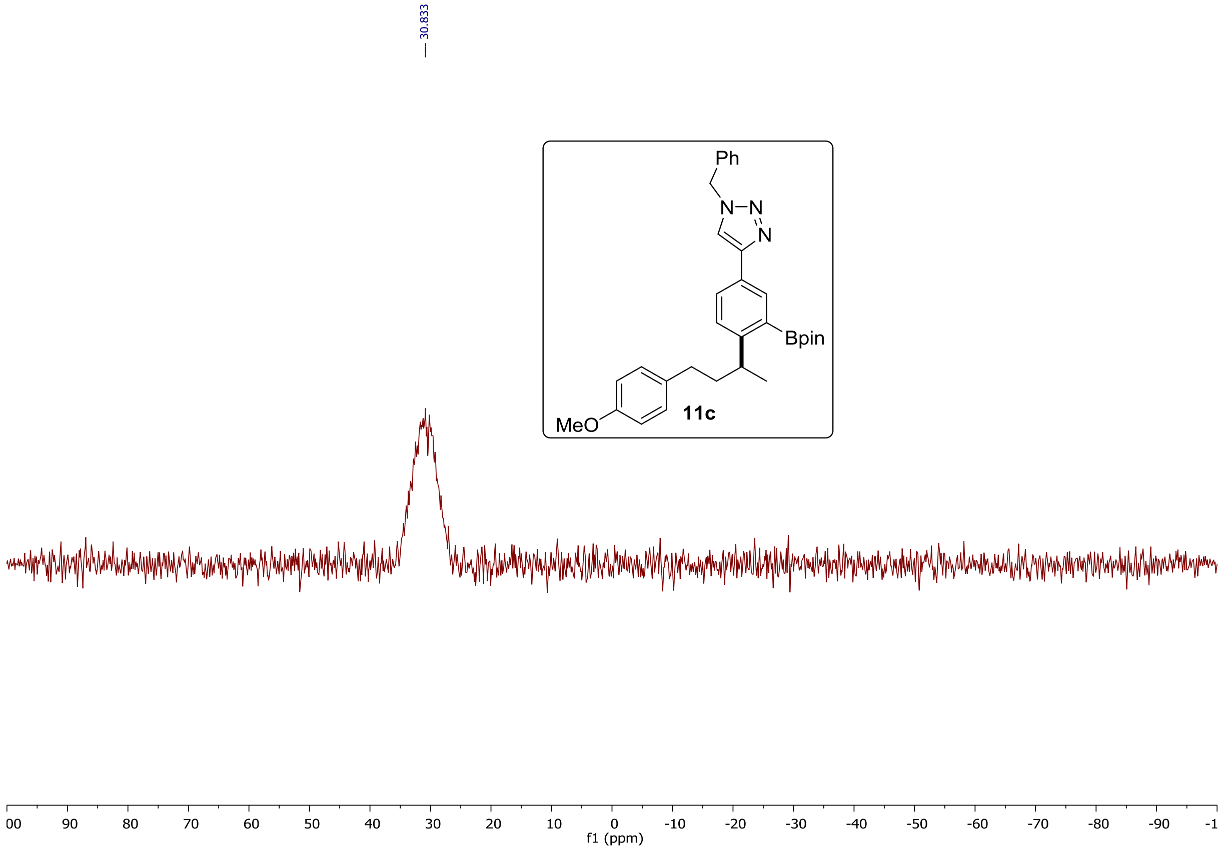
30.833
Ph
N
Bpin
MeO
11c
f1 (ppm)

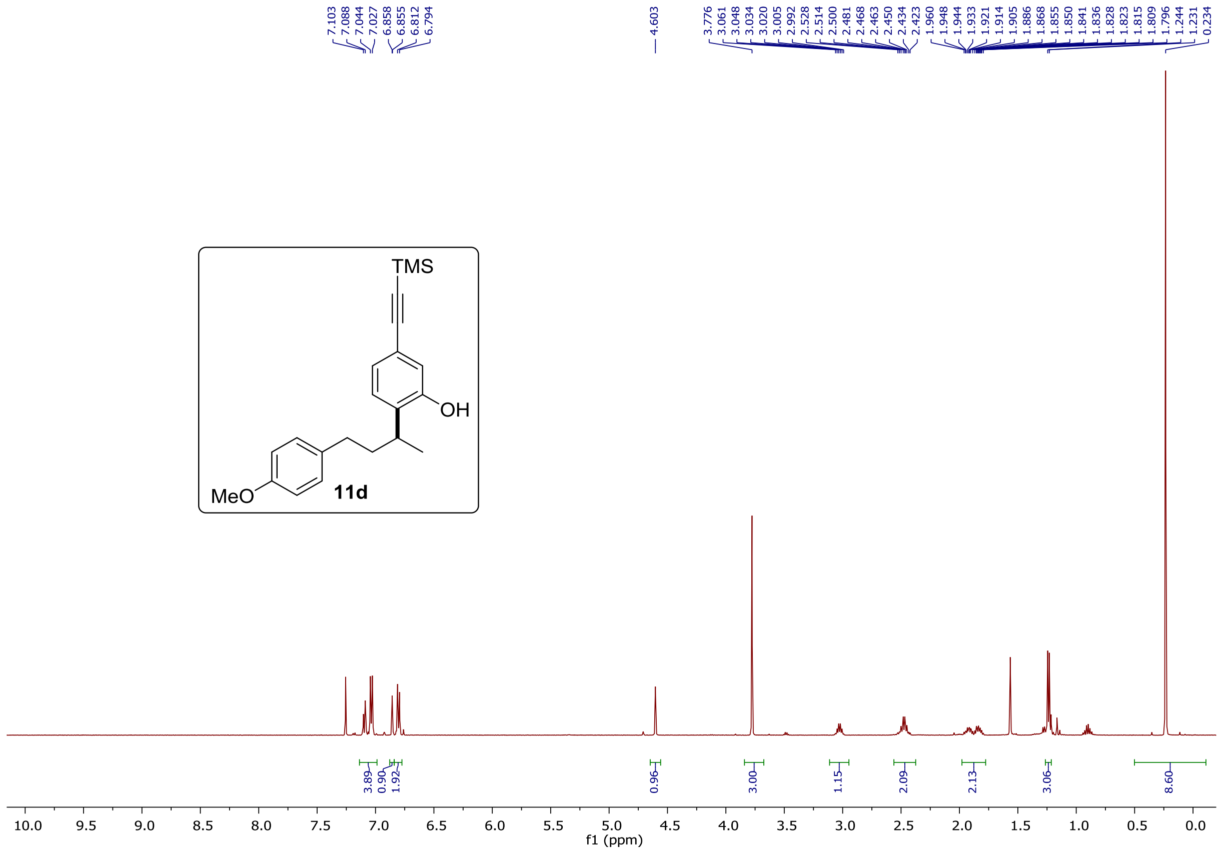
7.103
7.088
7.044
7.027
6.858
6.855
6.812
6.794
4.603
3.776
3.061
3.048
3.034
3.020
3.005
2.992
2.528
2.514
2.500
2.481
2.468
2.463
2.450
2.434
2.423
1.960
1.948
1.944
1.933
1.921
1.914
1.905
1.886
1.868
1.855
1.850
1.841
1.836
1.828
1.823
1.815
1.809
1.796
1.244
1.231
0.234
TMS
OH
MeO
11d
3.89
0.90
1.92
0.96
3.00
1.15
2.09
2.13
3.06
8.60
f1 (ppm)

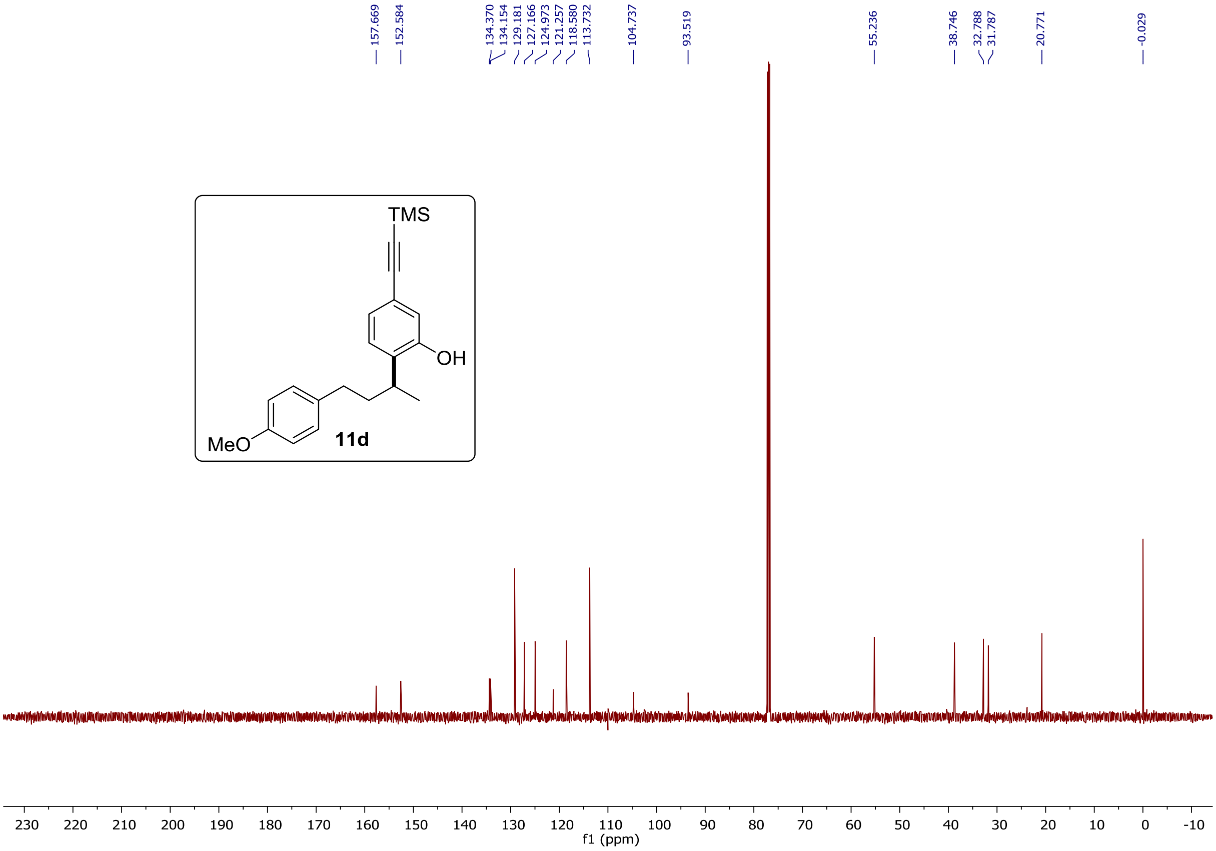
157.669
152.584
134.370
134.154
129.181
127.166
124.973
121.257
118.580
113.732
104.737
93.519
55.236
38.746
32.788
31.787
20.771
-0.029
TMS
OH
MeO
11d
230 220 210 200 190 180 170 160 150 140 130 120 110 100 90 80 70 60 50 40 30 20 10 0 -10
f1 (ppm)

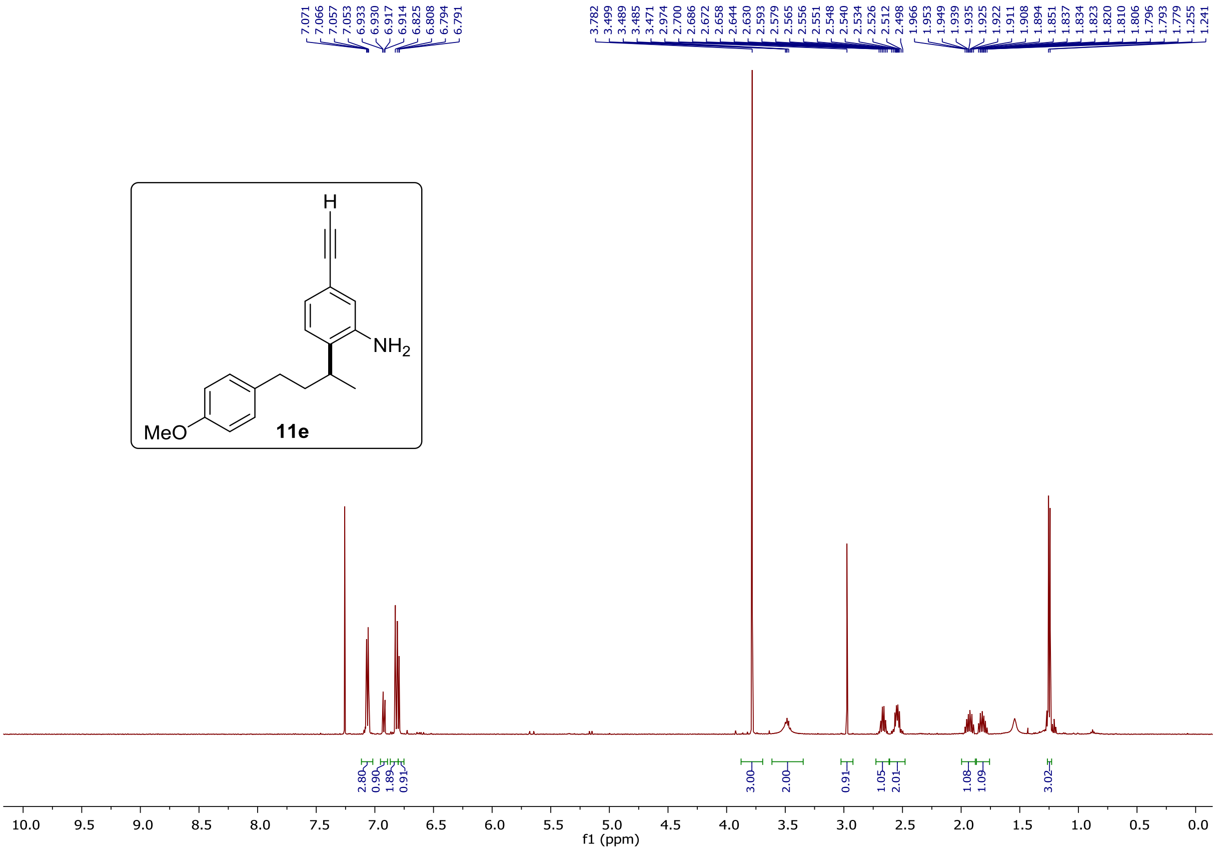
7.071
7.066
7.057
7.053
6.933
6.930
6.917
6.914
6.825
6.808
6.794
6.791
3.782
3.499
3.485
3.471
2.974
2.700
2.686
2.672
2.658
2.644
2.630
2.593
2.579
2.565
2.556
2.551
2.548
2.540
2.534
2.526
2.512
2.498
1.966
1.953
1.949
1.939
1.935
1.925
1.922
1.911
1.908
1.894
1.851
1.837
1.834
1.823
1.820
1.810
1.806
1.796
1.793
1.779
1.255
1.241
H
NH2
MeO
11e
2.80
0.90
1.89
0.91
3.00
2.00
0.91
1.05
2.01
1.08
1.09
3.02
10.0 9.5 9.0 8.5 8.0 7.5 7.0 6.5 6.0 5.5 5.0 4.5 4.0 3.5 3.0 2.5 2.0 1.5 1.0 0.5 0.0
f1 (ppm)

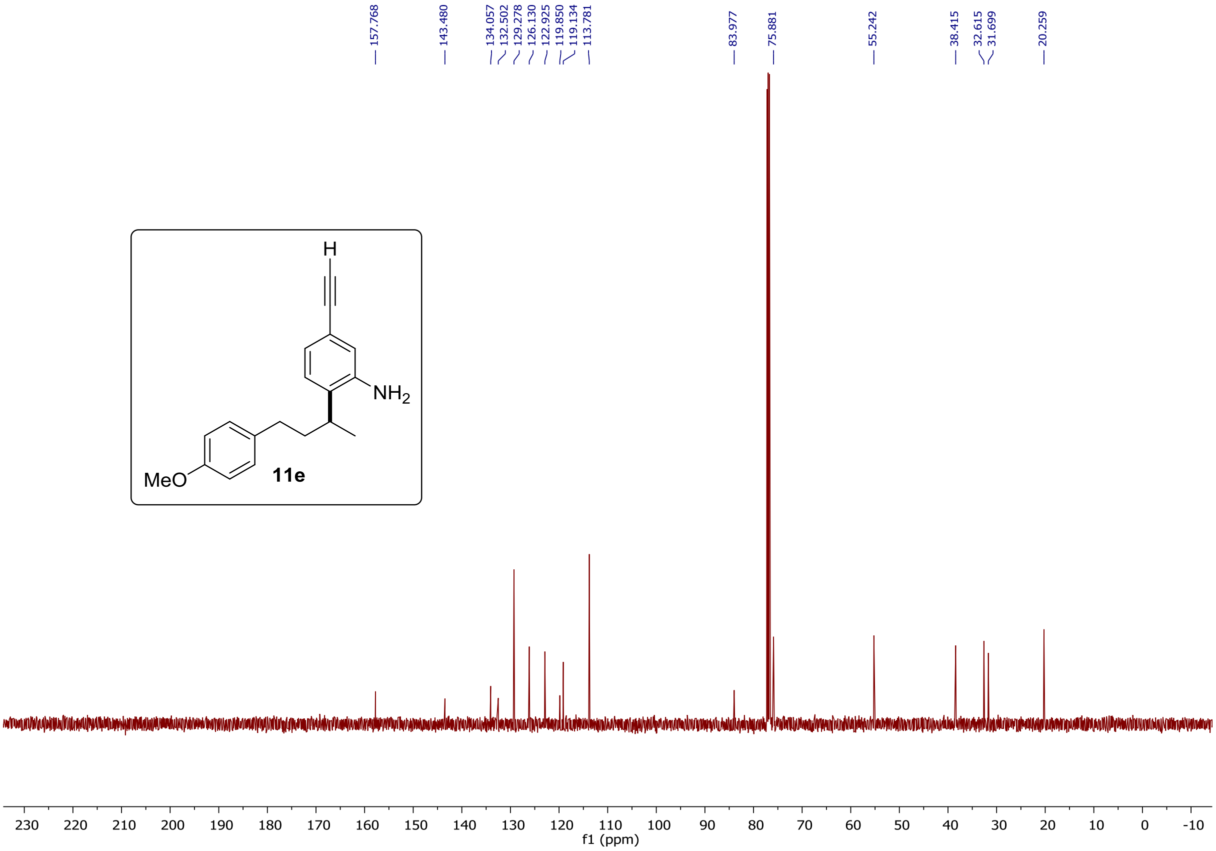
157.768
143.480
134.057
132.502
129.278
126.130
122.925
119.850
119.134
113.781
83.977
75.881
55.242
38.415
32.615
31.699
20.259
H
NH2
MeO
11e
f1 (ppm)

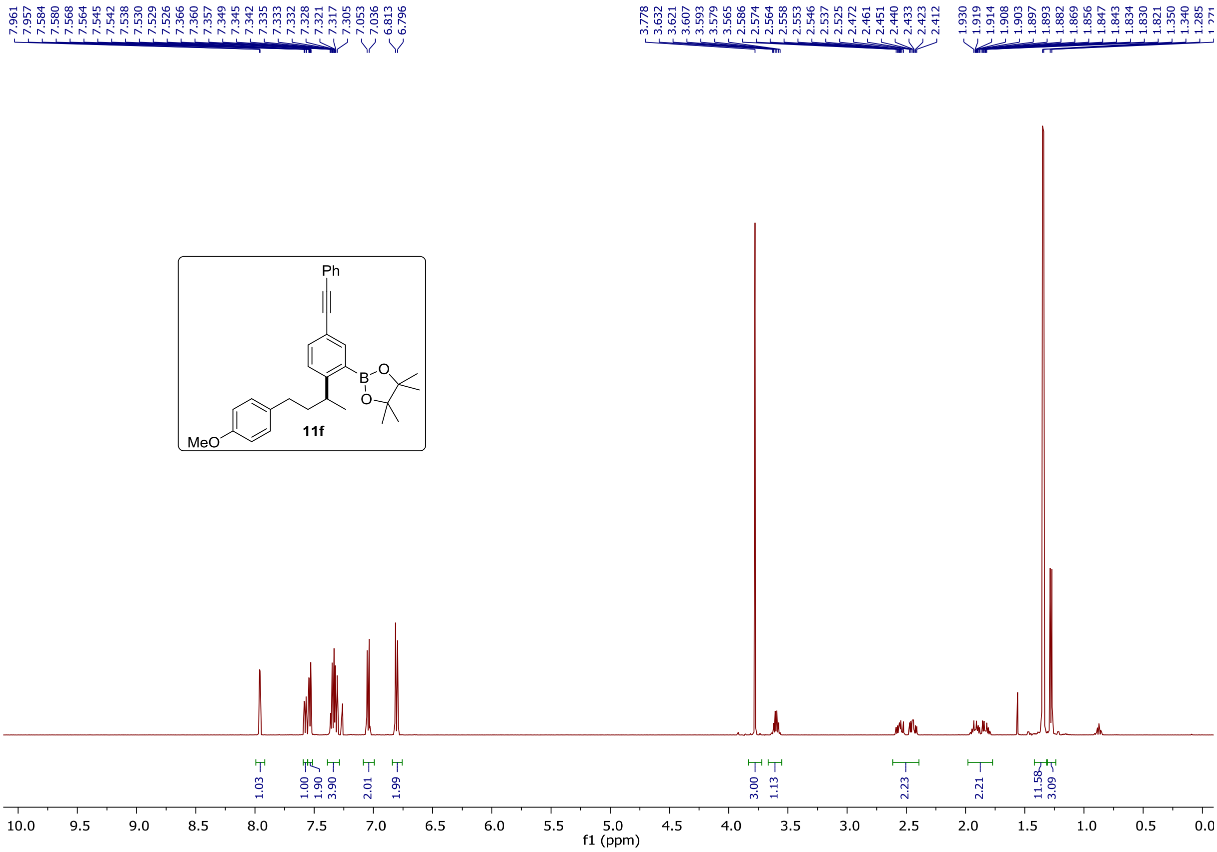
7.961 7.957 7.584 7.580 7.568 7.564 7.545 7.542 7.538 7.530 7.529 7.526 7.366 7.360 7.357 7.349 7.345 7.342 7.335 7.333 7.332 7.328 7.321 7.317 7.305 7.053 7.036 6.813 6.796
3.778 3.632 3.621 3.607 3.593 3.579 3.565 2.586 2.574 2.564 2.558 2.553 2.546 2.537 2.525 2.472 2.461 2.451 2.440 2.433 2.423 2.412
1.930 1.919 1.914 1.908 1.903 1.897 1.893 1.882 1.869 1.856 1.847 1.843 1.834 1.830 1.821 1.350 1.340 1.285
Ph
B
O
O
MeO
11f
1.03
1.00
1.90
3.90
2.01
1.99
3.00
1.13
2.23
2.21
11.58
3.09
f1 (ppm)

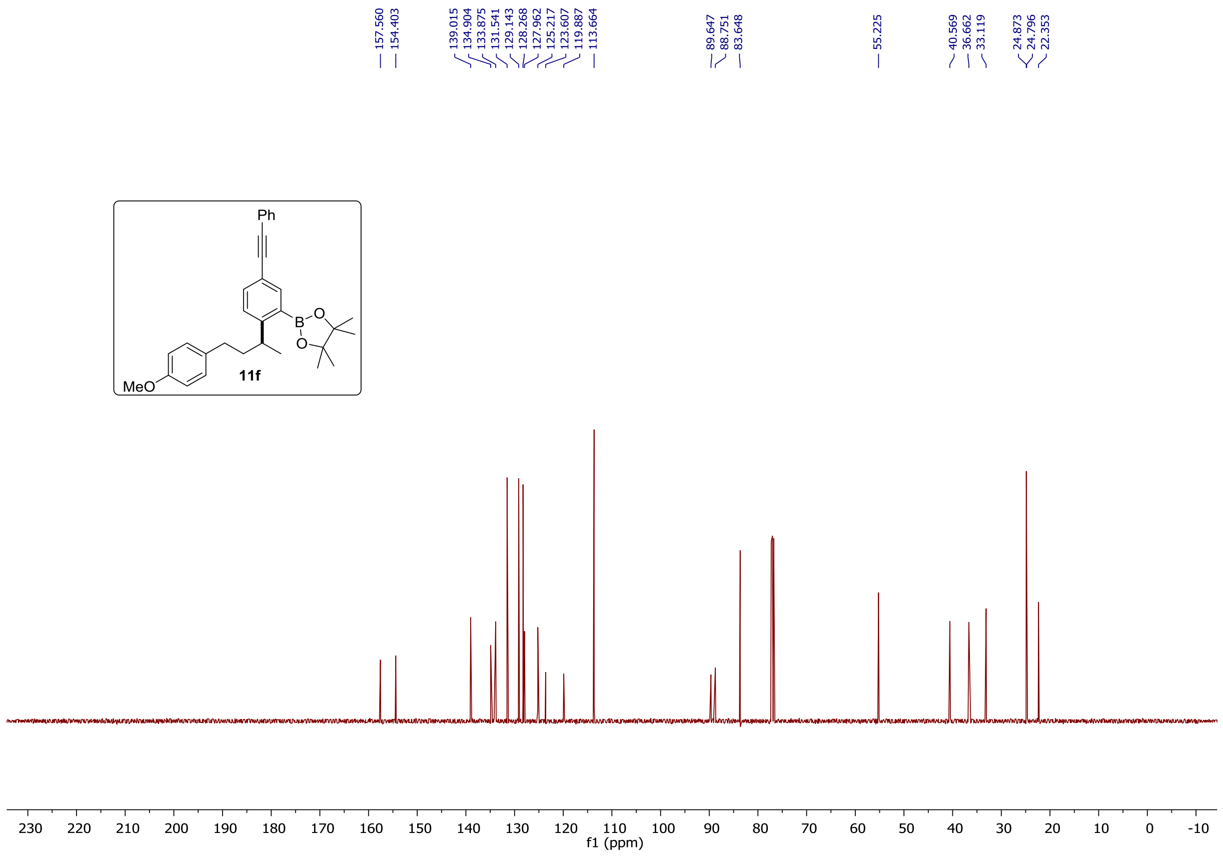
157.560
154.403
139.015
134.904
133.875
131.541
129.143
128.268
127.962
125.217
123.607
119.887
113.664
89.647
88.751
83.648
55.225
40.569
36.662
33.119
24.873
24.796
22.353
Ph
MeO
11f
f1 (ppm)

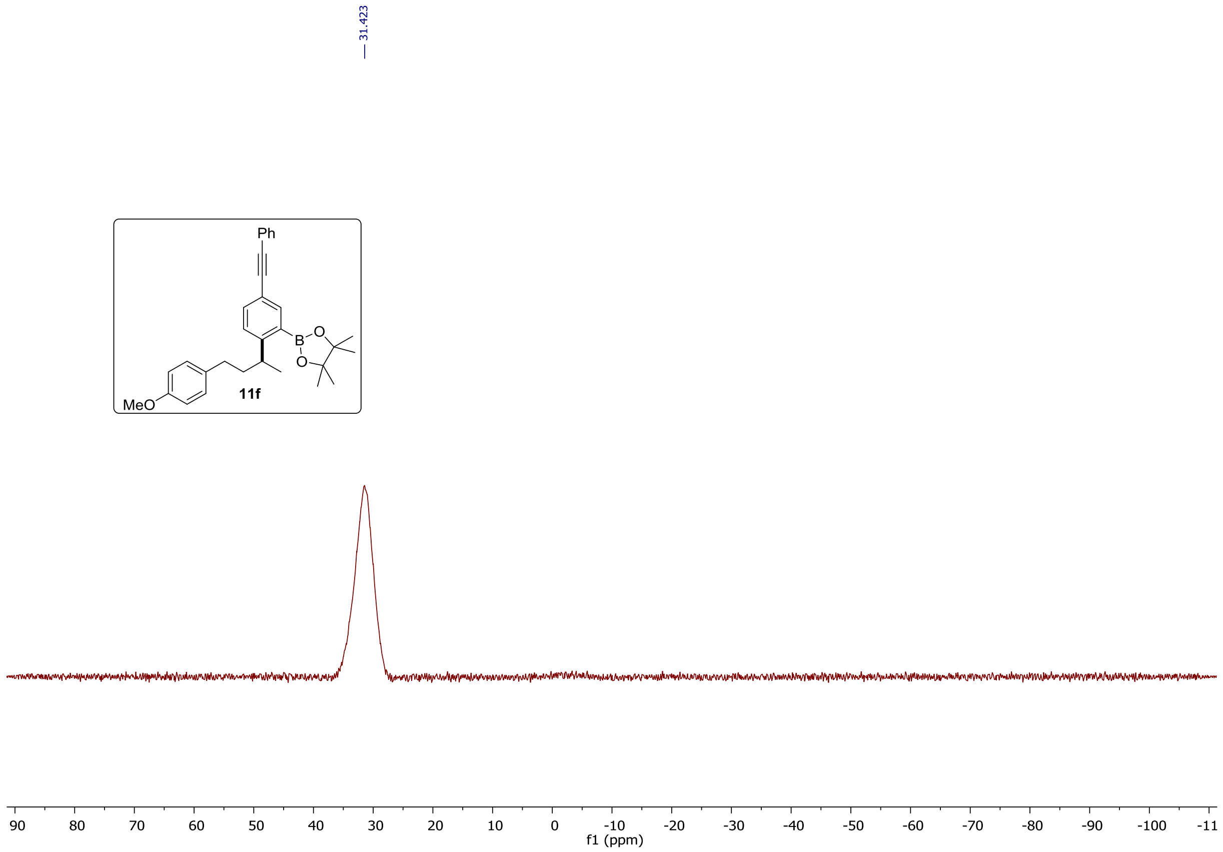
31.423
Ph
MeO
11f
f1 (ppm)

[1] X. Lou, Z.–Q. Zhang, J.–H. Liu, X.–Y. Lu, *Chemistry Letters* **2015**, *45*, 200–202.
[2] R. Larouche–Gauthier, T. G. Elford, V. K. Aggarwal, *J. Am. Chem. Soc.* **2011**, *133*, 16794–16797.
[3] C. Sandford, R. Rasappan, V. K. Aggarwal, *J. Am. Chem. Soc.* **2015**, *137*, 10100–10103.
[4] J. L. Stymiest, G. Dutheuil, A. Mahmood, V. K. Aggarwal, *Angew. Chem. Int. Ed.* **2007**, *46*, 7491–7494.
[5] R. Larouche–Gauthier, C. J. Fletcher, I. Couto, V. K. Aggarwal, *Chem. Commun.* **2011**, *47*, 12592–12594.
[6] A. P. Pulis, D. J. Blair, E. Torres, V. K. Aggarwal, *J. Am. Chem. Soc.* **2013**, *135*, 16054–16057.
[7] K. Kubota, Y. Watanabe, H. Ito, *Adv. Synth. Catal.* **2016**, *358*, 2379–2384.
